# A Regioselective Approach to Accessing Trisubstituted Pyrrolo[2,3-d][1,2,3]triazoles via an Orthogonal Protection Strategy

**DOI:** 10.3390/molecules31142509

**Published:** 2026-07-17

**Authors:** Kévin Brugemann, Simon Garnier, Johnny Vercouillie, Sylvain Routier, Frédéric Buron

**Affiliations:** 1 Institut de Chimie Organique et Analytique, ICOA, Université d’Orleans, CNRS UMR 7311, Rue de Chartres, BP 6759, 45067 Orléans, France; 2 iBrain, INSERM, UMR 1253, Université de Tours, 37032 Tours, France

**Keywords:** pyrrolo[2,3-d][1,2,3]triazoles, multicomponent cyclization, *N*-arylation

## Abstract

The first access to *N*-2, *N*-4-disubstituted pyrrolo[2,3-d][1,2,3]triazoles is reported herein. The series was generated using an orthogonal protecting group strategy from N-1, N-4-disubtituted pyrrolo[2,3-d][1,2,3]triazoles. N-Arylation by Ullman–Goldberg cross-coupling was achieved to explore the reactivity of the pyrrole core and to design N-4-substituted pyrrolo[2,3-d][1,2,3]triazole derivatives. Next, a transition-catalyst-free N-arylation methodology was implemented to achieve N-2 arylation of the triazole moiety. Each arylation strategy was optimized, and a representative library of various aryl iodines or bis-aryl iodonium salts was employed to establish the scope and limitations of each method.

## 1. Introduction

Heterocyclic compounds containing nitrogen atoms have long played a pivotal role in medicinal chemistry due to their structural diversity and wide range of biological activities [1,2,3,4,5]. Among them, pyrroles [6,7,8,9] and triazoles [10,11,12,13] have attracted significant attention as privileged scaffolds in organic chemistry, drug discovery and chemical biology [14,15,16]. For these reasons, their inclusion in medicinal chemistry programs has increased, particularly in the context of molecular diversity and the exploration of innovative chemical space [17]. These two small heterocycles have been fused into bicyclic systems, providing a set of building blocks for medicinal chemists to furnish a large panel of drugs used in various pathologies, including cancer [18,19,20,21,22], tuberculosis [23,24], and analgesia [25,26,27]. However, the fusion of a pyrrole and a triazole ring system to make pyrrolotriazoles is poorly described in the literature, and only one example of these [5:5] fused structures, designed by Cirrincione et al. to generate benzylated pyrrolo[2,3-*d*][1,2,3]triazoles, has been reported [28]. Currently, this method, the only one available, is not suitable for easily introducing the desired substituents at the *N*-2, *N*-4, *C*-5, and *C*-6 positions. This lack of methodology creates a gap in the exploration of the chemical space and prompted us to develop novel and efficient strategies to acquire polyfunctionalized pyrrolo[2,3-*d*][1,2,3]triazole derivatives.

**Scheme 1:**
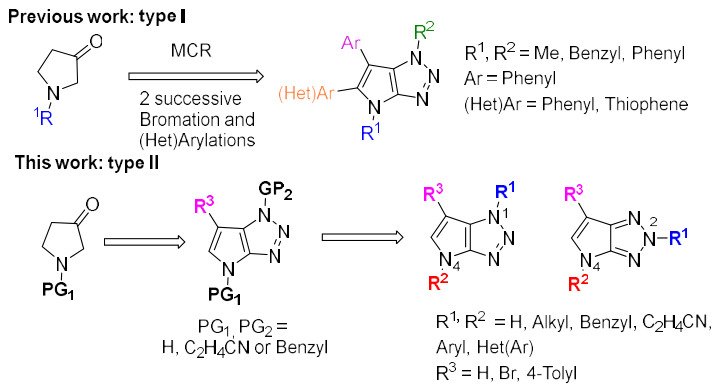
Acquisition of trisubstituted pyrrolo[2,3-*d*][1,2,3]triazoles.

Recently, we reported an efficient method of obtaining original tetra-substituted pyrrolo[2,3-*d*][1,2,3]triazoles corresponding to type **I** using a “one-pot” multicomponent reaction (MCR) followed by functionalization of the *C*-5 and *C*-6 positions through regioselective halogenation and Suzuki–Miyaura coupling reactions [29]. Given the high value of *N*-substituted triazoles or pyrroles for pharmaceutical applications, a new challenge has emerged: to develop a method with which to acquire *N*-arylated derivatives using versatile platforms in order to attain highly diversified structures in a minimal number of steps. Herein, we report our progress and an efficient way to prepare a library of *N*-2 and *N*-4 diarylated pyrrolo[2,3-*d*][1,2,3]triazoles corresponding to type **II** by employing an orthogonal protecting group strategy and copper-catalyzed *N*-arylation (Scheme 1).

## 2. Results

**Scheme 2:**
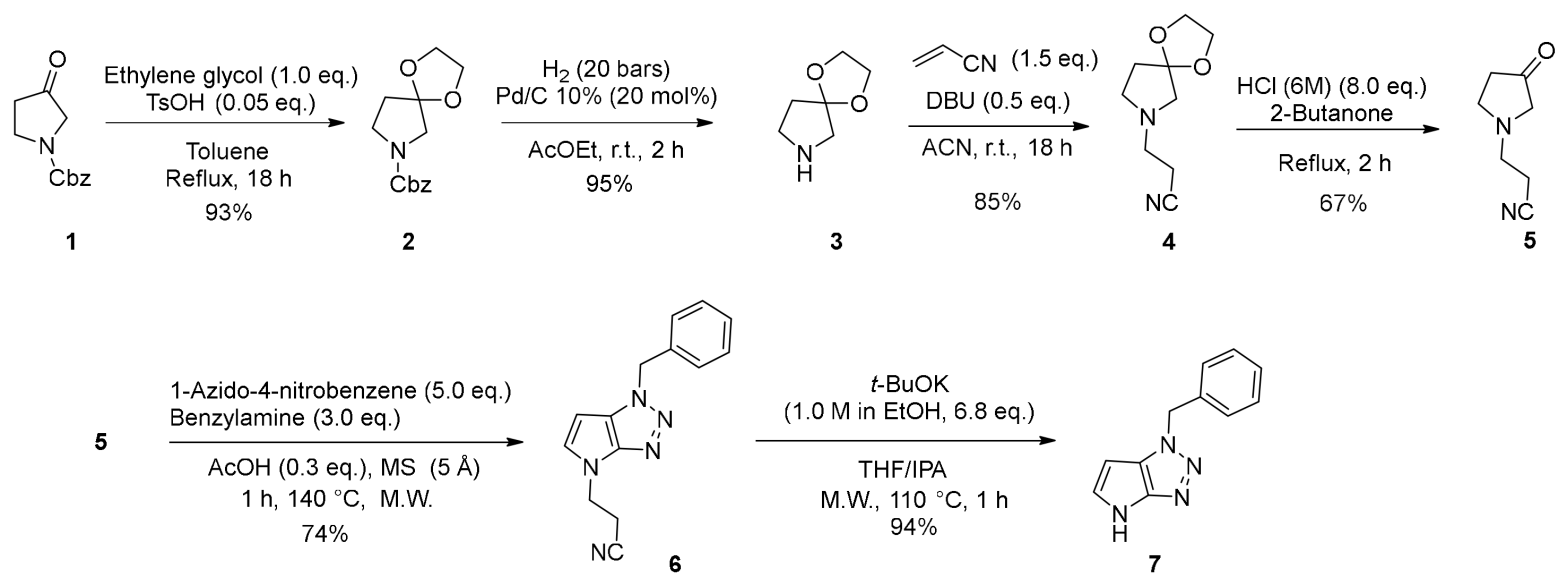
Synthesis of platform **7**.

In order to design a versatile platform that could be modified at the pyrrole NH position of the bicyclic scaffold, the synthesis of a 3-pyrrolidinone substituted by a propionitrile group was considered. The commercially available *N*-Cbz-3-pyrrolidinone ketone **1** was protected with an acetal group to give **2** in a 93% yield. The designed molecule underwent high-pressure hydrogenation for two hours to give **3** in a 95% yield. Secondary amine **3** reacted via an aza-Michael reaction with acrylonitrile to give compound **4**. The acetal was then removed with hydrochloric acid to give pyrrolidinone **5** in a 50% overall yield over four steps. Next, we engaged the pyrrolidinone **5** in a “one-pot” MCR with benzylamine in the presence of 4-nitrophenylazide and acetic acid as a catalyst under microwave irradiation for 1 h at 140 °C. Using these conditions, the desired product **6** was isolated in a 74% yield (Scheme 2). Finally, deprotection of the *N*-cyanoethyl moiety was achieved by a retro aza-Michael mechanism with *t*-BuOK to furnish **7** in a 94% yield [30].

With this compound in hand, we then performed *N*-arylation by Ullman–Goldberg cross-coupling to explore the reactivity of the pyrrole core to design *N*-4 substituted pyrrolo[2,3-*d*][1,2,3]triazole derivatives [31,32,33,34]. This objective prompted us to find a general and efficient catalytic system by optimizing the main reaction parameters (Table 1). First, using **7** as the starting material in the presence of 4-iodotoluene, we modified the catalyst, ligand, base, and solvent. Our first reaction was performed with 10 mol% catalyst (CuI), along with 20 mol% ligand (*L*-Proline) under microwave irradiation for only 30 min. at 110 °C. The desired product **8** was isolated in a low but encouraging 22% yield (Table 1, entry 1). An increase in temperature to 140 °C improved reactivity, and **8** was obtained in a 78% yield (entry 2). It should be noted that degradation was observed at higher temperatures (entry 3). The base was then switched to Na_2_CO_3_ or Cs_2_CO_3_, but no improvement in reactivity was observed (entries 4 and 5). Next, we investigated the influence of the catalyst with the use of CuI or copper(I) thiophene-2-carboxylate (CuTC). While this modification induced a slight decrease in yield with CuI, the reactivity was boosted by the CuTC, and product **8** was isolated in a good yield of 83% (entry 7). Finally, the nature of the solvent or a decrease in 4-iodotoluene did not lead to improved yields.

With these optimized conditions in place, several *N*-4 arylated compounds (**8**–**21**) were obtained by treating **7** with a variety of iodoaryls (Table 2). The *C*-*N* bond was efficiently generated, and products were isolated in good yields ranging from 70% to 83% (Table 2), except in the case of the use of 4-hydroxyphenyl, which partially inhibited reactivity (compound **15**). This limitation, due to the presence of an acidic proton, was easily negated using a THP protective group (compounds **15** and **16**) and subsequent deprotection in acid. Steric hindrance generated by the presence of a methyl group in the ortho vs. meta or para positions (compounds **8**, **11** and **12**) induced a slight decrease in yield. Switching to different heterocycles was studied with 2- or 3-thiophene, and reaction efficiency was sensitive to the positional isomers in favor of the *C*-3 isomer (76% versus 53%). Finally, the use of an iodoalkane was also studied. In our case, direct nucleophilic substitution using a base and a linear iodoalkane inexorably led to an inseparable mixture of compounds due to the presence of several nucleophilic nitrogen atoms on **7**. Using our arylation conditions, we fortunately observed high regioselectivity. Products were generated efficiently and in a regioselective manner, and we isolated compounds **19**–**20** in good yields ranging from 60% to 93. In contrast, the use of a secondary iodoalkane resulted in a decreased yield due to competition with a favorable β H-elimination reaction.

**Scheme 3:**
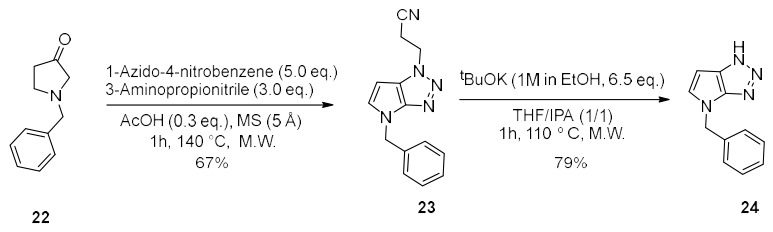
Access to the reactivity of triazole ring **24**.

Next, we focused our efforts on *N*-arylation of the triazole core [35,36]. In order to achieve *N*-2 arylation on the triazole moiety, we synthesized platform **24** using MCR conditions identical to those previously used for **6**, with commercially available *N*-benzylpyrrolidinone **22,** 1-azido-4-nitrobenzene and 3-aminopropionitrile. Triazole **23** was synthesized in a 67% yield; this was followed by deprotection to give **24** in a good yield (Scheme 3).

**Scheme 4:**
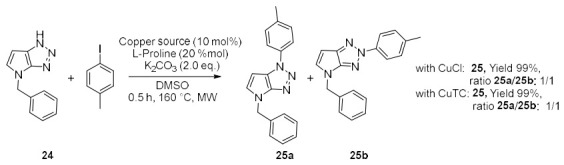
*N*-arylation of **24** using copper catalyst.

With this pyrrolo[2,3-*d*][1,2,3]triazole in hand, *N*-arylation was performed with the optimized copper-catalyzed conditions. Despite the excellent reactivity of this catalytic system, leading to quantitative conversion of the starting material regardless of which copper species we used, we systematically observed a competition between arylation of the two triazole nitrogens with the consequent isolation of the two regioisomers **25a** and **25b** in equivalent quantities (in a ratio of 1:1) without any reaction in the *N*-3 position (Scheme 4).

These results prompted us to switch to a transition-metal-catalyst-free *N*-arylation methodology that readily allows *N*-2 arylation of the triazole moiety. Recently, Prakash et al. reported an eco-friendly regiospecific *N*-arylation of amines using diaryl iodonium salts without the use of costly and toxic transition metal catalysts [37,38,39,40]. We first focused our attention on the reactivity of **24** with the commercially available tolyl(2,4,6-trimethoxyphenyl)iodonium trifluoroacetate in the presence of Na_2_CO_3_ at 100 °C over 1 h (Table 3, entry 1). These conditions led to a mixture of *N*-1 and *N*-2 arylated triazoles **25a** and **25b** in a 76% yield and unprecedented regioselectivity in favor of the *N*-2 isomer **25b** (ratio *N*-1/*N*-2 = 13/87). Under microwave activation, the reaction was complete in 1 h with comparable selectivity if the reaction temperature was limited to 150 °C, but with a drastic decrease in overall yield (entries 2–5). When the base was switched for potassium or cesium carbonate, reactivity improved, unlike with K_3_PO_4_ (entries 6–8). The best result was achieved with K_2_CO_3_ with excellent regio selectivity for the *N*-2 position (ratio *N*-1/*N*-2 11:89). Finally, increasing the amount of iodonium salt to 2.0 equivalents gave the best compromise between efficiency and selectivity (81%, ratio *N*-1/*N*-2: 17/83).

With the optimized conditions in place, we investigated the efficacy of this *N*-2-arylation with various aryl(2,4,6-trimethoxyphenyl)iodonium TMP iodonium salts (aryl TMP iodonium salts) and **24** (Table 4). The reaction proceeded with high reactivity with diaryl iodonium salts containing electron-donating or neutral substituents such as an H or OCH_3_ in the para position, affording the corresponding *N*-2 substituted products **26**–**27** in high yields (81% and 82%, respectively). Concerning the regioselectivity, the strongly electron-donating methoxy group led to a drastic reduction in selectivity relative to an inductive donor substituent such as a methyl (**27**, *N*-1/*N*-2: 1/3 vs. **25**, *N*-1/*N*-2: 17/83). The presence of an ortho methyl substituent led to a slight decrease in yield (**29**, 49%), while a meta methyl group led to the desired compound **28** in a 72% yield (Table 4). Whatever the position, the assay exhibited the same selectivity ratio of 83/17 for *N*-2/*N*-1 isomers. The presence of electron-withdrawing substituents dramatically decreased reaction efficiency, and compounds **30**–**32** were isolated in only 36%, 23% and 19% yields, respectively. This was countered by perfect regioselectivity in the *N*-2-arylated and hetero-arylated products **31b** and **32b**. For these last two reactions, the regioisomers **31a** and **32a** were solely detected in trace amounts by NMR analysis in the crude material.

**Scheme 5:**
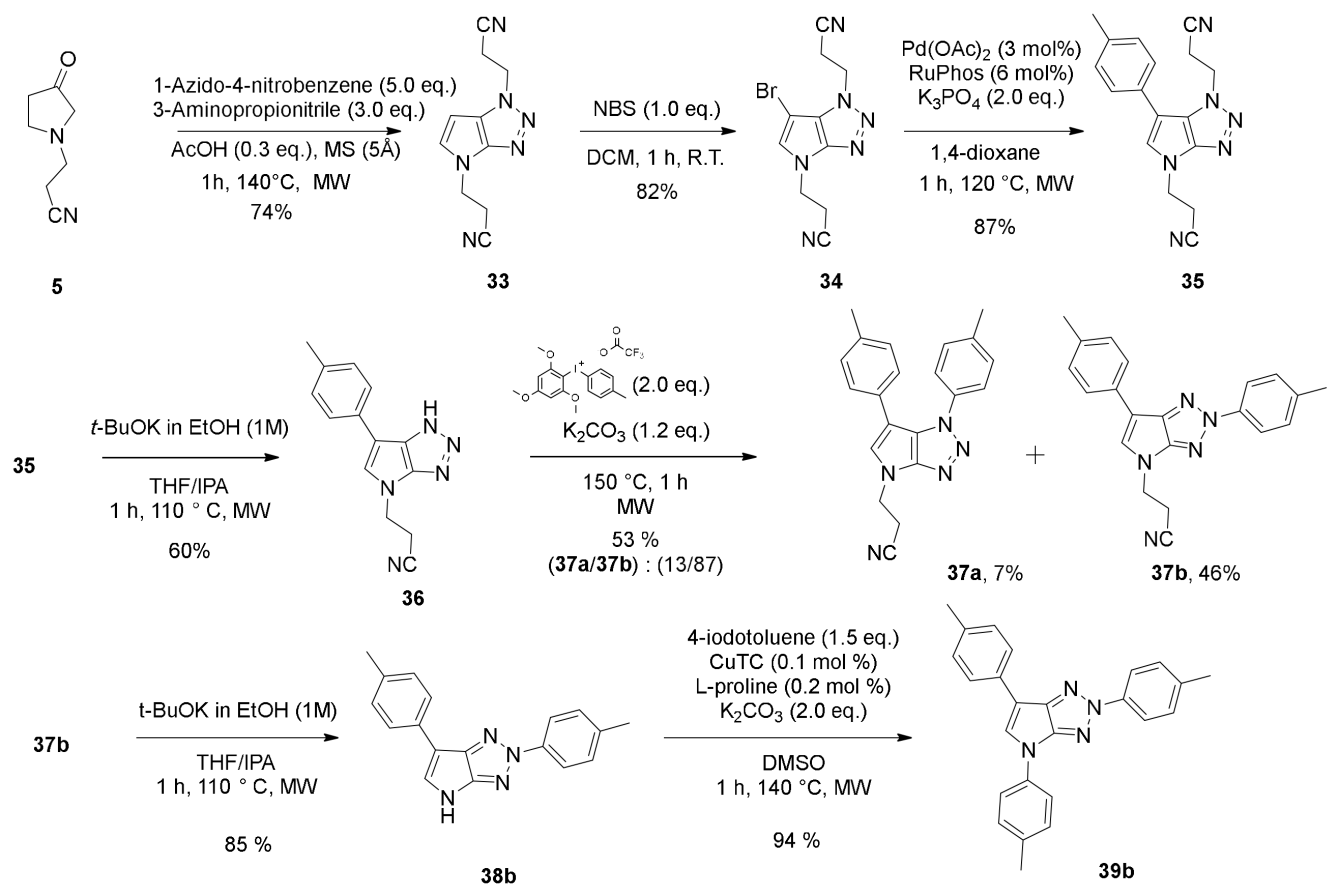
Selective modulation of triazole **33** and then *N*-4 arylation of the pyrrole ring.

With the pyrrole and triazole reactivity studies completed, we turned our attention to the development of a versatile molecule with which to acquire *N*-2, *N*-4 diarylated pyrrolo[2,3-*d*][1,2,3]triazole derivatives. To achieve this goal, two propionitrile groups in the *N*-1 and *N*-4 positions were introduced using the previously developed MCR via the use of pyrrolidinone **5** and aminopropionitrile to give **33** in a 74% yield (Scheme 5). Due to the volatility of the pyrrolo[2,3-*d*][1,2,3]triazole **33**, we directly employed a regioselective bromination/Suzuki–Miyaura sequence to generate compound **35** in a good yield. The selective deprotection in position *N*-1 was carried out in the presence of *t*-BuOK in a mixture THF/IPA at 110 °C under microwave irradiation to produce **36** in a 60% yield. This monodeprotection could be explained by a retro aza-Michael mechanism and the more stable delocalized anion generated by the triazole ring. The *N*-2 arylation with tolyl(TMP)iodonium salt gave solely **37b** in a 46% isolated yield (**37a** was only detected by ^1^H NMR analysis). Finally, the second protecting group was removed, and this was followed by *N*-arylation with a copper catalyst to give the desired compound **39b** in a good overall yield of 10% over seven steps.

**Scheme 6:**
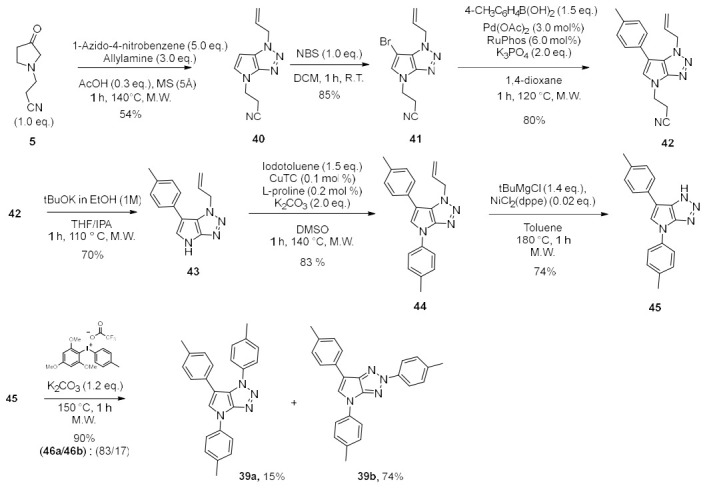
Selective modulation of pyrrole **43** and then *N*-2 arylation of triazole **45**.

To demonstrate the versatility of our strategy, we next investigated a synthetic pathway with a reverse *N*-arylation sequence (Scheme 6). To achieve this goal, an orthogonal protecting group strategy was employed using an allyl group on the *N*-1 triazole nitrogen atom and a propionitrile to mask the pyrrole NH. Having synthesized **42** with the previously developed synthetic pathway, we carried out the selective deprotection of pyrrole using *t*-BuOK under microwave irradiation to furnish **43** in a 70% yield. The copper-catalyzed *N*-arylation of **43** with 4-iodotoluene occurred in a good yield. Finally, de-allylation of the triazole core was completed by an Ni-H catalyst generated in situ by the reaction of NiCl_2_(dppe) with *t*-BuMgCl in toluene at 180 °C under microwave activation to give **45** in a 74% yield [41]. Finally, the last arylation in the *N*-2 position proceeded with an excellent conversion and high selectivity (ratio *N*-1/*N*-2: 17/83) for product **39b**, which was isolated in a 74% yield.

## 3. Materials and Methods

### 3.1. General Information

^1^H NMR and ^13^C NMR spectra were recorded on a Bruker DPX 400 Mhz instrument (Bruker, Champs sur Marne, France) using CDCl_3_ and DMSO-*d*_6_. The chemical shifts are reported in parts per million (*δ* scale), and all coupling constant (*J*) values are reported in hertz. The following abbreviations were used for the multiplicities: s (singlet), d (doublet), t (triplet), q (quartet), p (pentuplet), m (multiplet), sext (sextuplet), and dd (doublet of doublets). All compounds were characterized using ^1^H NMR and ^13^C NMR (Supplementary Materials). Melting points were determined on a Kofler bench apparatus (Servilab, Le Mans, France) and are uncorrected. IR absorption spectra were obtained on a PerkinElmer PARAGON 1000 PC (Waltham, MA, USA), and the values are reported in inverse centimeters. High-resolution mass spectra were performed on a Bruker Q-TOF MaXis mass spectrometer (Bruker Daltonics, Bremen, Germany) using the “Fédération de Recherche” ICOA/CBM (FR2708) platform, and NMR data were generated using the Salsa platform. Monitoring of the reactions was performed using silica gel TLC plates (Kieselgel 60F_254_, E. Merck, Rahway, NJ, USA). Spots were visualized by UV light (254 nm and 356 nm). Column chromatography was performed using silica gel 60 (0.063–0.200 mm, Merck). Microwave irradiation was carried out in sealed vessels placed in a Biotage Initiator or Biotage Initiator+ system (400 W maximum power, Biotage Sweden AB, Uppsala, Sweden). The temperatures were measured externally by IR. Pressure was measured by a non-invasive sensor integrated into the cavity lid. All reagents were purchased from commercial suppliers and were used without further purification.

### 3.2. General Procedure A: Deprotection of Propiocyanate Triazolo and Pyrrolo Protecting Groups of Bicycle Derivatives

In an inert atmosphere, the corresponding propiocyanate-triazolo or propiocyanate-pyrrolo bicycle derivatives (1.0 eq.) were dissolved in a (1/1) mix of dry 2-propanol and dry THF (0.1 M). A 1.0 M solution of potassium tert-butoxide in EtOH (6.8 eq.) was added, and the mixture was stirred for 1 h at 110 °C under microwave irradiation. The resulting mixture was neutralized with a 10% HCl solution until pH = 3 and extracted with EtOAc, and the organic layer was washed with a saturated aqueous sodium chloride solution and then dried over MgSO_4_. After being concentrated under vacuum, the residue was purified by flash chromatography on silica gel, affording the corresponding unprotected products.

### 3.3. General Procedure B: Pyrrole N-Aryl Substitution of 1-Ar-1,4-Dihydropyrrolo[2,3-d][1,2,3]triazole Derivatives

In an inert atmosphere, 1-*R*-1,4-dihydropyrrolo[2,3-*d*][1,2,3]triazole derivative (1.0 eq.), iodoaryl derivative (1.5 eq.), copper(I) thiophene-2-carboxylate (0.1 eq.), *L*-Proline (0.2 eq.) and potassium carbonate (2.0 eq.) were dissolved in dry DMSO (0.25 M), and the mixture was stirred for 30 min at 140 °C under microwave irradiation. After reaction, EtOAc was added to the mixture, and the organic layer was washed first with a saturated solution of NaHCO_3_ and then three times with water and dried over MgSO_4_. After being concentrated under vacuum, the residue was purified by flash chromatography on silica gel, affording the corresponding *N*-arylated derivatives.

### 3.4. General Procedure C: Classical Condition of N-Aryl Substitution of 4-Benzyl-1,4-dihydropyrrolo[2,3-d][1,2,3]triazole

In an inert atmosphere, 4-benzyl-1,4-dihydropyrrolo[2,3-*d*][1,2,3]triazole (1.0 eq.), potassium carbonate (2.0 eq.), iodoaryl derivative (1.5 eq.), copper(I) thiophene-2-carboxylate (0.1 eq.) and *L*-Proline (0.2 eq.) were dissolved in dry DMSO (0.25 M), and the mixture was stirred for 1 h at 140 °C under microwave irradiation. After reaction, water was added, and the mixture was extracted with DCM. Organic layer was washed with saturated aqueous sodium chloride solution and dried over MgSO_4_, and solvents were removed under reduced pressure. Crude product was purified by flash chromatography, affording the corresponding *N*-arylated derivatives.

### 3.5. General Procedure D: Triazole N-Aryl Substitution of 4-Ar-1,4-Dihydropyrrolo[2,3-d][1,2,3]triazole Derivatives

In an inert atmosphere, 4-*R*-1,4-dihydropyrrolo[2,3-*d*][1,2,3]triazole (1.0 eq.), potassium carbonate (1.2 eq.) and aryl(trimethoxyphenyl)iodonium trifluoroacetate derivative (2.0 eq.) were dissolved in dry toluene (0.15 M), and the mixture was stirred for 1 h at 150 °C under microwave irradiation. After reaction, water was added, and the mixture was extracted with DCM. Organic layer was washed with saturated aqueous sodium chloride solution and dried over MgSO_4_, and solvents were removed under reduced pressure. The crude product was purified by flash chromatography, affording the corresponding *N*-arylated derivatives.

### 3.6. General Procedure E: Deprotection of Allyl-Triazolo Protecting Group of Bicycle Derivative

In an inert atmosphere, corresponding allyl-*triazolo* bicycle derivative (1.0 eq.) and [1,2-Bis(diphenylphosphino)ethane]dichloronickel(II) (0.02 eq.) were dissolved in dry toluene (0.15 M). *Tert*-Butylmagnesium chloride, 2.0 M in Et_2_O (2.0 eq.), was added drop by drop, and the reaction mixture was stirred for 1 h at 180 °C under microwave irradiation. After reaction, DCM was added, and the organic layer was successively washed with a saturated solution of sodium bicarbonate, water and saturated aqueous sodium chloride solution. Organic layer was dried over MgSO_4_, and solvents were removed under reduced pressure. Crude product was purified by flash chromatography on silica gel, affording the corresponding unprotected product.

## 4. Conclusions

In summary, a quick means of acquiring original *N*-2, *N*-4 arylated-pyrrolo[2,3-*d*][1,2,3]triazoles has been described herein using an orthogonal protecting group strategy. First, we explored the reactivity of the pyrrole core with a copper-catalyzed *N*-arylation reaction to introduce (Het)aryl moieties with success. Next, a similar study was also carried out with the triazole moiety using an iodonium salt strategy that furnished easy access to *N*-2 arylpyrrolo[2,3-*d*][1,2,3]triazoles in a highly regioselective manner. Finally, versatile platforms containing orthogonal protecting groups for both the pyrazole and triazole moieties were designed to allow sequential and regioselective *N*-arylation with efficiency on either side of the heterocycle. Selective protection of nitrogen atoms led to an arylation of *N*-1 prior to *N*-6 or *N*-6 prior to *N*-1. Therefore, we believe that the work presented here provides a convenient method with which to furnish valuable new biologically active compounds that contain the rare pyrrolo[2,3-*d*][1,2,3]triazole scaffold as the central skeleton.

## Figures and Tables

**Table 1:** Optimization of the *N*-Arylation of **8** in the *N*-4 position.

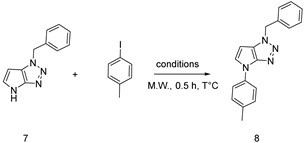							
Entry	4-Iodotoluene (eq.)	Catalyst (10 mol%)	Ligand (20 mol%)	Base (2.0 eq.)	Solvent	T (°C)	Cpd 8 Yield (%) ^1^
1	1.5	CuCl	*L*-Proline	K_2_CO_3_	DMSO	110	22
2	1.5	CuCl	*L*-Proline	K_2_CO_3_	DMSO	140	78
3	1.5	CuCl	*L*-Proline	K_2_CO_3_	DMSO	160	-
4	1.5	CuCl	*L*-Proline	Na_2_CO_3_	DMSO	140	49
5	1.5	CuCl	*L*-Proline	Cs_2_CO_3_	DMSO	140	-
6	1.5	CuI	*L*-Proline	K_2_CO_3_	DMSO	140	73
7	1.5	CuTC	*L*-Proline	K_2_CO_3_	DMSO	140	83
8	1.5	CuTC	-	K_2_CO_3_	DMSO	140	15
9	1.5	CuTC	DMG	K_2_CO_3_	DMSO	140	76
10	1.5	CuTC	*L*-Proline	K_2_CO_3_	DMF	140	73
11	1.1	CuTC	*L*-Proline	K_2_CO_3_	DMSO	140	64

^1^ Isolated yields.

**Table 2:** Synthesis of **8**–**21**.

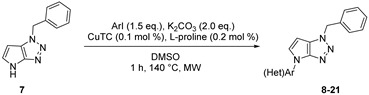				
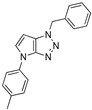	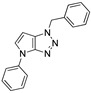	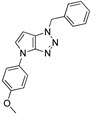	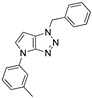	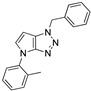
**8**, 83% ^1^	**9**, 76% ^1^	**10**, 82% ^1^	**11**, 75% ^1^	**12**, 70% ^1^
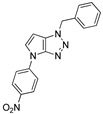	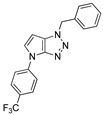	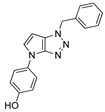	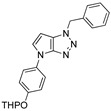	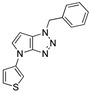
**13**, 78% ^1^	**14**, 78% ^1^	**15**, 28% ^1^ (90% ^1,2^)	**16**, 75% ^1^	**17**, 76% ^1^
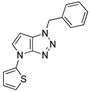	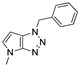	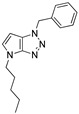	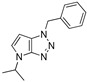	
**18**, 53% ^1^	**19**, 93% ^1^	**20**, 60% ^1^	**21**, 30% ^1^	

^1^ Isolated yields. ^2^ Also obtained via deprotection of **16** in HCl 4 M in 1,4-dioxane, 20 h at r.t.

**Table 3:** Optimization of the selective *N*-Arylation of **24**.

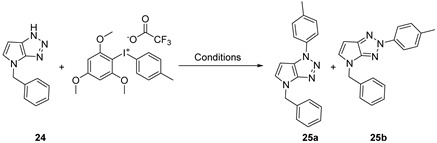							
Entry	Iodonium Salt (eq.)	Base (eq.)	Solvent	T (°C)	Time (h)	25, Yield (%) ^3^	Ratio 25a/25b
1	1.2	Na_2_CO_3_ (1.2 eq.)	Toluene	100 ^1^	24	76	13/87
2	1.2	Na_2_CO_3_ (1.2 eq.)	Toluene	100 ^2^	1	20	13/87
3	1.2	Na_2_CO_3_ (1.2 eq.)	Toluene	150 ^2^	1	48	15/85
4	1.2	Na_2_CO_3_ (1.2 eq.)	Toluene	180 ^2^	1	-	-
5	1.2	Na_2_CO_3_ (1.2 eq.)	Toluene	150 ^2^	2	51	20/80
6	1.2	K_2_CO_3_ (1.2 eq.)	Toluene	150 ^2^	1	64	11/89
7	1.2	Cs_2_CO_3_ (1.2 eq.)	Toluene	150 ^2^	1	71	19/81
8	1.2	K_3_PO_4_ (1.2 eq.)	Toluene	150 ^2^	1	44	23/77
9	2.0	K_2_CO_3_ (1.2 eq.)	Toluene	150 ^2^	1	81	17/83
10	3.0	K_2_CO_3_ (1.2 eq.)	Toluene	150 ^2^	1	86	24/76
11	2.0	K_2_CO_3_ (1.2 eq.)	Toluene	150 ^2^	1	75	14/86
12	2.0	K_2_CO_3_ (1.2 eq.)	DCM	150 ^2^	1	-	-
13	2.0	K_2_CO_3_ (1.2 eq.)	THF	150 ^2^	1		13/87

^1^ Thermal heating. ^2^ Microwave irradiation. ^3^ Isolated yields.

**Table 4:** Synthesis of **25**–**32**.

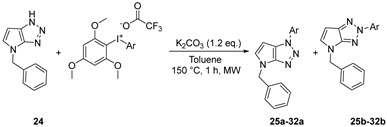			
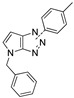	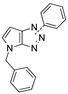	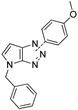	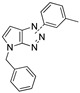
**25a/25b** 81% ^1^ 17/83	**26a/26b** 81% ^1^ 20/80	**27a/27b** 82% ^1^ 25/75	**28a/28b** 72% ^1^ 17/83
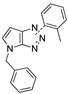	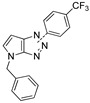	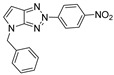	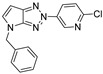
**29a/29b** 49% ^1^ 17/83	**30a/30b** 36% ^1^ 17/83	**31b** 23% ^1,2^	**32b** 19% ^1,2^

^1^ Yields were calculated after isolation of the product. ^2^ Isomers **31a** and **32a** were only detected by NMR analysis.

## Data Availability

All data are presented in the manuscript and the Supplementary Materials.

## Supplementary Materials

The following supporting information can be downloaded at https://www.mdpi.com/article/10.3390/molecules31142509/s1. ^1^H and ^13^C NMR data of all synthesized compounds.
