# Supplementary material for: A Regioselective Approach to Accessing Trisubstituted Pyrrolo[2,3-d][1,2,3]triazoles via an Orthogonal Protection Strategy
Source: Molecules. 2026 Jul 17;31(14):2509. doi: 10.3390/molecules31142509 (PMC13414714; doi:10.3390/molecules31142509)
Supplement: Supplementary file 1 [file molecules-31-02509-s001.zip › molecules-4402508-supplementary.pdf]

A regioselective approach to trisubstituted pyrrolo[2,3-*d*][1,2,3]triazoles via orthogonal protection strategy

Kévin Brugemann†, Simon Garnier†, Johnny Vercouillie, Sylvain Routier*, Frédéric Buron*

Institut de Chimie Organique et Analytique, ICOA, Univ Orleans, CNRS UMR 7311, Rue de Chartres, BP 6759, 45067 Orléans, France

UMR 1253, iBrain, Université de Tours, Inserm, Tours, France

†These authors contributed equally to this work.

TABLE OF CONTENTS

1. **General information……………………………………………………………………………………………………1**
2. **Procedure and Characterizations of compounds 1-48…………………………………………………………2-19**
3. **NMR spectra……20-72**

General Information

Chemicals and analytical grade solvents were purchased from commercial suppliers and used without further purification unless otherwise stated. ^{1}H NMR and ^{13}C NMR spectra were recorded on a Bruker DPX 400 Mhz instrument using $CDCl_3$ and DMSO–d_6. The chemical shifts are reported in parts per million (δ scale), and all coupling constant (J) values are reported in hertz. The following abbreviations were used for the multiplicities: s (singlet), d (doublet), t (triplet), q (quartet), p (pentuplet), m (multiplet), sext (sextuplet), and dd (doublet of doublets). All compounds were characterized by ^{1}H NMR, and ^{13}C NMR (Supplementary Materials). Melting points are uncorrected. IR absorption spectra were obtained on a PerkinElmer PARAGON 1000 PC, and the values are reported in inverse centimeters. HRMS spectra were acquired in positive mode with an ESI source on a Q–TOF mass by the "Fédération de Recherche" ICOA/CBM (FR2708) platform and NMR data were generated on the Salsa platform. Monitoring of the reactions was performed using silica gel TLC plates (silica Merck 60 F 254). Spots were visualized by UV light (254 nm and 356 nm). Column chromatography was performed using silica gel 60 (0.063–0.200 mm, Merck). Reactions requiring anhydrous conditions were performed under argon. Heating blocks were used for conventionally heated reactions. Microwave irradiation was carried out in sealed vessels placed in a Biotage Initiator or Biotage Initiator+ system (400 W maximum power). The temperatures were measured externally by IR. Pressure was measured by a non–invasive sensor integrated into the cavity lid.

Benzyl 1,4-dioxan-7-azaspiro[4.4]nonane-7-carboxylate (2)

To a stirred solution of benzyl 3-oxopyrrolidine-1-carboxylate (7.0 g, 31.9 mmol, 1.0 eq.) in toluene (100 mL), was added ethylene glycol (1.8 mL, 31.9 mmol, 1.0 eq.), and a catalytic amount of *p*-toluenesulfonic acid monohydrate (30 mg, 0.16 mmol, 0.05 eq.). The mixture was refluxed in a Dean-Stark apparatus for 48 hours. After cooling the reaction mixture to room temperature, the mixture was concentrated under reduced pressure. The crude compound was purified by flash column chromatography on silica gel (Petroleum ether (PE)/EtOAc: 70/30). The title product **2** (7.8 g, 93 %) was isolated as a colourless oil. Rf: 0.21 (PE/EtOAc: 70/30). IR (ATR diamond, cm^{-1}) ν: 2886, 1702, 1420, 1354, 1214, 1095, 1016, 948, 768. ^{1}H NMR (400 MHz, Chloroform-*d*): δ 7.39 – 7.27 (m, 5H, H-13, H-14 and H-15), 5.13 (s, 2H, H-11), 4.03 – 3.90 (m, 4H, H-7), 3.60 – 3.52 (m, 2H, H-4), 3.46 (d, *J* = 8.3 Hz, 2H, H-5), 2.09 – 2.00 (m, 2H, H-2). ^{13}C NMR (101 MHz, Chloroform-*d*): δ 155.0 (C_8), 136.9 (C_{12}), 128.6 (2 x C_{14}), 128.1 (C_{15}), 128.0 (2 x C_{13}), 113.7 (d, *J* = 83.3 Hz, C_3), 67.0 (C_{11}), 65.0 (C_7), 53.5 (C_5), 44.5 (d, *J* = 23.1 Hz, C_4), 34.5 (d, *J* = 53.1 Hz, C_2). HRMS (EI^+) *m/z* calculated for $C_{14}H_{18}NO_4$ $[M+H]^+$: 264.1230, found: 264.1236.

1,4-Dioxa-7-azaspiro[4.4]nonane (3)

To a stirred solution of benzyl 1,4-dioxan-7-azaspiro[4.4]nonane-7-carboxylate **2** (6.1 g, 23.17 mmol, 1.0 eq.) in ethanol (150 mL), was added 10% Pd-C (500 mg). The mixture was stirred for 2 hours under a hydrogen atmosphere (20 bar). After reaction, the catalyst was filtered on celite, the filtrate was concentrated under reduced pressure to give the title product **3** (2.83 g, 95 %) was isolated as a colourless oil. Rf: 0.17 (DCM/MeOH: 90/10). IR (ATR diamond, cm^{-1}) ν: 2886, 1634, 1417, 1417, 1258, 1097, 1033, 947, 901, 810. ^{1}H NMR (400 MHz, Chloroform-*d*): δ 3.97 – 3.85 (m, 4H, H-7), 3.04 (t, $J_{4\text{-}5}$ = 7.2 Hz, 2H, H-5), 2.89 (s, 2H, H-2), 2.20 (bs, 1H, N_1-H), 1.95 (t, $J_{4\text{-}5}$ = 7.2 Hz, 2H, H-4). ^{13}C NMR (101 MHz, Chloroform-*d*): δ 117.7 (C_3), 64.7 (C_7), 55.5 (C_2), 46.0 (C_5), 36.9 (C_4). HRMS (EI^+) *m/z* calculated for $C_6H_{12}NO_2$ $[M+H]^+$: 130.0863, found: 130.0863.

3-(1,4-Dioxa-7-azaspiro[4.4]nonane-7-yl)propanenitrile (4)

To a stirred solution of 1,4-dioxa-7-azaspiro[4.4]nonane **3** (2.83 g, 21.91 mmol, 1.0 eq.) in acetonitrile (150 mL), were added acrylonitrile (2.18 mL, 32.87 mmol, 1.5 eq.) and DBU (1.64 mL, 10.96 mmol, 0.5 eq.). The mixture was stirred at room temperature for 18 hours. After the reaction, the mixture was concentrated under reduced pressure. The crude compound was purified by flash column chromatography on silica gel (PE/EtOAc: 50/50). The title product **4** (3.4 g, 85 %) was isolated as a colourless oil. Rf: 0.13 (PE/EtOAc: 50/50). IR (ATR diamond, cm^{-1}) ν: 2926, 2248, 1705, 1683, 1506, 1489, 1418, 1223. ^{1}H NMR (400 MHz, Chloroform-*d*): δ 3.97 – 3.82 (m, 4H, H-7), 2.77 (t, $J_{4\text{-}5}$ = 7.2 Hz, 2H, H-5), 2.75 – 2.69 (m, 4H, H-2 and H-8), 2.51 (t, $J_{4\text{-}5}$ = 7.2 Hz, 2H, H-4), 2.05 (t, $J_{8\text{-}9}$ = 7.0 Hz, 2H, H-9). ^{13}C NMR (101 MHz, Chloroform-*d*): δ 118.7 (C_{10}), 115.4 (C_3), 64.6 (C_7), 62.7 (CH_2), 52.6 (CH_2), 51.4 (C_5), 36.5 (C_9), 17.2 (C_4). HRMS (EI^+) *m/z* calculated for $C_{10}H_{15}N_2O_2$ $[M+H]^+$: 183.1128, found: 183.1130.

3-(3-Oxopyrrolidin-1-yl)propanenitrile (5)

To a stirred solution of 3-(1,4-dioxa-7-azaspiro[4.4]nonane-7-yl)propanenitrile **4** (3.41 g, 18.7 mmol, 1.0 eq.) in butanone (100 mL), was added 6.0 M HCl (25 mL). The mixture was refluxed for 2 hours. After reaction, the mixture was quenched at pH 7 with NaOH saturated in water. The mixture was extracted with DCM (3 x 100 mL). The organic phase was washed with brine (50 mL), dried over $MgSO_4$, filtered and concentrated under reduced pressure. The crude compound was purified by flash column chromatography on silica gel (PE/EtOAc: 50/50). The title product **5** (1.75 g, 67 %) was isolated as a brown oil. Rf: 0.13 (PE/EtOAc: 50/50). IR (ATR diamond, cm^{-1}) ν: 2995, 2109, 1695, 1553, 1506, 1320, 1223, 993, 810. ^{1}H NMR (400 MHz, Chloroform-*d*): δ 3.02 (s, 2H, H-2), 2.99 (t, $J_{4\text{-}5}$ = 7.0 Hz, 2H, H-5),

2.86 (t, $J_{8\text{-}9}$ = 6.8 Hz, 2H, H-8), 2.56 (t, $J_{8\text{-}9}$ = 6.8 Hz, 2H, H-9), 2.43 (t, $J_{4\text{-}5}$ = 7.0 Hz, 2H, H-4). ^{13}C NMR (101 MHz, Chloroform-*d*): δ 212.7 (C_3), 118.4 (C_{10}), 60.9 (CH_2), 51.5 (C_8), 51.3 (CH_2), 38.0 (C_4), 17.2 (C_9). HRMS (EI$^+$) *m/z* calculated for $C_7H_{11}N_2O$ [M+H]$^+$: 139.0866, found: 139.0866.

3-(1-Benzylpyrrolo[2,3-*d*][1,2,3]triazol-4(1*H*)-yl)propanenitrile (6)

In a microwave vial already filled with anhydrous toluene (6 mL) and molecular sieve (3 Å), was successively added 3-(3-oxopyrrolidin1-yl)propanenitrile **5** (207 mg, 1.5 mmol, 1.0 eq.), benzylamine (0.49 mL, 4.5 mmol, 3.0 eq.), 1-azido-4-nitrobenzene (1.23 g, 7.5 mmol, 5.0 eq.) and acetic acid (0.025 mL, 0.45 mmol, 0.3 eq.). The vial was finally capped and stirred 1h at 140°C under microwave irradiation. The resulting mixture was reduced under vacuum and filtered through a pad of charcoal. The crude mixture was purified by flash chromatography on silica gel using first DCM and then (PE/EtOAc: 40/60) to afford **6** as a white solid (250 mg, 67 %). Rf: 0.41 (PE/EtOAc: 40/60). MP: 96–98 °C. IR (ATR diamond, cm^{-1}) ν: 2942, 2251, 1650, 1490, 1366, 1223, 1087, 1051, 941, 723, 692. ^{1}H NMR (400 MHz, Chloroform-*d*): δ 7.42 – 7.29 (m, 5H, H-9, H-10 and H-11), 6.90 (d, $J_{5\text{-}6}$ = 3.2 Hz, 1H, H-5), 5.62 (d, $J_{5\text{-}6}$ = 3.2 Hz, 1H, H-6), 5.60 (s, 2H, H-7), 4.36 (t, $J_{12\text{-}13}$ = 6.6 Hz, 2H, H-12), 3.01 (t, $J_{12\text{-}13}$ = 6.6 Hz, 2H, H-13). ^{13}C NMR (101 MHz, Chloroform-*d*): δ 149.8 (C_{3a}), 134.6 (C_8), 130.7 (C_5), 129.1 (2 x CH_{Ar}), 128.7 (C_{11}), 128.6 (C_{6a}), 128.5 (2 x CH_{Ar}), 117.2 (C_{14}), 89.5 (C_6), 53.9 (C_7), 43.1 (C_{12}), 19.3 (C_{13}). HRMS (EI$^+$) *m/z* calculated for $C_{14}H_{14}N_5$ [M+H]$^+$: 252.1244, found: 252.1244.

1-Benzyl-1,4-dihydropyrrolo[2,3-*d*][1,2,3]triazole (7)

The reaction was carried out as described in general procedure **A** using 3-(1-benzylpyrrolo[2,3-*d*][1,2,3]triazol-4(1*H*)-yl)propanenitrile **6** (50 mg, 0.20 mmol, 1.0 eq.) and 1.0 M solution of potassium *tert*-butoxide in EtOH (1.35 mL, 1.35 mmol, 6.8 eq.). Crude product was purified by flash chromatography on silica gel using a gradient (DCM: MeOH) from (100: 0) to (95: 5) yielding to corresponding product **7** (37 mg, 94 %) as a white solid. Rf: 0.39 (DCM/MeOH: 95/5). MP: 159–161 °C. IR (ATR diamond, cm^{-1}) ν: 3116, 2938, 1518, 1354. ^{1}H NMR (400 MHz, Chloroform-*d*): δ 8.84 (s, 1H, N_4-H), 7.34 (m, 5H, H-9, H-10 and H-11), 7.08 – 6.95 (m, 1H, H-5), 5.76 – 5.65 (m, 1H, H-6), 5.62 (s, 2H, H-7). ^{13}C NMR (101 MHz, Chloroform-*d*): δ 150.4 (C_{3a}), 134.9 (C_8), 129.0 (2 x CH_{Ar}), 128.6 (C_{11}), 128.5 (2 x CH_{Ar}), 128.4 (C_{6a}), 127.9 (C_5), 89.4 (C_6), 53.8 (C_7). HRMS (EI$^+$) *m/z* calculated for $C_{11}H_{11}N_4$ [M+H]$^+$: 199.0978, found: 199.0980.

1-Benzyl-4-(*p*-tolyl)-1,4-dihydropyrrolo[2,3-*d*][1,2,3]triazole (8)

The reaction was carried out as described in general procedure **B** using 1-benzyl-1,4-dihydropyrrolo[2,3-*d*][1,2,3]triazole **7** (50 mg, 0.25 mmol, 1.0 eq.), 1-iodo-4-methylbenzene (80 mg, 0.30 mmol, 1.5 eq.) as iodoaryl derivative, Copper(I) thiophene-2-carboxylate (2.5 mg, 0.013 mmol, 0.1 eq.), *L*-Proline (5.80 mg, 0.05 mmol, 0.2 eq.) and potassium carbonate (160 mg, 0.50 mmol, 2.0 eq.) in dry DMSO (0.25 M). Crude product was purified by flash chromatography on silica gel using a gradient (PE: EtOAc) from (1: 0) to (7: 3) yielding to corresponding product **8** (59 mg, 83 %) as a white solid. Rf: 0.55 (PE/EtOAc: 70/30). MP: 112–114 °C. IR (ATR diamond, cm^{-1}) ν: 3102, 1525, 1344, 1174, 1107, 1085, 815, 745, 696. ^{1}H NMR (400 MHz, Chloroform-*d*): δ 7.67 (d, $J_{13\text{-}14}$ = 8.3 Hz, 2H, H-13), 7.48 – 7.32 (m, 5H, H-9, H-10 and H-11), 7.31 (d, $J_{5\text{-}6}$ = 3.2 Hz, 1H, H-5), 7.26 (d, $J_{13\text{-}14}$ = 8.3 Hz, 2H, H-14), 5.75 (d, $J_{5\text{-}6}$ = 3.2 Hz, 1H, H-6), 5.64 (s, 2H, H-7), 2.37 (s, 3H, H-16). ^{13}C NMR (101 MHz, Chloroform-*d*): δ 149.1 (C_{3a}), 135.8 (C_q), 135.1 (C_q), 134.5 (C_8), 130.3 (2 x C_{14}), 129.4 (C_q), 129.1 (2 x CH_{Ar}), 128.8 (C_{11}), 128.5 (2 x CH_{Ar}), 127.8 (C_5), 118.8 (2 x C_{13}), 90.6 (C_6), 53.9 (C_7), 21.0 (C_{16}). HRMS (EI$^+$) *m/z* calculated for $C_{18}H_{17}N_4$ [M+H]$^+$: 289.1448, found: 289.1450.

1-Benzyl-4-phenyl-1,4-dihydropyrrolo[2,3-*d*][1,2,3]triazole (9)

The reaction was carried out as described in general procedure **B** using 1-benzyl-1,4-dihydropyrrolo[2,3-*d*][1,2,3]triazole **7** (50 mg, 0.25 mmol, 1.0 eq.), iodobenzene (70 mg, 0.30 mmol, 1.5 eq.) as iodoaryl derivative, Copper(I) thiophene-2-carboxylate (2.5 mg, 0.013 mmol, 0.1 eq.), *L*-Proline (5.8 mg, 0.05 mmol, 0.2 eq.) and potassium carbonate (160 mg, 0.50 mmol, 2.0 eq.) in dry DMSO (0.25 M). Crude product was purified by flash chromatography on silica gel using a gradient (PE: EtOAc) from (1: 0) to (7: 3) yielding to corresponding product **9** (52 mg, 76 %) as a white solid. Rf: 0.43 (PE/EtOAc: 70/30). MP: 113–115 °C. IR (ATR diamond, cm^{-1}) ν: 3111, 1523, 1506, 1329, 1175, 1084, 841, 756, 721. ^{1}H NMR (400 MHz, Chloroform-*d*): δ 7.85 (d, J_{13-14} = 7.8 Hz, 2H, H-13), 7.47 (t, $J_{13-14-15}$ = 7.8 Hz, 2H, H-14), 7.41 – 7.31 (m, 6H, H-5, H-9, H-10 and H-11), 7.21 (t, J_{14-15} = 7.8 Hz, 1H, H-15), 5.78 (d, J_{5-6} = 3.4 Hz, 1H, H-6), 5.65 (s, 2H, H-7). ^{13}C NMR (101 MHz, Chloroform-*d*): δ 149.6 (C_{3a}), 138.4 (C_{12}), 134.8 (C_8), 129.8 (2 x C_{14}), 129.3 (C_{6a}), 129.1 (2 x CH_{Ar}), 128.7 (C_{11}), 128.5 (2 x CH_{Ar}), 127.1 (C_5), 125.0 (C_{15}), 118.5 (2 x C_{13}), 91.0 (C_6), 53.8 (C_7). HRMS (EI^+) *m/z* calculated for $C_{17}H_{15}N_4$ $[M+H]^+$: 247.1230, found: 247.1235.

1-Benzyl-4-(4-methoxyphenyl)-1,4-dihydropyrrolo[2,3-*d*][1,2,3]triazole (10)

The reaction was carried out as described in general procedure **B** using 1-Benzyl-1,4-dihydropyrrolo[2,3-*d*][1,2,3]triazole **7** (50 mg, 0.25 mmol, 1.0 eq.), 1-iodo-4-methoxybenzene (70 mg, 0.30 mmol, 1.5 eq.) as iodoaryl derivative, Copper(I) thiophene-2-carboxylate (2.5 mg, 0.013 mmol, 0.1 eq.), *L*-Proline (5.8 mg, 0.05 mmol, 0.2 eq.) and potassium carbonate (160 mg, 0.50 mmol, 2.0 eq.) in dry DMSO (0.25 M). Crude product was purified by flash chromatography on silica gel using a gradient (PE: EtOAc) from (1: 0) to (7: 3) yielding to corresponding product **10** (62 mg, 82 %) as a white solid. Rf: 0.10 (PE/EtOAc: 70/30). MP: 124–126 °C. IR (ATR diamond, cm^{-1}) ν: 3105, 2828, 1523, 1507, 1484, 1297, 1175, 1037, 968, 852, 797, 708. ^{1}H NMR (400 MHz, Chloroform-*d*): δ 7.73 (d, J_{13-14} = 9.0 Hz, 2H, H-13), 7.40 – 7.32 (m, 5H, H-9, H-10 and H-11), 7.26 (d, J_{5-6} = 3.3 Hz, 1H, H-5), 7.00 (d, J_{13-14} = 9.0 Hz, 2H, H-14), 5.74 (d, J_{5-6} = 3.3 Hz, 1H, H-6), 5.65 (s, 2H, H-7), 3.84 (s, 3H, H-17). ^{13}C NMR (101 MHz, Chloroform-*d*): δ 157.2 (C_{15}), 149.6 (C_{3a}), 134.8 (C_8), 132.0 (C_{12}), 129.1 (2 x CH_{Ar}), 129.0 (C_{6a}), 128.7 (C_{11}), 128.5 (2 x CH_{Ar}), 127.5 (C_5), 120.2 (2 x C_{13}), 115.0 (2 x C_{14}), 90.2 (C_6), 55.7 (C_{17}), 53.8 (C_7). HRMS (EI^+) *m/z* calculated for $C_{18}H_{17}N_4O$ $[M+H]^+$: 305.1397, found: 305.1399.

1-Benzyl-4-(*m*-tolyl)-1,4-dihydropyrrolo[2,3-*d*][1,2,3]triazole (11)

The reaction was carried out as described in general procedure **B** using 1-benzyl-1,4-dihydropyrrolo[2,3-*d*][1,2,3]triazole **7** (50 mg, 0.25 mmol, 1.0 eq.), 1-iodo-3-methylbenzene (80 mg, 0.30 mmol, 1.5 eq.) as iodoaryl derivatives, Copper(I) thiophene-2-carboxylate (2.5 mg, 0.013 mmol, 0.1 eq.), *L*-Proline (5.8 mg, 0.05 mmol, 0.2 eq.) and potassium carbonate (160 mg, 0.50 mmol, 2.0 eq.) in dry DMSO (0.25 M). Crude product was purified by flash chromatography on silica gel using a gradient (PE: EtOAc) from (1: 0) to (7: 3) yielding to corresponding product **11** (54 mg, 75 %) as a white solid. Rf: 0.53 (PE/EtOAc: 70/30). MP: 79–81 °C. IR (ATR diamond, cm^{-1}) ν: 3129, 2860, 1541, 1323, 1201, 1172, 1113, 914, 829, 741, 712, 685. ^{1}H NMR (400 MHz, Chloroform-*d*): δ 7.70 (s, 1H, H-13), 7.62 (d, J_{16-17} = 8.1 Hz, 1H, H-17), 7.43 – 7.31 (m, 7H, H-5, H-9, H-10, H-11 and H-16), 7.03 (d, J_{15-16} = 7.6 Hz, 1H, H-15), 5.76 (d, J_{5-6} = 3.4 Hz, 1H, H-6), 5.65 (s, 2H, H-7), 2.44 (s, 3H, H-18). ^{13}C NMR (101 MHz, Chloroform-*d*): δ 149.6 (C_{3a}), 139.9 (C_{14}), 138.3 (C_q), 134.8 (C_q), 129.6 (CH_{Ar}), 129.2 (C_q), 129.1 (2 x CH_{Ar}), 128.7 (CH_{Ar}), 128.5 (2 x CH_{Ar}), 127.2 (C_5), 125.9 (C_{15}), 119.3 (C_{13}), 115.6 (C_{17}), 90.8 (C_6), 53.8 (C_7), 21.7 (C_{18}). HRMS (EI^+) *m/z* calculated for $C_{18}H_{17}N_4$ $[M+H]^+$: 289.1448, found: 289.1452.

1-Benzyl-4-(*o*-tolyl)-1,4-dihydropyrrolo[2,3-*d*][1,2,3]triazole (12)

The reaction was carried out as described in general procedure **B** using 1-benzyl-1,4-dihydropyrrolo[2,3-*d*][1,2,3]triazole **7** (50 mg, 0.25 mmol, 1.0 eq.), 1-iodo-2-methylbenzene (80 mg, 0.30 mmol, 1.5 eq.) as iodoaryl derivatives, Copper(I) thiophene-2-carboxylate (2.5 mg, 0.013 mmol,

0.1 eq.), *L*-Proline (5.8 mg, 0.05 mmol, 0.2 eq.) and potassium carbonate (160 mg, 0.50 mmol, 2.0 eq.) in dry DMSO (0.25 M). Crude product was purified by flash chromatography on silica gel using a gradient (PE: EtOAc) from (1: 0) to (7: 3) yielding to corresponding product **12** (50 mg, 70 %) as a colourless oil. Rf: 0.50 (PE/EtOAc: 70/30). IR (ATR diamond, cm^{-1}) ν: 3108, 2927, 1516, 1494, 1455, 1175, 1082, 1050, 798, 740, 696. ^{1}H NMR (400 MHz, Chloroform-*d*): δ 7.44 – 7.24 (m, 9H, H-9, H-10, H-11, H-14, H-15, H-16 and H-17), 6.97 (d, $J_{5\text{-}6}$ = 3.2 Hz, 1H, H-5), 5.74 (d, $J_{5\text{-}6}$ = 3.2 Hz, 1H, H-6), 5.64 (s, 2H, H-7), 2.30 (s, 3H, H-18). ^{13}C NMR (101 MHz, Chloroform-*d*): δ 150.8 (C_{3a}), 137.2 (C_{13}), 134.8 (C_q), 134.1 (C_q), 131.6 (CH_{Ar}), 131.4 (C_5), 129.0 (2 x CH_{Ar}), 128.6 (CH_{Ar}), 128.6 (2 x CH_{Ar}), 128.2 (C_q), 127.9 (CH_{Ar}), 126.9 (CH_{Ar}), 126.5 (CH_{Ar}), 89.5 (C_6), 53.8 (C_7), 18.6 (C_{18}). HRMS (EI^+) *m/z* calculated for $C_{18}H_{17}N_4$ $[M+H]^+$: 289.1448, found: 289.1452.

1-Benzyl-4-(4-nitrophenyl)-1,4-dihydropyrrolo[2,3-*d*][1,2,3]triazole (13)

The reaction was carried out as described in general procedure **B** using 1-benzyl-1,4-dihydropyrrolo[2,3-*d*][1,2,3]triazole **7** (50 mg, 0.25 mmol, 1.0 eq.), 1-iodo-4-nitrobenzene (70 mg, 0.30 mmol, 1.5 eq.) as iodoaryl derivative, Copper(I) thiophene-2-carboxylate (2.5 mg, 0.013 mmol, 0.1 eq.), *L*-Proline (5.8 mg, 0.05 mmol, 0.2 eq.) and potassium carbonate (160 mg, 0.50 mmol, 2.0 eq.) in dry DMSO (0.25 M). Crude product was purified by flash chromatography on silica gel using a gradient (PE: EtOAc) from (1: 0) to (7: 3) yielding to corresponding product **13** (62 mg, 78 %) as a white solid. Rf: 0.72 (PE/EtOAc: 50/50). MP: 189–191 °C. IR (ATR diamond, cm^{-1}) ν: 2822, 1522, 1346, 1111, 1084, 842, 736, 632. ^{1}H NMR (400 MHz, Chloroform-*d*): δ 8.35 (d, $J_{13\text{-}14}$ = 9.2 Hz, 2H, H-14), 8.05 (d, $J_{13\text{-}14}$ = 9.2 Hz, 2H, H-13), 7.45 – 7.34 (m, 6H, H-5, H-9, H-10 and H-11), 5.88 (d, $J_{5\text{-}6}$ = 3.5 Hz, 1H, H-6), 5.67 (s, 2H, H-7). ^{13}C NMR (101 MHz, Chloroform-*d*): δ 149.6 (C_{3a}), 144.2 (C_{12}), 143.1 (2 x C_{14}), 134.2 (C_8), 129.9 (C_{6a}), 129.2 (2 x CH_{Ar}), 129.0 (C_{11}), 128.6 (2 x CH_{Ar}), 126.6 (C_5), 125.9 (C_{14}), 117.7 (2 x C_{13}), 94.1 (C_6), 54.0 (C_7). HRMS (EI^+) *m/z* calculated for $C_{17}H_{14}N_5O_2$ $[M+H]^+$: 320.1142, found: 320.1140.

1-Benzyl-4-(4-trifluoromethyl)phenyl-1,4-dihydropyrrolo[2,3-*d*][1,2,3]triazole (14)

The reaction was carried out as described in general procedure **B** using 1-benzyl-1,4-dihydropyrrolo[2,3-*d*][1,2,3]triazole **7** (50 mg, 0.25 mmol, 1.0 eq.), 1-iodo-4-(trifluoromethyl)benzene (80 mg, 0.30 mmol, 1.50 eq.) as iodoaryl derivative, Copper(I) thiophene-2-carboxylate (2.50 mg, 0.013 mmol, 0.1 eq.), *L*-Proline (5.80 mg, 0.05 mmol, 0.2 eq.) and potassium carbonate (160 mg, 0.50 mmol, 2.0 eq.) in dry DMSO (0.25 M). Crude product was purified by flash chromatography on silica gel using a gradient (PE: EtOAc) from (1: 0) to (7: 3) yielding to corresponding product **14** (67 mg, 78 %) as a white solid. Rf: 0.75 (PE/EtOAc: 70/30). MP: 159–161 °C. IR (ATR diamond, cm^{-1}) ν: 3106, 2828, 1524, 1344, 1178, 1074, 1058, 968, 832, 741. ^{1}H NMR (400 MHz, Chloroform-*d*): δ 7.99 (d, $J_{13\text{-}14}$ = 8.4 Hz, 2H, H-13), 7.72 (d, $J_{13\text{-}14}$ = 8.4 Hz, 2H, H-14), 7.46 – 7.33 (m, 6H, H-5, H-9, H-10 and H-11), 5.83 (d, $J_{5\text{-}6}$ = 3.5 Hz, 1H, H-6), 5.66 (s, 2H, H-7). ^{13}C NMR (101 MHz, Chloroform-*d*): δ 149.5 (C_{3a}), 140.8 (C_{12}), 134.4 (C_q), 129.4 (C_q), 129.0 (2 x CH_{Ar}), 128.7 (C_{11}), 128.4 (2 x CH_{Ar}), 127.0 (q, *J* = 3.8 Hz, 2 x C_{14}), 126.8 (C_q), 126.5 (C_5), 124.0 (d, *J* = 271.5 Hz, C_{16}), 117.9 (2 x C_{13}), 92.5 (C_6), 53.8 (C_7). ^{19}F NMR (376 MHz, Chloroform-*d*): δ -62.1 (F-16). HRMS (EI^+) *m/z* calculated for $C_{18}H_{14}F_3N_4$ $[M+H]^{+:}$ 343.1165, found: 343.1169.

4-(1-Benzylpyrrolo[2,3-*d*][1,2,3]triazol-4(1*H*)-yl)phenol (15)

The reaction was carried out as described in general procedure **B** using 1-benzyl-1,4-dihydropyrrolo[2,3-*d*][1,2,3]triazole **7** (50 mg, 0.25 mmol, 1.0 eq.), 4-iodo-phenol (70 mg, 0.30 mmol, 1.5 eq.) as iodoaryl derivatives, Copper(I) thiophene-2-carboxylate (2.5 mg, 0.013 mmol, 0.1 eq.), *L*-Proline (5.8 mg, 0.05 mmol, 0.2 eq.) and potassium carbonate (160 mg, 0.50 mmol, 2.0 eq.) in dry DMSO (0.25 M). Crude product was purified by flash chromatography on silica gel using a gradient of (PE: EtOAc) from (1: 0) to (7: 3) yielding to corresponding product **15** (20 mg, 28 %) as a colourless oil.

Or under inert gas, 1-benzyl-4-(4-((tetrahydro-2H-pyraN2-yl)oxy)phenyl)-1.4-dihydropyrrolo [2,3-*d*][1,2,3]triazole **16** (40 mg, 0.10 mmol, 1.0 eq.) was dissolved in 3 mL of a mix (DCM 1/1 1,4-dioxane) and HCl 4M in 1,4-dioxane (0.33 mL, 1.30 mmol, 13.0 eq.) was added dropwise. The reaction mixture was stirred at room temperature during 18 h. After full consumption of reactants, the mixture was reduced under *vaccuo*. Residue was taken up in water before to be quenched with a saturated solution of Na_2CO_3 until pH = 9 and back extracted with DCM. Resultant organic layer was washed with a brine solution, dried over $MgSO_4$ and solvents were removed under reduced pressure. Crude product was directly purified by flash chromatography on silica gel using a gradient of (PE: EtOAc) from (1: 0) to (7: 3) yielding to the corresponding product **15** (27 mg, 0.09 mmol, 93%) as a colourless oil. Rf: 0.61 (PE/EtOAc: 30/70). IR (ATR diamond, cm^{-1}) ν: 3197, 2900, 2680, 1455, 1397, 1221, 1174, 1089, 775, 759, 632. ^1H NMR (400 MHz, Chloroform-*d*): δ 7.50 (d, $J_{13\text{-}14}$ = 8.4 Hz, 2H, H-13), 7.36 (m, 5H, H-9, H-10 and H-11), 7.19 (d, $J_{5\text{-}6}$ = 3.3 Hz, 1H, H-5), 6.91 (d, $J_{13\text{-}14}$ = 8.4 Hz, 2H, H-14), 5.73 (d, $J_{5\text{-}6}$ = 3.3 Hz, 1H, H-6), 5.64 (s, 2H, H-7). ^{13}C NMR (101 MHz, Chloroform-*d*): δ 154.5 (C_{15}), 149.2 (C_{3a}), 134.5 (C_8), 131.0 (C_{12}), 129.0 (2 x CH_{Ar}), 128.9 (C_{6a}), 128.6 (C_{11}), 128.5 (C_5), 128.4 (2 x CH_{Ar}), 121.3 (2 x C_{13}), 116.7 (2 x C_{14}), 89.8 (C_6), 53.8 (C_7). HRMS (EI^+) *m/z* calculated for $C_{17}H_{15}N_4O$ $[M+H]^+$: 291.1240, found: 291.1246.

1-Benzyl-4-(4-((tetrahydro-2*H*-pyraN2-yl)oxy)phenyl)-1.4-dihydropyrrolo[2,3-*d*][1,2,3]triazole (16)

The reaction was carried out as described in general procedure **B** using 1-benzyl-1,4-dihydropyrrolo[2,3-*d*][1,2,3]triazole **7** (50 mg, 0.25 mmol, 1.0 eq.), 2-(4-iodophenoxy)tetrahydro-2*H*-pyran (90 mg, 0.30 mmol, 1.5 eq.) as iodoaryl derivative, Copper(I) thiophene-2-carboxylate (2.5 mg, 0.013 mmol, 0.1 eq.), *L*-Proline (5.8 mg, 0.05 mmol, 0.2 eq.) and potassium carbonate (160 mg, 0.50 mmol, 2.0 eq.) in dry DMSO (0.25 M). Crude product was purified by flash chromatography on silica gel using a gradient (PE: EtOAc) from (1: 0) to (7: 3) yielding to corresponding product **16** (70 mg, 75 %) as a white solid. Rf: 0.35 (PE/EtOAc: 70/30). MP: 155–157 °C. IR (ATR diamond, cm^{-1}) ν: 3111, 2822, 1522, 1486, 1235, 1206, 1122, 1084, 888, 739, 720. ^1H NMR (400 MHz, Chloroform-*d*): δ 7.72 (d, $J_{13\text{-}14}$ = 9.0 Hz, 2H, H-13), 7.42 – 7.32 (m, 5H, H-9, H-10 and H-11), 7.26 (d, $J_{5\text{-}6}$ = 3.1 Hz, 1H, H-5), 7.16 (d, $J_{13\text{-}14}$ = 9.0 Hz, 2H, H-14), 5.74 (d, $J_{5\text{-}6}$ = 3.1 Hz, 1H, H-6), 5.64 (s, 2H, H-7), 5.48 – 5.37 (m, 1H, H-17), 3.93 (m, 1H, H-19), 3.74 – 3.41 (m, 1H, H-19), 2.01 (m, 1H, H-21), 1.88 (m, 2H, H-22), 1.76 – 1.57 (m, 3H, H-20 and H-21). ^{13}C NMR (101 MHz, Chloroform-*d*): δ 154.6 (C_{15}), 149.6 (C_{3a}), 134.8 (C_8), 132.6 (C_{12}), 129.0 (2 x CH_{Ar}), 129.0 (C_{6a}), 128.6 (C_{11}), 128.5 (2 x CH_{Ar}), 127.5 (C_5), 120.0 (2 x C_{13}), 117.7 (2 x C_{14}), 96.9 (C_{17}), 90.2 (C_6), 62.2 (C_{19}), 53.7 (C_7), 30.5 (C_{22}), 25.3 (C_{20}), 18.9 (C_{21}). HRMS (EI^+) *m/z* calculated for $C_{22}H_{23}N_4O_2$ $[M+H]^+$: 375.1816, found: 375.1818.

1-Benzyl-4-(thiophen-3-yl)-1,4-dihydropyrrolo[2,3-*d*][1,2,3]triazole (17)

The reaction was carried out as described in general procedure **B** using 1-benzyl-1,4-dihydropyrrolo[2,3-*d*][1,2,3]triazole **7** (50 mg, 0.25 mmol, 1.0 eq.), 3-iodothiophene (60 mg, 0.30 mmol, 1.5 eq.) as iodoaryl derivative, Copper(I) thiophene-2-carboxylate (2.5 mg, 0.013 mmol, 0.1 eq.), *L*-Proline (5.8 mg, 0.05 mmol, 0.2 eq.) and potassium carbonate (160 mg, 0.50 mmol, 2.0 eq.) in dry DMSO (0.25 M). Crude product was purified by flash chromatography on silica gel using a gradient (PE: EtOAc) from (1: 0) to (7: 3) yielding to corresponding product **17** (53 mg, 76 %) as a white solid. Rf: 0.42 (PE/EtOAc: 80/20). MP: 113–115 °C. IR (ATR diamond, cm^{-1}) ν: 3105, 2828, 1563, 1491, 1297, 1218, 1204, 1173, 1108, 1048, 1038, 819. ^1H NMR (400 MHz, Chloroform-*d*): δ 7.51 (m, 2H, H-13 and H-15), 7.42 – 7.31 (m, 6H, H-9, H-10, H-11 and H-16), 7.23 (d, $J_{5\text{-}6}$ = 3.4 Hz, 1H, H-5), 5.73 (d, $J_{5\text{-}6}$ = 3.4 Hz, 1H, H-6), 5.64 (s, 2H, H-7). ^{13}C NMR (101 MHz, Chloroform-*d*): δ 149.5 (C_{3a}), 137.1 (C_{12}), 134.8 (C_8), 129.1 (2 x CH_{Ar}), 128.7 (C_{11}), 128.6 (C_{6a}), 128.5 (2 x CH_{Ar}), 127.9 (C_5), 126.6 (C_{16}), 119.8 (CH_{Ar}), 108.9 (CH_{Ar}), 90.5 (C_6), 53.8 (C_7). HRMS (EI^+) *m/z* calculated for $C_{15}H_{13}N_4S$ $[M+H]^+$: 281.0855, found: 281.0857.

1-Benzyl-4-(thiophen-2-yl)-1,4-dihydropyrrolo[2,3-*d*][1,2,3]triazole (18)

The reaction was carried out as described in general procedure **B** using 1-benzyl-1,4-dihydropyrrolo[2,3-*d*][1,2,3]triazole **7** (50 mg, 0.25 mmol, 1.0 eq.), 2-iodothiophene (60 mg, 0.30 mmol, 1.5 eq.) as iodoaryl derivative, Copper(I) thiophene-2-carboxylate (2.5 mg, 0.013 mmol, 0.1 eq.), *L*-Proline (5.8 mg, 0.05 mmol, 0.2 eq.) and potassium carbonate (160 mg, 0.50 mmol, 2.0 eq.) in dry DMSO (0.25 M). Crude product was purified by flash chromatography on silica gel using a gradient (PE: EtOAc) from (1: 0) to (7: 3) yielding to corresponding product **18** (37 mg, 53 %) as a white solid. Rf: 0.68 (PE/EtOAc: 70/30). MP: 104–106 °C. IR (ATR diamond, cm^{-1}) ν: 3105, 2831, 1547, 1508, 1224, 968, 850, 740, 639. ^{1}H NMR (400 MHz, Chloroform-*d*): δ 7.43 – 7.29 (m, 6H, H-9, H-10, H-11 and H-14), 7.12 (d, J_{5-6} = 3.4 Hz, 1H, H-5), 7.01 – 6.95 (m, 2H, H-15 and H-16), 5.73 (d, J_{5-6} = 3.4 Hz, 1H, H-6), 5.64 (s, 2H, H-7). ^{13}C NMR (101 MHz, Chloroform-*d*): δ 149.8 (C_{3a}), 140.6 (C_{12}), 134.6 (C_8), 129.4 (C_5), 129.1 (2 x CH_{Ar}), 128.8 (C_{11}), 128.6 (C_{6a}), 128.5 (2 x CH_{Ar}), 126.6 (CH_{Ar}), 118.4 (CH_{Ar}), 115.6 (CH_{Ar}), 91.2 (C_6), 53.9 (C_7). HRMS (EI^+) *m/z* calculated for $C_{15}H_{13}N_4S$ $[M+H]^+$: 281.0855, found: 281.0854.

1-Benzyl-4-methyl-1,4-dihydropyrrolo[2,3-*d*][1,2,3]triazole (19)

Under an inert gas, 1-benzyl-1,4-dihydropyrrolo[2,3-*d*][1,2,3]triazole **7** (50 mg, 0.25 mmol, 1.0 eq.) was dissolved in dry THF (0.025 M). The reactional mixture was cooled to 0 °C and sodium hydride (9.1 mg, 0.38 mmol, 1.5 eq.) was added portion wise and, then, stirred 15 min at 0 °C. Iodomethane (56 mg, 0.328 mmol, 1.3 eq.) was added and the mixture was finally stirred 1h at room temperature. After full consumption of reactants, reaction was cooled, water was added and the aqueous layer was extracted with DCM. The resultant organic layer was washed with brine and solvents were removed under reduced pressure. Crude product was purified by flash chromatography on silica gel using a gradient of (PE: EtOAc) from (1: 0) to (1: 1) yielding to corresponding product **19** (49 mg, 93 %) as a yellow oil. Rf: 0.37 (PE/EtOAc: 50/50). MP: 81–83 °C. IR (ATR diamond, cm^{-1}) ν: 2924, 1540, 1357, 1329, 1169, 1096, 1066, 934, 779, 738, 700. ^{1}H NMR (400 MHz, Chloroform-*d*): δ 7.40 – 7.31 (m, 5H, H-9, H-10 and H-11), 6.79 (d, J_{5-6} = 3.1 Hz, 1H, H-5), 5.61 (s, 2H, H-7), 5.58 (d, J_{5-6} = 3.1 Hz, 1H, H-6), 3.80 (s, 3H, H-12). ^{13}C NMR (101 MHz, Chloroform-*d*): δ 151.2 (C_{3a}), 135.1 (C_8), 131.5 (C_5), 129.0 (2 x CH_{Ar}), 128.5 (C_{11}), 128.4 (2 x CH_{Ar}), 127.9 (C_{6a}), 87.7 (C_6), 53.7 (C_7), 33.2 (C_{12}). HRMS (EI^+) *m/z* calculated for $C_{12}H_{13}N_4$ $[M+H]^+$: 213.1135, found: 213.1138.

1-Benzyl-4-pentyl-1,4-dihydropyrrolo[2,3-*d*][1,2,3]triazole (20)

Under inert gas, 1-benzyl-1,4-dihydropyrrolo[2,3-*d*][1,2,3]triazole **7** (50 mg, 0.25 mmol, 1.0 eq.) was dissolved in dry THF (0.025 M). Reaction mixture was cooled to 0 °C and sodium hydride (9.1 mg, 0.38 mmol, 1.5 eq.) was added portion wise and, then, stirred 15 min at 0 °C. Iodopentane (70 mg, 0.33 mmol, 1.3 eq.) was added and the mixture was finally stirred 19 hours at reflux. After full consumption of reactants, reaction was cooled, water was added and the aqueous layer was extracted with DCM. The resultant organic layer was washed with brine and solvents were removed under reduced pressure. Crude product was purified by flash chromatography on silica gel using a gradient of (PE: EtOAc) from (1: 0) to (1: 1) yielding to corresponding product **20** (40 mg, 60 %) as a yellow oil. Rf: 0.41 (PE/EtOAc: 80/20). IR (ATR diamond, cm^{-1}) ν: 2929, 2860, 2364, 1521, 1490, 1179, 1084, 731, 696. ^{1}H NMR (400 MHz, Chloroform-*d*): δ 7.38 – 7.28 (m, 5H, H-9, H-10 and H-11), 6.79 (d, J_{5-6} = 3.1 Hz, 1H, H-5), 5.57 (s, 2H, H-7), 5.53 (d, J_{5-6} = 3.1 Hz, 1H, H-6), 4.03 (t, J_{12-13} = 7.2 Hz, 2H, H-12), 1.91 (p, $J_{12-13-14}$ = 7.3 Hz, 2H, H-13), 1.37 – 1.24 (m, 4H, H-14 and H-15), 0.87 (t, J_{15-16} = 6.9 Hz, 3H, H-16). ^{13}C NMR (101 MHz, Chloroform-*d*): δ 150.6 (C_{3a}), 135.0 (C_8), 130.5 (C_5), 128.9 (2 x CH_{Ar}), 128.4 (C_{11}), 128.4 (2 x CH_{Ar}), 127.9 (C_{6a}), 87.3 (C_6), 53.6 (C_7), 47.25 (C_{12}) 30.0 (C_{13}), 28.9 (C_{14}), 22.3 (C_{15}), 14.0 (C_{16}). HRMS (EI^+) *m/z* calculated for $C_{16}H_{21}N_4$ $[M+H]^+$: 269.1761, found: 269.1763.

1-Benzyl-4-isopropyl-1,4-dihydropyrrolo[2,3-*d*][1,2,3]triazole (21)

Under inert gas, 1-benzyl-1,4-dihydropyrrolo[2,3-*d*][1,2,3]triazole **7** (50 mg, 0.25 mmol, 1.0 eq.) was dissolved in dry THF (0.025 M). Reactional mixture was cooled to 0 °C and sodium hydride (9.1 mg, 0.38 mmol, 1.5 eq.) was added portion wise and then, stirred 15 min at 0 °C. 2-iodopropane (56 mg, 0.328 mmol, 1.3 eq.) was added and the mixture was finally stirred 19 hours at reflux. After full consumption of reactants, reaction was cooled, water was added and the aqueous layer was extracted with DCM. The resultant organic layer was washed with brine and solvents were removed under reduced pressure. Crude product was purified by flash chromatography on silica gel using a gradient of (PE: EtOAc) from (1: 0) to (1: 1) yielding to corresponding product **21** (18 mg, 30 %) as a yellow oil. Rf: 0.52 (PE/EtOAc: 50/50). IR (ATR diamond, cm^{-1}) ν: 2975, 1512, 1487, 1369, 1271, 1206, 1106, 1083, 1056, 1029. ^1H NMR (400 MHz, Chloroform-*d*): δ 7.38 – 7.29 (m, 5H, H-9, H-10 and H-11), 6.87 (d, J_{5-6} = 2.5 Hz, 1H, H-5), 5.58 (s, 2H, H-7), 5.53 (d, J_{5-6} = 2.7 Hz, 1H, H-6), 4.49 (p, J_{12-13} = 7.0 Hz, 1H, H-12), 1.58 (d, J_{12-13} = 7.0 Hz, 6H, H-13). ^{13}C NMR (101 MHz, Chloroform-*d*): δ 149.9 (C_{3a}), 135.1 (C_8), 128.9 (2 x CH_{Ar}), 128.5 (2 x CH_{Ar}), 128.5 (C_{11}), 128.2 (C_{6a}), 128.1 (C_5), 87.1 (C_6), 53.6 (C_7), 49.9 (C_{12}), 22.9 (C_{13}). HRMS (EI^+) *m/z* calculated for $C_{14}H_{17}N_4$ $[M+H]^+$: 241.1448, found: 241.1449.

3-(4-benzylpyrrolo[2,3-*d*][1,2,3]triazol-1(4*H*)-yl)propanenitrile (23)

1-Benzylpyrrolidin-3-one (260 mg, 1.50 mmol, 1.0 eq.), 3-aminopropanenitrile (320 mg, 4.50 mmol, 3.0 eq.), 1-Azido-4-nitrobenzene (1.23 g, 7.50 mmol, 5.0 eq.), acetic acid (0.03 mL, 0.45 mmol, 0.3 eq.) and molecular sieve (3 Å) were added at anhydrous toluene (0.25 M) and reactional mixture was stirred 1h at 140°C under microwave irradiation. The resulting mixture was reduced under vacuum and filtered through a pad of charcoal. The crude mixture was purified by flash chromatography on silica gel using first DCM and then (PE/EtOAc: 60/40) yielding to corresponding product **23** (250 mg, 67 %) as a white solid. Rf: 0.14 (EP/EtOAc: 70/30). MP: 72–74 °C. IR (ATR diamond, cm^{-1}) ν: 2940, 2254, 1534, 1490, 1366, 1184, 1088, 1063, 940. ^1H NMR (400 MHz, Chloroform-*d*): δ 7.38 – 7.27 (m, 5H, H-13, H-14, H-15), 6.96 (d, J_{5-6} = 3.2 Hz, 1H, H-5), 6.06 (d, J_{5-6} = 3.2 Hz, 1H, H-6), 5.27 (s, 2H, H-11), 4.74 (t, J_{7-8} = 6.8 Hz, 2H, H-7), 3.02 (t, J_{7-8} = 6.8 Hz, 2H, H-8). ^{13}C NMR (101 MHz, Chloroform-*d*): δ 150.8 (C_{3a}), 136.7 (C_{12}), 131.2 (C_5), 129.0 (2 x CH_{Ar}), 128.2 (C_{15}), 128.1 (C_{6a}), 127.8 (2 x CH_{Ar}), 117.0 (C_9), 88.2 (C_6), 50.8 (C_{11}), 45.2 (C_7), 19.0 (C_8). HRMS (EI^+) *m/z* calculated for $C_{14}H_{14}N_5$ $[M+H]^+$: 252.1244, found: 252.1247.

4-benzyl-1,4-dihydropyrrolo[2,3-*d*][1,2,3]triazole (24)

The reaction was carried out as described in general procedure **A** using 3-(4-benzylpyrrolo[2,3-*d*][1,2,3]triazol-1(4*H*)-yl)propanenitrile **23** (620 mg, 2.47 mmol, 1.0 eq.) and a 1.0 M solution of potassium *tert*-butoxide in EtOH (16.80 mL, 16.80 mmol, 6.8 eq). Crude product was purified by flash chromatography on silica gel using a gradient of (PE/EtOAc) from (1: 0) to (1: 1) yielding to corresponding product **24** (390 mg, 79 %) as a white solid. Rf: 0.25 (EP/EtOAc: 80/20). MP: 137–139 °C. IR (ATR diamond, cm^{-1}) ν: 3061, 2916, 2789, 1544, 1453, 1238, 1144, 1053, 1039, 762, 739, 704. ^1H NMR (400 MHz, Chloroform-*d*): δ 12.35 (br, 1H, N_1-H), 7.35 – 7.23 (m, 5H, H-9, H-10 and H-11), 7.04 (d, J_{5-6} = 3.3 Hz, 1H, H-5), 6.15 (d, J_{5-6} = 3.3 Hz, 1H, H-6), 5.24 (s, 2H, H-7). ^{13}C NMR (101 MHz, Chloroform-*d*): δ 149.6 (C_{3a}), 136.9 (C_8 and C_{6a}), 133.5 (C_5), 128.9 (2 x CH_{Ar}), 128.1 (C_{11}), 127.7 (2 x CH_{Ar}), 91.0 (C_6), 51.0 (CH_7). HRMS (EI^+) *m/z* calculated for $C_{11}H_{11}N_4$ $[M+H]^+$: 199.0978, found: 199.0977.

4-Benzyl-1-(*p*-tolyl)-1,4-dihydropyrrolo[2,3-*d*][1,2,3]triazole (25a)

The reaction was carried out as described in general procedure **C** using 4-benzyl-1,4-dihydropyrrolo[2,3-*d*][1,2,3]triazole **24** (40 mg, 0.20 mmol, 1.0 eq.) as triazole derivative, 4-iodotoluene (70 mg, 0.30 mmol, 1.5 eq.) as iodoaryl derivative, potassium carbonate (60 mg, 0.40 mmol, 2.0 eq.), Copper(I) thiophene-2-carboxylate (3.81 mg, 0.02 mmol, 0.1 eq.) and *L*-Proline (4.61 mg, 0.04, 0.2 eq.) in dry DMSO (0.25 M). Crude product was purified by flash chromatography on silica

gel column using a gradient (PE: EtOAc) from (1: 0) to (8: 2) yielding to corresponding product **25a** (24 mg, 42 %) as a white solid.

Or the reaction was carried out as described in general procedure **D** using 4-benzyl-1,4-dihydropyrrolo[2,3-*d*][1,2,3]triazole **24** (50 mg, 0.25 mmol, 1.0 eq.) as triazole derivative, 4-methylphenyl(2,4,6-trimethoxyphenyl)iodonium trifluoroacetate (252 mg, 0.51 mmol, 2.0 eq.) as aryliodonium salt and potassium carbonate (100 mg, 0.30 mmol, 1.2 eq.) in dry toluene (0.15 M). Crude product was purified by flash chromatography on C18-reversed silica gel column using a gradient (H_2O: ACN) from (5: 5) to (4: 6) yielding to corresponding product **25a** (8 mg, 14 %) as a white solid. Rf: 0.12 (EP/EtOAc: 90/10). MP: 161–163 °C. IR (ATR diamond, cm^{-1}) ν: 3243, 3105, 1771, 1661, 1523, 1477, 1295, 1177, 1058, 837, 767, 686. ^1H NMR (400 MHz, Chloroform-*d*): δ 7.76 (d, J_{8-9} = 8.4 Hz, 2H, H-8), 7.38 – 7.28 (m, 7H, H-9, H-14, H-15 and H-16), 7.00 (d, J_{5-6} = 3.1 Hz, 1H, H-5), 6.22 (d, J_{5-6} = 3.1 Hz, 1H, H-6), 5.33 (s, 2H, H-12), 2.41 (s, 3H, H-11). ^{13}C NMR (101 MHz, Chloroform-*d*): δ 150.8 (C_{3a}), 136.9 (C_q), 136.9 (C_q), 136.1 (C_{13}), 130.8 (C_5), 130.2 (2 x CH_{Ar}), 129.0 (2 x CH_{Ar}), 128.2 (CH_{Ar}), 127.9 (2 x CH_{Ar}), 126.2 (C_{6a}), 118.8 (2 x C_8), 89.8 (C_6), 50.8 (C_{12}), 21.2 (C_{11}). HRMS (EI^+) *m/z* calculated for $C_{18}H_{17}N_4$ $[M+H]^+$: 289.1448, found: 289.1443.

4-Benzyl-2-(*p*-tolyl)-2,4-dihydropyrrolo[2,3-*d*][1,2,3]triazole (25b)

The reaction was carried out as described in general procedure **C** using 4-benzyl-1,4-dihydropyrrolo[2,3-*d*][1,2,3]triazole **24** (40 mg, 0.20 mmol, 1.0 eq.) as triazole derivative, 4-iodotoluene (70 mg, 0.30 mmol, 1.5 eq.) as iodoaryl derivative, potassium carbonate (60 mg, 0.40 mmol, 2.0 eq.), Copper(I) thiophene-2-carboxylate (3.81 mg, 0.02 mmol, 0.1 eq.) and *L*-Proline (4.61 mg, 0.04 mmol, 0.2 eq.) in dry DMSO (0.25 M). Crude product was purified by flash chromatography in silica gel column using a gradient (PE: EtOAc) from (1: 0) to (8: 2) yielding to corresponding product **25b** (30 mg, 52 %) as a white solid.

Or the reaction was carried out as described in general procedure **D** using 4-benzyl-1,4-dihydropyrrolo[2,3-*d*][1,2,3]triazole **24** (40 mg, 0.20 mmol, 1.0 eq.) as triazole derivative, 4-methylphenyl(2,4,6-trimethoxyphenyl)iodonium trifluoroacetate (201 mg, 0.40 mmol, 2.0 eq.) as aryliodonium salt and potassium carbonate (30 mg, 0.24 mmol, 1.2 eq.) in dry toluene (0.15 M). Crude product was purified by flash chromatography on C18-reversed silica gel column using a gradient (H_2O: ACN) from (5: 5) to (4: 6) yielding to corresponding product **25b** (39 mg, 67 %) as a white solid. Rf: 0.34 (EP/EtOAc: 90/10). MP: 133–135 °C. IR (ATR diamond, cm^{-1}) ν: 3243, 1661, 1523, 1295, 1263, 1177, 1058, 1023, 837, 767, 686. ^1H NMR (400 MHz, Chloroform-*d*): δ 7.97 (d, J_{8-9} = 8.5 Hz, 2H, H-8), 7.40 – 7.18 (m, 7H, H-9, H-14, H-15 and H-16), 7.02 (d, J_{5-6} = 3.4 Hz, 1H, H-5), 6.23 (d, J_{5-6} = 3.4 Hz, 1H, H-6), 5.21 (s, 2H, H-12), 2.38 (s, 3H, H-11). ^{13}C NMR (101 MHz, Chloroform-*d*): δ 150.4 (C_{3a}), 142.8 (C_{6a}), 139.4 (C_7), 136.9 (C_{10}), 136.4 (C_{13}), 134.9 (C_5), 129.8 (2 x CH_{Ar}), 128.9 (2 x CH_{Ar}), 128.1 (C_{16}), 127.6 (2 x CH_{Ar}), 118.9 (2 x C_8), 93.0 (C_5), 51.0 (C_{12}), 21.1 (C_{11}). HRMS (EI^+) *m/z* calculated for $C_{18}H_{17}N_4$ $[M+H]^+$: 289.1448, found: 289.1446.

4-Benzyl-1-phenyl-1,4-dihydropyrrolo[2,3-*d*][1,2,3]triazole (26a)

The reaction was carried out as described in general procedure **C** using 4-benzyl-1,4-dihydropyrrolo[2,3-*d*][1,2,3]triazole **24** (40 mg, 0.20 mmol, 1.0 eq.) as triazole derivative, iodobenzene (62 mg, 0.30 mmol, 1.5 eq.) as iodoaryl derivative, potassium carbonate (56 mg, 0.40 mmol, 2.0 eq.), Copper(I) thiophene-2-carboxylate (3.81 mg, 0.02 mmol, 0.1 eq.) and *L*-Proline (4.61 mg, 0.04 mmol, 0.2 eq.) in dry DMSO (0.25 M). Crude product was purified by flash chromatography in silica gel column using a gradient (PE: EtOAc) from (1: 0) to (8: 2) yielding to corresponding product **26a** (27 mg, 50 %) as a white solid.

Or the reaction was carried out as described in general procedure **D** using 4-benzyl-1,4-dihydropyrrolo[2,3-*d*][1,2,3]triazole **24** (40 mg, 0.20 mmol, 1.0 eq.) as triazole derivative, phenyl(2,4,6-trimethoxyphenyl)iodonium trifluoroacetate (196 mg, 0.40 mmol, 2.0 eq.) as aryliodonium salt and potassium carbonate (30 mg, 0.24 mmol, 1.2 eq.) in dry toluene (0.15 M). Crude product was purified by flash chromatography on C18-reversed silica gel column using a gradient (H_2O: ACN) from (5: 5) to (4: 6) yielding to corresponding product **26a** (9 mg, 16 %) as a white solid. Rf: 0.21 (H_2O/ACN: 40/60). MP: 75–77 °C. IR (ATR diamond, cm^{-1}) ν: 3140, 2919, 2840, 1613, 1519, 1376, 1247, 1158, 1100, 897, 695, 664. ^1H NMR (400 MHz, Chloroform-*d*): δ 7.89 (dd, J_{8-9} = 8.6, J_{8-10} = 1.2 Hz, 2H, H-8), 7.52 (dd, J_{8-9} = 8.6, J_{9-10} = 7.5 Hz, 2H, H-9), 7.40 – 7.28 (m, 6H, H-10, H-13, H-14 and H-15), 7.02 (d, J_{5-6} = 3.1 Hz, 1H, H-5), 6.24 (d, J_{5-6} = 3.2 Hz, 1H, H-6), 5.34 (s, 2H, H-11). ^{13}C NMR (101 MHz, Chloroform-*d*): δ 150.8 (C_{3a}), 138.4 (C_7), 136.8 (C_{12}), 130.9 (C_5), 129.7 (2 x C_9), 129.0 (2 x CH_{Ar}), 128.2 (C_{15}), 127.9 (2 x CH_{Ar}), 127.0 (C_{10}), 126.2 (C_{6a}), 118.8 (2 x C_8), 89.9 (C_6), 50.8 (C_{11}). HRMS (EI^+) *m/z* calculated for $C_{17}H_{15}N_4$ $[M+H]^+$: 275.1291, found: 275.1296.

4-Benzyl-2-phenyl-2,4-dihydropyrrolo[2,3-*d*][1,2,3]triazole (26b)

The reaction was carried out as described in general procedure **C** using 4-benzyl-1,4-dihydropyrrolo[2,3-*d*][1,2,3]triazole **24** (40 mg, 0.20 mmol, 1.0 eq.) as triazole derivative, iodobenzene (62 mg, 0.30 mmol, 1.5 eq.) as iodoaryl derivative, potassium carbonate (56 mg, 0.40 mmol, 2.0 eq.), Copper(I) thiophene-2-carboxylate (3.81 mg, 0.02 mmol, 0.1 eq.) and *L*-Proline (4.61 mg, 0.04 mmol, 0.2 eq.) in dry DMSO (0.25 M). Crude product was purified by flash chromatography in silica gel column using a gradient (PE: EtOAc) from (1: 0) to (8: 2) yielding to corresponding product **26b** (27 mg, 50 %) as a white solid.

Or the reaction was carried out as described in general procedure **D** using 4-benzyl-1,4-dihydropyrrolo[2,3-*d*][1,2,3]triazole **24** (40 mg, 0.20 mmol, 1.0 eq.) as triazole derivative, phenyl(2,4,6-trimethoxyphenyl)iodonium trifluoroacetate (196 mg, 0.40 mmol, 2.0 eq.) as aryliodonium salt and potassium carbonate (30 mg, 0.24 mmol, 1.2 eq.) in dry toluene (0.15 M). Crude product was purified by flash chromatography on C18-reversed silica gel column using a gradient (H_2O: ACN) from (5: 5) to (4: 6) yielding to corresponding product **26b** (36 mg, 65 %) as a white solid. Rf: 0.32 (H_2O/ACN: 40/60). Mp: 82–84 °C. IR (ATR diamond, cm^{-1}) ν: 2930, 1595, 1518, 1342, 150, 1068, 935, 752, 728, 697. ^1H NMR (400 MHz, Chloroform-*d*): δ 8.11 (dd, J_{8-9} = 8.6, J_{8-10} = 1.2 Hz, 2H, H-8), 7.46 (dd, J_{8-9} = 8.6, J_{9-10} = 7.3 Hz, 2H, H-9), 7.39 – 7.18 (m, 6H, H-10, H-13, H-14 and H-15), 7.05 (d, J_{5-6} = 3.4 Hz, 1H, H-5), 6.25 (d, J_{5-6} = 3.4 Hz, 1H, H-6), 5.23 (s, 2H, H-11). ^{13}C NMR (101 MHz, Chloroform-*d*): δ 150.6 (C_{3a}), 143.1 (C_{6a}), 141.5 (C_7), 136.9 (C_{12}), 135.3 (C_5), 129.3 (2 x CH_{Ar}), 129.0 (2 x CH_{Ar}), 128.1 (CH_{Ar}), 127.6 (2 x CH_{Ar}), 126.6 (CH_{Ar}), 118.9 (2 x C_8), 93.0 (C_6), 51.0 (C_{11}). HRMS (EI^+) *m/z* calculated for $C_{17}H_{15}N_4$ $[M+H]^+$: 275.1291, found: 275.1294.

4-Benzyl-1-(4-methoxyphenyl)-1,4-dihydropyrrolo[2,3-*d*][1,2,3]triazole (27a)

The reaction was carried out as described in general procedure **C** using 4-benzyl-1,4-dihydropyrrolo[2,3-*d*][1,2,3]triazole **24** (40 mg, 0.20 mmol, 1.0 eq.) as triazole derivative, 4-iodoanisole (71 mg, 0.30 mmol, 1.5 eq.) as iodoaryl derivative, potassium carbonate (56 mg, 0.40 mmol, 2.0 eq.), Copper(I) thiophene-2-carboxylate (3.81 mg, 0.02 mmol, 0.1 eq.) and *L*-Proline (4.61 mg, 0.04 mmol, 0.20 eq.) in dry DMSO (0.25 M). Crude product was purified by flash chromatography in silica gel column using a gradient (PE: EtOAc) from (1: 0) to (8: 2) yielding to corresponding product **27a** (31 mg, 50 %) as a white solid.

Or the reaction was carried out as described in general procedure **D** using 4-benzyl-1,4-dihydropyrrolo[2,3-*d*][1,2,3]triazole **24** (40 mg, 0.20 mmol, 1.0 eq.) as triazole derivative, 4-methoxyphenyl(2,4,6-trimethoxyphenyl)iodonium trifluoroacetate (208 mg, 0.40 mmol, 2.0 eq.) as

aryliodonium salt and potassium carbonate (30 mg, 0.24 mmol, 1.2 eq.) in dry toluene (0.15 M). Crude product was purified by flash chromatography on C18-reversed silica gel column using a gradient (H_2O: ACN) from (5: 5) to (4: 6) yielding to corresponding product **27a** (10 mg, 16 %) as a white solid. Rf: 0.11 (H_2O/ACN: 40/60). MP: 139–141 °C. IR (ATR diamond, cm^{-1}) ν: 3000, 2846, 1516, 1305, 1201, 1072, 825, 766, 750, 737, 648. ^1H NMR (400 MHz, Chloroform-*d*): δ 7.79 (d, J_{8-9} = 9.0 Hz, 2H, H-8), 7.38 – 7.28 (m, 5H, H-15, H-16 and H-17), 7.04 (d, J_{8-9} = 9.0 Hz, 2H, H-9), 7.00 (d, J_{5-6} = 3.2 Hz, 1H, H-5), 6.19 (d, J_{5-6} = 3.2 Hz, 1H, H-6), 5.33 (s, 2H, H-13), 3.87 (s, 3H, H-12). ^{13}C NMR (101 MHz, Chloroform-*d*): δ 158.6 (C_{10}), 150.8 (C_{3a}), 136.9 (C_{14}), 132.0 (C_7), 130.9 (C_5), 129.0 (2 x CH_{Ar}), 128.2 (C_{17}), 127.9 (2 x CH_{Ar}), 126.3 (C_{6a}), 120.4 (2 x C_8), 114.9 (2 x C_9), 89.5 (C_6), 55.7 (C_{12}), 50.8 (C_{13}). HRMS (EI^+) *m/z* calculated for $C_{18}H_{17}N_4O$ $[M+H]^+$: 305.1397, found: 305.1402

4-Benzyl-2-(4-methoxyphenyl)-2,4-dihydropyrrolo[2,3-*d*][1,2,3]triazole (27b)

The reaction was carried out as described in general procedure **C** using 4-benzyl-1,4-dihydropyrrolo[2,3-*d*][1,2,3]triazole **24** (40 mg, 0.20 mmol, 1.0 eq.) as triazole derivative, 4-iodoanisole (71 mg, 0.30 mmol, 1.5 eq.) as iodoaryl derivative, potassium carbonate (56 mg, 0.40 mmol, 2.0 eq.), Copper(I) thiophene-2-carboxylate (3.81 mg, 0.02 mmol, 0.1 eq.) and *L*-Proline (4.61 mg, 0.04 mmol, 0.20 eq.) in dry DMSO (0.25 M). Crude product was purified by flash chromatography in silica gel column using a gradient (PE: EtOAc) from (1: 0) to (8: 2) yielding to corresponding product **27b** (25 mg, 40 %) as a white solid.

Or the reaction was carried out as described in general procedure **D** using 4-benzyl-1,4-dihydropyrrolo[2,3-*d*][1,2,3]triazole **24** (40 mg, 0.20 mmol, 1.0 eq.) as triazole derivative, 4-methoxyphenyl(2,4,6-trimethoxyphenyl)iodonium trifluoroacetate (208 mg, 0.40 mmol, 2.0 eq.) as aryliodonium salt and potassium carbonate (30 mg, 0.24 mmol, 1.2 eq.) in dry toluene (0.15 M). Crude product was purified by flash chromatography on C18-reversed silica gel column using a gradient (H_2O: ACN) from (5: 5) to (4: 6) yielding to corresponding product **27b** (30 mg, 49 %) as a white solid. Rf: 0.26 (H_2O/ACN: 40/60). MP: 121–123 °C. IR (ATR diamond, cm^{-1}) ν: 3117, 2919, 2828, 1585, 1518, 1447, 1174, 1072, 1048, 959, 827, 751, 705. ^1H NMR (400 MHz, Chloroform-*d*): δ 8.00 (d, J_{8-9} = 9.1 Hz, 2H, H-8), 7.36 – 7.24 (m, 5H, H-15, H-16 and H-17), 7.02 (d, J_{5-6} = 3.4 Hz, 1H, H-5), 6.97 (d, J_{8-9} = 9.1 Hz, 2H, H-9), 6.23 (d, J_{5-6} = 3.4 Hz, 1H, H-6), 5.22 (s, 2H, H-13), 3.85 (s, 3H, H-12). ^{13}C NMR (101 MHz, Chloroform-*d*): δ 158.5 (C_{10}), 150.3 (C_{3a}), 142.6 (C_{6a}), 137.0 (C_{14}), 135.4 (C_7), 134.5 (C_5), 128.9 (2 x CH_{Ar}), 128.1 (C_{17}), 127.6 (2 x CH_{Ar}), 120.4 (2 x C_8), 114.4 (2 x C_9), 92.9 (C_6), 55.7 (C_{12}), 51.0 (C_{13}). HRMS (EI^+) *m/z* calculated for $C_{18}H_{17}N_4O$ $[M+H]^+$: 305.1397, found: 305.1401.

4-Benzyl-1-(*m*-tolyl)-1,4-dihydropyrrolo[2,3-*d*][1,2,3]triazole (28a)

The reaction was carried out as described in general procedure **C** using 4-benzyl-1,4-dihydropyrrolo[2,3-*d*][1,2,3]triazole **24** (35 mg, 0.18 mmol, 1.0 eq.) as triazole derivative, 3-iodotoluene (58 mg, 0.27 mmol, 1.5 eq.) as iodoaryl derivative, potassium carbonate (49 mg, 0.35 mmol, 2.0 eq.), Copper(I) thiophene-2-carboxylate (3.81 mg, 0.02 mmol, 0.1 eq.) and *L*-Proline (4.14 mg, 0.04 mmol, 0.2 eq.) in dry DMSO (0.25 M). Crude product was purified by flash chromatography in silica gel column using a gradient (PE: EtOAc) from (1: 0) to (8: 2) yielding to corresponding product **28a** (17 mg, 33 %) as a brown oil.

Or the reaction was carried out as described in general procedure **D** using 4-benzyl-1,4-dihydropyrrolo[2,3-*d*][1,2,3]triazole **24** (40 mg, 0.20 mmol, 1.0 eq.) as triazole derivative, 3-methylphenyl(2,4,6-trimethoxyphenyl)iodonium trifluoroacetate (252 mg, 0.40 mmol, 2.0 eq.) as aryliodonium salt and potassium carbonate (30 mg, 0.24 mmol, 1.2 eq.) in dry toluene (0.15 M). Crude product was purified by flash chromatography on C18-reversed silica gel column using a gradient (H_2O: ACN) from (5: 5) to (4: 6) yielding to corresponding product **28a** (7 mg, 12 %) as a brown oil. Rf: 0.09

(H_2O/ACN: 40/60). IR (ATR diamond, cm^{-1}) ν: 2916, 1700, 1610, 1521, 1244, 1204, 1097, 1035, 837, 698. ^1H NMR (400 MHz, Chloroform-*d*): δ 7.74 (s, 1H, H-8), 7.65 (d, J_{11-12} = 7.8 Hz, 1H, H-12), 7.40 (t, $J_{10-11-12}$ = 7.8 Hz, 1H, H-11), 7.36 – 7.28 (m, 5H, H-16, H-17 and H-18), 7.15 (d, J_{10-11} = 7.8 Hz, 1H, H-10), 7.01 (d, J_{5-6} = 3.2 Hz, 1H, H-5), 6.24 (d, J_{5-6} = 3.2 Hz, 1H, H-6), 5.34 (s, 2H, H-14), 2.46 (s, 3H, H-13). ^{13}C NMR (101 MHz, Chloroform-*d*): δ 150.8 (C_{3a}), 139.8 (C_9), 138.3 (C_q), 136.8 (C_{15}), 130.9 (C_5), 129.5 (CH_{Ar}), 129.0 (2 x CH_{Ar}), 128.2 (CH_{Ar}), 127.9 (3 x CH_{Ar}), 126.3 (C_q), 119.6 (C_8), 116.0 (C_{12}), 90.0 (C_6), 50.8 (C_{14}), 21.7 (C_{13}). HRMS (EI^+) *m/z* calculated for $C_{18}H_{17}N_4$ $[M+H]^+$: 289.1448, found: 289.1449.

4-Benzyl-2-(*m*-tolyl)-2,4-dihydropyrrolo[2,3-*d*][1,2,3]triazole (28b)

The reaction was carried out as described in general procedure **C** using 4-benzyl-1,4-dihydropyrrolo[2,3-*d*][1,2,3]triazole **24** (35 mg, 0.18 mmol, 1.0 eq.) as triazole derivative, 3-iodotoluene (58 mg, 0.27 mmol, 1.5 eq.) as iodoaryl derivative, potassium carbonate (49 mg, 0.35 mmol, 2.0 eq.), Copper(I) thiophene-2-carboxylate (3.81 mg, 0.02 mmol, 0.1 eq.) and *L*-Proline (4.14 mg, 0.04 mmol, 0.2 eq.) in dry DMSO (0.25 M). Crude product was purified by flash chromatography in silica gel column using a gradient (PE: EtOAc) from (1: 0) to (8: 2) yielding to corresponding product **28b** (16 mg, 33 %) as a brown oil.

Or the reaction was carried out as described in general procedure **D** using 4-benzyl-1,4-dihydropyrrolo[2,3-*d*][1,2,3]triazole **24** (40 mg, 0.20 mmol, 1.0 eq.) as triazole derivative, 3-methylphenyl(2,4,6-trimethoxyphenyl)iodonium trifluoroacetate (252 mg, 0.40 mmol, 2.0 eq.) as aryliodonium salt and potassium carbonate (30 mg, 0.24 mmol, 1.2 eq.) in dry toluene (0.15 M). Crude product was purified by flash chromatography on C18-reversed silica gel column using a gradient (H_2O: ACN) from (5: 5) to (4: 6) yielding to corresponding product **28b** (35 mg, 60 %) as a brown oil. Rf: 0.14 (H_2O/ACN: 40/60). IR (ATR diamond, cm^{-1}) ν: 1581, 1359, 1206, 956, 765, 713, 699, 684, 628, 605. ^1H NMR (400 MHz, Chloroform-*d*) δ 7.98 – 7.83 (m, 2H, H-8 and H-12), 7.40 – 7.28 (m, 6H, H-11, H-16, H-17 and H-18), 7.15 – 7.08 (m, 1H, H-10), 7.04 (d, J_{5-6} = 3.4 Hz, 1H, H-5), 6.24 (d, J_{5-6} = 3.4 Hz, 1H, H-6), 5.23 (s, 2H, H-14), 2.44 (s, 3H, H-13). ^{13}C NMR (101 MHz, Chloroform-*d*): δ 150.5 (C_{3a}), 143.0 (C_{6a}), 141.5 (C_7), 139.3 (C_9), 136.9 (C_{15}), 135.1 (C_5), 129.1 (CH_{Ar}), 129.0 (2 x CH_{Ar}), 128.1 (CH_{Ar}), 127.6 (2 x CH_{Ar}), 127.4 (CH_{Ar}), 119.5 (C_8), 116.2 (C_{12}), 93.0 (C_6), 51.0 (C_{14}), 21.6 (C_{13}). HRMS (EI^+) *m/z* calculated for $C_{18}H_{17}N_4$ $[M+H]^+$: 289.1448, found: 289.1451.

4-Benzyl-1-(*o*-tolyl)-1,4-dihydropyrrolo[2,3-*d*][1,2,3]triazole (29a)

The reaction was carried out as described in general procedure **C** using 4-benzyl-1,4-dihydropyrrolo[2,3-*d*][1,2,3]triazole **24** (50 mg, 0.25 mmol, 1.0 eq.) as triazole derivative, 2-iodotoluene (83 mg, 0.38 mmol, 1.5 eq.) as iodoaryl derivative, potassium carbonate (71 mg, 0.51 mmol, 2.0 eq.), Copper(I) thiophene-2-carboxylate (4.77 mg, 0.025 mmol, 0.1 eq.) and *L*-Proline (5.87 mg, 0.051, 0.2 eq.) in dry DMSO (0.25 M). Crude product was purified by flash chromatography in silica gel column using a gradient (PE: EtOAc) from (1: 0) to (8: 2) yielding to corresponding product **29a** (10 mg, 14 %) as a brown oil.

Or the reaction was carried out as described in general procedure **D** using 4-benzyl-1,4-dihydropyrrolo[2,3-*d*][1,2,3]triazole **24** (40 mg, 0.20 mmol, 1.0 eq.) as triazole derivative, 2-methylphenyl(2,4,6-trimethoxyphenyl)iodonium trifluoroacetate (252 mg, 0.40 mmol, 2.0 eq.) as aryliodonium salt and potassium carbonate (30 mg, 0.24 mmol, 1.2 eq.) in dry toluene (0.15 M). Crude product was purified by flash chromatography on C18-reversed silica gel column using a gradient (H_2O: ACN) from (5: 5) to (4: 6) yielding to corresponding product **29a** (5 mg, 7 %) as a brown oil. Rf: 0.08 (H_2O/ACN: 40/60). IR (ATR diamond, cm^{-1}) ν: 1517, 1244, 1175, 1057, 1029, 798, 727, 698, 600. ^1H NMR (400 MHz, Chloroform-*d*): δ 7.50 – 7.45 (m, 1H, H-12), 7.40 – 7.29 (m, 8H, H-9, H-10, H-11, H-16, H-17 and H-18), 6.97 (d, J_{5-6} = 3.2 Hz, 1H, H-5), 5.95 (d, J_{5-6} = 3.2 Hz, 1H, H-6), 5.34 (s, 2H, H-14), 2.33 (s, 3H,

H-13). ^{13}C NMR (101 MHz, Chloroform-*d*): δ 150.1 (C_{3a}), 137.1 (C_q), 136.9 (C_q), 133.5 (C_q), 131.7 (CH_{Ar}), 130.8 (C_5), 129.1 (C_q), 129.0 (2 x CH_{Ar}), 128.9 (CH_{Ar}), 128.2 (CH_{Ar}), 128.0 (2 x CH_{Ar}), 126.9 (CH_{Ar}), 125.8 (CH_{Ar}), 89.1 (C_6), 50.8 (C_{14}), 18.6 (C_{13}). HRMS (EI$^+$) *m/z* calculated for $C_{18}H_{17}N_4$ [M+H]$^+$: 289.0978, found: 289.0785.

4-Benzyl-2-(*o*-tolyl)-2,4-dihydropyrrolo[2,3-*d*][1,2,3]triazole (29b)

The reaction was carried out as described in general procedure **C** using 4-benzyl-1,4-dihydropyrrolo[2,3-*d*][1,2,3]triazole **24** (50 mg, 0.25 mmol, 1.0 eq.) as triazole derivative, 2-iodotoluene (83 mg, 0.38 mmol, 1.5 eq.) as iodoaryl derivative, potassium carbonate (71 mg, 0.51 mmol, 2.0 eq.), Copper(I) thiophene-2-carboxylate (4.77 mg, 0.025 mmol, 0.1 eq.) and *L*-Proline (5.87 mg, 0.05 mmol, 0.2 eq.) in dry DMSO (0.25 M). Crude product was purified by flash chromatography in silica gel column using a gradient (PE: EtOAc) from (1: 0) to (8: 2) yielding to corresponding product **29b** (9 mg, 12 %) as a white solid.

Or The reaction was carried out as described in general procedure **D** using 4-benzyl-1,4-dihydropyrrolo[2,3-*d*][1,2,3]triazole **24** (40 mg, 0.20 mmol, 1.0 eq.) as triazole derivative, 2-methylphenyl(2,4,6-trimethoxyphenyl)iodonium trifluoroacetate (252 mg, 0.40 mmol, 2.0 eq.) as aryliodonium salt and potassium carbonate (30 mg, 0.24 mmol, 1.2 eq.) in dry toluene (0.15 M). Crude product was purified by flash chromatography on C18-reversed silica gel column using a gradient (H_2O: ACN) from (5: 5) to (4: 6) yielding to corresponding product **29b** (31 mg, 42 %) as a white solid. Rf: 0.25 (H_2O/ACN: 40/60). MP: 59–61 °C. IR (ATR diamond, cm^{-1}) ν: 3008, 2923, 2197, 1590, 1514, 1336, 1160, 1128, 1058, 836. ^{1}H NMR (400 MHz, Chloroform-*d*): δ 7.64 – 7.57 (m, 1H, H-12), 7.38 – 7.28 (m, 8H, H-9, H-10, H-11, H-16, H-17, H-18), 7.07 (d, $J_{5\text{-}6}$ = 3.5 Hz, 1H, H-5), 6.27 (d, $J_{5\text{-}6}$ = 3.5 Hz, 1H, H-6), 5.23 (s, 2H, H-14), 2.42 (s, 3H, H-13). ^{13}C NMR (101 MHz, Chloroform-*d*): δ 150.2 (C_{3a}), 142.6 (C_{6a}), 141.0 (C_7), 136.9 (C_{15}), 134.3 (C_5), 133.1 (C_8), 131.7 (CH_{Ar}), 128.9 (2 x CH_{Ar}), 128.3 (CH_{Ar}), 128.1 (CH_{Ar}), 127.7 (2 x CH_{Ar}), 126.5 (CH_{Ar}), 126.0 (C_{12}), 92.9 (C_6), 51.1 (C_{14}), 19.2 (C_{13}). HRMS (EI$^+$) *m/z* calculated for $C_{18}H_{17}N_4$ [M+H]$^+$: 289.1448, found: 289.1449.

4-Benzyl-1-(4-(trifluoromethyl)phenyl)-1,4-dihydropyrrolo[2,3-*d*][1,2,3]triazole (30a)

The reaction was carried out as described in general procedure **C** using 4-benzyl-1,4-dihydropyrrolo[2,3-*d*][1,2,3]triazole **24** (50 mg, 0.25 mmol, 1.0 eq.) as triazole derivative, 1-iodo-4-(trifluoromethyl)benzene (93 mg, 0.34 mmol, 1.5 eq.) as iodoaryl derivative, potassium carbonate (56 mg, 0.40 mmol, 2.0 eq.), Copper(I) thiophene-2-carboxylate (3.81 mg, 0.02 mmol, 0.1 eq.) and *L*-Proline (4.61 mg, 0.04 mmol, 0.2 eq.) in dry DMSO (0.25 M). Crude product was purified by flash chromatography in silica gel column using a gradient (PE: EtOAc) from (1: 0) to (8: 2) yielding to corresponding product **30a** (25 mg, 40 %) as a white solid.

Or the reaction was carried out as described in general procedure **D** using 4-benzyl-1,4-dihydropyrrolo[2,3-*d*][1,2,3]triazole **24** (40 mg, 0.20 mmol, 1.0 eq.) as triazole derivative, 4-(trifluoromethyl)phenyl(2,4,6-trimethoxyphenyl)iodonium trifluoroacetate (223 mg, 0.40 mmol, 2.0 eq.) as aryliodonium salt and potassium carbonate (30 mg, 0.24 mmol, 1.2 eq.) in dry toluene (0.15 M). Crude product was purified by flash chromatography on C18-reversed silica gel column using a gradient (H_2O: ACN) from (5: 5) to (4: 6) yielding to corresponding product **30a** (3 mg, 4 %) as a white solid. **Rf:** 0.08 (H_2O/ACN: 40/60). MP: 163–165 °C. IR (ATR diamond, cm^{-1}) ν: 2926, 2837, 1518, 1482, 1245, 1168, 1112, 1066, 1034, 1010, 849, 765. ^{1}H NMR (400 MHz, Chloroform-*d*): δ 8.01 (d, $J_{8\text{-}9}$ = 8.2 Hz, 2H, H-8), 7.79 (d, $J_{8\text{-}9}$ = 8.2 Hz, 2H, H-9), 7.38 – 7.30 (m, 5H, H-14, H-15 and H-16), 7.07 (d, $J_{5\text{-}6}$ = 3.2 Hz, 1H, H-6), 6.27 (d, $J_{5\text{-}6}$ = 3.2 Hz, 1H, H-5), 5.35 (s, 2H, H-12). ^{13}C NMR (101 MHz, Chloroform-*d*): δ 150.8 (C_{3a}), 140.9 (C_7), 136.5 (C_{13}), 131.4 (C_5), 129.1 (2 x CH_{Ar}), 128.8 (d, *J* = 32.9 Hz, C_{10}), 128.4 (C_{16}), 127.9 (2 x CH_{Ar}), 127.1 (q, *J* = 3.8 Hz, 2 x C_9), 126.0 (C_{6a}), 124.0 (d, *J* = 271.8 Hz, C_{11}), 118.6 (2 x C_8), 90.0 (C_6), 50.9 (C_{12}).

^{19}F NMR (376 MHz, Chloroform-*d*): δ -62.4 (F-11). HRMS (EI$^+$) *m/z* calculated for $C_{18}H_{14}F_3N_4$ [M+H]$^+$: 343.1165, found: 343.1169.

4-Benzyl-2-(4-(trifluoromethyl)phenyl)-2,4-dihydropyrrolo[2,3-*d*][1,2,3]triazole (30b)

The reaction was carried out as described in general procedure **C** using 4-benzyl-1,4-dihydropyrrolo[2,3-*d*][1,2,3]triazole **24** (50 mg, 0.25 mmol, 1.0 eq.) as triazole derivative, 1-iodo-4-(trifluoromethyl)benzene (93 mg, 0.34 mmol, 1.5 eq.) as iodoaryl derivative, potassium carbonate (56 mg, 0.40 mmol, 2.0 eq.), Copper(I) thiophene-2-carboxylate (3.81 mg, 0.02 mmol, 0.1 eq.) and *L*-Proline (4.61 mg, 0.04 mmol, 0.2 eq.) in dry DMSO (0.25 M). Crude product was purified by flash chromatography in silica gel column using a gradient (PE: EtOAc) from (1: 0) to (8: 2) yielding to corresponding product **30b** (18 mg, 21 %) as a white solid.

Or the reaction was carried out as described in general procedure **D** using 4-benzyl-1,4-dihydropyrrolo[2,3-*d*][1,2,3]triazole **24** (40 mg, 0.20 mmol, 1.0 eq.) as triazole derivative, 4-(trifluoromethyl)phenyl(2,4,6-trimethoxyphenyl)iodonium trifluoroacetate (223 mg, 0.40 mmol, 2.0 eq.) as aryliodonium salt and potassium carbonate (30 mg, 0.24 mmol, 1.2 eq.) in dry toluene (0.15 M). Crude product was purified by flash chromatography on C18-reversed silica gel column using a gradient (H_2O: ACN) from (5: 5) to (4: 6) yielding to corresponding product **30b** (22 mg, 32 %) as a white solid. Rf: 0.21 (H_2O/ACN: 40/60). MP: 129–131 °C. IR (ATR diamond, cm^{-1}) ν: 2930, 1482, 1353, 1158, 1098, 901, 844, 752. ^{1}H NMR (400 MHz, Chloroform-*d*): δ 8.22 (d, $J_{8\text{-}9}$ = 8.5 Hz, 2H, H-8), 7.70 (d, $J_{8\text{-}9}$ = 8.5 Hz, 2H, H-9), 7.41 – 7.23 (m, 5H, H-14, H-15 and H-16), 7.10 (d, $J_{5\text{-}6}$ = 3.5 Hz, 1H, H-5), 6.24 (d, $J_{5\text{-}6}$ = 3.5 Hz, 1H, H-6), 5.22 (s, 2H, H-12). ^{13}C NMR (101 MHz, Chloroform-*d*): δ 151.0 (C_{3a}), 144.1 (C_{6a}), 143.8 (C_7), 136.6 (C_{13}), 136.6 (C_5), 129.0 (2 x CH_{Ar}), 128.3 (C_{16}), 128.2 (d, *J* = 32.7 Hz, C_{10}), 127.6 (2 x CH_{Ar}), 126.6 (q, *J* = 3.8 Hz, 2 x C_9), 124.2 (d, *J* = 271.7 Hz, C_{11}), 118.7 (2 x C_8), 93.1 (C_6), 51.0 (C_{12}). ^{19}F NMR (376 MHz, Chloroform-*d*): δ -62.2 (F-11). HRMS (EI$^+$) *m/z* calculated for $C_{18}H_{14}F_3N_4$ [M+H]$^+$: 343.1165, found: 343.1165.

4-Benzyl-2-(4-nitrophenyl)-2,4-dihydropyrrolo[2,3-*d*][1,2,3]triazole (31b)

The reaction was carried out as described in general procedure **D** using 4-benzyl-1,4-dihydropyrrolo[2,3-*d*][1,2,3]triazole **24** (40 mg, 0.20 mmol, 1.0 eq.) as triazole derivative, 4-nitrophenyl(2,4,6-trimethoxyphenyl)iodonium trifluoroacetate (213 mg, 0.40 mmol, 2.0 eq.) as aryliodonium salt and potassium carbonate (30 mg, 0.24 mmol, 1.2 eq.) in dry toluene (0.15 M). Crude product was purified by flash chromatography on C18-reversed silica gel column using a gradient (H_2O: ACN) from (5: 5) to (4: 6) yielding to corresponding product **31b** (15 mg, 23 %) as a brown solid. Rf: 0.11 (H_2O/ACN: 40/60). MP: 95–97 °C. IR (ATR diamond, cm^{-1}) ν: 3064, 2365, 1696, 1684, 1616, 1524, 1328. ^{1}H NMR (400 MHz, Acetone-d_6): δ 8.40 (d, $J_{8\text{-}9}$ = 9.3 Hz, 2H, H-9), 8.32 (d, $J_{8\text{-}9}$ = 9.3 Hz, 2H, H-10), 7.59 (d, $J_{5\text{-}6}$ = 3.5 Hz, 1H, H-5), 7.45 – 7.29 (m, 5H, H-14, H-15 and H-16), 6.29 (d, $J_{5\text{-}6}$ = 3.5 Hz, 1H, H-6), 5.34 (s, 2H, H-12). ^{13}C NMR (101 MHz, Acetone-d_6): δ 152.1 (C_{3a}), 146.5 (C_q), 146.4 (C_q), 146.1 (C_q), 140.0 (C_5), 138.1 (C_q), 129.6 (2 x CH_{Ar}), 128.8 (CH_{Ar}), 128.5 (2 x CH_{Ar}), 126.1 (2 x C_9), 119.3 (2 x C_8), 93.0 (C_6), 51.3 (C_{12}). HRMS (EI$^+$) *m/z* calculated for $C_{17}H_{14}N_5O_2$ [M+H]$^+$: 320.1142, found: 320.1147.

4-Benzyl-2-(6-chloropyridin-3-yl)-2,4-dihydropyrrolo[2,3-*d*][1,2,3]triazole (32b)

The reaction was carried out as described in general procedure **D** using 4-benzyl-1,4-dihydropyrrolo[2,3-*d*][1,2,3]triazole **24** (40 mg, 0.20 mmol, 1.0 eq.) as triazole derivative, (6-chloropyridin-3-yl)(2,4,6-trimethoxyphenyl)iodonium trifluoroacetate (210 mg, 0.40 mmol, 2.0 eq.) as aryliodonium salt and potassium carbonate (34 mg, 0.24 mmol, 1.2 eq.) in dry toluene (0.15 M). Crude product was purified by flash chromatography on C18-reversed silica gel column using a gradient (H_2O: ACN) from (5: 5) to (4: 6) yielding to corresponding product **32b** (12 mg, 19 %) as a brown solid. Rf:

0.31 (H_2O/ACN: 40/60). MP: 120–122 °C. IR (ATR diamond, cm^{-1}) ν: 3085, 2950, 1608, 1460, 1353, 1124, 948, 743. ^1H NMR (400 MHz, Chloroform-*d*): δ 9.15 (d, J_{8-12} = 2.7 Hz, 1H, H-8), 8.34 (dd, J_{11-12} = 8.7, J_{8-12} = 2.7 Hz, 1H, H-12), 7.40 (d, J_{11-12} = 8.7 Hz, 1H, H-11), 7.38 – 7.27 (m, 5H, H-16, H-17 and H-18), 7.12 (d, J_{5-6} = 3.4 Hz, 1H, H-5), 6.24 (d, J_{5-6} = 3.4 Hz, 1H, H-6), 5.22 (s, 2H, H-14). ^{13}C NMR (101 MHz, Chloroform-*d*): δ 151.0 (C_{3a}), 148.3 (C_7), 144.2 (C_{6a}), 140.0 (C_8), 137.1 (C_q), 136.8 (C_5), 136.5 (C_{15}), 129.1 (C_q), 128.6 (2 x CH_{Ar}), 128.3 (CH_{Ar}), 127.6 (CH_{Ar}), 124.5 (2 x CH_{Ar}), 93.1 (C_6), 51.1 (C_{14}). HRMS (EI^+) *m/z* calculated for $C_{16}H_{13}ClN_5$ $[M+H]^+$: 310.0856, found: 310.0854.

3,3'-(Pyrrolo[2,3-*d*][1,2,3]triazol-1,4-diyl)dipropanenitrile (33)

3-(3-oxopyrrolidin-1-yl)propanenitrile **5** (207 mg, 1.50 mmol, 1.0 eq.), 3-aminopropanenitrile (315 mg, 4.50 mmol, 3.0 eq.), 1-Azido-4-nitrobenzene (1.23 g, 7.50 mmol, 5.0 eq.), acetic acid (0.03 mL, 0.45 mmol, 0.3 eq.) and molecular sieve (3 Å) were added at anhydrous toluene (0.25 M) and reactional mixture was stirred 1h at 140°C under microwave irradiation. The resulting mixture was reduced under vacuum and filtered through a pad of charcoal. The crude mixture was purified by flash chromatography on silica gel using first DCM and then (PE/EtOAc: 60/40) yielding to corresponding product **33** (200 mg, 62 %) as a white solid. Rf: 0.15 (PE/EtOAc: 30/70). MP: 155–157 °C. IR (ATR diamond, cm^{-1}) ν: 3108, 2958, 2935, 2245, 1576, 1524, 1486, 1372, 1299, 1228, 1095, 979, 743. ^1H NMR (400 MHz, Chloroform-*d*): δ 7.06 (d, J_{5-6} = 3.2 Hz, 1H, H-5), 6.12 (d, J_{5-6} = 3.2 Hz, 1H, H-6), 4.75 (t, J_{7-8} = 6.8 Hz, 2H, H-7), 4.40 (t, J_{10-11} = 6.6 Hz, 2H, H-10), 3.04 (m, 4H, H-8 and H-11). ^{13}C NMR (101 MHz, Chloroform-*d*): δ 149.8 (C_{3a}), 131.5 (C_5), 128.7 (C_{6a}), 117.0 (C_{12}), 116.8 (C_9), 89.2 (C_6), 45.3 (C_7), 43.2 (C_{10}), 19.3 (CH_2), 19.0 (CH_2). HRMS (EI^+) *m/z* calculated for $C_{10}H_{11}N_6$ $[M+H]^+$: 215.1040, found: 215.1044.

3,3'-(6-Bromopyrrolo[2,3-*d*][1,2,3]triazol-1,4-diyl)dipropanenitrile (34)

Under inert gas, 3,3'-(pyrrolo[2,3-*d*][1,2,3]triazol-1,4-diyl)dipropanenitrile **33** (100 mg, 0.47 mmol, 1.0 eq.) was dissolved in dry DCM (0.15 M). *N*-bromosuccinimide (89 mg, 0.47 mmol, 1.0 eq.) was added in one portion and the mixture was stirred 2 hours at room temperature. After full consumption of reactant, water was added and the mixture was extracted with DCM. The resultant organic layer was washed with brine, dried over MgSO4 and solvents were removed under reduced pressure yielding to the pure corresponding product **34** (112 mg, 82 %) as a white solid. Rf: 0.35 (PE/EtOAc: 30/70). MP: 133–135 °C. IR (ATR diamond, cm^{-1}) ν: 3121, 2937, 2369, 2240, 1716, 1558, 1412, 1181, 1094, 1020, 878. ^1H NMR (400 MHz, Chloroform-*d*): δ 7.06 (s, 1H, H-5), 4.80 (t, J_{7-8} = 7.0 Hz, 2H, H-7), 4.38 (t, J_{10-11} = 6.5 Hz, 2H, H-10), 3.12 (t, J_{7-8} = 7.0 Hz, 2H, H-8), 3.03 (t, J_{10-11} = 6.5 Hz, 2H, H-11). ^{13}C NMR (101 MHz, Chloroform-*d*): δ 149.1 (C_{3a}), 130.2 (C_5), 126.9 (C_{6a}), 116.7 (C_{12}), 116.1 (C_9), 75.3 (C_6), 44.5 (C_7), 43.4 (C_{10}), 19.5 (C_8), 19.3 (C_{11}). HRMS (EI^+) *m/z* calculated for $C_{10}H_{10}BrN_6$ $[M+H]^+$: 293.0145, found: 293.0146.

3,3'-(6-(*p*-Tolyl)pyrrolo[2,3-*d*][1,2,3]triazol-1,4-diyl)dipropanenitrile (35)

Under inert gas, 3,3'-(6-bromopyrrolo[2,3-*d*][1,2,3]triazol-1,4-diyl)dipropanenitrile **34** (94 mg, 0.32 mmol, 1.0 eq.), *p*-tolylboronic acid (65 mg, 0.48 mmol, 1.5 eq.), potassium phosphate tribasic (139 mg, 0.64 mmol, 2.0 eq.), Palladium(II) acetate (2.2 mg, 0.01 mmol, 0.03 eq.) and RuPhos (9 mg, 0.02 mmol, 0.06 eq.) were dissolved in dry 1,4-dioxane (0.15 M) and the mixture was stirred 1h at 120 °C under microwave irradiation. After reaction, the mixture was filtered through a pad of celite and the filtrate was reduced to dryness under vacuum. Residue was taken up in CH_2Cl_2, washed with H_2O and dried over $MgSO_4$. After being concentrated under vacuum, the residue was purified by flash chromatography on silica gel using a gradient of (PE: EtOAc) from (1: 0) to (6: 4) yielding to corresponding product **35** (84 mg, 87 %) as a white solid. Rf: 0.31 (PE/EtOAc: 30/70). MP: 117–119 °C. IR (ATR diamond, cm^{-1}) ν: 3100, 2920, 2356 2353, 1576, 1558, 1424, 1200, 1063, 812. ^1H NMR (400 MHz, Chloroform-*d*): δ 7.32 (d, J_{14-15} = 8.1 Hz, 2H, H-14), 7.27 (d, J_{14-15} = 7.7 Hz, 2H, H-15), 7.12 (s, 1H, H-5), 4.80 (t, J_{10-11} = 7.2 Hz, 2H, H-10), 4.43 (t, J_{7-8} = 6.6 Hz, 2H, H-7), 3.07 (t, J_{7-8} = 6.6 Hz, 2H, H-8), 2.93

(t, $J_{10\text{-}11}$ = 7.2 Hz, 2H, H-11), 2.40 (s, 3H, H-17). ^{13}C NMR (101 MHz, Chloroform-*d*): δ 150.2 (C_{3a}), 137.3 (C_{16}), 130.2 (2 x C_{15}), 129.4 (C_q), 128.1 (C_5), 127.3 (2 x C_{14}), 126.5 (C_q), 117.1 (C_9), 116.2 (C_{12}), 107.7 (C_6), 45.4 (C_{10}), 43.2 (C_7), 21.3 (C_{17}), 19.4 (C_8), 19.1 (C_{11}). HRMS (EI$^+$) *m/z* calculated for $C_{17}H_{17}N_6$ [M+H]$^+$: 305.1509, found: 305.1508.

3-(6-(*p*-Tolyl)pyrrolo[2,3-*d*][1,2,3]triazol-4(1*H*)-yl)propanenitrile (36)

The reaction was carried out as described in general procedure **A** using 3,3'-(6-(*p*-tolyl)pyrrolo[2,3-*d*][1,2,3]triazol-1,4-diyl)dipropanenitrile **35** (74 mg, 0.24 mmol, 1.0 eq.) and a 1.0 M solution of potassium tert-butoxide in EtOH (1.65 mL, 1.65 mmol, 6.8 eq.). Crude product was purified by flash chromatography on silica gel using a gradient of (PE/EtOAc) from (1: 0) to (1: 1) yielding to corresponding product **36** (37 mg, 63 %) as a white solid. Rf: 0.48 (EtOAc: 100). MP: 90–92 °C. IR (ATR diamond, cm^{-1}) ν: 3147, 2918, 2855, 2353, 1558, 1506, 1346, 1196, 1152, 874, 781. ^{1}H NMR (400 MHz, Methanol-*d*$_4$): δ 7.65 (s, 1H, H-5), 7.63 (d, $J_{11\text{-}12}$ = 8.2 Hz, 2H, H-11), 7.20 (d, $J_{11\text{-}12}$ = 8.2 Hz, 2H, H-12), 4.41 (t, $J_{7\text{-}8}$ = 6.6 Hz, 2H, H-7), 3.10 (t, $J_{7\text{-}8}$ = 6.6 Hz, 2H, H-8), 2.34 (s, 3H, H-14). ^{13}C NMR (101 MHz, Methanol-*d*$_4$): δ 150.5 (C_{3a}), 136.7 (C_q), 136.3 (C_q), 131.5 (C_q), 130.5 (2 x C_{12}), 130.4 (C_5), 129.6 (C_q), 125.9 (2 x C_{11}), 118.8 (C_9), 44.1 (C_7), 21.2 (C_{14}), 19.3 (C_8). HRMS (EI$^+$) *m/z* calculated for $C_{14}H_{14}N_5$ [M+H]$^+$: 252.1244, found: 252.1245.

3-(1,6-Di-*p*-tolylpyrrolo[2,3-*d*][1,2,3]triazol-4(1*H*)-yl)propanenitrile (37)

The reaction was carried out as described in general procedure **D** using 3-(6-(*p*-tolyl)pyrrolo[2,3-*d*][1,2,3]triazol-4(1*H*)-yl)propanenitrile **36** (45 mg, 0.18 mmol, 1.0 eq.) as triazole derivative, 4-methylphenyl(2,4,6-trimethoxyphenyl)iodonium trifluoroacetate (178 mg, 0.36 mmol, 2.0 eq.) as aryliodonium salt and potassium carbonate (30 mg, 0.22 mmol, 1.2 eq.) in dry toluene (0.15 M). Crude product was purified by flash chromatography on C18-reversed silica gel column using a gradient (H_2O: ACN) from (5: 5) to (4: 6) yielding to corresponding product **37a** (4 mg, 7 %) as a white solid. Rf: 0.30 (PE/EtOAc: 90/10). MP: 197–199 °C. IR (ATR diamond, cm^{-1}) ν: 2923, 2249, 1575, 1558, 1418, 1200, 1083, 1034, 815, 740. ^{1}H NMR (400 MHz, Chloroform-*d*): δ 7.38 (d, $J_{8\text{-}9}$ = 8.4 Hz, 2H, H-8), 7.24 – 7.21 (m, 3H, H-5 and H-9), 7.11 – 7.05 (m, 4H, H-16 and H-17), 4.51 (t, $J_{12\text{-}13}$ = 6.6 Hz, 2H, H-12), 3.14 (t, $J_{12\text{-}13}$ = 6.6 Hz, 2H, H-13), 2.44 (s, 3H, H-11), 2.38 (s, 3H, H-19). ^{13}C NMR (101 MHz, Chloroform-*d*): δ 150.1 (C_{6a}), 138.4 (C_{10}), 136.6 (C_{18}), 135.2 (C_q), 129.6 (2 x CH_{Ar}), 129.1 (C_q), 129.1 (2 x CH_{Ar}), 128.3 (2 x CH_{Ar}), 128.1 (C_5), 125.4 (C_q), 123.5 (2 x C_8), 117.2 (C_{14}), 108.5 (C_q), 43.2 (C_{12}), 21.3 (C_{19}), 21.3 (C_{11}), 19.4 (C_{13}). HRMS (EI$^+$) *m/z* calculated for $C_{21}H_{20}N_5$ [M+H]$^+$: 342.1713, found: 342.1709.

3-(2,6-Di-*p*-tolylpyrrolo[2,3-*d*][1,2,3]triazol-4(1*H*)-yl)propanenitrile (37b)

The reaction was carried out as described in general procedure **D** using 3-(6-(*p*-Tolyl)pyrrolo[2,3-*d*][1,2,3]triazol-4(1*H*)-yl)propanenitrile **36** (45 mg, 0.18 mmol, 1.0 eq.) as triazole derivative, 4-methylphenyl(2,4,6-trimethoxyphenyl)iodonium trifluoroacetate (178 mg, 0.36 mmol, 2.0 eq.) as aryliodonium salt and potassium carbonate (30 mg, 0.22 mmol, 1.2 eq.) in dry toluene (0.15 M). Crude product was purified by flash chromatography on C18-reversed silica gel column using a gradient (H_2O: ACN) from (5: 5) to (4: 6) yielding to corresponding product **37b** (28 mg, 46 %) as a white solid. Rf: 0.10 (PE/EtOAc: 90/10). MP: 193–195 °C. IR (ATR diamond, cm^{-1}) ν: 2922, 2254, 1575, 1558, 1200, 1063, 815, 740, 707. ^{1}H NMR (400 MHz, Chloroform-*d*): δ 8.04 (d, $J_{8\text{-}9}$ = 8.5 Hz, 2H, H-8), 7.76 (d, $J_{16\text{-}17}$ = 8.1 Hz, 2H, H-16), 7.36 (s, 1H, H-5), 7.29 (d, $J_{8\text{-}9}$ = 8.5 Hz, 2H, H-9), 7.24 (d, $J_{16\text{-}17}$ = 8.1 Hz, 2H, H-17), 4.34 (t, $J_{12\text{-}13}$ = 6.8 Hz, 2H, H-12), 3.00 (t, $J_{12\text{-}13}$ = 6.8 Hz, 2H, H-13), 2.41 (s, 3H, H-11), 2.38 (s, 3H, H-19). ^{13}C NMR (101 MHz, Chloroform-*d*): δ 149.8 (C_{3a}), 141.4 (C_{6a}), 139.1 (C_q), 136.9 (C_{10}), 136.2 (C_{18}), 129.9 (2 x C_9), 129.7 (C_q), 129.6 (2 x C_{17}), 129.1 (C_5), 125.3 (2 x C_{16}), 119.1 (2 x C_8), 117.1 (C_{14}), 110.9 (C_q), 43.4 (CH_2), 21.4 (C_{19}), 21.1 (C_{11}), 18.8 (C_{13}). HRMS (EI$^+$) *m/z* calculated for $C_{21}H_{20}N_5$ [M+H]$^+$: 342.1713, found: 342.1711.

2,6-Di-*p*-tolyl-2.4-dihydropyrrolo[2,3-*d*][1,2,3]triazole (38b)

The reaction was carried out as described in general procedure **A** using 3-(2,6-Di-*p*-tolylpyrrolo[2,3-*d*][1,2,3]triazol-4(1*H*)-yl)propanenitrile **37b** (22 mg, 0.07 mmol, 1.0 eq.) and a 1.0 M solution of potassium tert-butoxide in EtOH (0.44 mL, 0.44 mmol, 6.8 eq.). Crude product was purified by flash chromatography on silica gel using a gradient (PE: EtOAc) from (1: 0) to (8: 2) yielding to corresponding product **38b** (16 mg, 85 %) as a white solid. Rf: 0.43 (EP/EtOAc: 80/20). MP: 218–220 °C. IR (ATR diamond, cm^{-1}) ν: 3100, 2922, 2842, 2352, 1575, 1558, 1518, 1418, 1200, 1083, 1063, 1035, 815. ^1H NMR (400 MHz, Methanol-d_4): δ 8.00 (d, $J_{8\text{-}9}$ = 8.5 Hz, 2H, H-8), 7.78 (d, $J_{13\text{-}14}$ = 8.1 Hz, 2H, H-13), 7.69 (s, 1H, H-5), 7.31 (d, $J_{8\text{-}9}$ = 8.5 Hz, 2H, H-9), 7.21 (d, $J_{13\text{-}14}$ = 8.1 Hz, 2H, H-14), 2.40 (s, 3H, H-11), 2.35 (s, 3H, H-16). ^{13}C NMR (101 MHz, Methanol-d_4): δ 151.6 (C_{3a}), 142.7 (C_{6a}), 140.6 (C_q), 137.7 (C_q), 136.3 (C_q), 132.0 (C_q), 130.8 (2 x C_9), 130.3 (2 x C_{14}), 129.2 (C_5), 126.0 (2 x C_{13}), 119.8 (2 x C_8), 110.2 (C_6), 21.2 (C_{16}), 21.0 (C_{11}). HRMS (EI^+) *m/z* calculated for $C_{18}H_{17}N_4$ $[M+H]^+$: 289.1448, found: 289.1452.

1,4,6-Tri-*p*-tolyl-1,4-dihydrpyrrolo[2,3-*d*][1,2,3]triazole (39a)

The reaction was carried out as described in general procedure **D** using 4,6-Di-*p*-tolyl-1,4-dihydropyrrolo[2,3-*d*][1,2,3]triazole **45** (40 mg, 0.14 mmol, 1.0 eq.) as triazole derivative, 4-methylphenyl(2,4,6-trimethoxyphenyl)iodonium trifluoroacetate (140 mg, 0.28 mmol, 2.0 eq.) as aryliodonium salt and potassium carbonate (23 mg, 0.17 mmol, 1.2 eq.) in dry toluene (0.15 M). Crude product was purified by flash chromatography on silica gel using a gradient (PE: EtOAc) from (1: 0) to (7: 3) yielding to corresponding product **39a** (8 mg, 15 %) as a white solid. Rf: 0.10 (PE/EtOAc: 80/20). MP: 195–197 °C. IR (ATR diamond, cm^{-1}) ν: 2919, 2856, 1519, 1344, 1211, 1107, 1059, 812, 799, 669. ^1H NMR (400 MHz, Chloroform-*d*): δ 8.13 (d, $J_{8\text{-}9}$ = 8.5 Hz, 2H, H-8), 7.89 (s, 1H, H-5), 7.86 (d, $J_{13\text{-}14}$ = 8.1 Hz, 2H, H-13), 7.74 (d, $J_{18\text{-}19}$ = 8.5 Hz, 2H, H-18), 7.33 – 7.27 (m, 6H, H-9, H-14 and H-19), 2.43 (s, 3H, H-11), 2.40 (s, 6H, H-16 and H-21). ^{13}C NMR (101 MHz, Chloroform-*d*): δ 149.2 (C_{3a}), 142.1 (C_q), 139.3 (C_q), 136.8 (C_q), 136.6 (C_q), 136.4 (C_q), 136.0 (C_q), 134.2 (C_q), 130.4 (2 x CH_{Ar}), 129.9 (2 x CH_{Ar}), 129.7 (2 x CH_{Ar}), 125.5 (2 x C_{13}), 125.3 (C_5), 119.2 (2 x C_8), 117.8 (2 x C_{18}), 112.0 (C_q), 21.4 (CH_3), 21.2 (CH_3), 21.0 (CH_3). HRMS (EI^+) *m/z* calculated for $C_{25}H_{23}N_4$ $[M+H]^+$: 379.1917, found: 379.1926.

2,4,6-tri-*p*-tolyl-2,4-dihydropyrrolo[2,3-*d*][1,2,3]triazole (39b)

The reaction was carried out as described in general procedure **B** using 2,6-Di-*p*-tolyl-2.4-dihydropyrrolo[2,3-*d*][1,2,3]triazole **38** (12 mg, 0.04 mmol, 1.0 eq.), 1-iodo-4-methylbenzene (14 mg, 0.06 mmol, 1.5 eq.) as iodoaryl derivative, Copper(I) thiophene-2-carboxylate (0.8 mg, 0.004 mmol, 0.1 eq.), *L*-Proline (0.9 mg, 0.008 mmol, 0.2 eq.) and potassium carbonate (27 mg, 0.08 mmol, 2.0 eq.) in dry DMSO (0.25 M). Crude product was purified by flash chromatography on silica gel using a gradient (PE: EtOAc) from (1: 0) to (7: 3) yielding to corresponding product **39b** (15 mg, 94 %) as a white solid.

Or the reaction was carried out as described in general procedure **D** using 4,6-Di-*p*-tolyl-1,4-dihydropyrrolo[2,3-*d*][1,2,3]triazole **45** (40 mg, 0.14 mmol, 1.0 eq.) as triazole derivative, 4-methylphenyl(2,4,6-trimethoxyphenyl)iodonium trifluoroacetate (138 mg, 0.28 mmol, 2.0 eq.) as aryliodonium salt and potassium carbonate (23 mg, 0.17 mmol, 1.2 eq.) in dry toluene (0.15 M). Crude product was purified by flash chromatography on C18-reversed silica gel column using a gradient (H_2O: ACN) from (5: 5) to (4: 6) yielding to corresponding product **39b** (39 mg, 74 %) as a white solid. Rf: 0.12 (EP: 100). MP: 219–221 °C. IR (ATR diamond, cm^{-1}) ν: 3094, 2922, 2248, 1558, 1518, 1200, 1083, 1063, 1035, 943, 815, 628. ^1H NMR (400 MHz, Chloroform-*d*): δ 8.07 (d, $J_{8\text{-}9}$ = 8.5 Hz, 2H, H-8), 7.83 (s, 1H, H-5), 7.80 (d, $J_{13\text{-}14}$ = 8.2 Hz, 2H, H-13), 7.68 (d, $J_{18\text{-}19}$ = 8.5 Hz, 2H, H-18), 7.26 – 7.19 (m, 6H, H-9, H-14 and H-19), 2.37 (s, 3H, H-11), 2.34 (s, 6H, H-16 and H-21). ^{13}C NMR (101 MHz, Chloroform-*d*): δ 149.2 (C_{3a}), 142.1 (C_{6a}), 139.3 (C_q), 136.8 (C_q), 136.4 (C_q), 136.0 (C_q), 134.2 (C_q), 130.3 (2 x CH_{Ar}), 129.9 (2 x CH_{Ar}),

129.7 (C_q), 129.7 (2 x CH_{Ar}), 125.5 (2 x C_{13}), 125.3 (C_5), 119.2 (2 x C_8), 117.8 (2 x C_{18}), 112.0 (C_q), 21.4 (CH_3), 21.2 (CH_3), 21.0 (CH_3). HRMS (EI^+) *m/z* calculated for $C_{25}H_{23}N_4$ $[M+H]^+$: 379.1917, found: 379.1920

3-(1-Allylpyrrolo[2,3-*d*][1,2,3]triazol-4(1*H*)-yl)propanenitrile (40)

3-(3-oxopyrrolidin-1-yl)propanenitrile **5** (210 mg, 1.50 mmol, 1.0 eq.), allyl amine (257 mg, 4.50 mmol, 3.0 eq.), 1-Azido-4-nitrobenzene (1.23 g, 7.50 mmol, 5.0 eq.), acetic acid (0.025 mL, 0.45 mmol, 0.3 eq.) and molecular sieve (3 Å) were added at anhydrous toluene (0.25 M) and reactional mixture was stirred 1h at 140°C under microwave irradiation. The resulting mixture was reduced under vacuum and filtered through a pad of charcoal. The crude mixture was purified by flash chromatography on silica gel using first DCM and then (PE/EtOAc: 40/60) yielding to corresponding product **40** (165 mg, 54 %) as a yellow solid. Rf: 0.23 (PE/EtOAc: 50/50). MP: 57–59 °C. IR (ATR diamond, cm^{-1}) ν: 3108, 2936, 2248, 1519, 1491, 1367, 1179, 1088, 987, 736, 698, 645. ^1H NMR (400 MHz, Chloroform-*d*): δ 6.99 (d, J_{5-6} = 2.6 Hz, 1H, H-5), 6.06 (m, 1H, H-8), 5.97 (d, J_{5-6} = 2.6 Hz, 1H, H-6), 5.38 – 5.31 (m, 2H, H-9), 5.10 – 5.00 (m, 2H, H-7), 4.38 (t, J_{10-11} = 6.8 Hz, 2H, H-10), 3.02 (t, J_{10-11} = 6.8 Hz, 2H, H-11). ^{13}C NMR (101 MHz, Chloroform-*d*): δ 149.8 (C_{3a}), 131.2 (C_8), 130.8 (C_5), 128.7 (C_{6a}), 119.9 (C_9), 117.2 (C_{12}), 89.4 (C_6), 52.4 (C_7), 43.1 (C_{10}), 19.3 (C_{11}). HRMS (EI^+) *m/z* calculated for $C_{10}H_{12}N_5$ $[M+H]^+$: 202.1087, found: 202.1089.

3-(1-Allyl-6-bromopyrrolo[2,3-*d*][1,2,3]triazol-4(1*H*)-yl)propanenitrile (41)

Under inert gas, 3-(1-Allylpyrrolo[2,3-*d*][1,2,3]triazol-4(1*H*)-yl)propanenitrile **40** (131 mg, 0.65 mmol, 1.0 eq.) was dissolved in dry DCM (0.15 M). *N*-bromosuccinimide (123 mg, 0.65 mmol, 1.0 eq.) was added in one portion and the mixture was stirred 2 hours at room temperature. After full consumption of reactants, water was added and the mixture was extracted with DCM. The resultant organic layer was washed with brine, dried over $MgSO_4$ and solvents were removed under reduced pressure yielding to the pure corresponding product **41** (155 mg, 81 %) as a brown solid. Rf: 0.44 (PE/EtOAc: 50/50). MP: 84–86 °C. IR (ATR diamond, cm^{-1}) ν: 3117, 2933, 2248, 1518, 1447, 1177, 1080, 948, 816, 698. ^1H NMR (400 MHz, Chloroform-*d*): δ 7.01 (s, 1H, H-5), 6.19 – 6.01 (m, 1H, H-8), 5.40 – 5.25 (m, 2H, H-9), 5.13 – 5.08 (m, 2H, H-7), 4.36 (t, J_{10-11} = 6.5 Hz, 2H, H-10), 3.02 (t, J_{10-11} = 6.5 Hz, 2H, H-11). ^{13}C NMR (101 MHz, Chloroform-*d*): δ 149.1 (C_{3a}), 131.8 (C_8), 129.6 (C_5), 126.9 (C_{6a}), 119.6 (C_9), 116.7 (C_{12}), 75.7 (C_6), 51.6 (C_7), 43.2 (C_{10}), 19.2 (C_{11}). HRMS (EI^+) *m/z* calculated for $C_{10}H_{11}BrN_5$ $[M+H]^+$: 280.0192, found: 280.0195.

3-(1-Allyl-6-(*p*-tolyl)pyrrolo[2,3-*d*][1,2,3]triazol-4(1*H*)-yl)propanenitrile (42)

Under inert gas, 3-(1-Allyl-6-bromopyrrolo[2,3-*d*][1,2,3]triazol-4(1*H*)-yl)propanenitrile **41** (400 mg, 1.43 mmol, 1.0 eq.), *p*-tolylboronic acid (292 mg, 2.15 mmol, 1.5 eq.), potassium phosphate tribasic (608 mg, 2.87 mmol, 2.0 eq.), palladium(II) acetate (10 mg, 0.04 mmol, 0.03 eq.) and RuPhos (41 mg, 0.09 mmol, 0.06 eq.) were dissolved in dry 1,4-dioxane (0.15 M) and the mixture was stirred 1h at 120 °C under microwave irradiation. After reaction, the mixture was filtered through a pad of celite and the filtrate was reduced to dryness under vacuum. Residue was taken up in CH_2Cl_2, washed with H_2O and dried over $MgSO_4$. After being concentrated under vacuum, the residue was purified by flash chromatography on silica gel using a gradient of (PE: EtOAc) from (1: 0) to (6: 4) yielding to corresponding product **42** (332 mg, 80 %) as a white solid. Rf: 0.38 (PE/EtOAc: 50/50). MP: 117–119 °C. IR (ATR diamond, cm^{-1}) ν: 3117, 2938, 2249, 1540, 1447, 1367, 1182, 1079, 1000, 829, 761. ^1H NMR (400 MHz, Chloroform-*d*): δ 7.35 (d, J_{14-15} = 7.7 Hz, 2H, H-14), 7.21 (d, J_{14-15} = 7.7 Hz, 2H, H-15), 7.09 (s, 1H, H-5), 6.00 (ddt, J_{8-9} = 16.8, J_{8-9} = 10.3, J_{7-8} = 5.3 Hz, 1H, H-8), 5.20 (d, J_{8-9} = 10.3 Hz, 1H, H-9), 5.15 (d, J_{7-8} = 5.3 Hz, 2H, H-7), 5.04 (d, J_{8-9} = 16.8 Hz, 1H, H-9), 4.42 (t, J_{10-11} = 6.7 Hz, 2H, H-10), 3.06 (t, J_{10-11} = 6.7 Hz, 2H, H-11), 2.38 (s, 3H, H-17). ^{13}C NMR (101 MHz, Chloroform-*d*): δ 150.3 (C_{3a}), 136.8 (C_q), 132.3 (C_8), 129.8 (C_q), 129.7 (2 x C_{15}), 127.5 (C_5), 127.3 (2 x C_{14}), 126.6 (C_q), 118.8 (C_9), 117.2 (C_{12}), 108.0 (C_6), 52.4 (C_7), 43.1 (C_{10}), 21.3 (C_{17}), 19.3 (C_{11}). HRMS (EI^+) *m/z* calculated for $C_{17}H_{18}N_5$ $[M+H]^+$: 292.1557, found: 292.1556.

1-Allyl-6-(*p*-tolyl)-1,4-dihydrpyrrolo[2,3-*d*][1,2,3]triazole (43)

The reaction was carried out as described in general procedure **A** using 3-(2-Allyl-6-(*p*-tolyl)pyrrolo[2,3-*d*][1,2,3]triazol-4(2*H*)-yl)propanenitrile **42** (53 mg, 0.18 mmol, 1.0 eq.) and a 1.0 M solution of potassium tert-butoxide in EtOH (1.23 mL, 1.23 mmol, 6.8 eq.). Crude product was purified by flash chromatography on silica gel using a gradient (DCM: MeOH) from (1: 0) to (95: 5) yielding to corresponding product **43** (30 mg, 72 %) as an off-white solid. Rf: 0.42 (PE/EtOAc: 50/50). MP: 126–128 °C. IR (ATR diamond, cm^{-1}) ν: 3181, 3111, 2916, 2843, 2350, 1533, 1323, 1189, 977, 818, 717. ^{1}H NMR (400 MHz, Chloroform-*d*): δ 9.43 (s, 1H, N_4-H), 7.38 (d, J_{11-12} = 8.0 Hz, 2H, H-11), 7.24 – 7.19 (m, 3H, H-5 and H-12), 6.03 (ddt, J_{8-9} = 17.1, J_{7-8} = 10.6, J_{7-8} = 5.5 Hz, 1H, H-8), 5.23 – 5.15 (m, 3H, H-7 and H-9), 5.05 (d, J_{8-9} = 17.1 Hz, 1H, H-9), 2.39 (s, 3H, H-14). ^{13}C NMR (101 MHz, Chloroform-*d*): δ 150.8 (C_{3a}), 136.3 (C_q), 132.3 (C_8), 130.3 (C_q), 129.5 (2 x C_{12}), 127.2 (2 x C_{11}), 126.4 (C_{6a}), 124.9 (C_5), 118.5 (C_9), 107.8 (C_6), 52.2 (C_7), 21.1 (C_{14}). HRMS (EI^+) *m/z* calculated for $C_{14}H_{15}N_4$ $[M+H]^+$: 239.1291, found: 239.1290.

1-Allyl-4,6-di-*p*-tolyl-1,4-dihydrpyrrolo[2,3-*d*][1,2,3]triazole (44)

The reaction was carried out as described in general procedure **B** using 6-(*p*-Tolyl)-1,4-dihydropyrrolo[2,3-*d*][1,2,3]triazole **43** (60 mg, 0.25 mmol, 1.0 eq.), 1-iodo-4-methylbenzene (83 mg, 0.38 mmol, 1.5 eq.) as iodoaryl derivative, Copper(I) thiophene-2-carboxylate (5 mg, 0.025 mmol, 0.1 eq.), *L*-Proline (7 mg, 0.05 mmol, 0.2 eq.) and potassium carbonate (163 mg, 0.50 mmol, 2.0 eq.) in dry DMSO (0.25 M). Crude product was purified by flash chromatography on silica gel using a gradient (PE: EtOAc) from (1: 0) to (7: 3) yielding to corresponding product **44** (68 mg, 83 %) as a white solid. Rf: 0.62 (PE/EtOAc: 80/20). MP: 100–102 °C. IR (ATR diamond, cm^{-1}) ν: 2917, 1558, 1519, 1351, 1193, 1106, 989, 834, 805, 778, 699, 608. ^{1}H NMR (400 MHz, Chloroform-d): δ 7.79 (d, J_{11-12} = 8.1 Hz, 2H, H-11), 7.50 (s, 1H, H-5), 7.42 (d, J_{16-17} = 7.8 Hz, 2H, H-16), 7.29 (d, J_{11-12} = 8.1 Hz, 2H, H-12), 7.24 (d, J_{16-17} = 7.8 Hz, 2H, H-17), 6.02 (ddt, J_{8-9} = 17.2, J_{7-8} = 9.9, J_{7-8} = 5.5 Hz, 1H, H-8), 5.28 – 5.13 (m, 3H, H-7 and H-9), 5.04 (d, J_{8-9} = 17.2 Hz, 1H, H-9), 2.40 (s, 3H, H-14), 2.39 (s, 3H, H-19). ^{13}C NMR (101 MHz, Chloroform-d): δ 150.0 (C_{3a}), 136.9 (C_q), 135.9 (C_q), 134.8 (C_q), 132.5 (C_8), 130.3 (2 x C_{12}), 130.0 (C_q), 129.7 (2 x C_{17}), 127.4 (2 x C_{16}), 127.3 (C_q), 124.0 (C_5), 118.6 (C_9), 118.5 (2 x C_{11}), 109.0 (C_6), 52.2 (C_7), 21.3 (CH_3), 21.0 (CH_3). HRMS (EI^+) m/z calculated for $C_{21}H_{21}N_4$ $[M+H]^+$: 329.1761, found: 329.1755

4,6-Di-*p*-tolyl-1,4-dihydrpyrrolo[2,3-*d*][1,2,3]triazole (45)

The reaction was carried out as described in general procedure **E** using 2-allyl-4-benzyl-6-(*p*-tolyl)-2,4-dihydropyrrolo[2,3-*d*][1,2,3]triazole **44** (68 mg, 0.21 mmol, 1.0 eq.) as allyl-triazole bicycle derivative, [1,2-Bis(diphenylphosphino)ethane]dichloronickel(II) (2.3 mg, 0.004 mmol, 0.02 eq.) and *Tert*-Butylmagnesium chloride, 2.0 M in Et_2O (0.22 mL, 0.43 mmol, 2.0 eq.) in dry toluene (0.15 M). Crude product was purified by flash chromatography on silica gel using a gradient of (PE: EtOAc) from (1: 0) to (1: 1) yielding to corresponding product **45** (40 mg, 67 %) as a white solid. Rf: 0.39 (PE/EtOAc: 50/50). MP: 241–243 °C. IR (ATR diamond, cm^{-1}) ν: 2917, 1570, 1521, 1244, 1181, 1163, 995, 873, 813, 798, 634. ^{1}H NMR (400 MHz, Acetone-d_6): δ 14.12 (br, 1H, N_1-H), 8.44 (s, 1H, H-5), 7.96 – 7.88 (m, 4H, H-8 and H-13), 7.34 (d, J_{8-9} = 8.4 Hz, 2H, H-9), 7.25 (d, J_{13-14} = 7.9 Hz, 2H, H-14), 2.36 (s, 3H, H-11), 2.35 (s, 3H, H-16). ^{13}C DEPT 135 NMR (101 MHz, Acetone-d_6): δ 130.9 (2 x C_9), 130.2 (2 x C_{14}), 126.1 (2 x CH_{Ar}), 125.8 (C_5), 118.2 (2 x CH_{Ar}), 21.2 (CH_3), 20.8 (CH_3). HRMS (EI^+) m/z calculated for $C_{18}H_{17}N_4$ $[M+H]^+$: 289.1448, found: 289.1453.

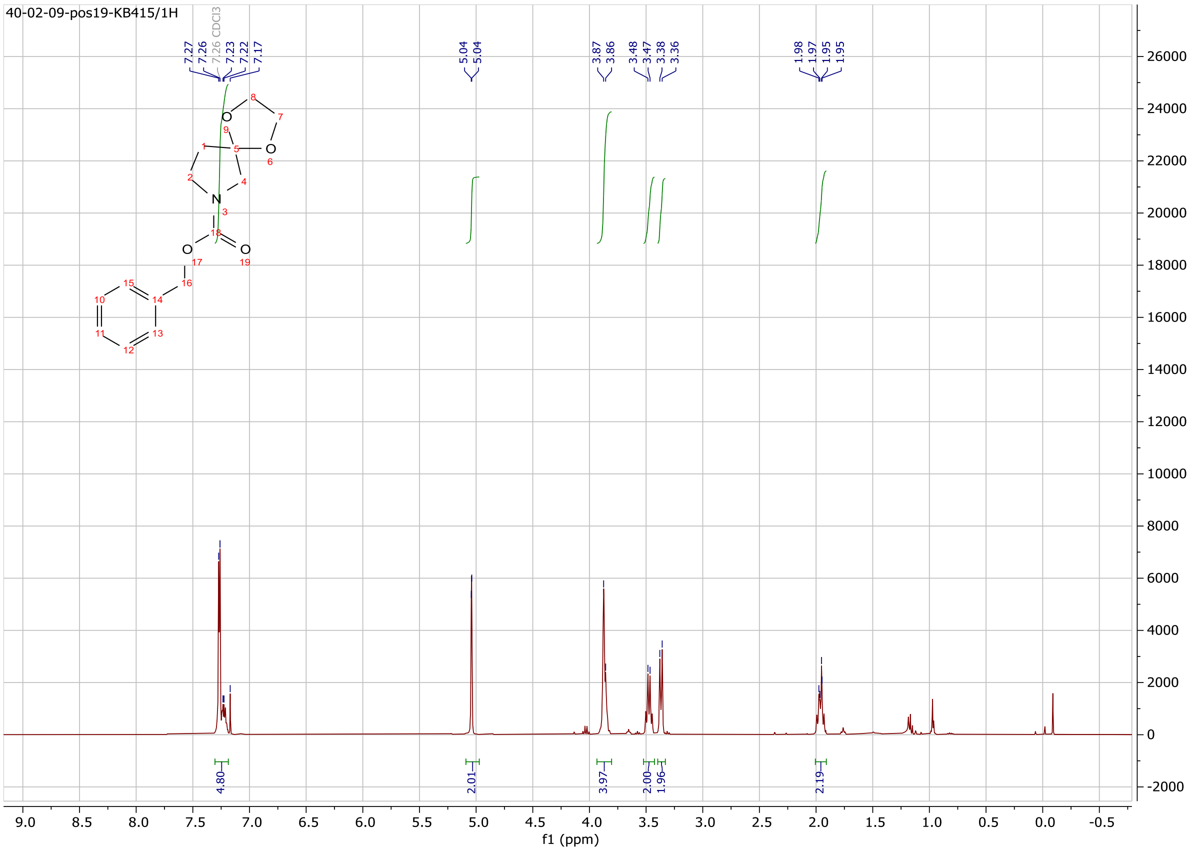
40-02-09-pos19-KB415/1H
7.27
7.26
7.26 CDCl3
7.23
7.22
7.17
5.04
5.04
3.87
3.86
3.48
3.47
3.38
3.36
1.98
1.97
1.95
1.95
4.80
2.01
3.97
2.00
1.96
2.19
f1 (ppm)

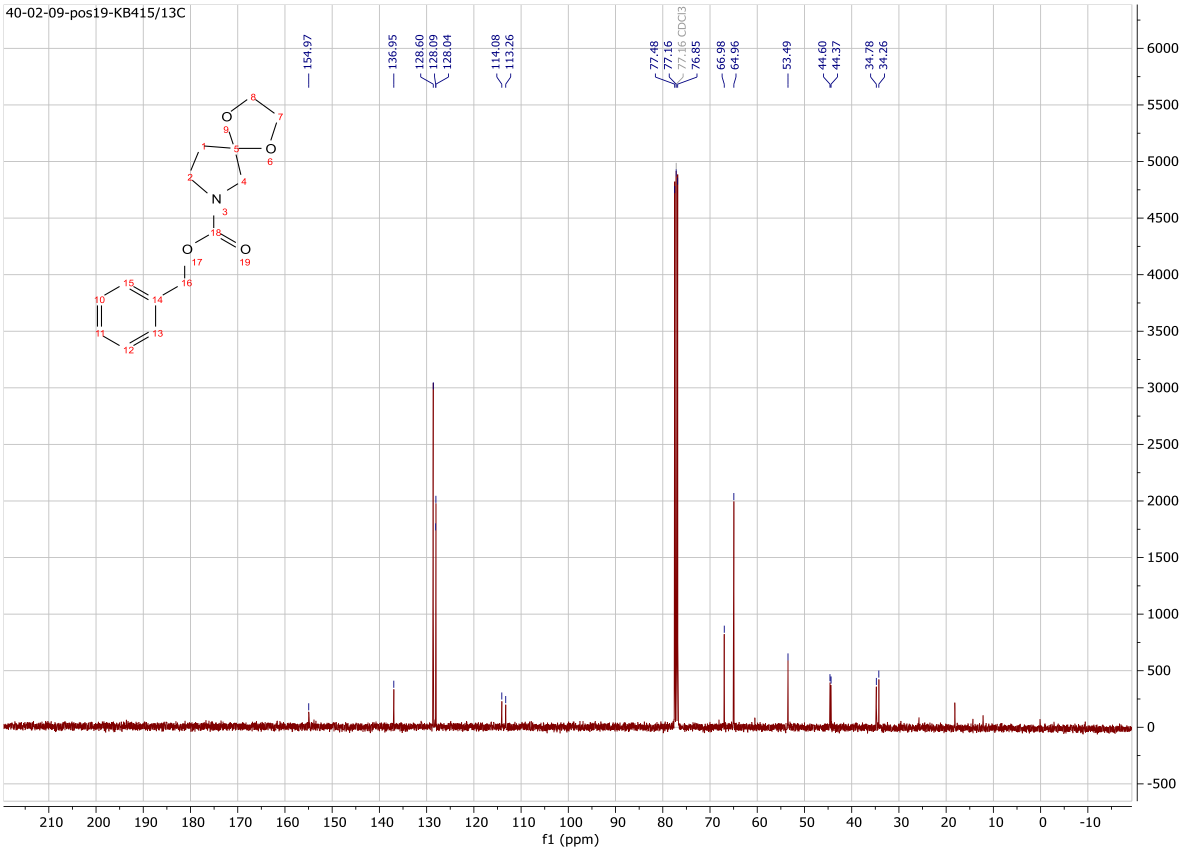
40-02-09-pos19-KB415/13C
154.97
136.95
128.60
128.09
128.04
114.08
113.26
77.48
77.16
77.16 CDCl3
76.85
66.98
64.96
53.49
44.60
44.37
34.78
34.26
f1 (ppm)

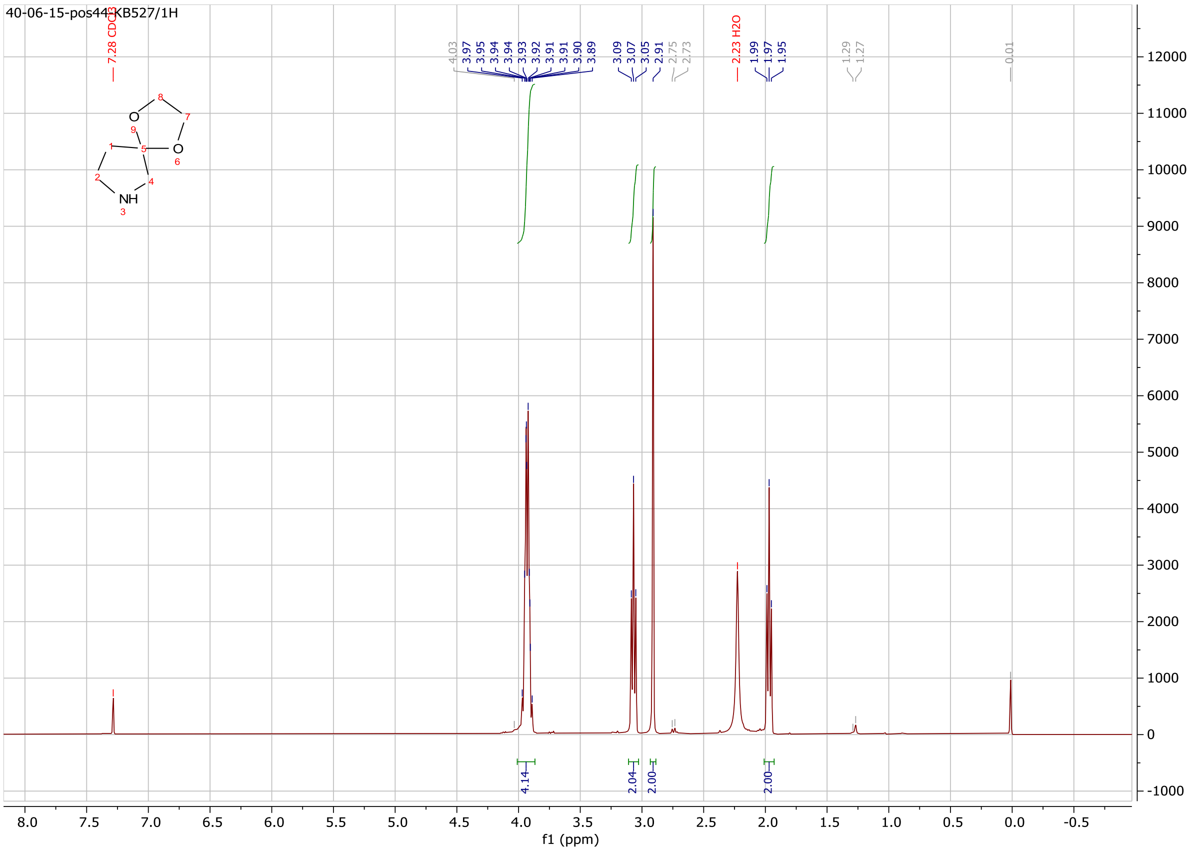
40-06-15-pos44-KB527/1H
7.28 CDCl3
2.23 H2O
4.14
2.04
2.00
2.00
f1 (ppm)

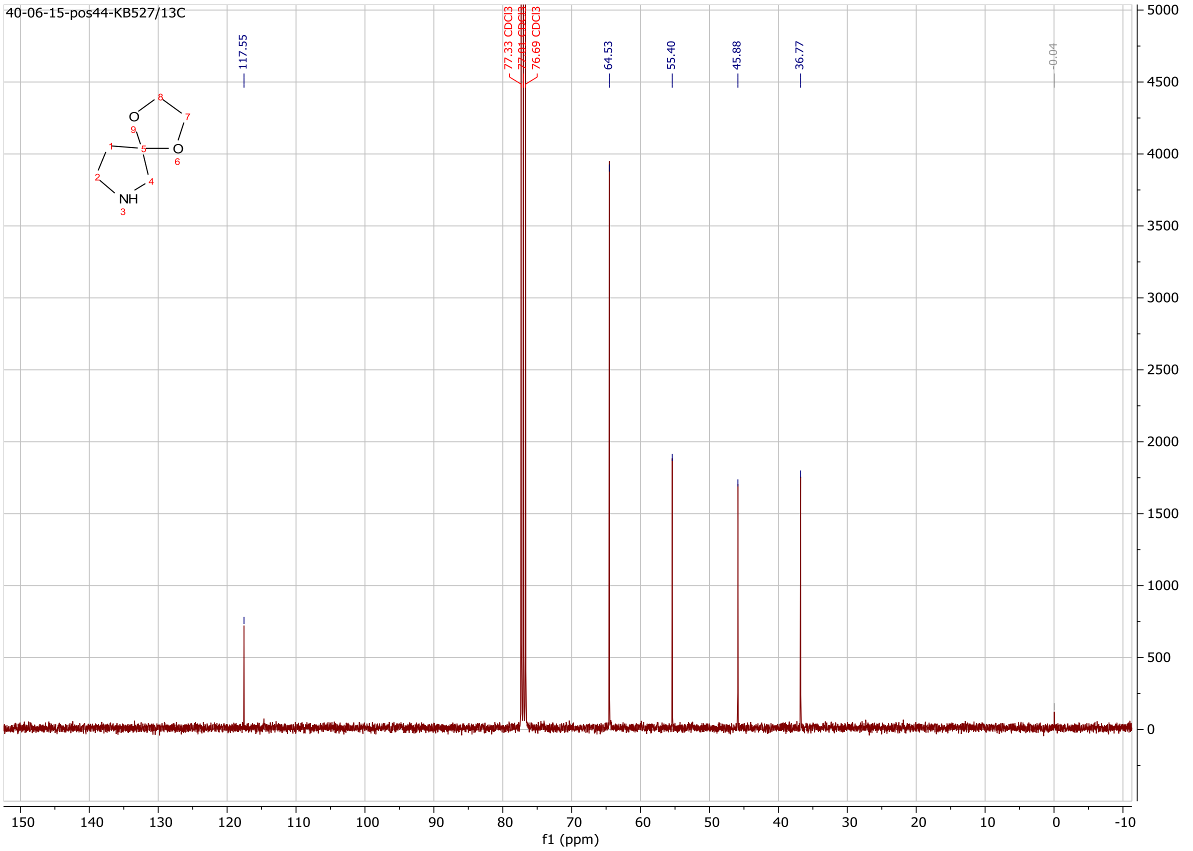
40-06-15-pos44-KB527/13C
117.55
77.33 CDCl3
76.69 CDCl3
64.53
55.40
45.88
36.77
f1 (ppm)

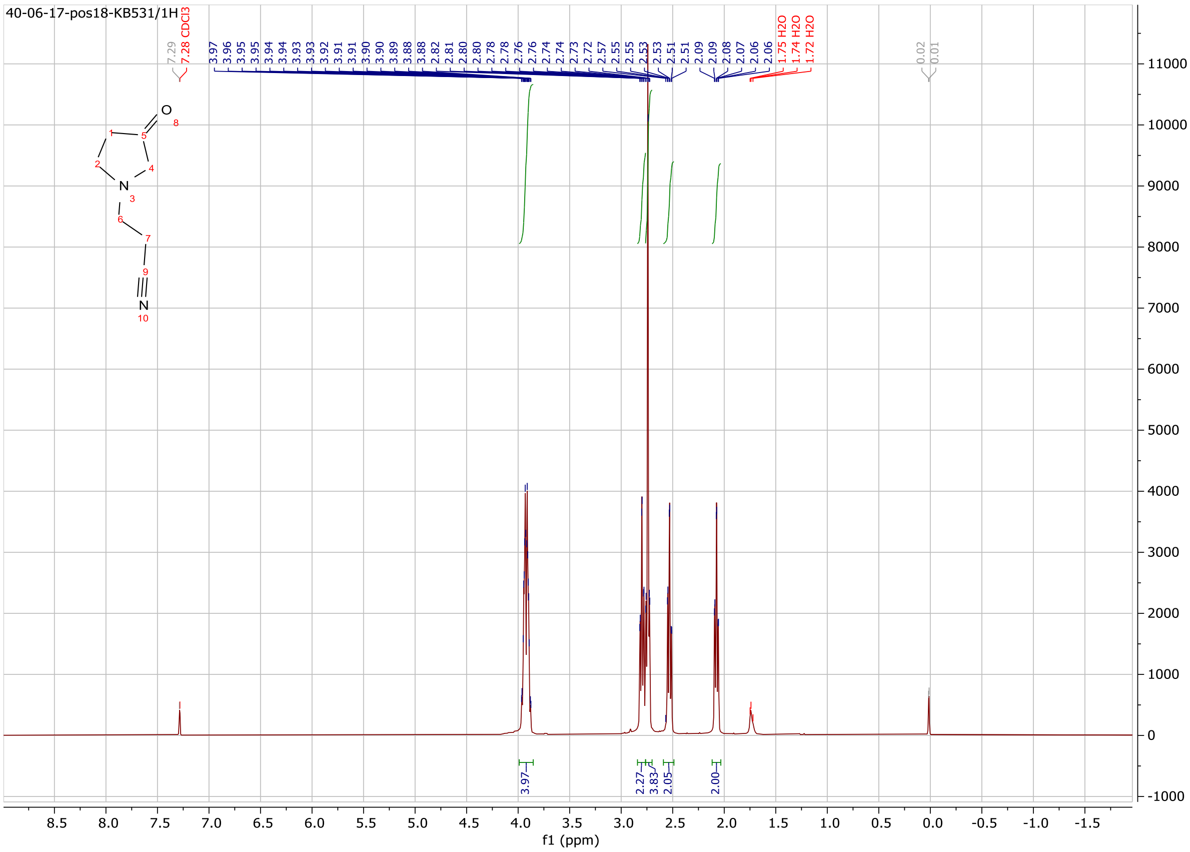
40-06-17-pos18-KB531/1H
7.29
7.28 CDCl3
3.97 3.96 3.95 3.95 3.94 3.94 3.93 3.93 3.92 3.91 3.91 3.90 3.90 3.89 3.88 3.88 2.82 2.81 2.80 2.80 2.78 2.78 2.76 2.76 2.74 2.74 2.73 2.72 2.57 2.55 2.55 2.53 2.53 2.51 2.51 2.09 2.09 2.08 2.07 2.06 2.06
1.75 H2O
1.74 H2O
1.72 H2O
0.02
0.01
O
N
N
3.97
2.27
3.83
2.05
2.00
f1 (ppm)

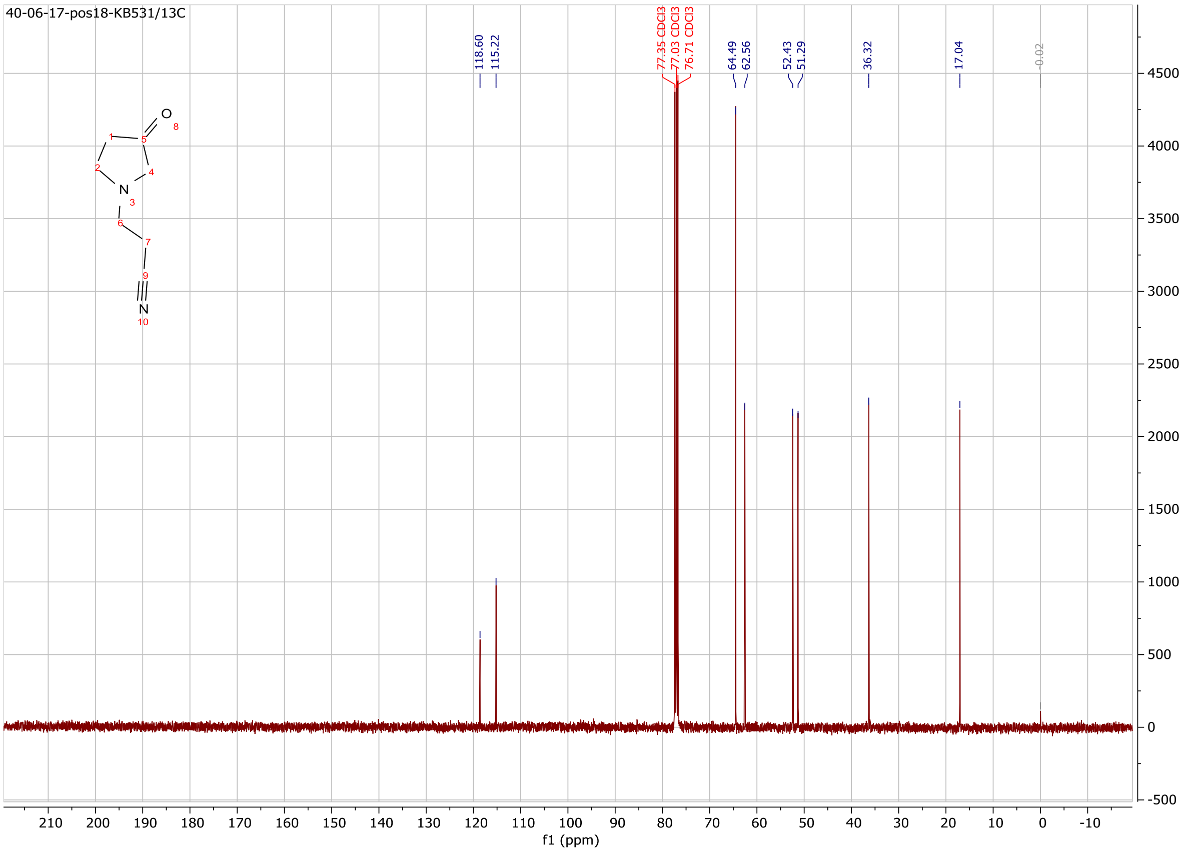
40-06-17-pos18-KB531/13C
118.60
115.22
77.35 CDCl3
77.03 CDCl3
76.71 CDCl3
64.49
62.56
52.43
51.29
36.32
17.04
0.02
O
N
N
f1 (ppm)

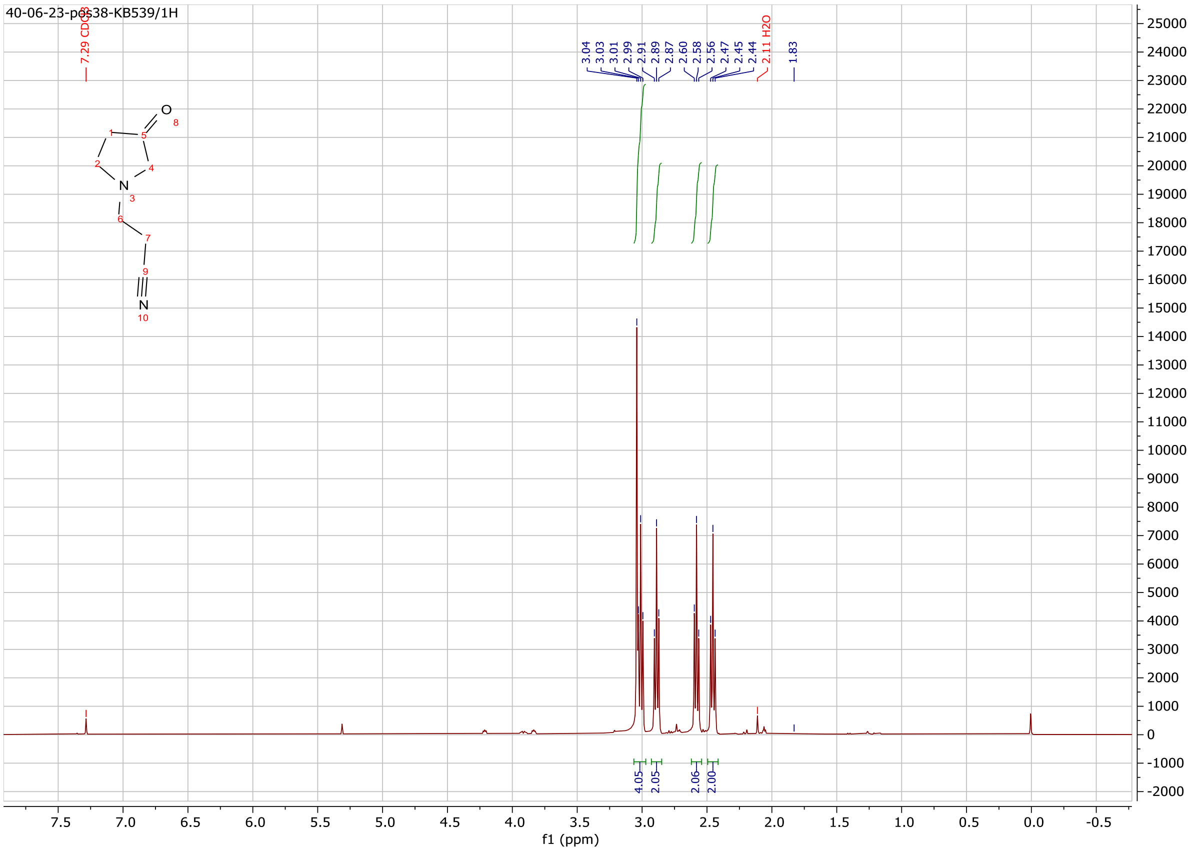
40-06-23-pos38-KB539/1H
7.29 CDCl3
3.04
3.03
3.01
2.99
2.91
2.89
2.87
2.60
2.58
2.56
2.47
2.45
2.44
2.11 H2O
1.83
4.05
2.05
2.06
2.00
f1 (ppm)

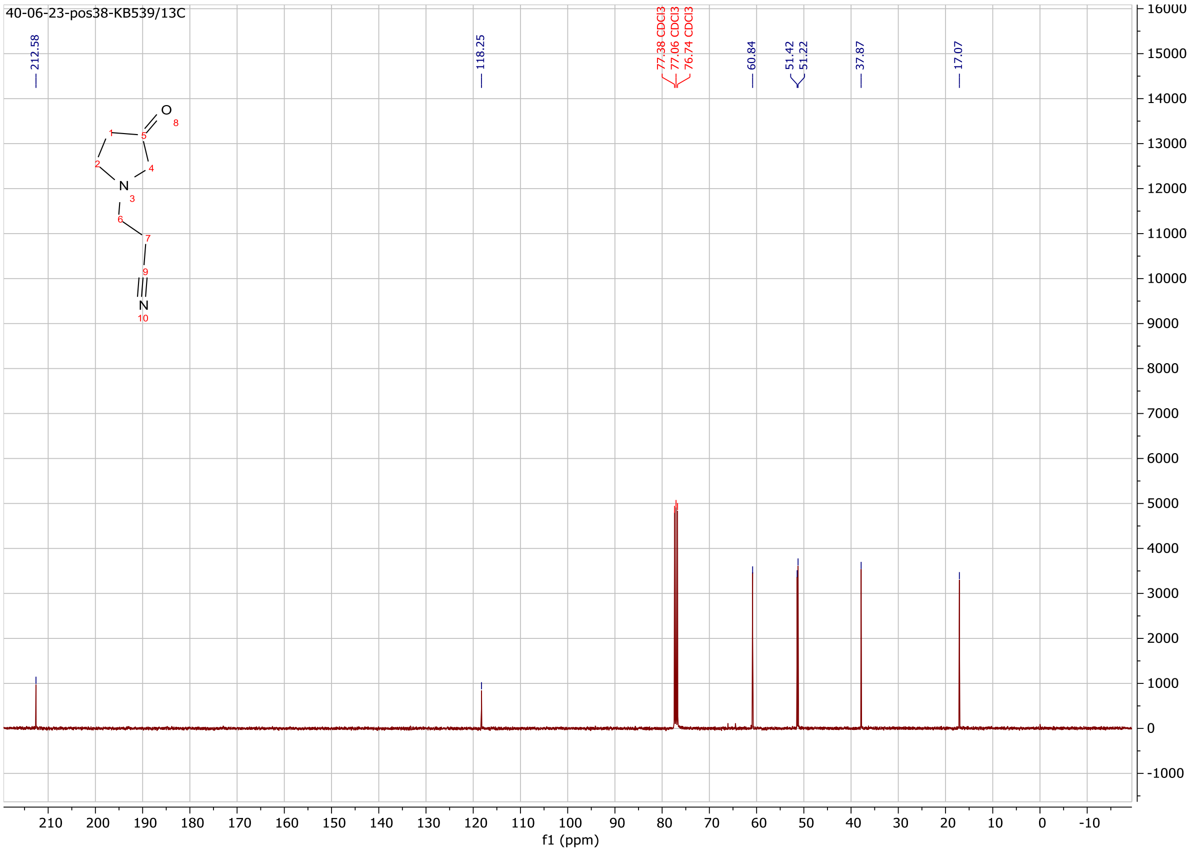
40-06-23-pos38-KB539/13C
212.58
118.25
77.38 CDCl3
77.06 CDCl3
76.74 CDCl3
60.84
51.42
51.22
37.87
17.07
f1 (ppm)

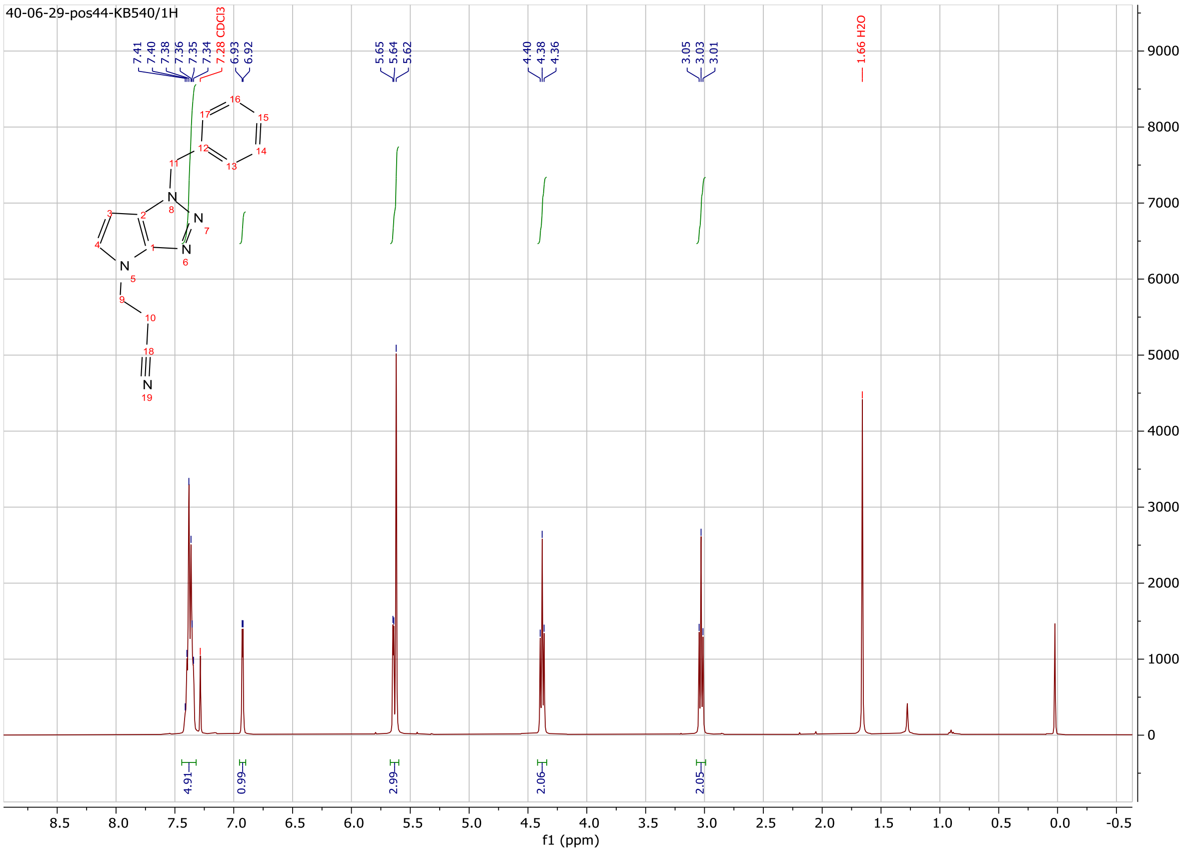
40-06-29-pos44-KB540/1H
7.41
7.40
7.38
7.36
7.35
7.34
7.28 CDCl3
6.93
6.92
5.65
5.64
5.62
4.40
4.38
4.36
3.05
3.03
3.01
1.66 H2O
4.91
0.99
2.99
2.06
2.05
f1 (ppm)

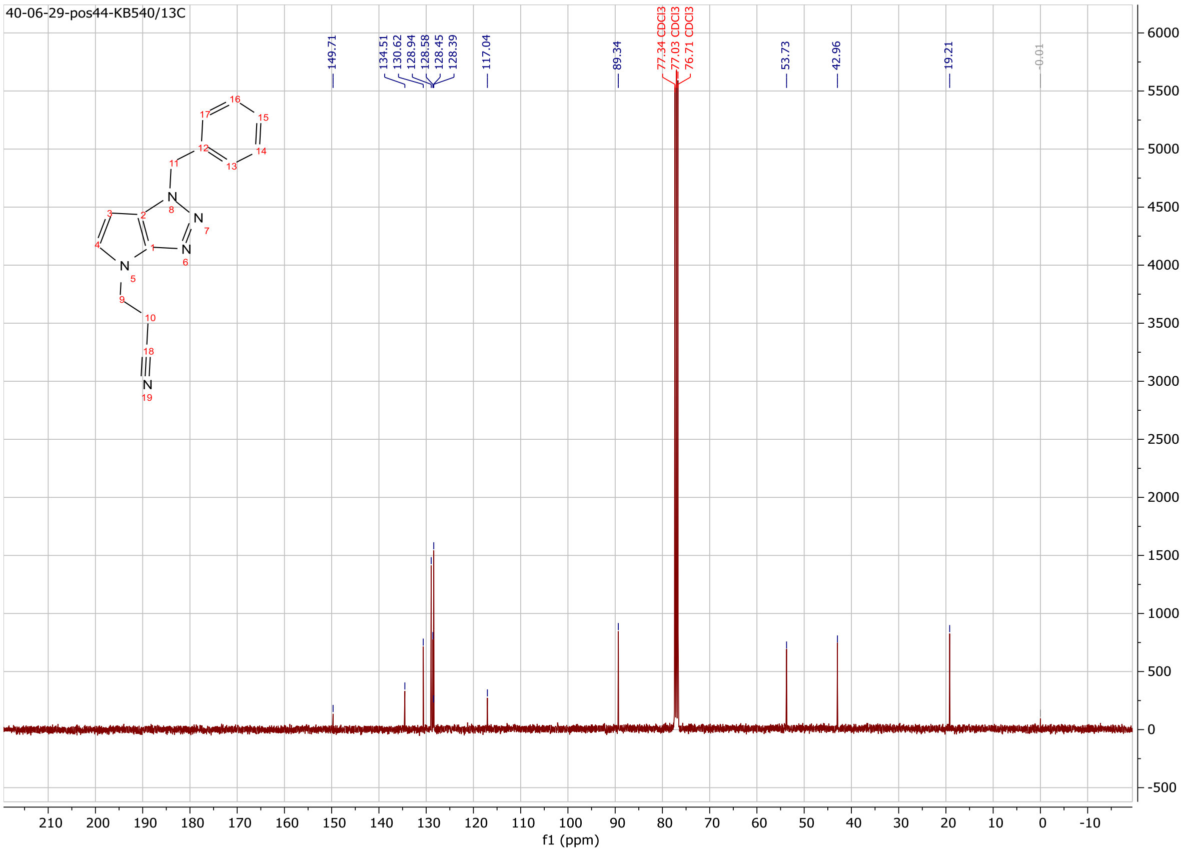
40-06-29-pos44-KB540/13C
149.71
134.51
130.62
128.94
128.58
128.45
128.39
117.04
89.34
77.34 CDCl3
77.03 CDCl3
76.71 CDCl3
53.73
42.96
19.21
-0.01
f1 (ppm)

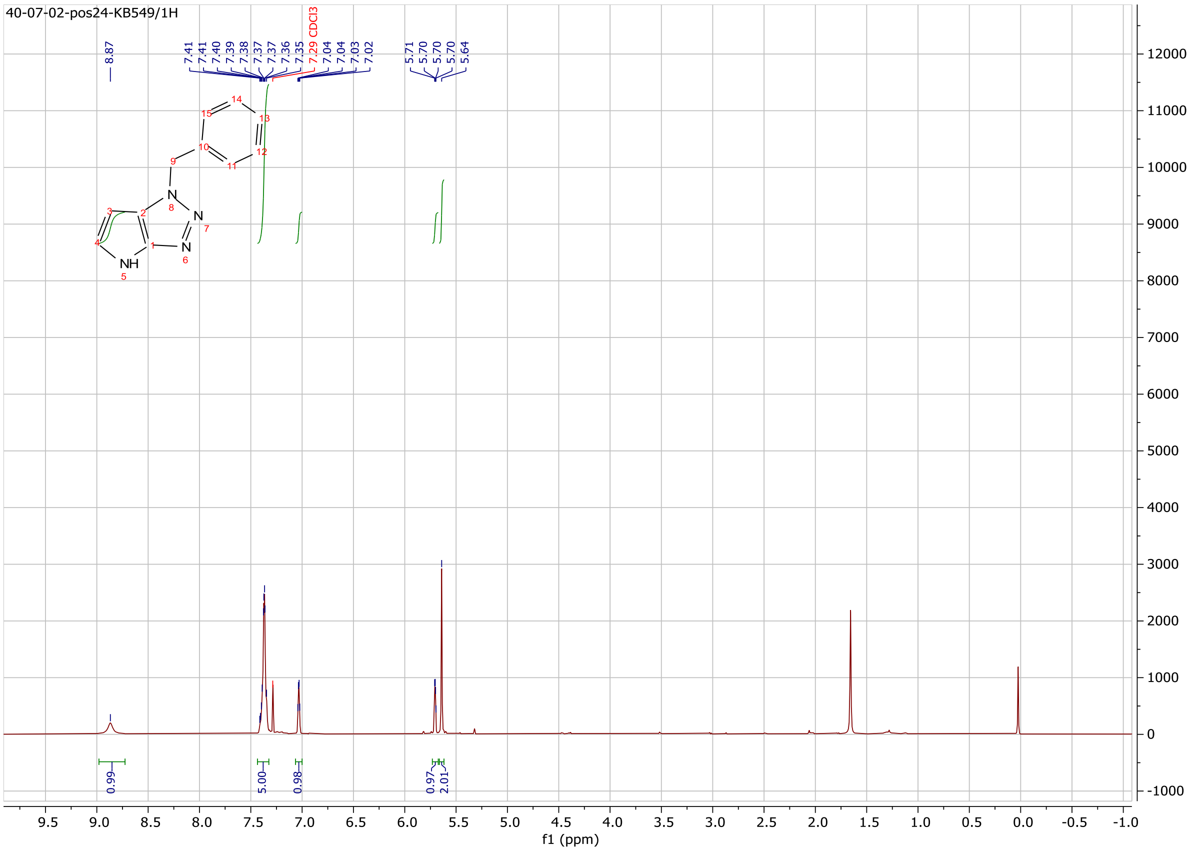
40-07-02-pos24-KB549/1H
8.87
7.41 7.41 7.40 7.39 7.38 7.37 7.37 7.36 7.35 7.29 CDCl3 7.04 7.04 7.03 7.02
5.71 5.70 5.70 5.70 5.64
0.99
5.00
0.98
0.97
2.01
f1 (ppm)

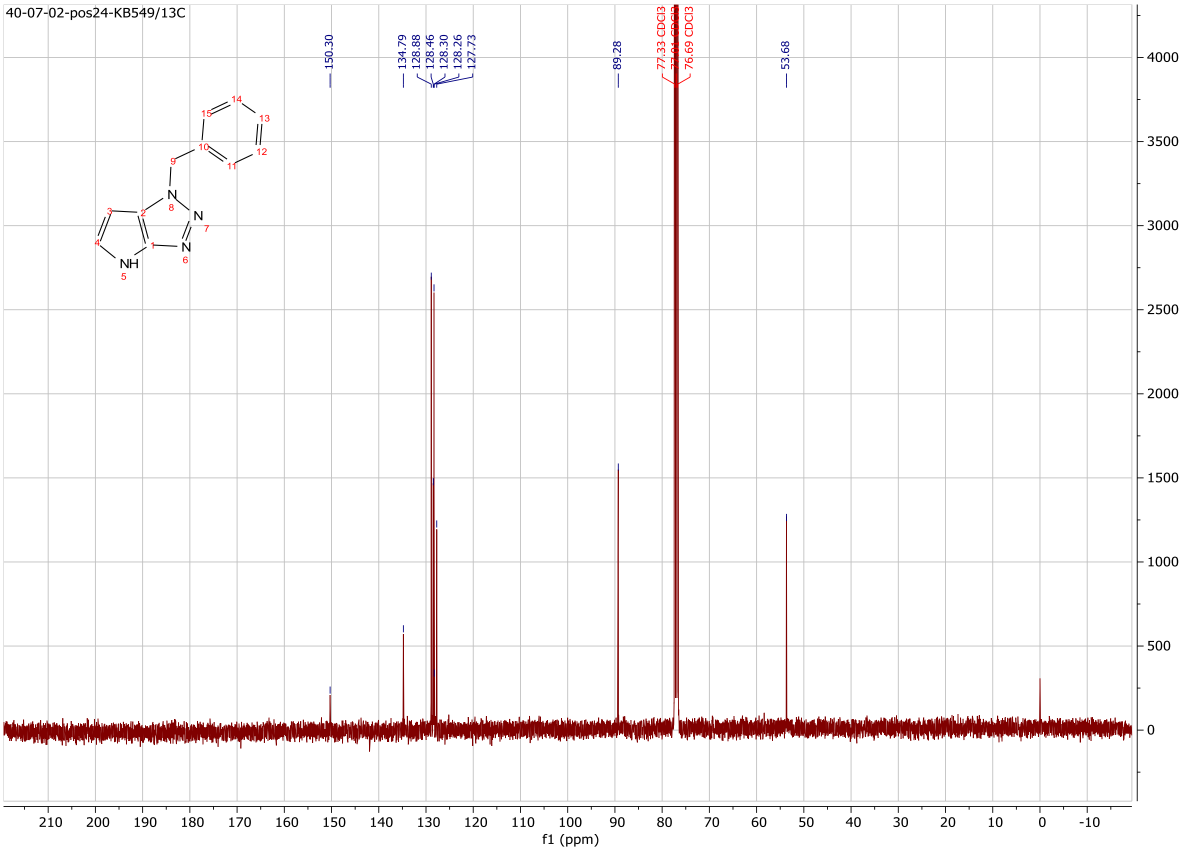
40-07-02-pos24-KB549/13C
150.30
134.79 128.88 128.46 128.30 128.26 127.73
89.28
77.33 CDCl3
76.69 CDCl3
53.68
f1 (ppm)

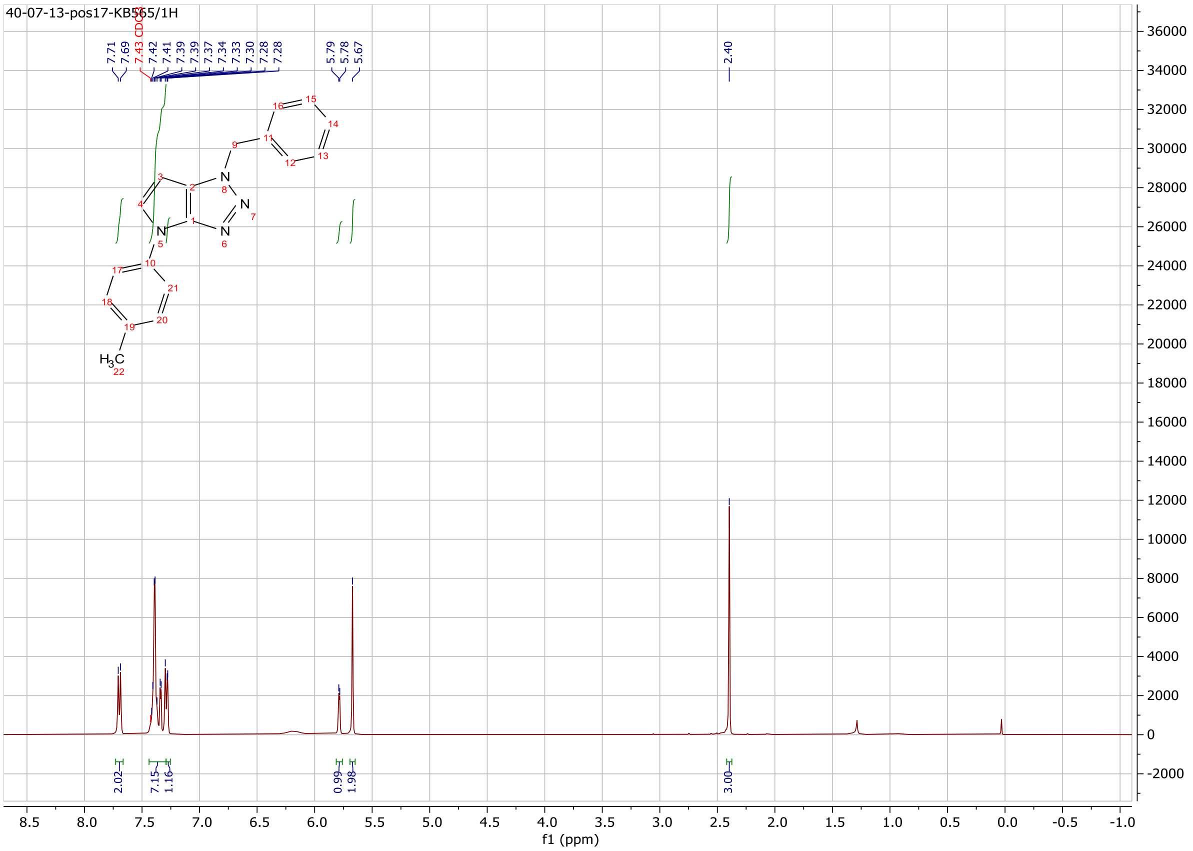
40-07-13-pos17-KB565/1H
f1 (ppm)

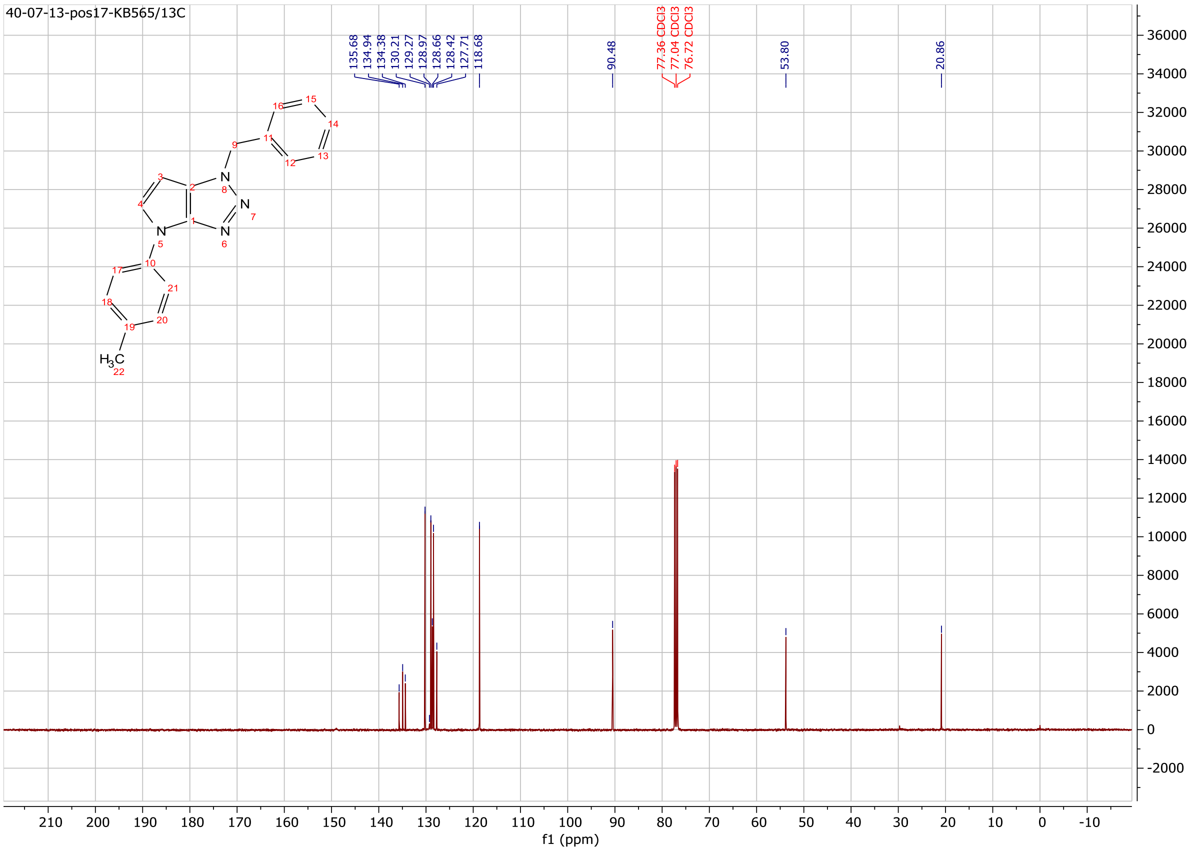
40-07-13-pos17-KB565/13C
f1 (ppm)

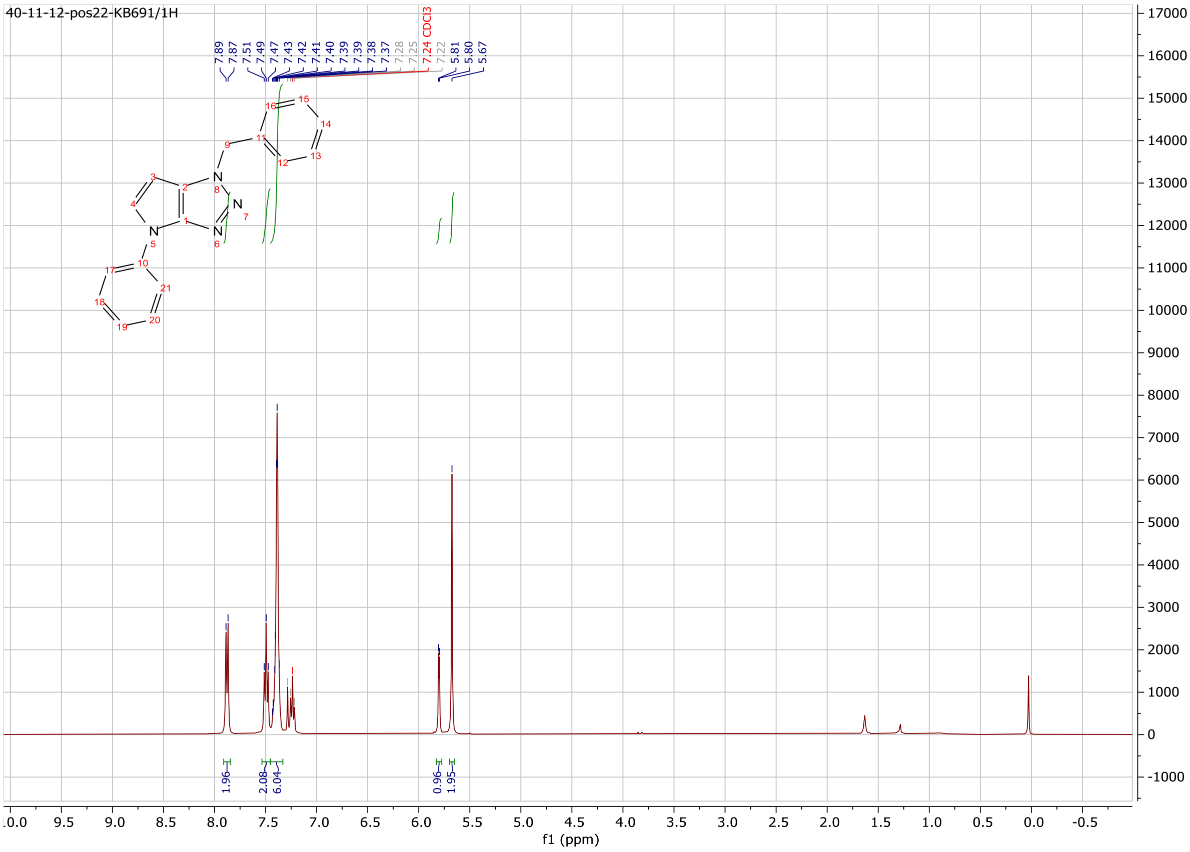
40-11-12-pos22-KB691/1H
7.89
7.87
7.51
7.49
7.47
7.43
7.42
7.41
7.40
7.39
7.39
7.38
7.37
7.28
7.25
7.24 CDCl3
7.22
5.81
5.80
5.67
1.96
2.08
6.04
0.96
1.95
f1 (ppm)

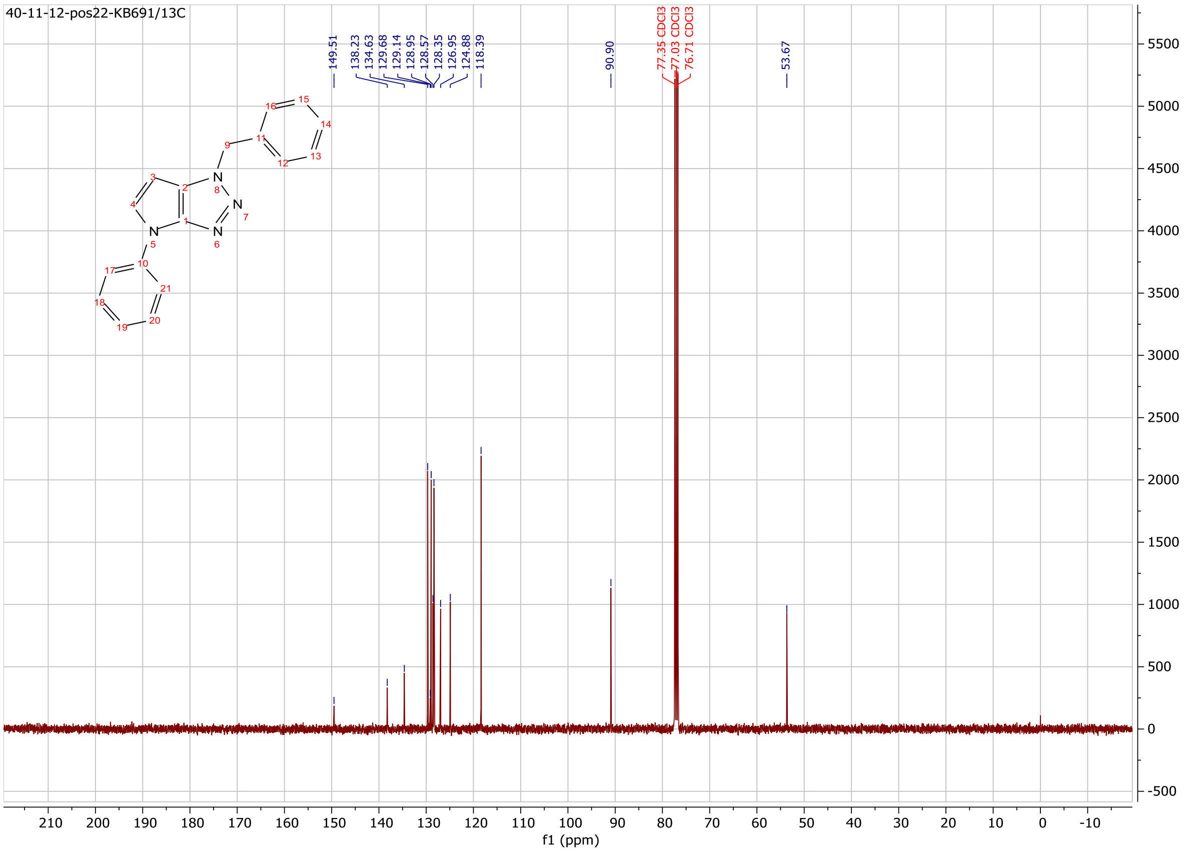
40-11-12-pos22-KB691/13C
149.51
138.23
134.63
129.68
129.14
128.95
128.57
128.35
126.95
124.88
118.39
90.90
77.35 CDCl3
77.03 CDCl3
76.71 CDCl3
53.67
f1 (ppm)

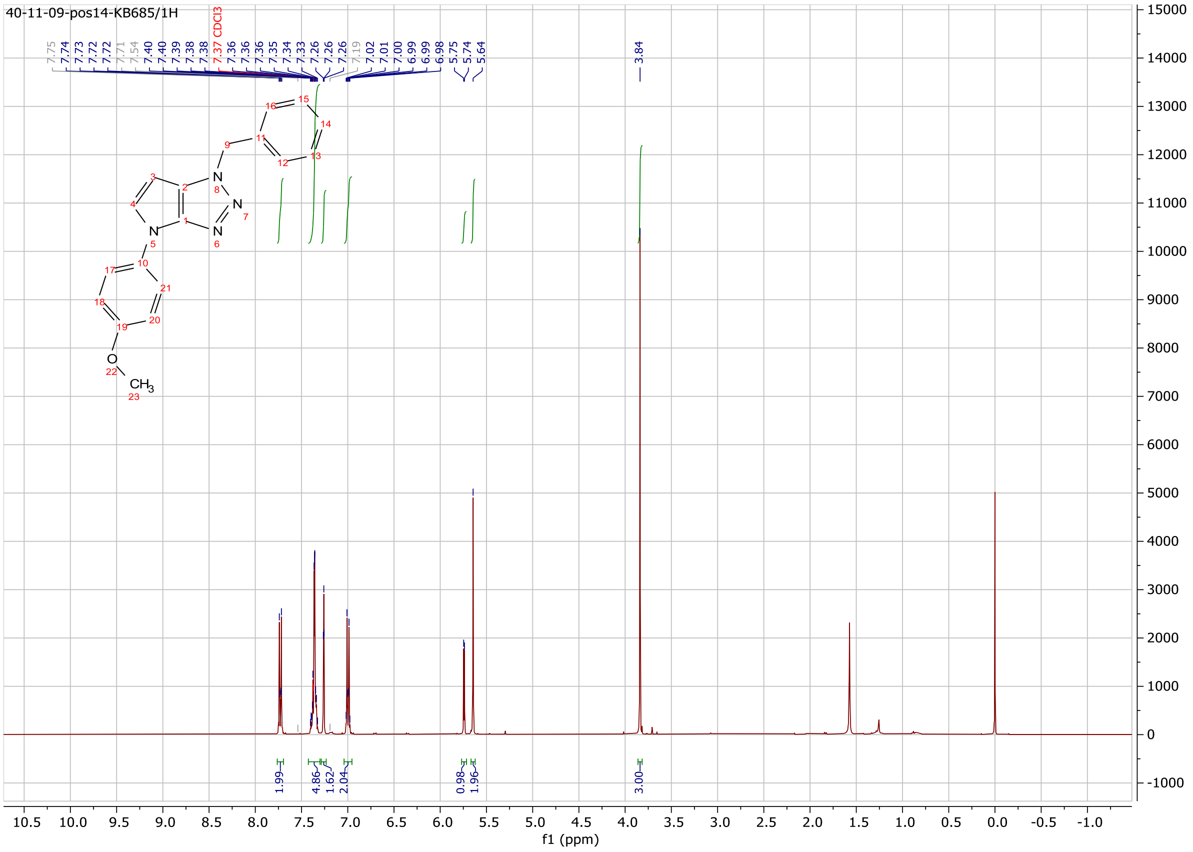
40-11-09-pos14-KB685/1H
f1 (ppm)

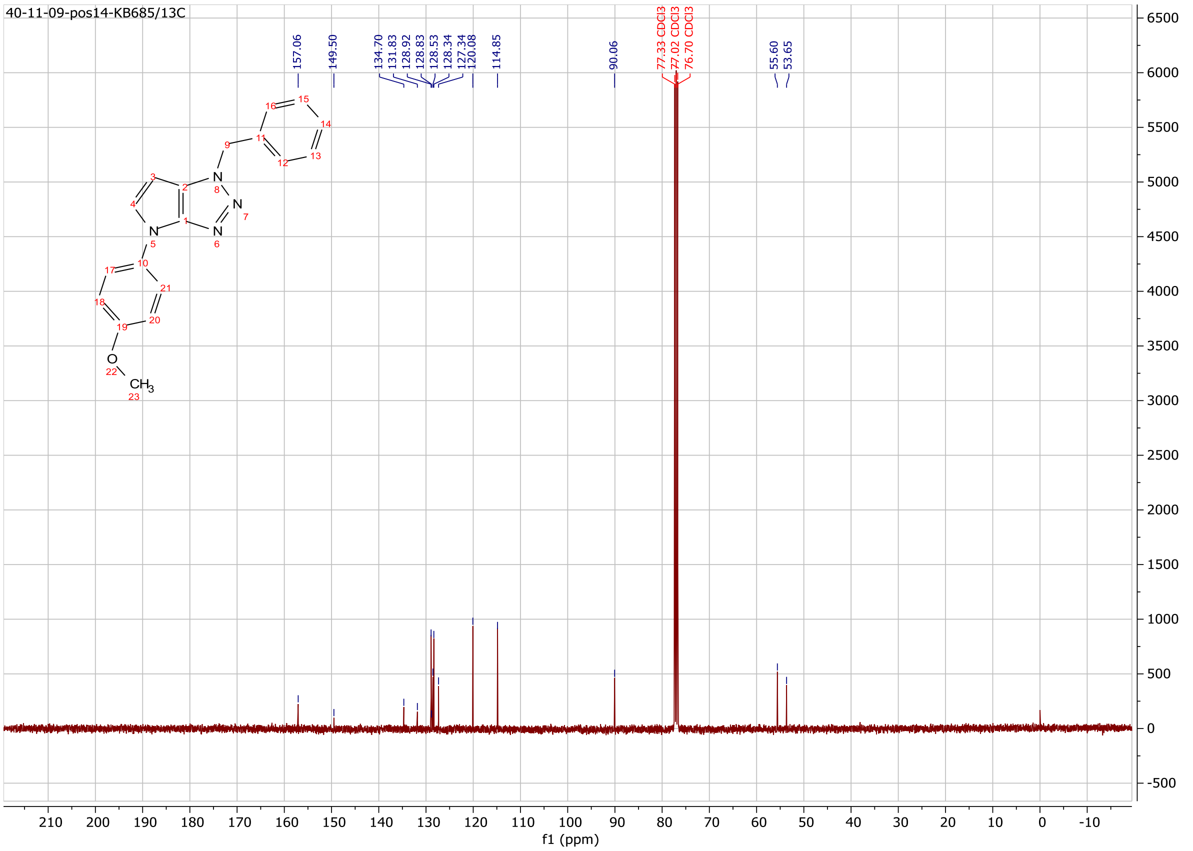
40-11-09-pos14-KB685/13C
f1 (ppm)

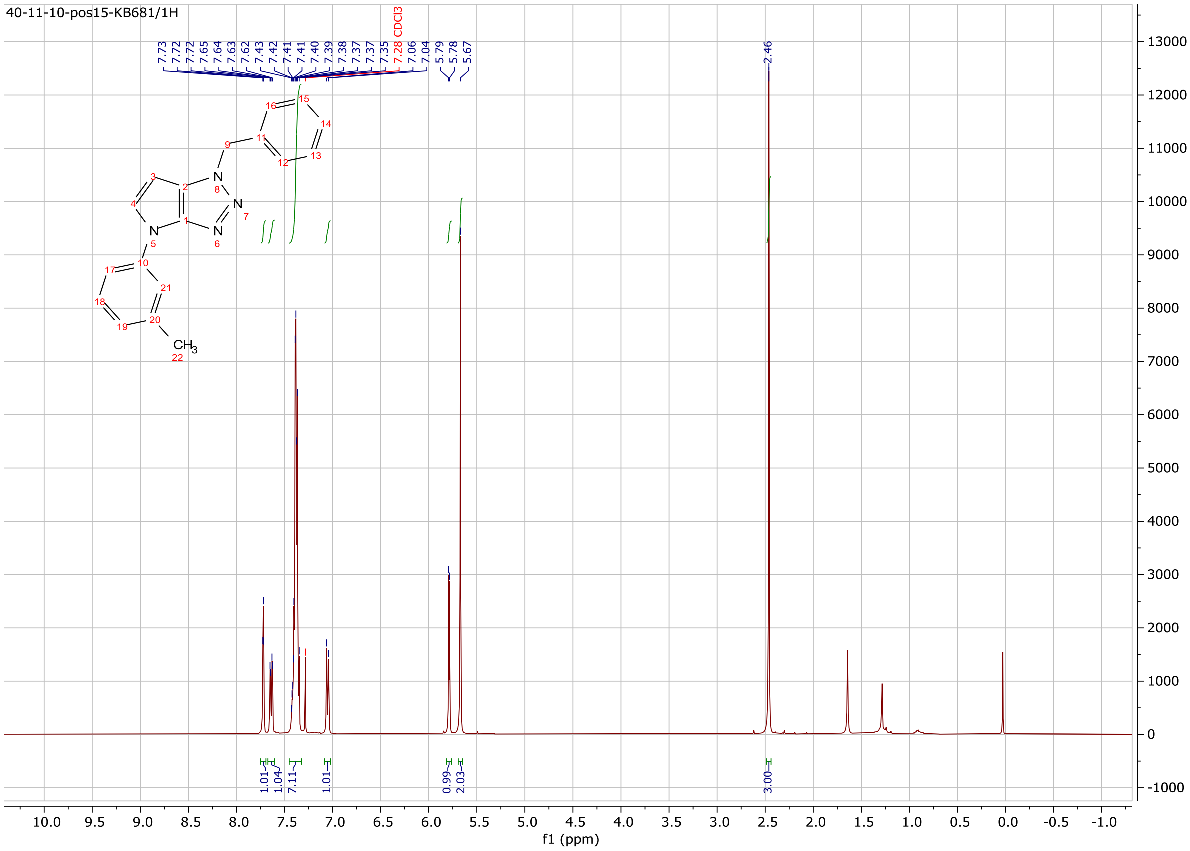
40-11-10-pos15-KB681/1H
7.73
7.72
7.72
7.65
7.64
7.63
7.62
7.43
7.42
7.41
7.41
7.40
7.39
7.38
7.37
7.37
7.35
7.28 CDCl3
7.06
7.04
5.79
5.78
5.67
2.46
1.01
1.04
7.11
1.01
0.99
2.03
3.00
CH3
f1 (ppm)

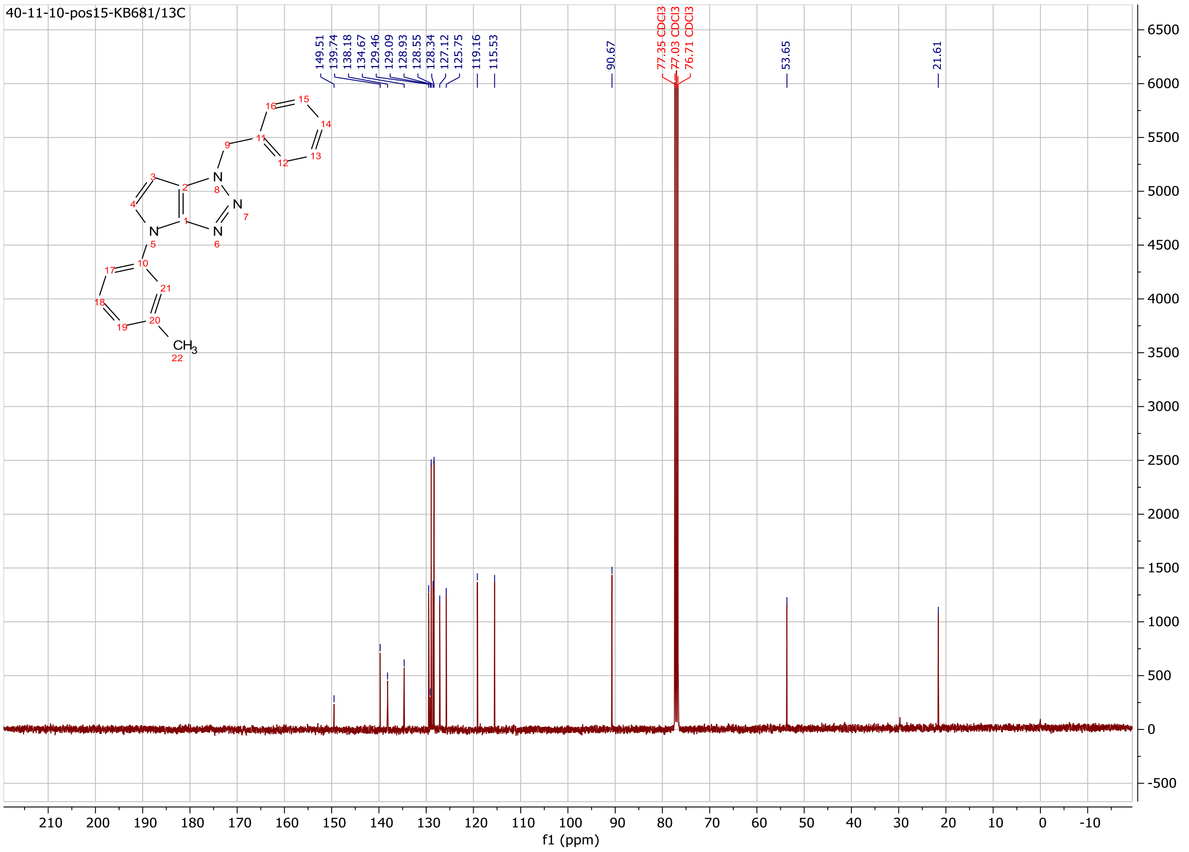
40-11-10-pos15-KB681/13C
149.51
139.74
138.18
134.67
129.46
129.09
128.93
128.55
128.34
127.12
125.75
119.16
115.53
90.67
77.35 CDCl3
77.03 CDCl3
76.71 CDCl3
53.65
21.61
CH3
f1 (ppm)

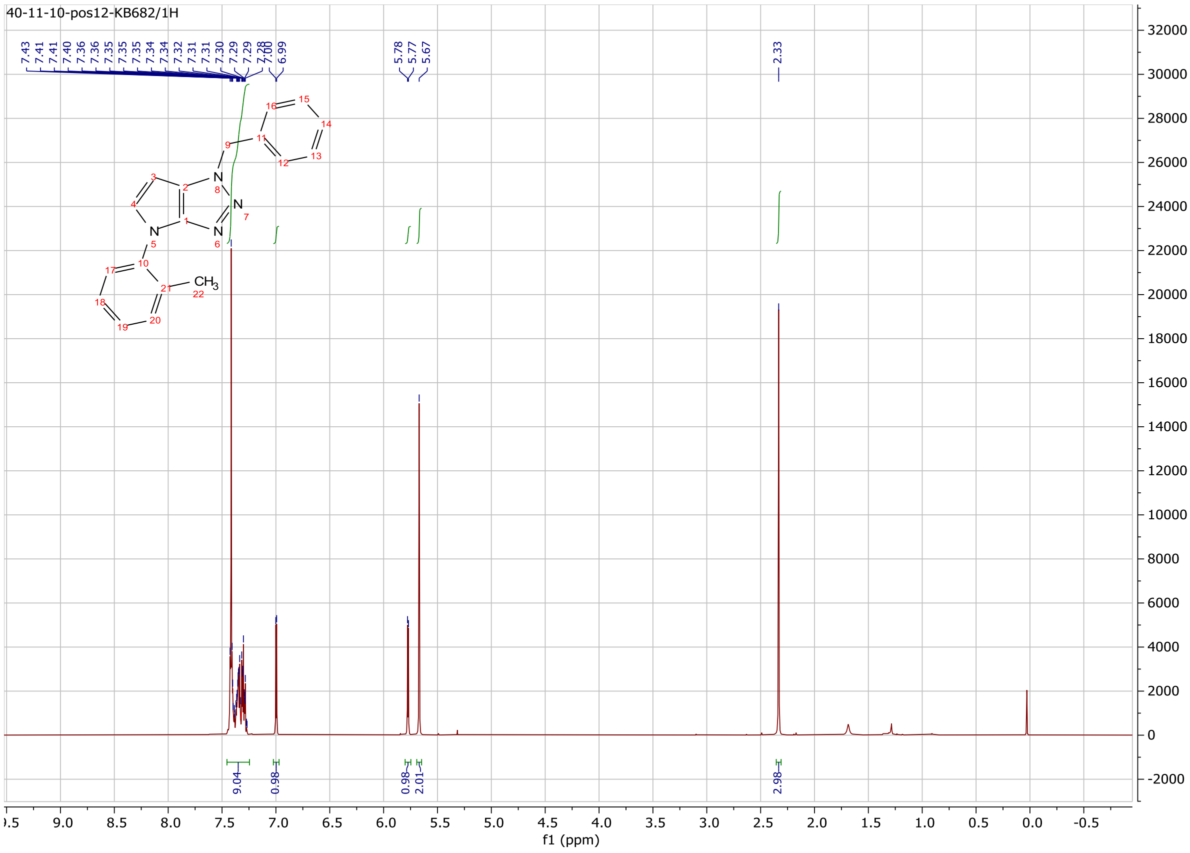
40-11-10-pos12-KB682/1H
f1 (ppm)

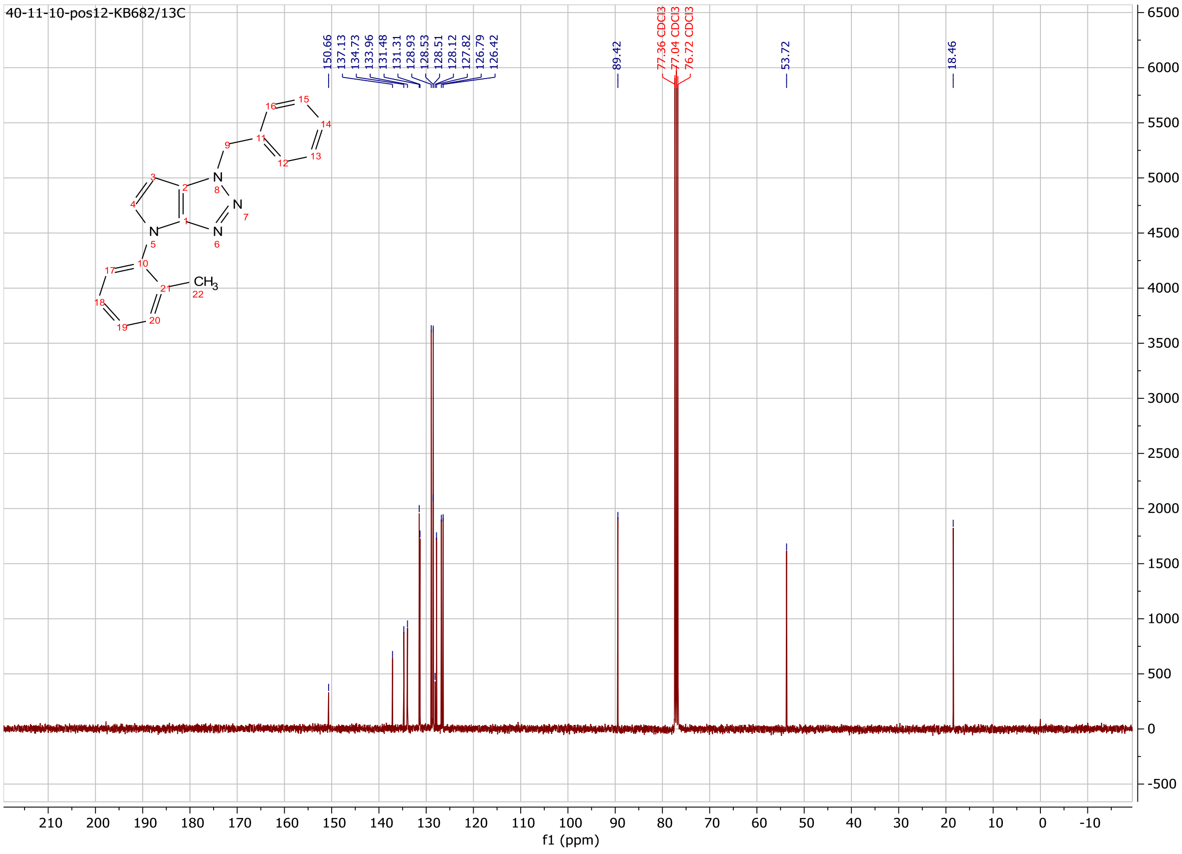
40-11-10-pos12-KB682/13C
f1 (ppm)

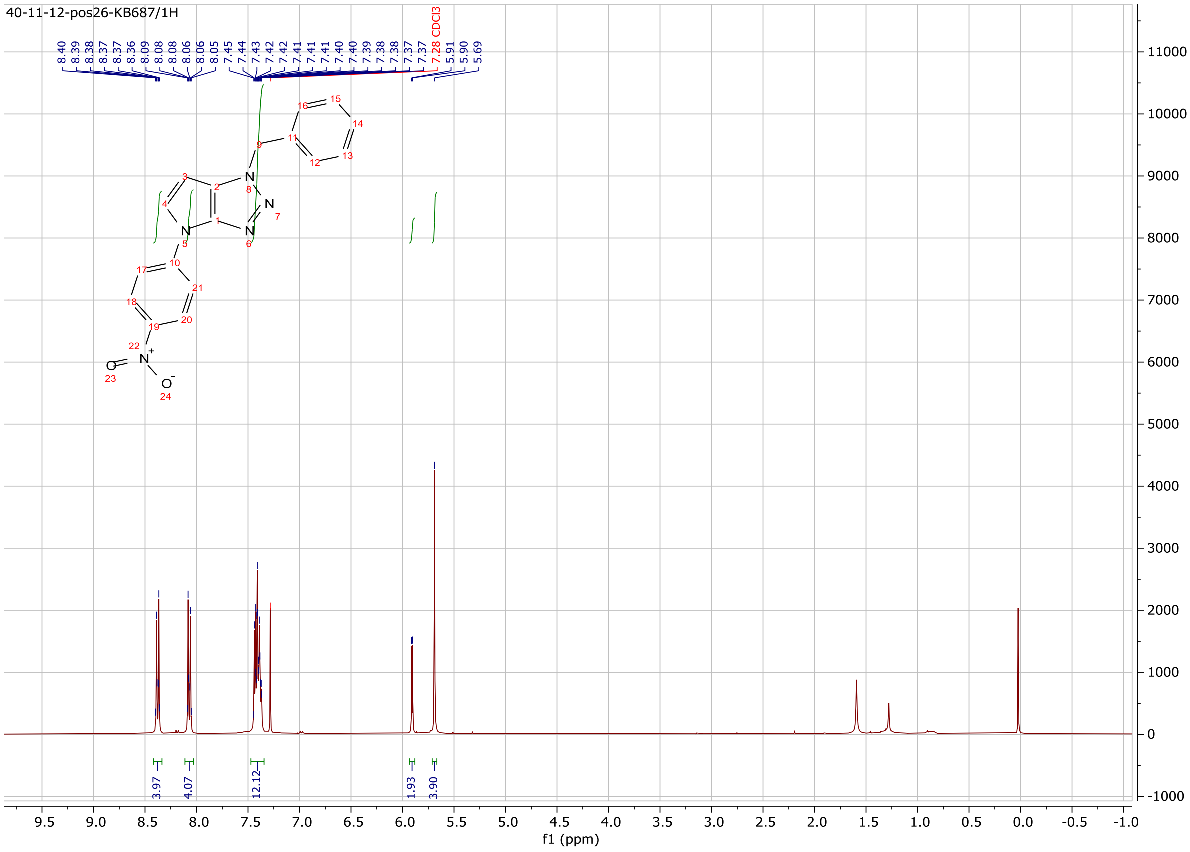
40-11-12-pos26-KB687/1H
8.40 8.39 8.38 8.37 8.37 8.36 8.09 8.08 8.08 8.06 8.06 8.05 7.45 7.44 7.43 7.42 7.42 7.41 7.41 7.41 7.40 7.40 7.39 7.38 7.38 7.37 7.37 7.28 CDCl3 5.91 5.90 5.69
3.97 4.07 12.12 1.93 3.90
f1 (ppm)

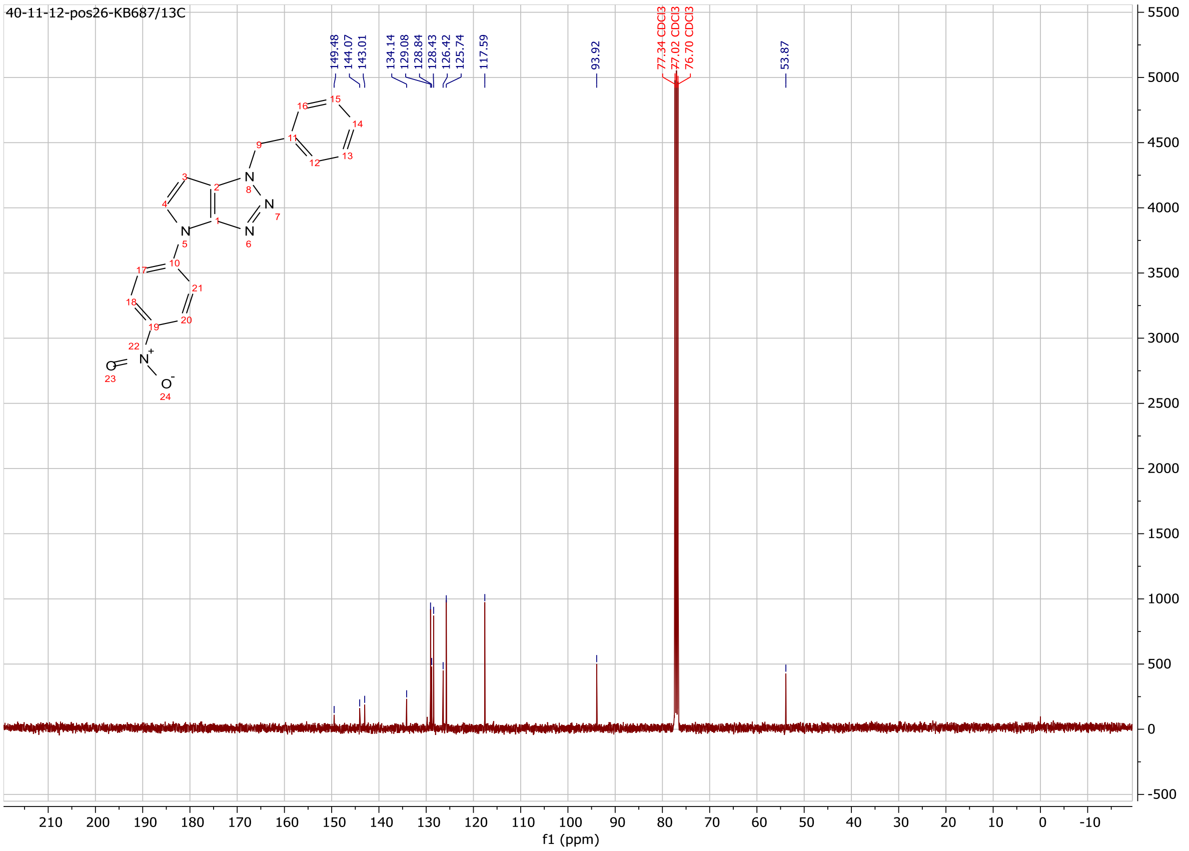
40-11-12-pos26-KB687/13C
149.48 144.07 143.01 134.14 129.08 128.84 128.43 126.42 125.74 117.59 93.92 77.34 CDCl3 77.02 CDCl3 76.70 CDCl3 53.87
f1 (ppm)

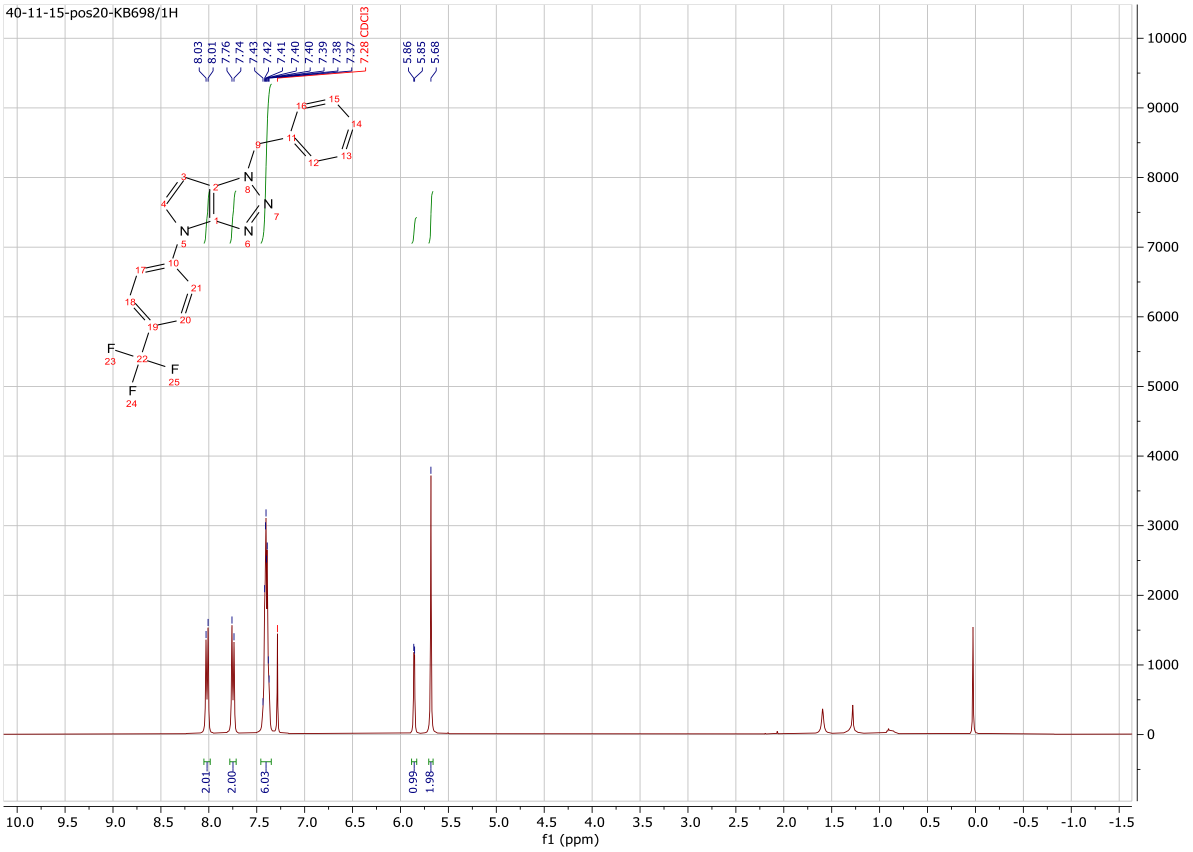
40-11-15-pos20-KB698/1H
8.03
8.01
7.76
7.74
7.43
7.42
7.41
7.40
7.39
7.38
7.37
7.28 CDCl3
5.86
5.85
5.68
2.01
2.00
6.03
0.99
1.98
f1 (ppm)

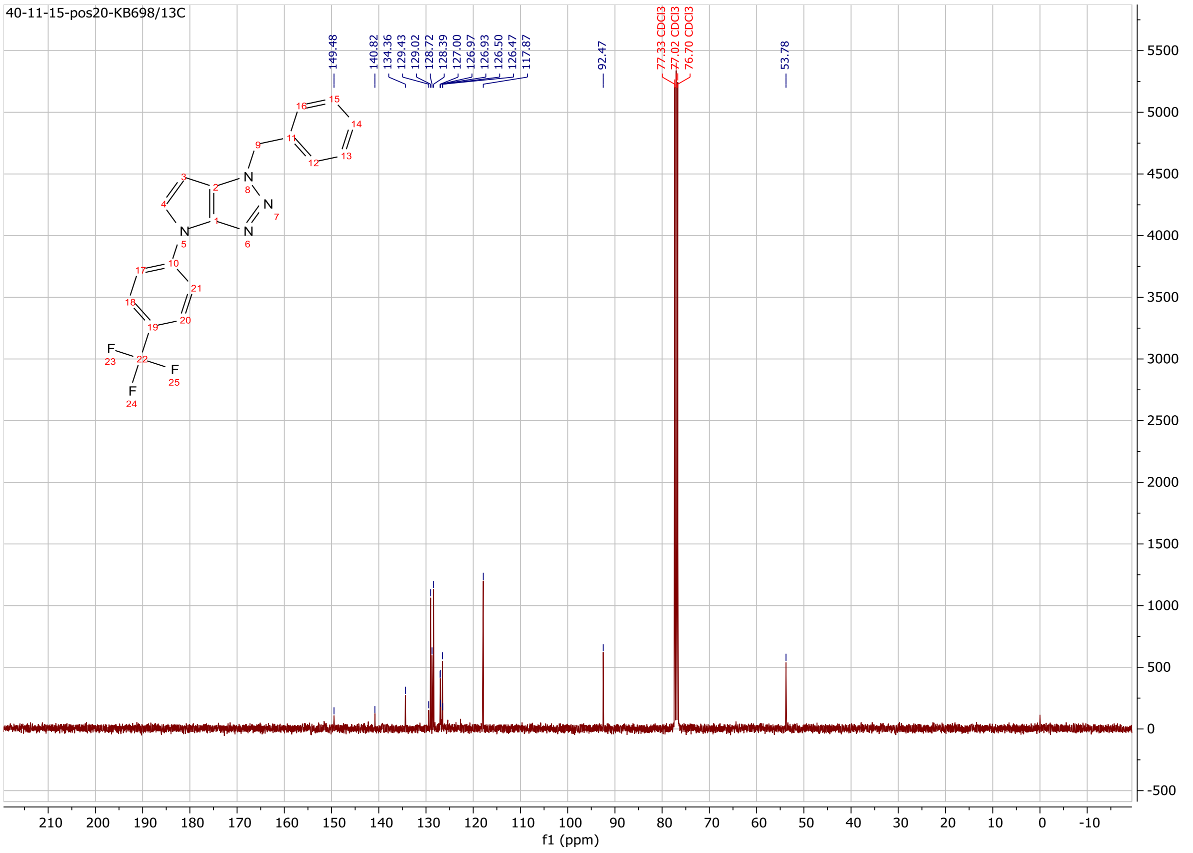
40-11-15-pos20-KB698/13C
149.48
140.82
134.36
129.43
129.02
128.72
128.39
127.00
126.97
126.93
126.50
126.47
117.87
92.47
77.33 CDCl3
77.02 CDCl3
76.70 CDCl3
53.78
f1 (ppm)

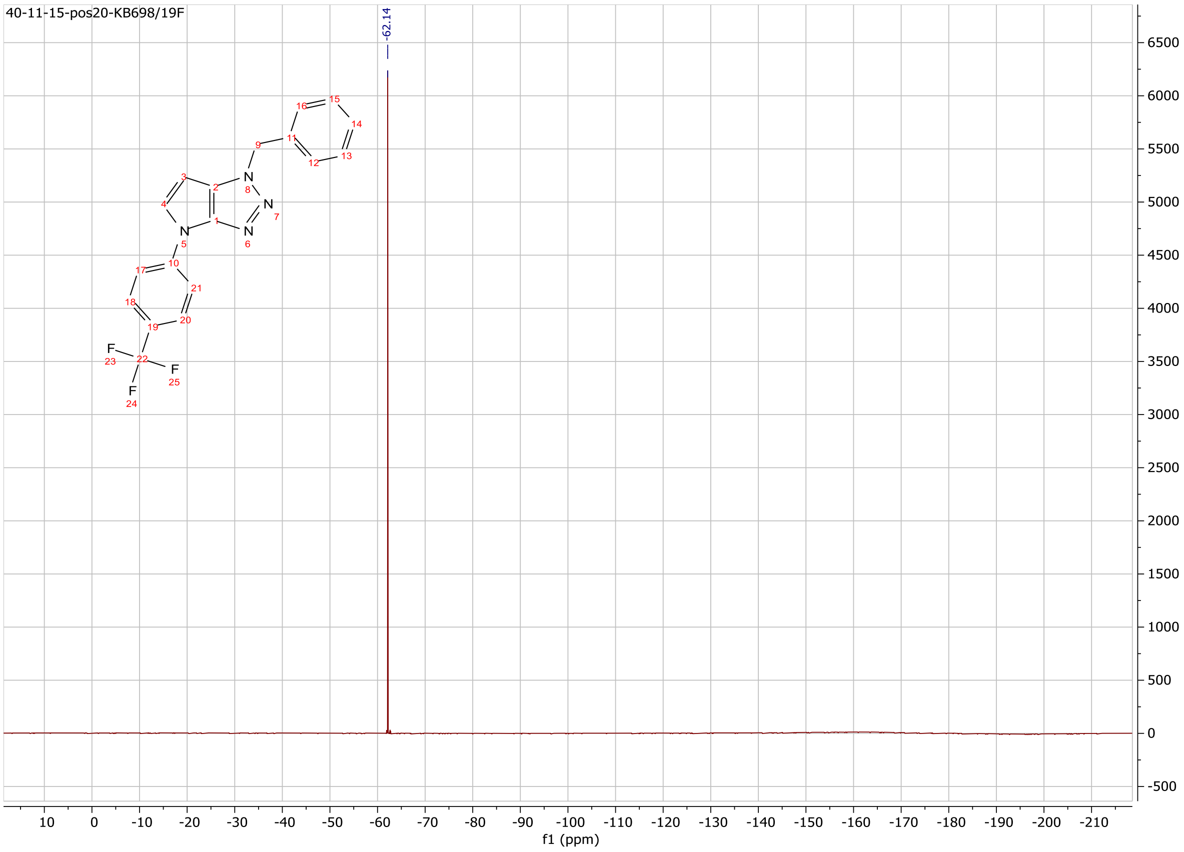
40-11-15-pos20-KB698/19F
-62.14
f1 (ppm)

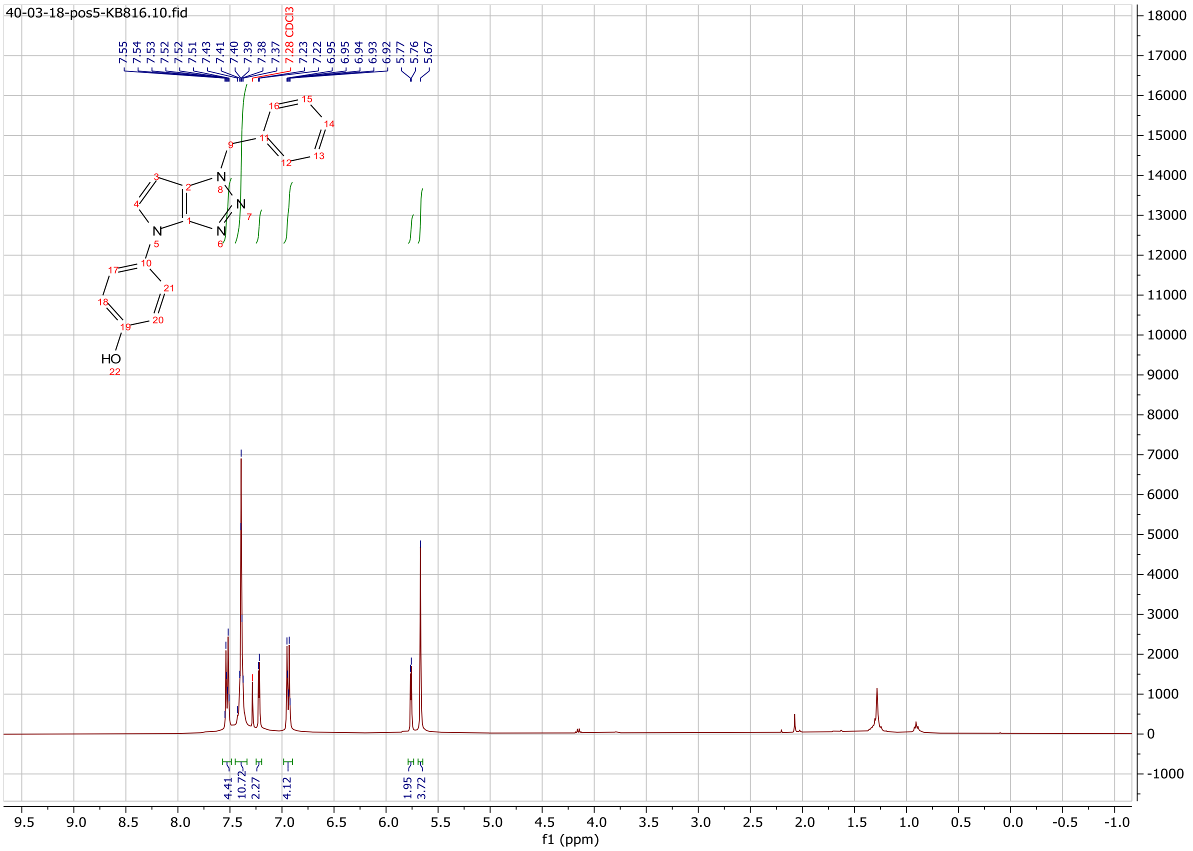
40-03-18-pos5-KB816.10.fid
f1 (ppm)

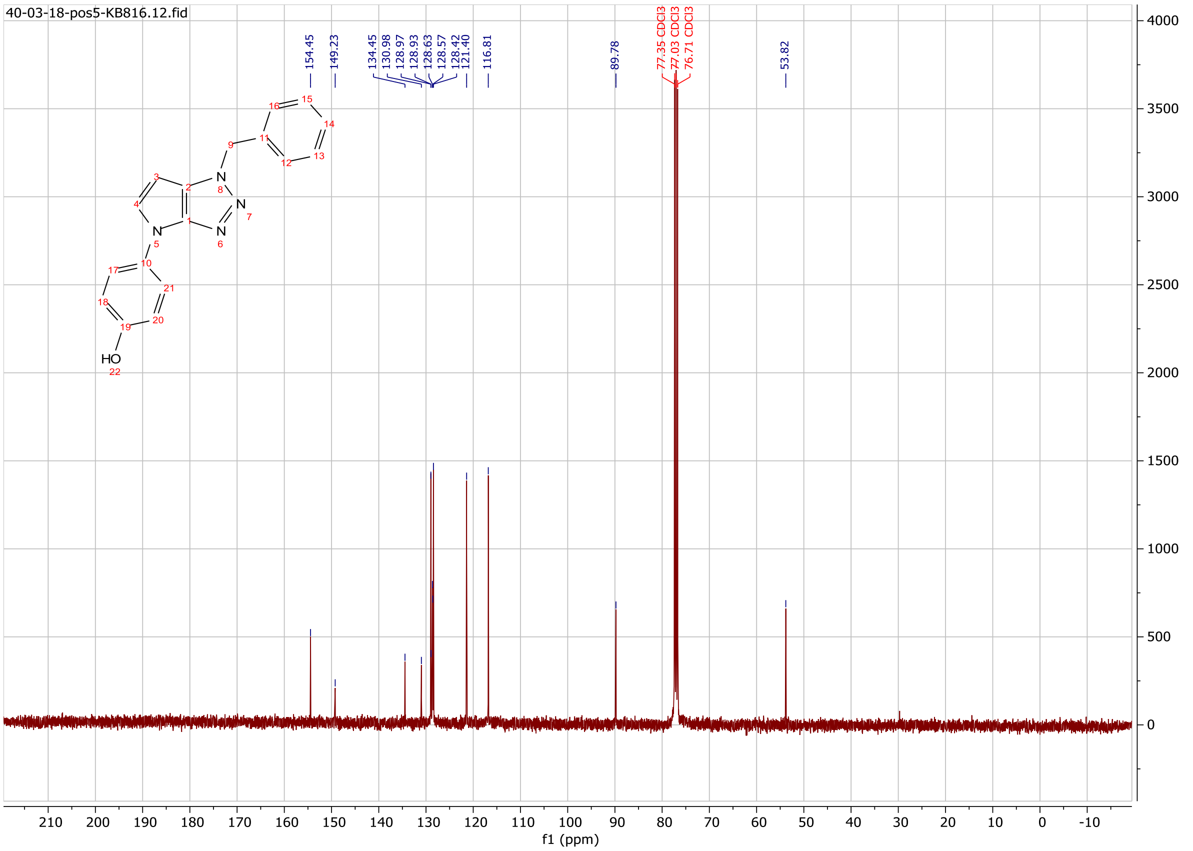
40-03-18-pos5-KB816.12.fid
f1 (ppm)

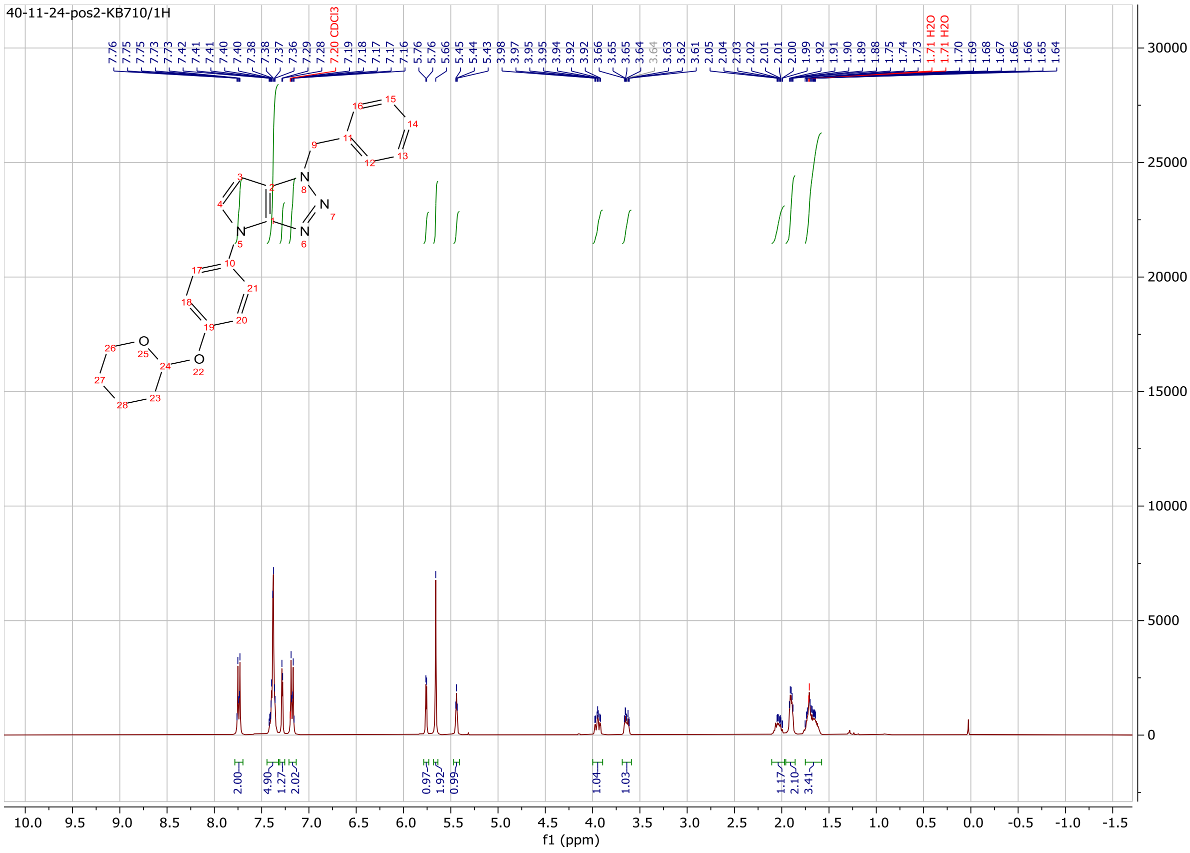
40-11-24-pos2-KB710/1H
f1 (ppm)

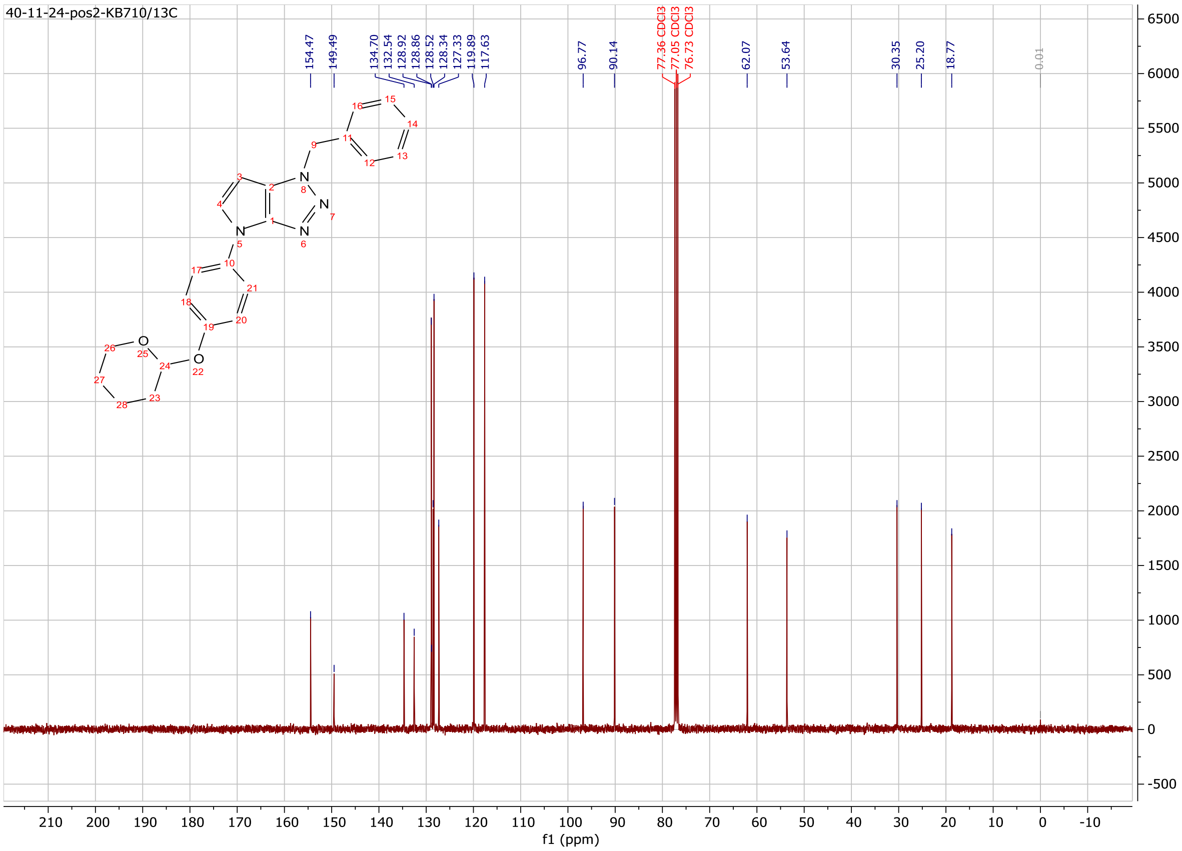
40-11-24-pos2-KB710/13C
f1 (ppm)

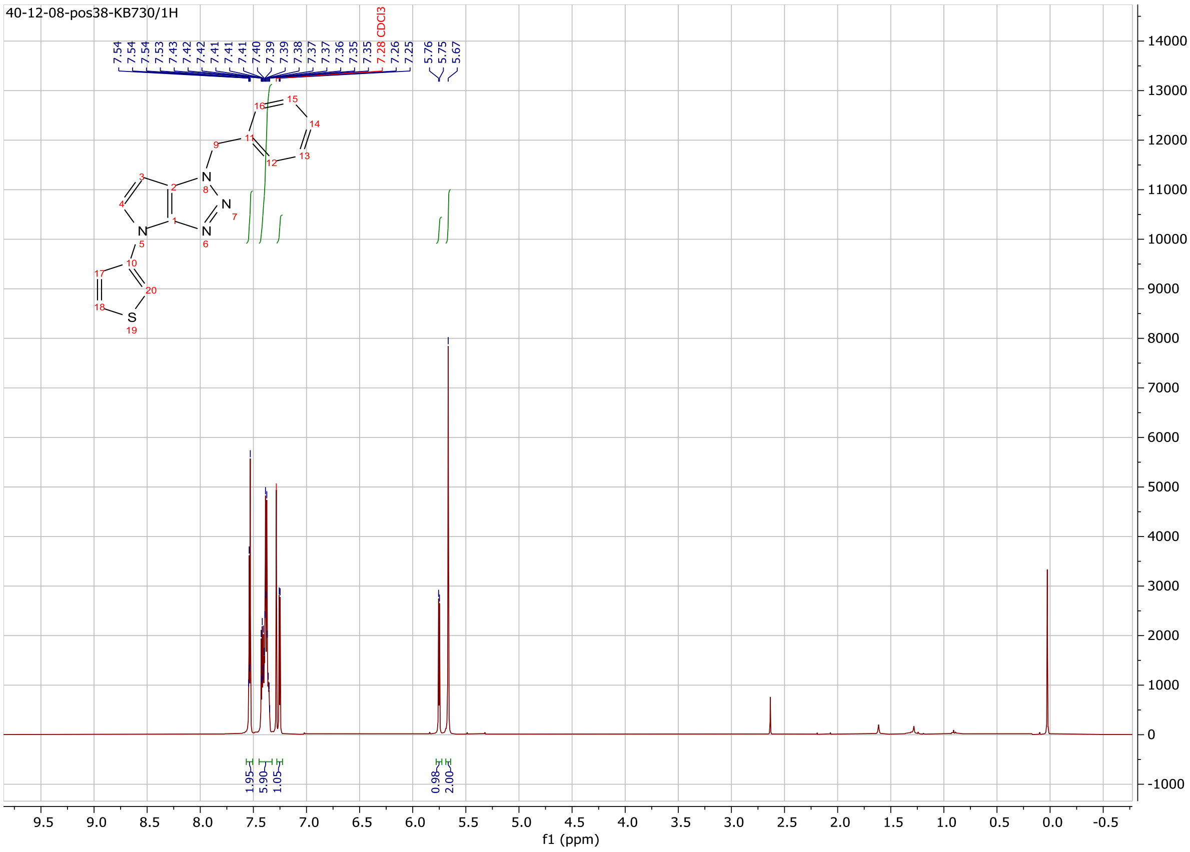
40-12-08-pos38-KB730/1H
7.54
7.54
7.54
7.53
7.43
7.42
7.42
7.41
7.41
7.40
7.39
7.39
7.38
7.37
7.37
7.36
7.35
7.28 CDCl3
7.26
7.25
5.76
5.75
5.67
1.95
5.90
1.05
0.98
2.00
f1 (ppm)

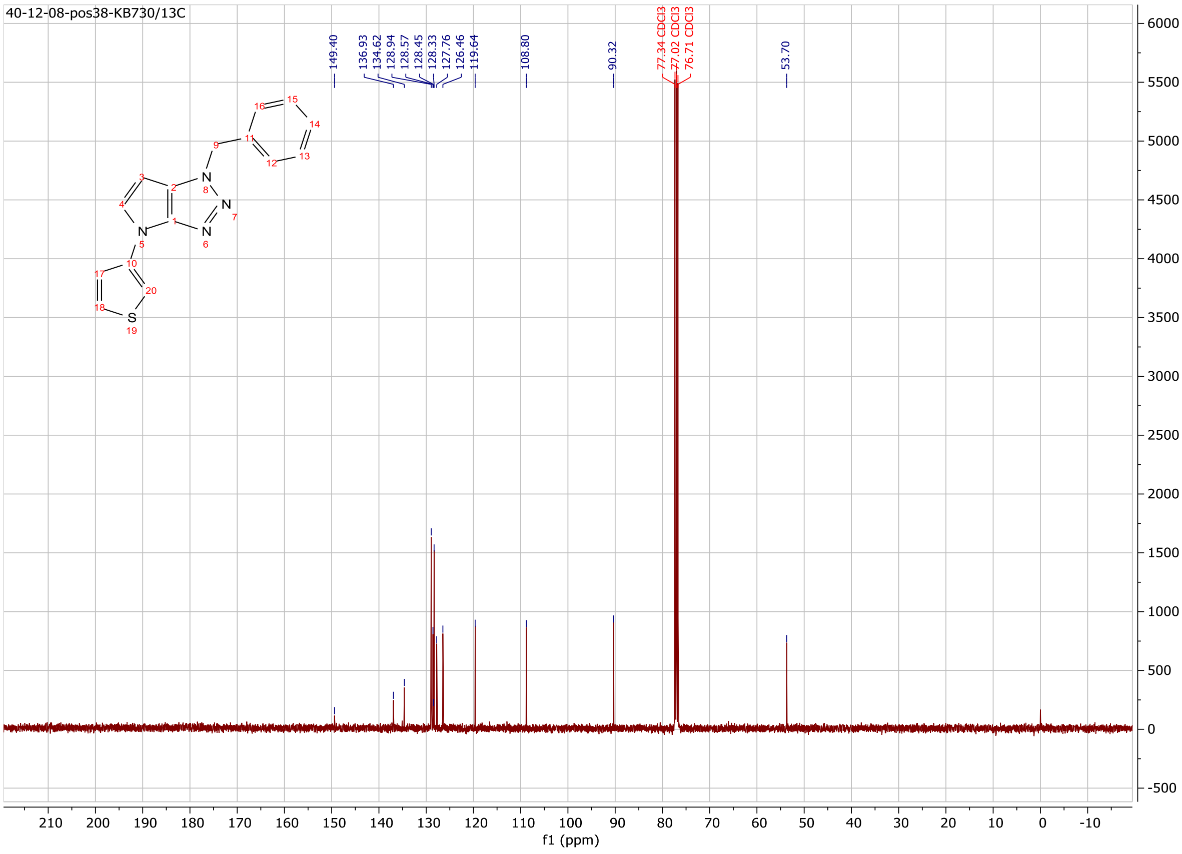
40-12-08-pos38-KB730/13C
149.40
136.93
134.62
128.94
128.57
128.45
128.33
127.76
126.46
119.64
108.80
90.32
77.34 CDCl3
77.02 CDCl3
76.71 CDCl3
53.70
f1 (ppm)

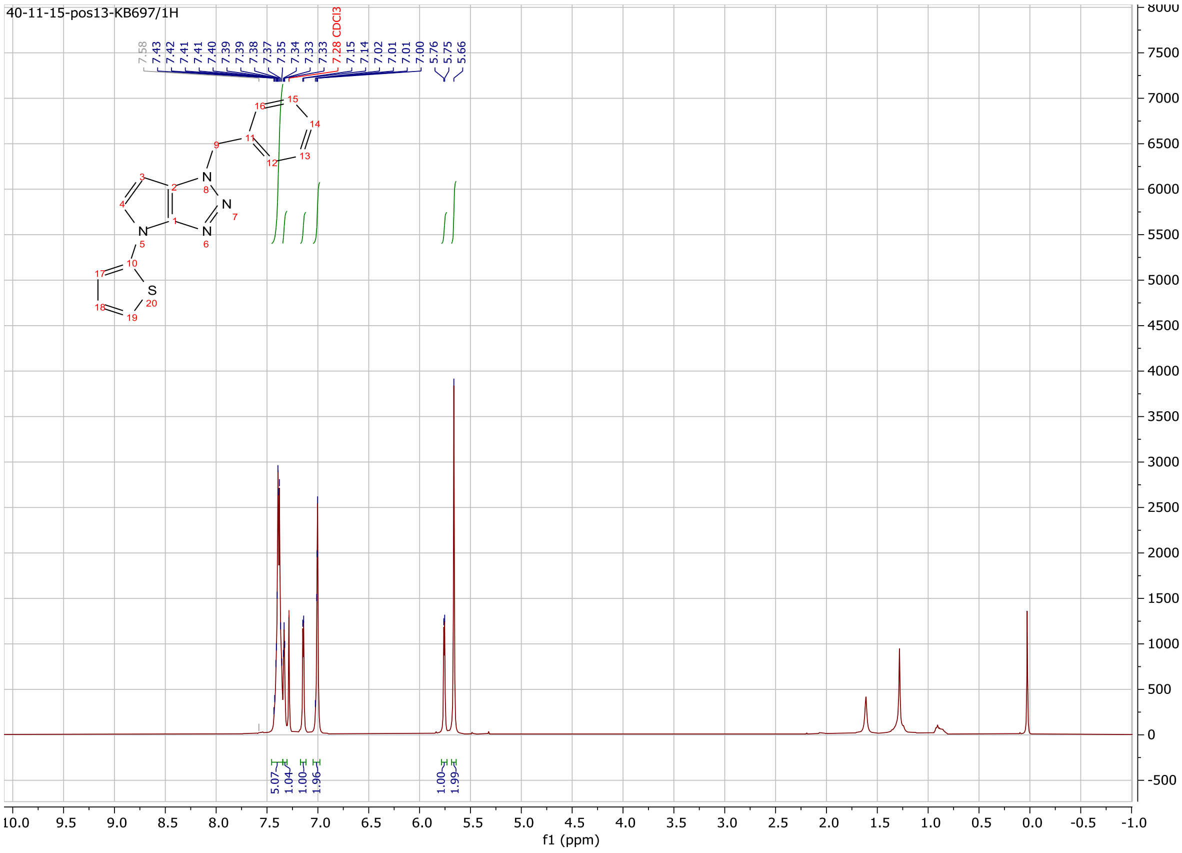
40-11-15-pos13-KB697/1H
7.58
7.43
7.42
7.41
7.41
7.40
7.39
7.39
7.38
7.37
7.35
7.34
7.33
7.33
7.28 CDCl3
7.15
7.14
7.02
7.01
7.01
7.00
5.76
5.75
5.66
5.07
1.04
1.00
1.96
1.00
1.99
f1 (ppm)

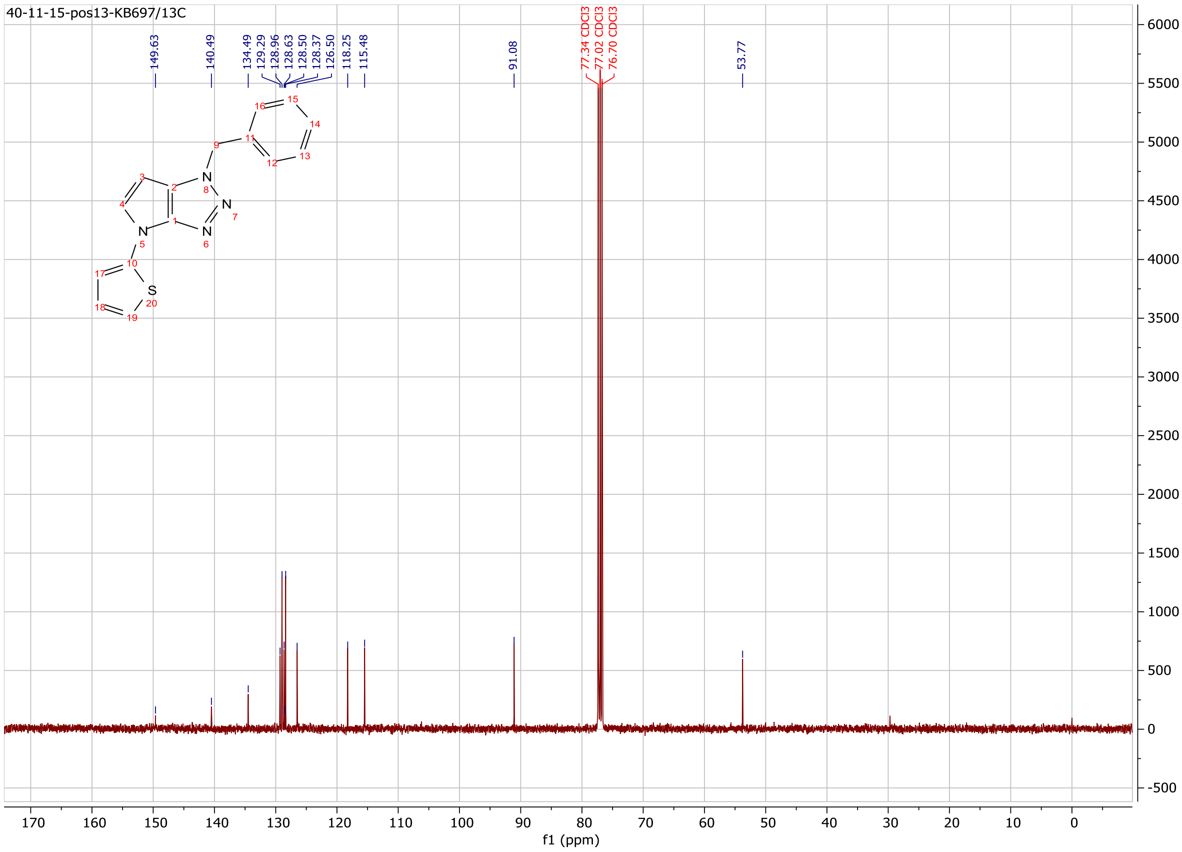
40-11-15-pos13-KB697/13C
149.63
140.49
134.49
129.29
128.96
128.63
128.50
128.37
126.50
118.25
115.48
91.08
77.34 CDCl3
77.02 CDCl3
76.70 CDCl3
53.77
f1 (ppm)

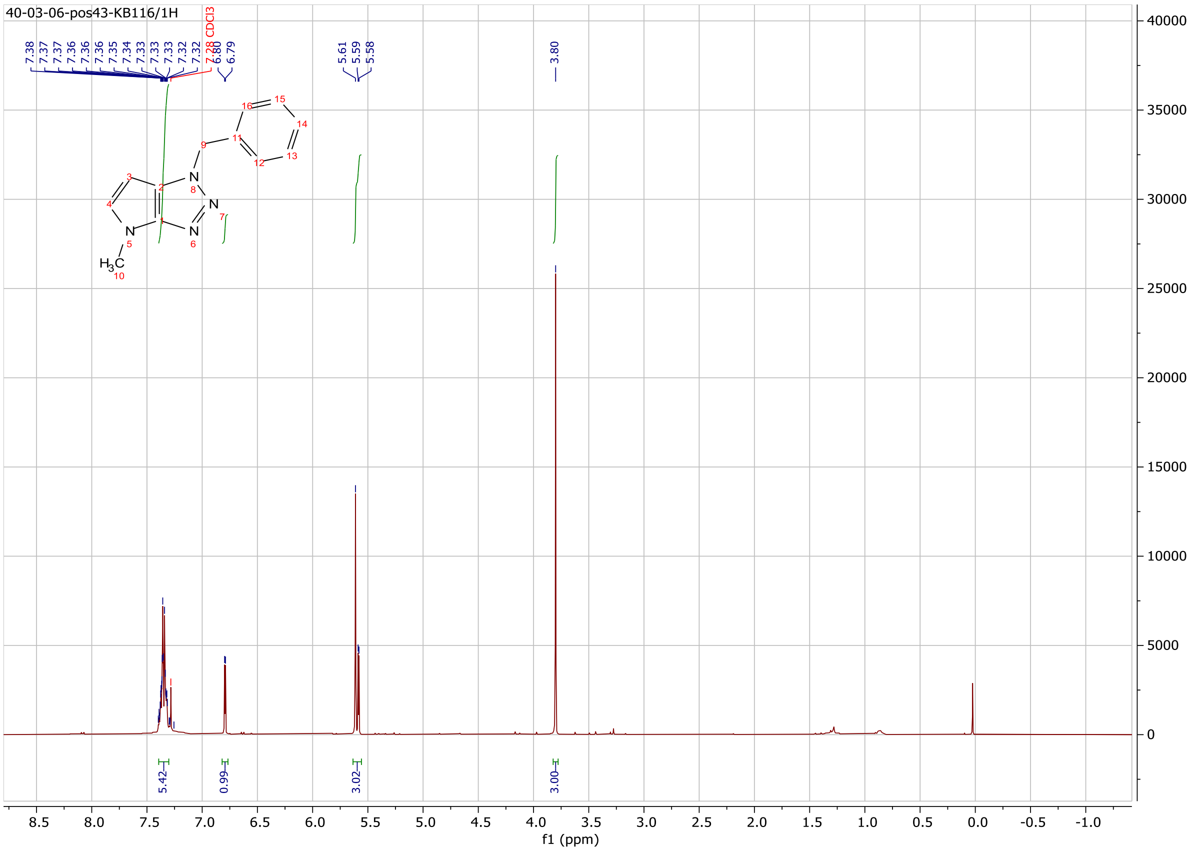
40-03-06-pos43-KB116/1H
7.38
7.37
7.37
7.36
7.36
7.36
7.35
7.34
7.33
7.33
7.33
7.32
7.32
7.28 CDCl3
6.80
6.79
5.61
5.59
5.58
3.80
5.42
0.99
3.02
3.00
f1 (ppm)

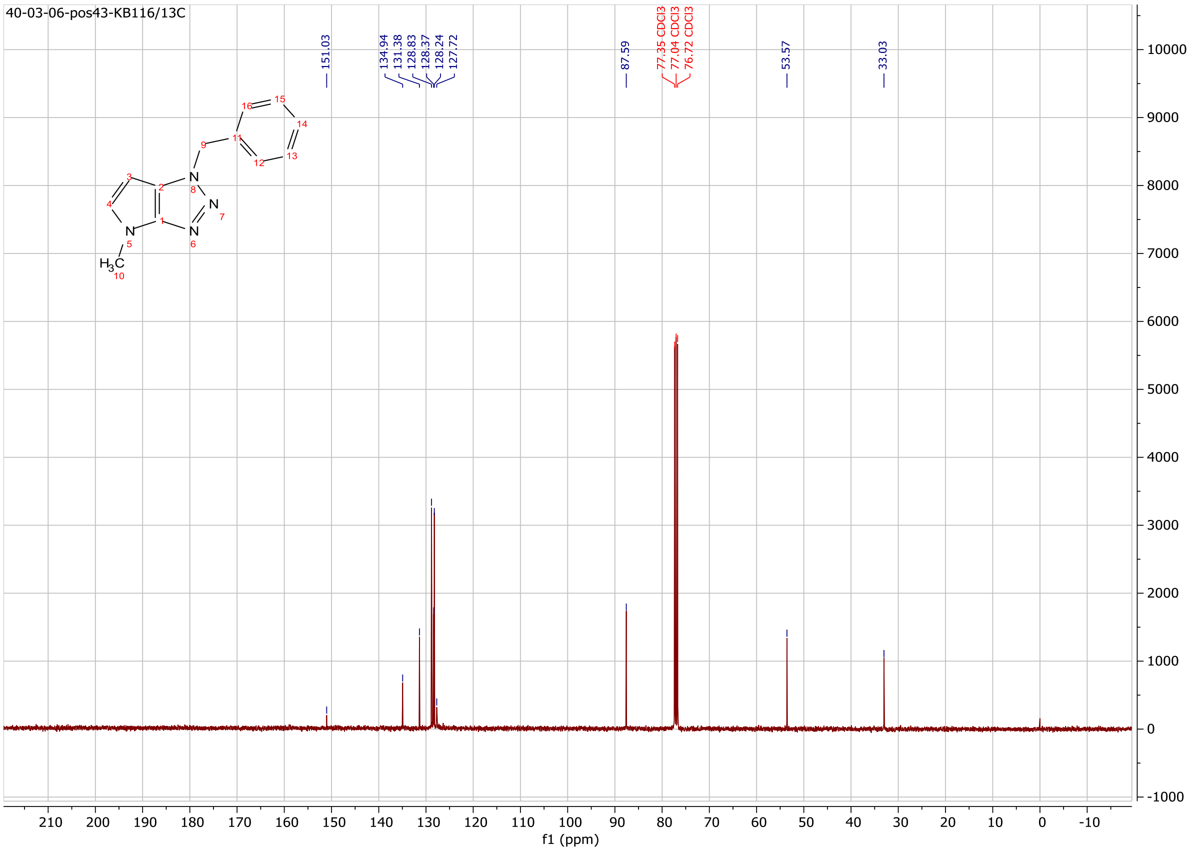
40-03-06-pos43-KB116/13C
151.03
134.94
131.38
128.83
128.37
128.24
127.72
87.59
77.35 CDCl3
77.04 CDCl3
76.72 CDCl3
53.57
33.03
f1 (ppm)

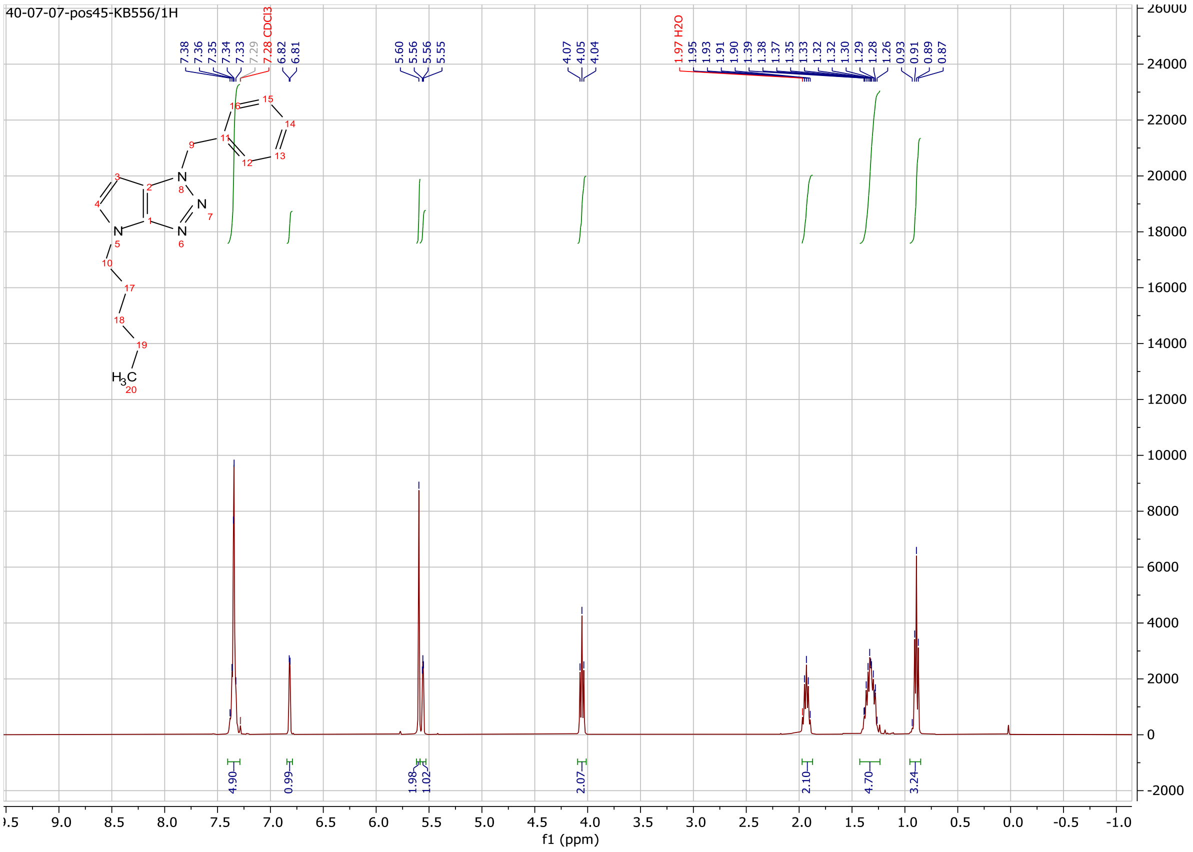
40-07-07-pos45-KB556/1H
f1 (ppm)

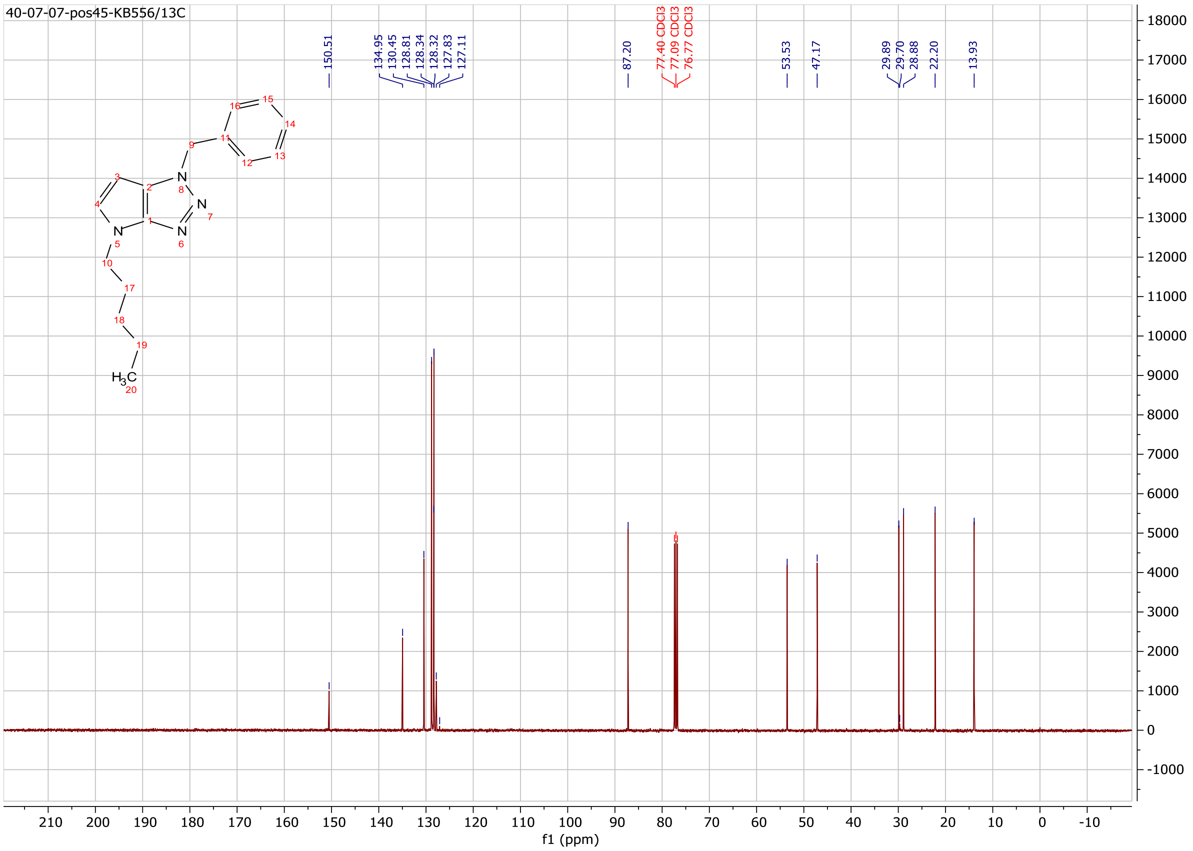
40-07-07-pos45-KB556/13C
f1 (ppm)

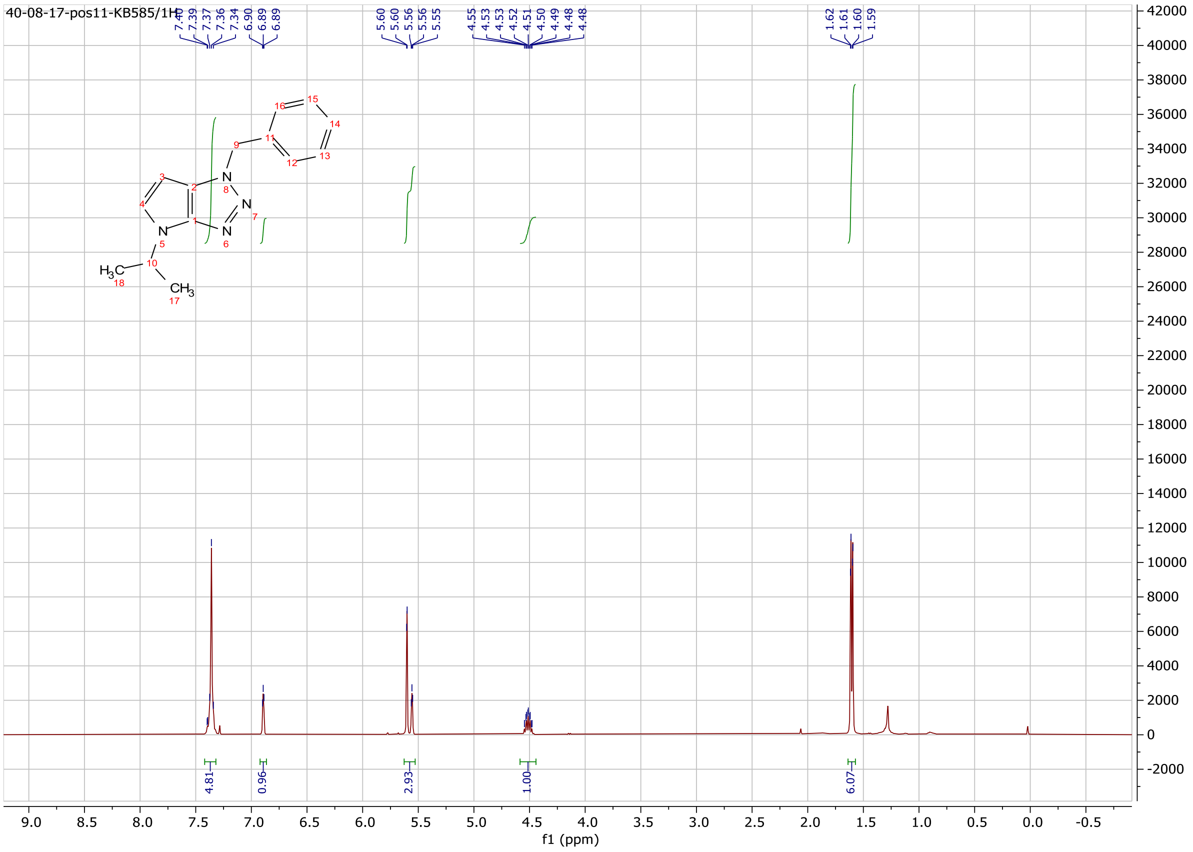
40-08-17-pos11-KB585/1H
7.40
7.39
7.37
7.36
7.34
6.90
6.89
6.89
5.60
5.60
5.56
5.56
5.55
4.55
4.53
4.53
4.52
4.51
4.50
4.49
4.48
4.48
1.62
1.61
1.60
1.59
4.81
0.96
2.93
1.00
6.07
f1 (ppm)

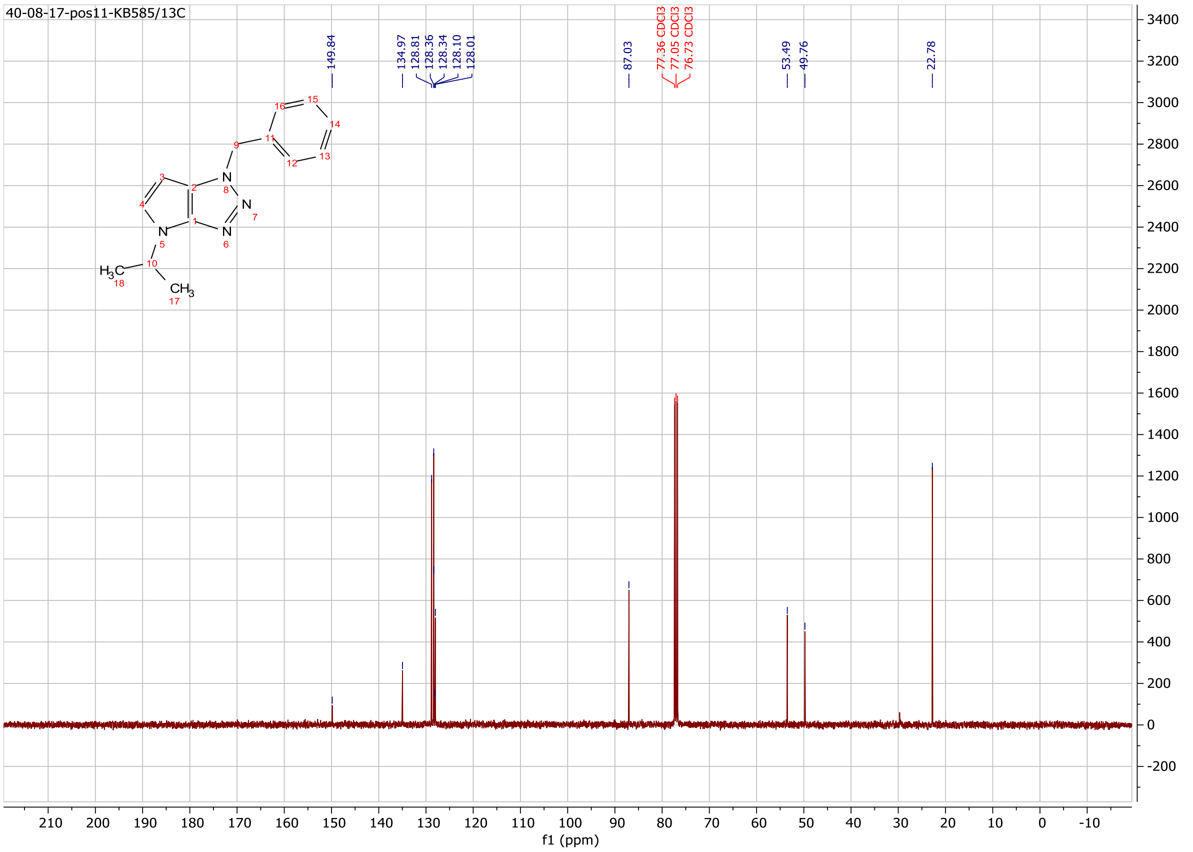
40-08-17-pos11-KB585/13C
149.84
134.97
128.81
128.36
128.34
128.10
128.01
87.03
77.36 CDCl3
77.05 CDCl3
76.73 CDCl3
53.49
49.76
22.78
f1 (ppm)

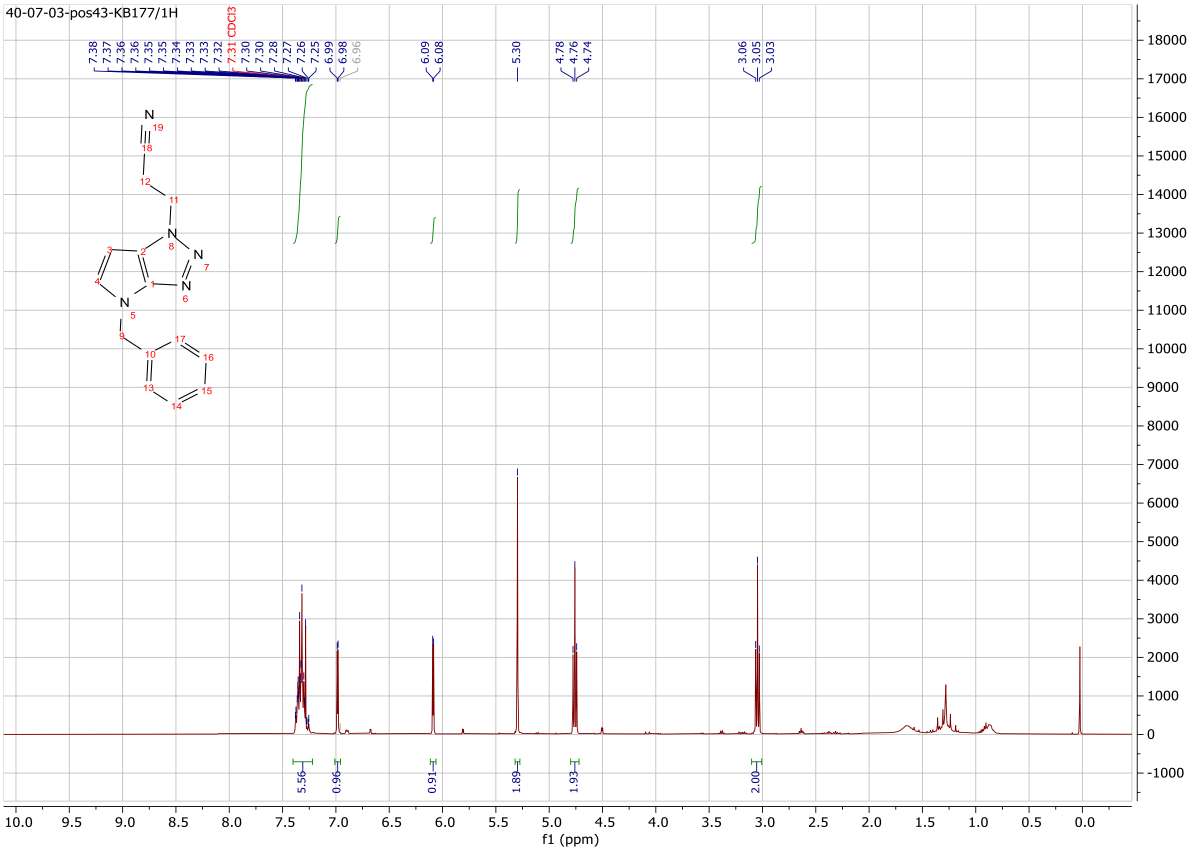
40-07-03-pos43-KB177/1H
7.38
7.37
7.36
7.36
7.35
7.35
7.34
7.33
7.33
7.32
7.31 CDCl3
7.30
7.30
7.28
7.27
7.26
7.25
6.99
6.98
6.96
6.09
6.08
5.30
4.78
4.76
4.74
3.06
3.05
3.03
5.56
0.96
0.91
1.89
1.93
2.00
f1 (ppm)

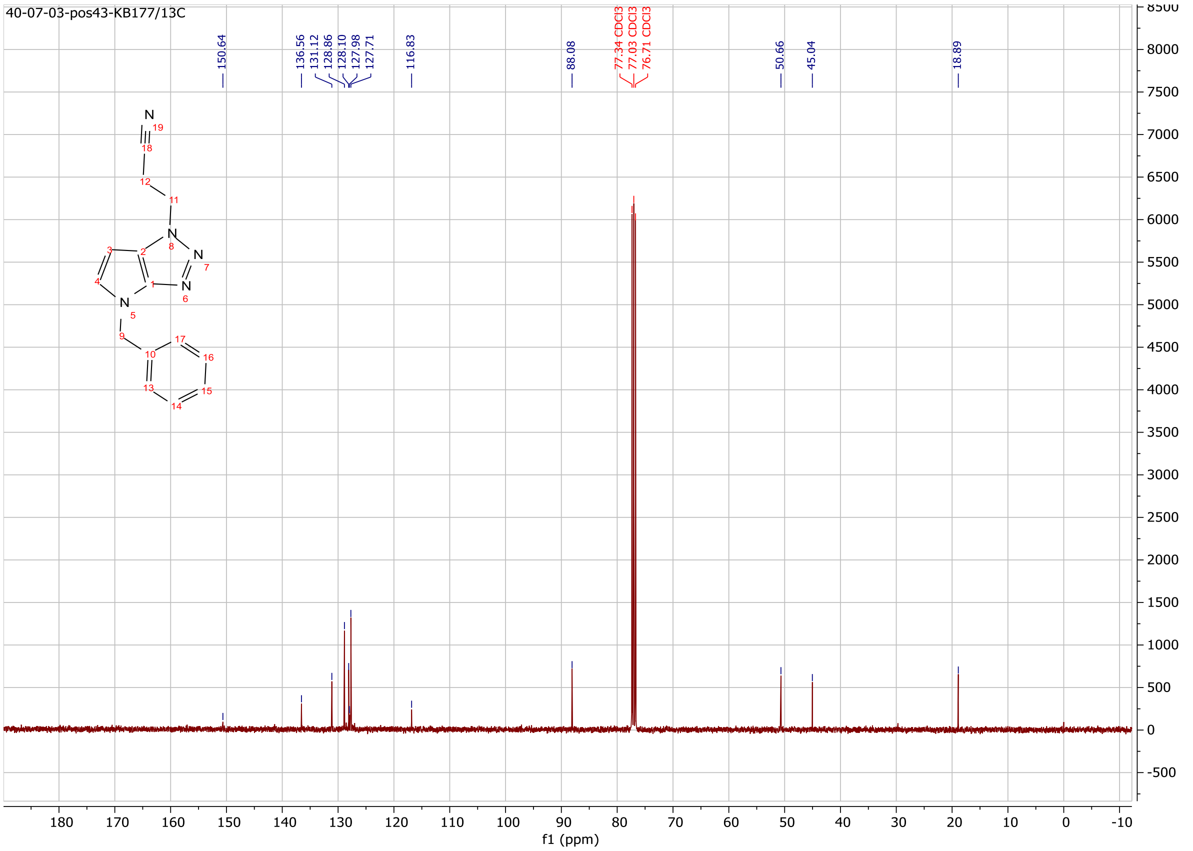
40-07-03-pos43-KB177/13C
150.64
136.56
131.12
128.86
128.10
127.98
127.71
116.83
88.08
77.34 CDCl3
77.03 CDCl3
76.71 CDCl3
50.66
45.04
18.89
f1 (ppm)

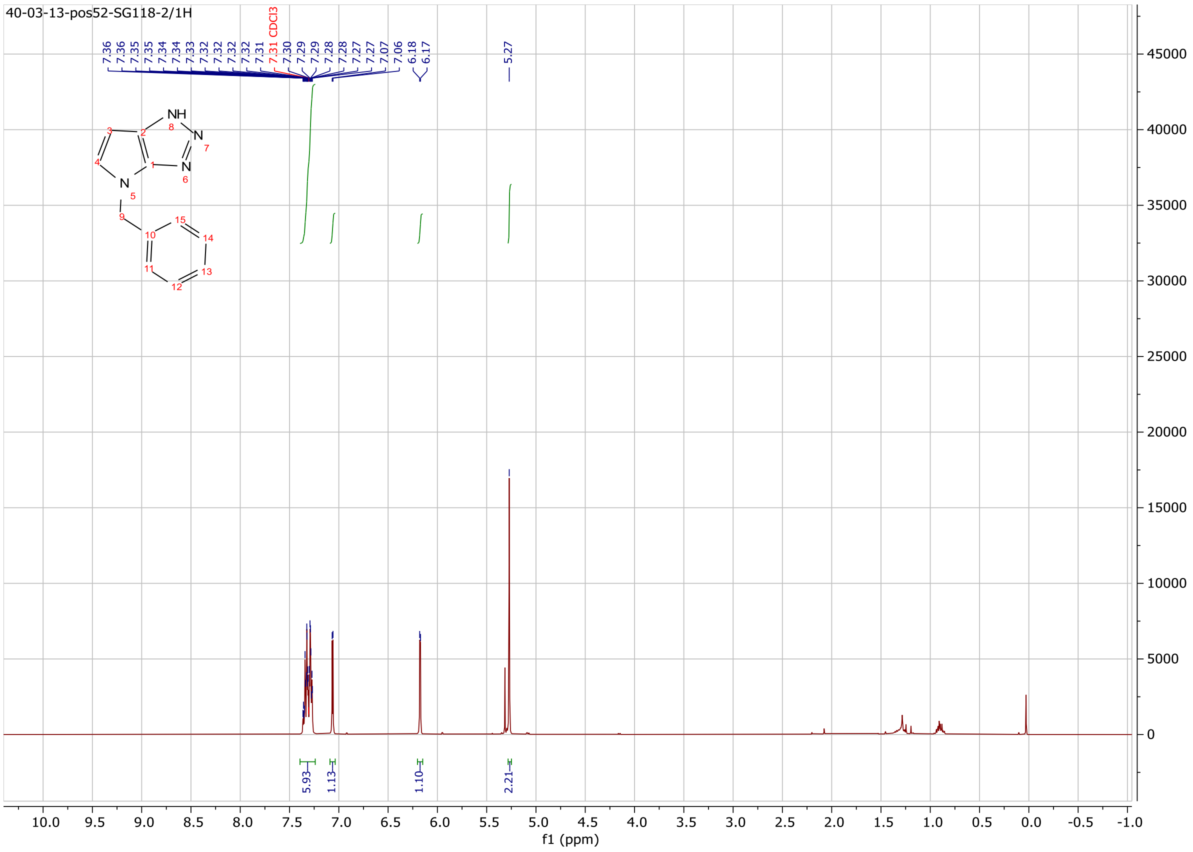
40-03-13-pos52-SG118-2/1H
7.36
7.36
7.35
7.35
7.34
7.34
7.33
7.32
7.32
7.32
7.32
7.31
7.31 CDCl3
7.30
7.29
7.29
7.28
7.28
7.27
7.27
7.07
7.06
6.18
6.17
5.27
NH
N
N
N
5.93
1.13
1.10
2.21
f1 (ppm)

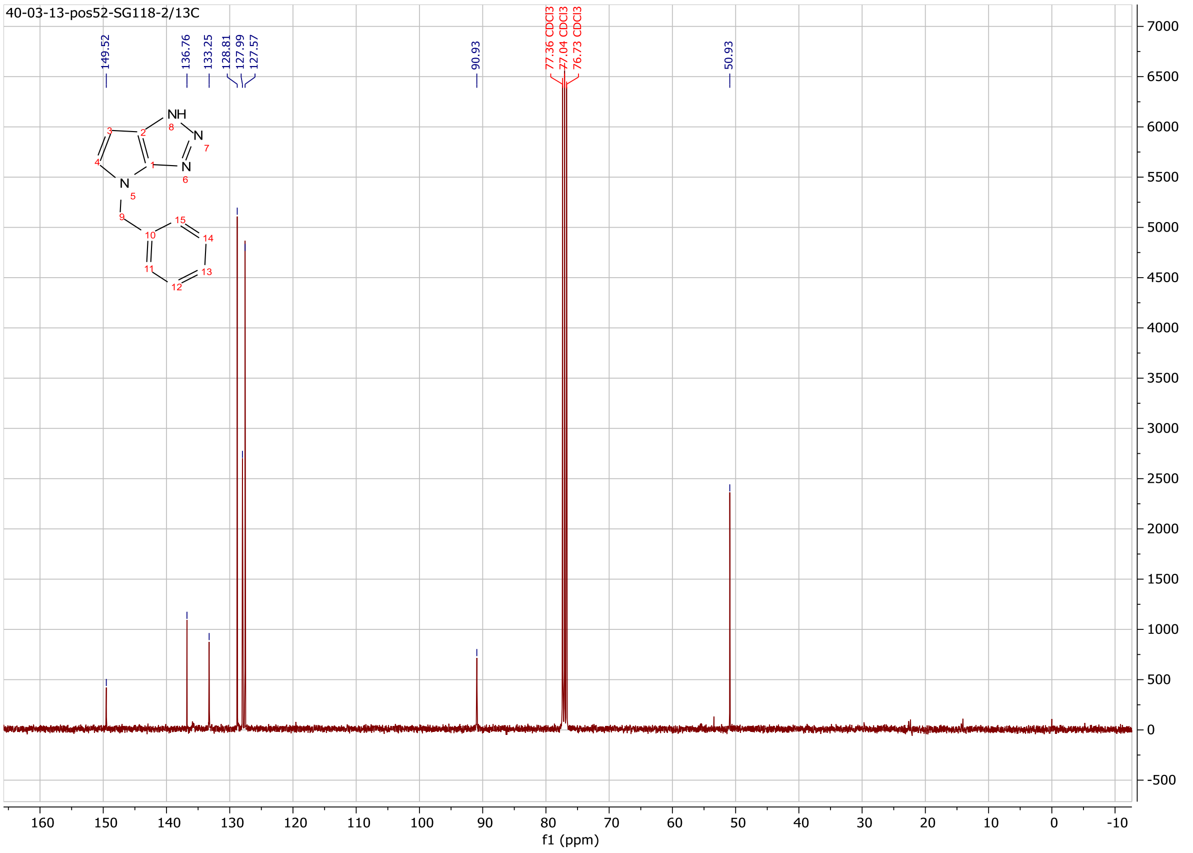
40-03-13-pos52-SG118-2/13C
149.52
136.76
133.25
128.81
127.99
127.57
90.93
77.36 CDCl3
77.04 CDCl3
76.73 CDCl3
50.93
NH
N
N
N
f1 (ppm)

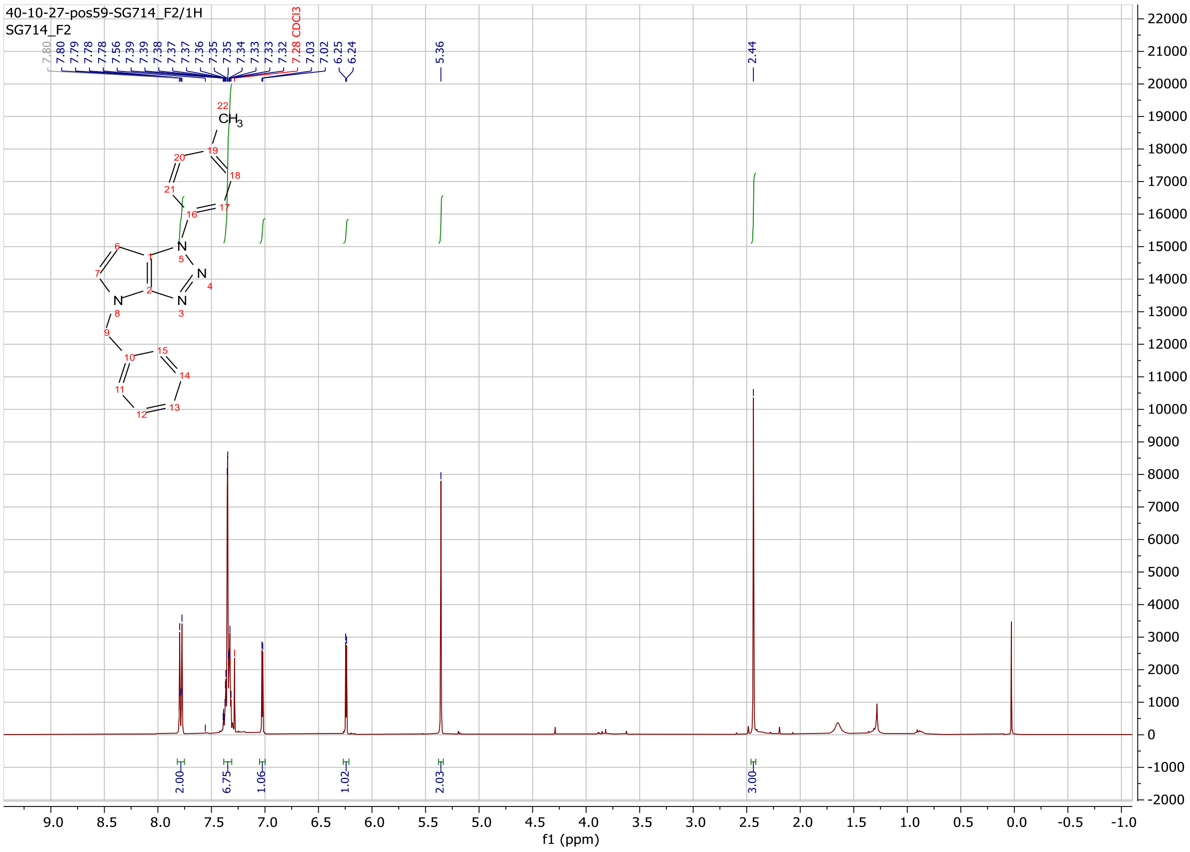
40-10-27-pos59-SG714_F2/1H
SG714_F2
7.80 7.79 7.78 7.78 7.56 7.39 7.39 7.38 7.37 7.37 7.36 7.35 7.35 7.34 7.33 7.33 7.32 7.28 CDCl3 7.03 7.02 6.25 6.24
5.36
2.44
2.00 6.75 1.06 1.02 2.03 3.00
f1 (ppm)

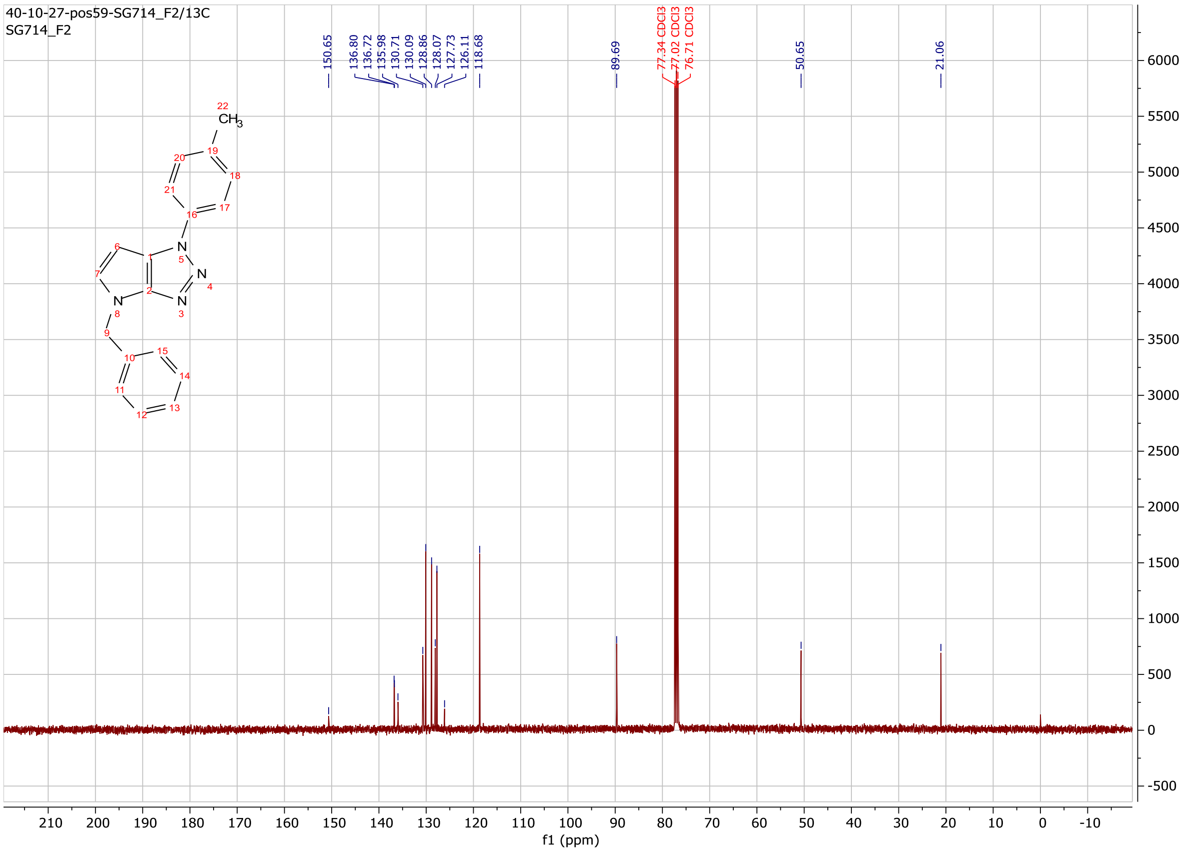
40-10-27-pos59-SG714_F2/13C
SG714_F2
150.65
136.80 136.72 135.98 130.71 130.09 128.86 128.07 127.73 126.11 118.68
89.69
77.34 CDCl3 77.02 CDCl3 76.71 CDCl3
50.65
21.06
f1 (ppm)

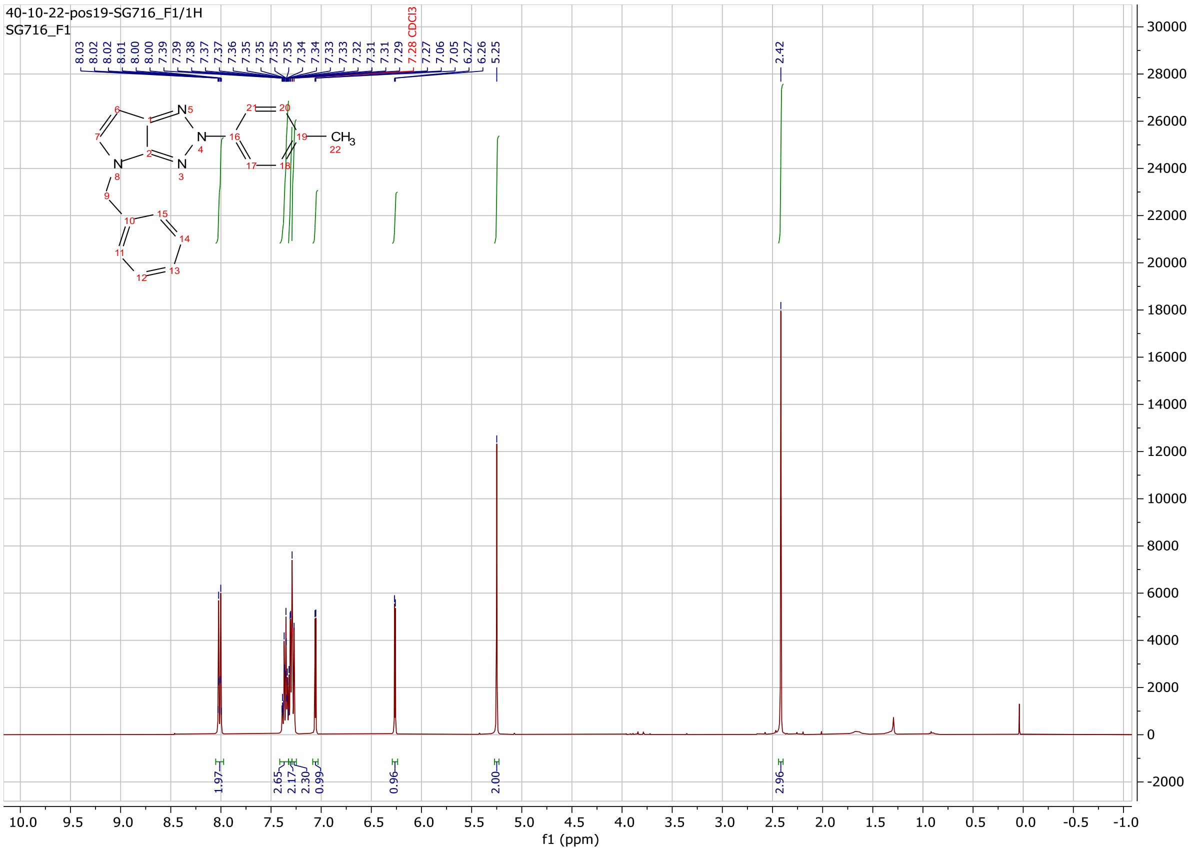
40-10-22-pos19-SG716_F1/1H
SG716_F1
8.03 8.02 8.02 8.01 8.00 8.00 7.39 7.39 7.38 7.37 7.37 7.36 7.35 7.35 7.35 7.35 7.34 7.34 7.33 7.33 7.32 7.31 7.31 7.29 7.28 CDCl3 7.27 7.06 7.05 6.27 6.26 5.25
2.42
1.97 2.65 2.17 2.30 0.99 0.96 2.00 2.96
f1 (ppm)

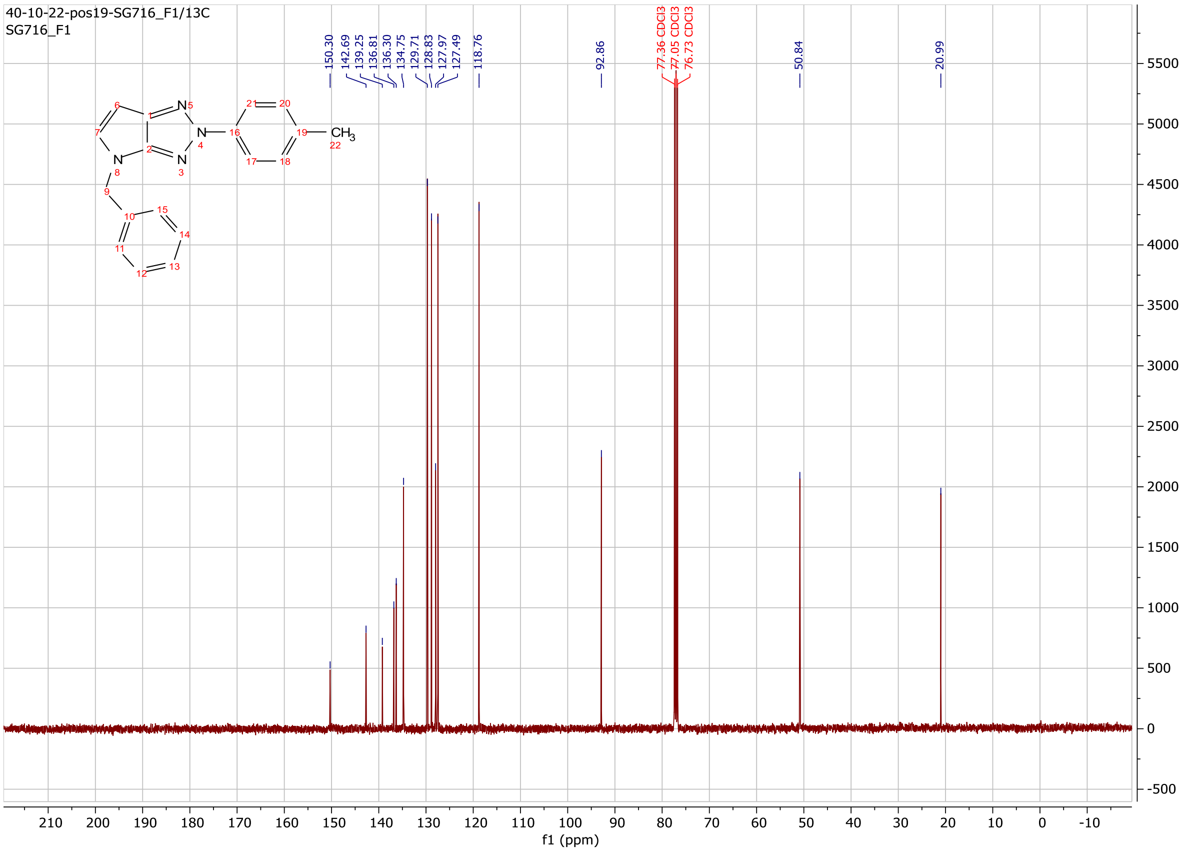
40-10-22-pos19-SG716_F1/13C
SG716_F1
150.30 142.69 139.25 136.81 136.30 134.75 129.71 128.83 127.97 127.49 118.76
92.86
77.36 CDCl3 77.05 CDCl3 76.73 CDCl3
50.84
20.99
f1 (ppm)

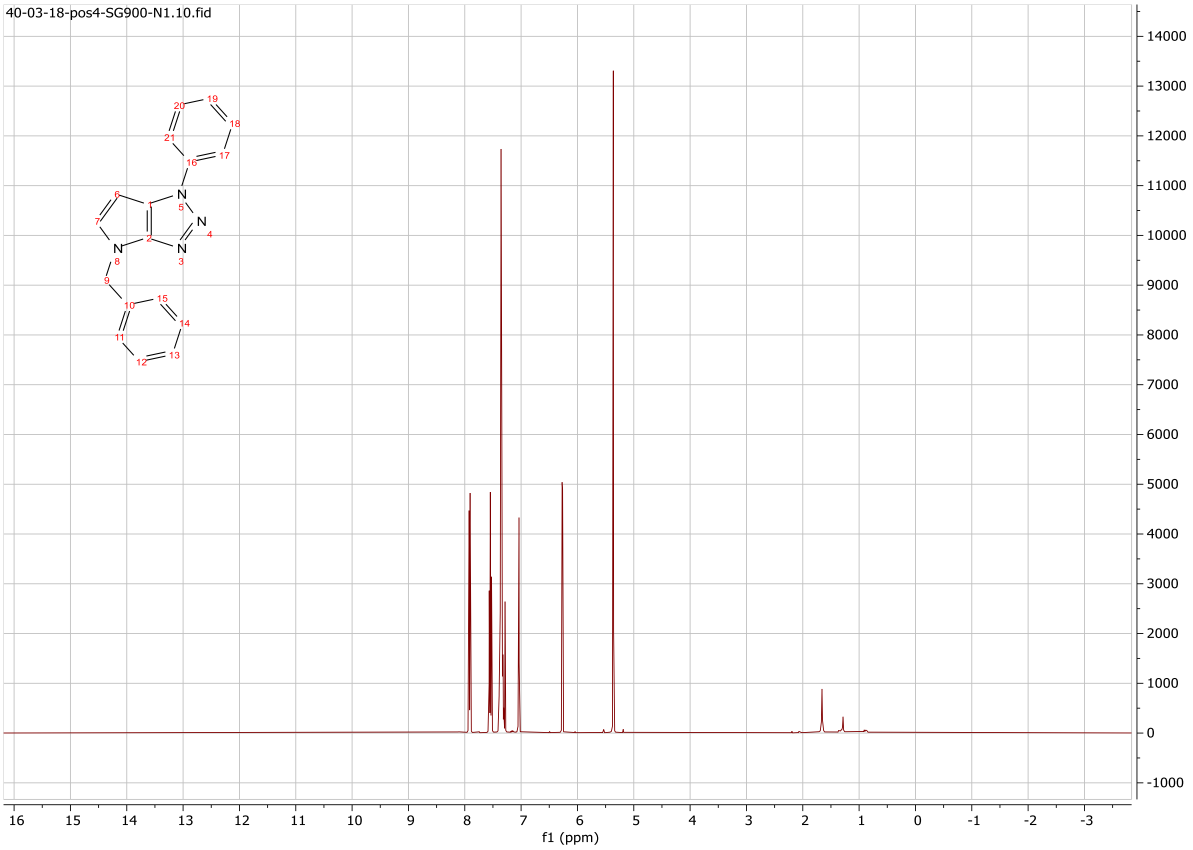
40-03-18-pos4-SG900-N1.10.fid
f1 (ppm)

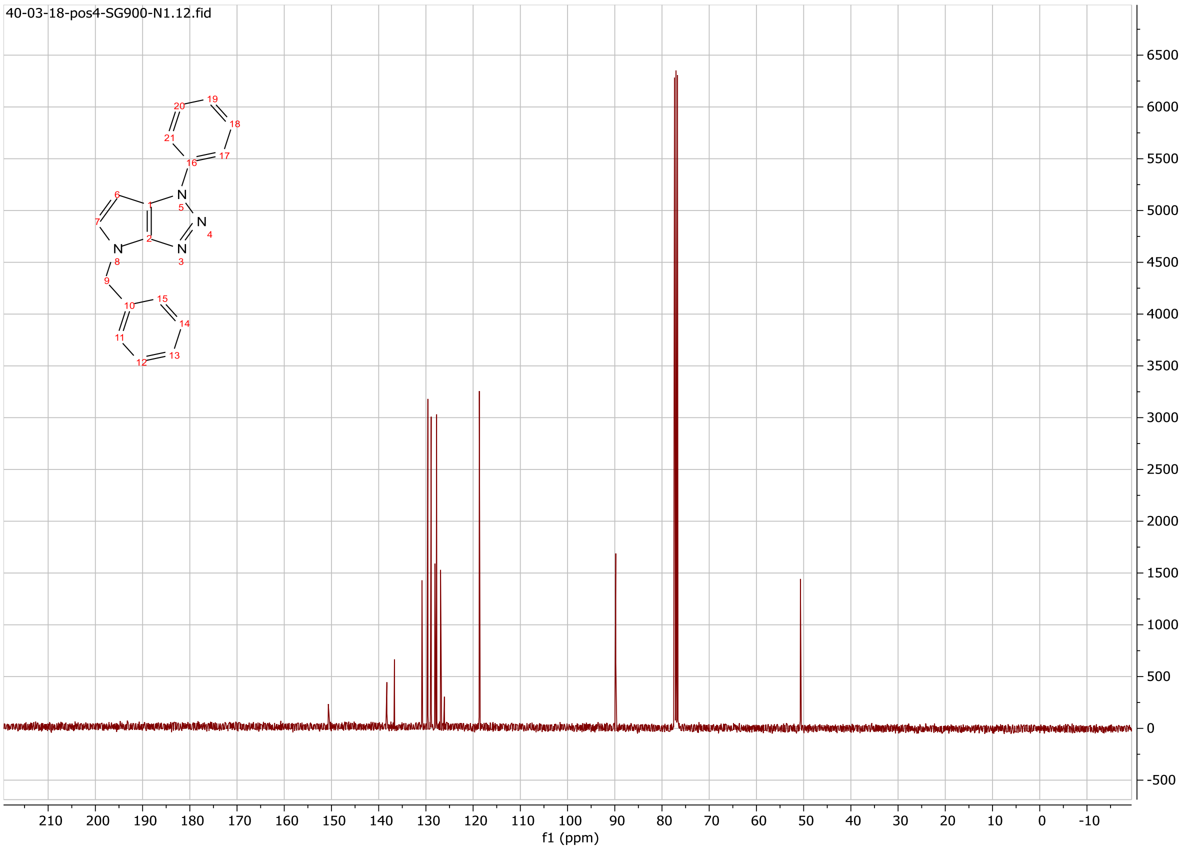
40-03-18-pos4-SG900-N1.12.fid
f1 (ppm)

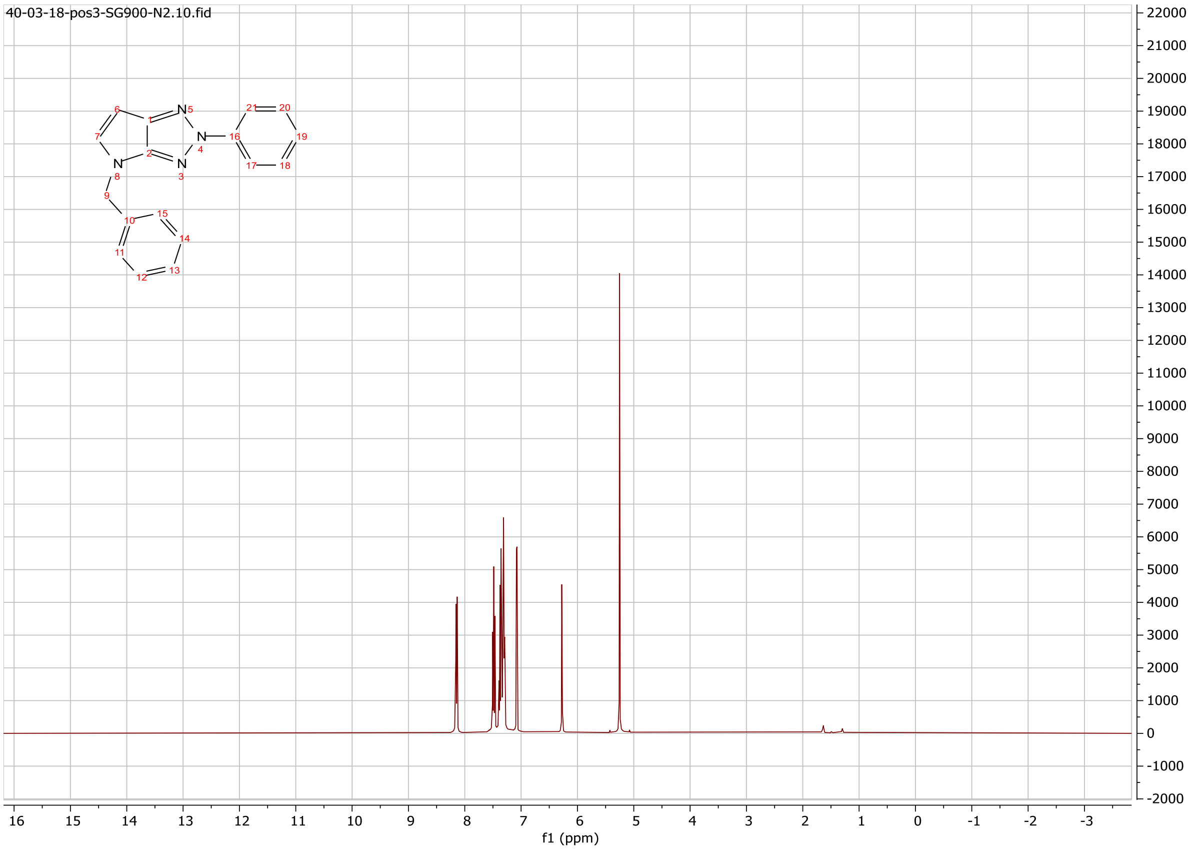
40-03-18-pos3-SG900-N2.10.fid
f1 (ppm)

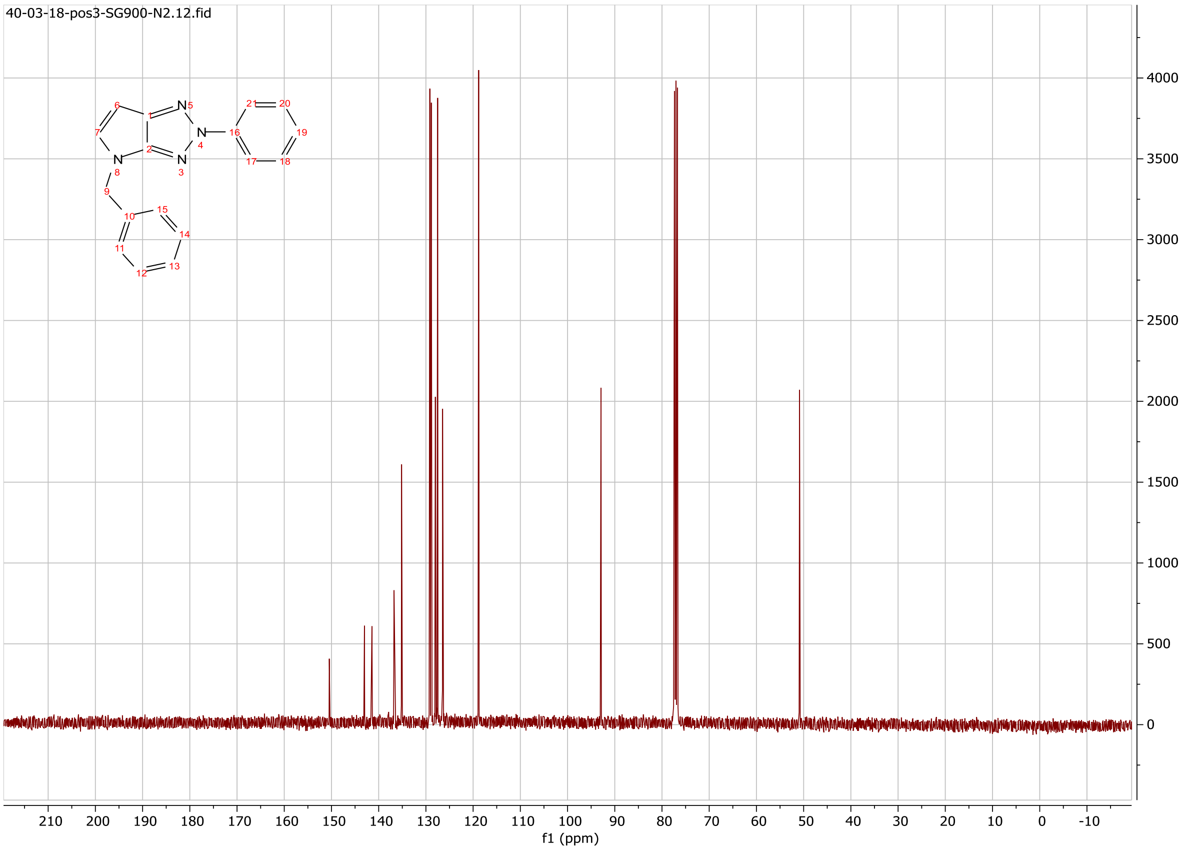
40-03-18-pos3-SG900-N2.12.fid
f1 (ppm)

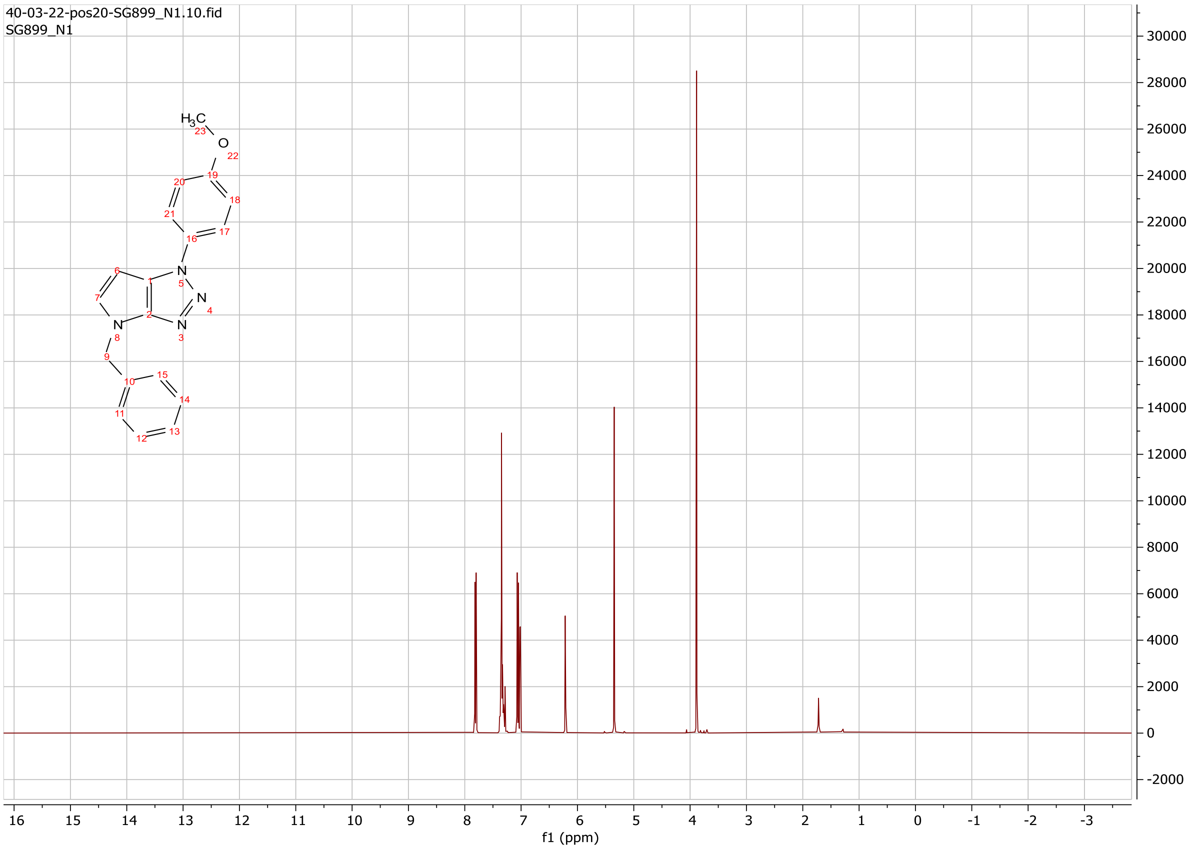
40-03-22-pos20-SG899_N1.10.fid
SG899_N1
f1 (ppm)

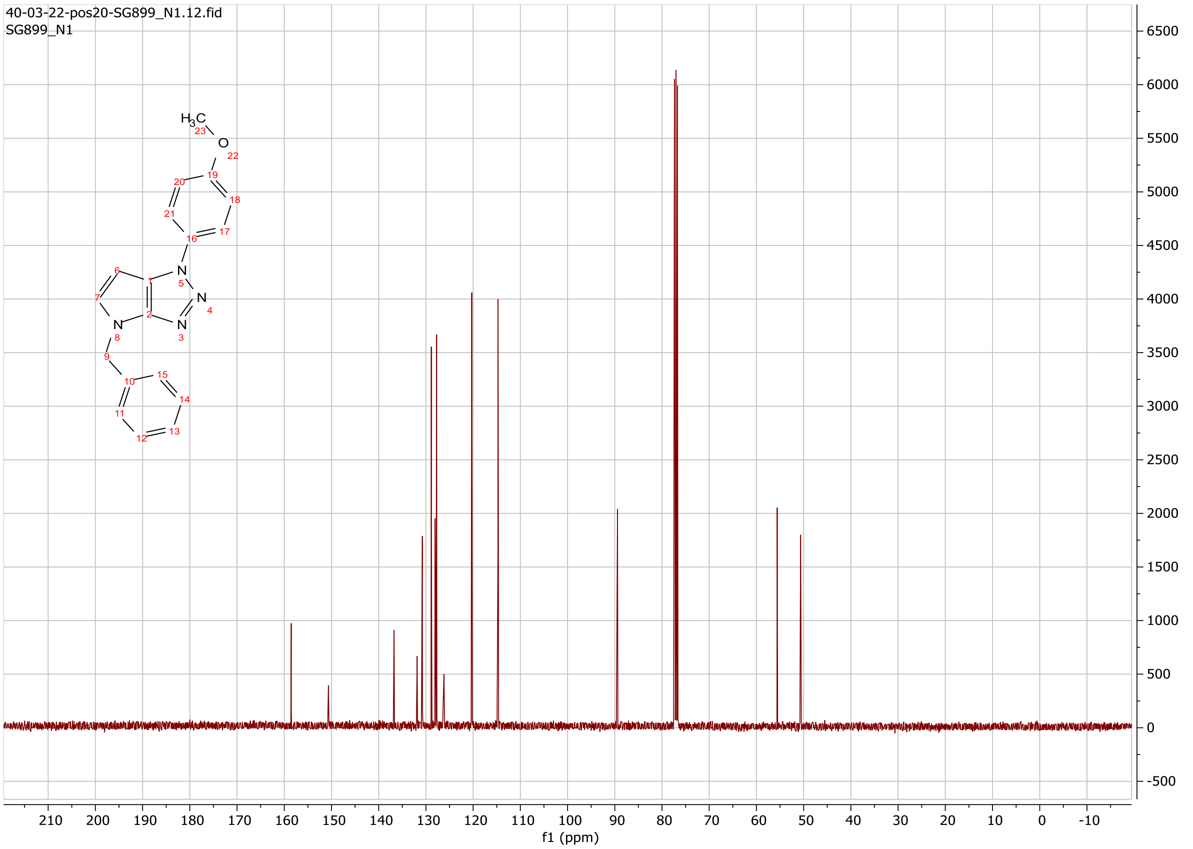
40-03-22-pos20-SG899_N1.12.fid
SG899_N1
f1 (ppm)

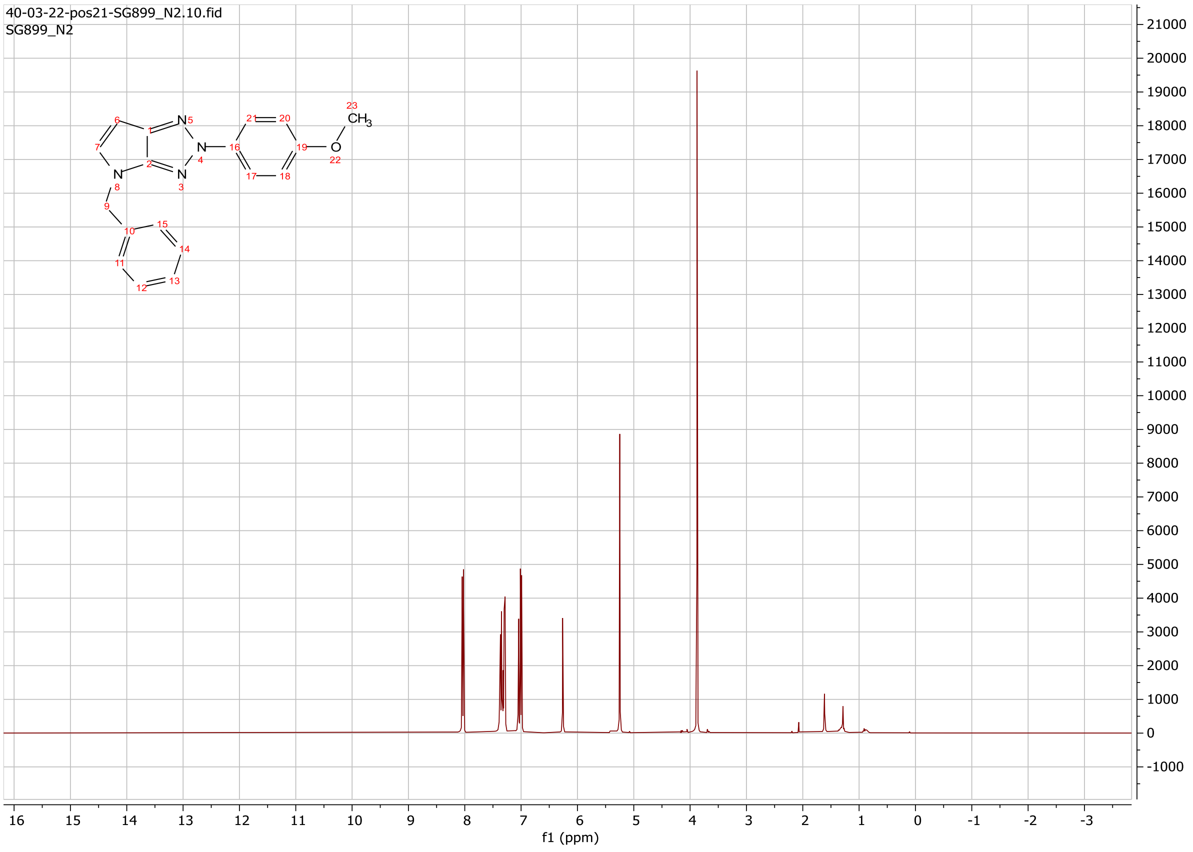
40-03-22-pos21-SG899_N2.10.fid
SG899_N2
f1 (ppm)

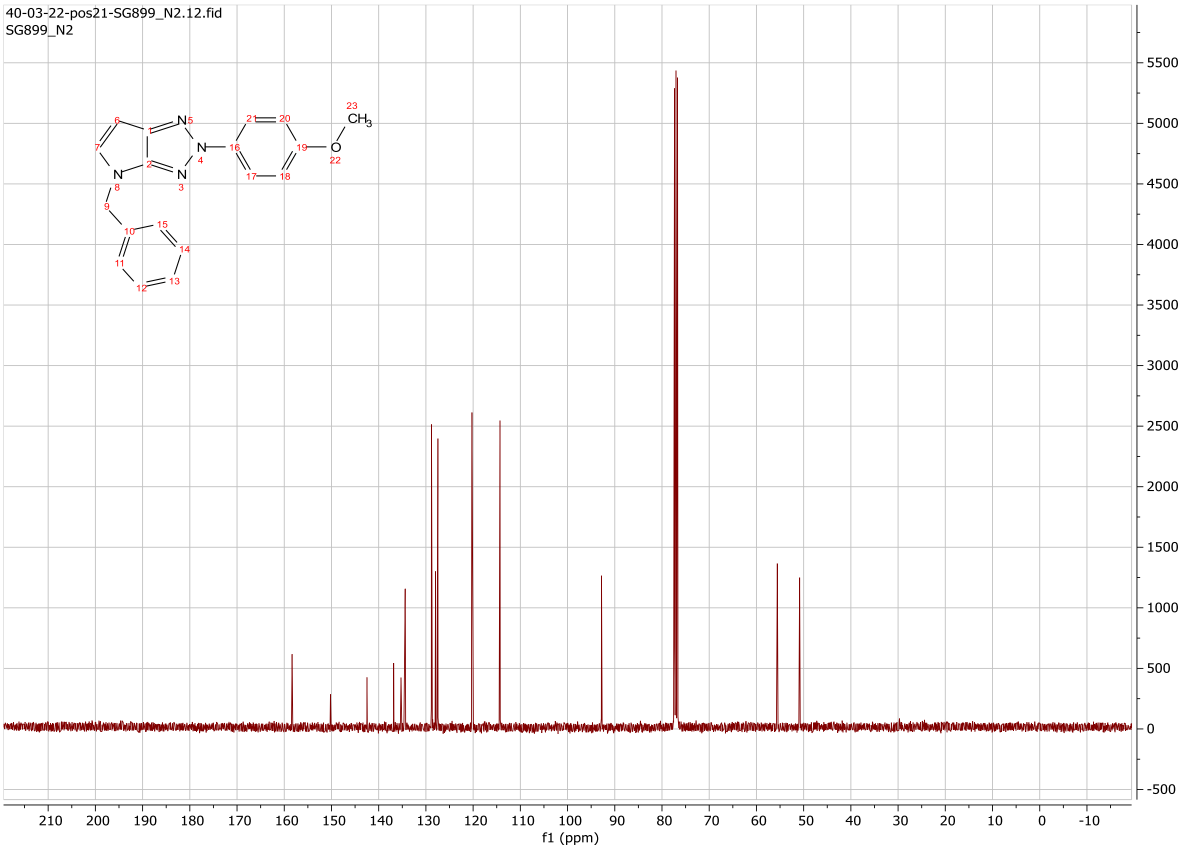
40-03-22-pos21-SG899_N2.12.fid
SG899_N2
f1 (ppm)

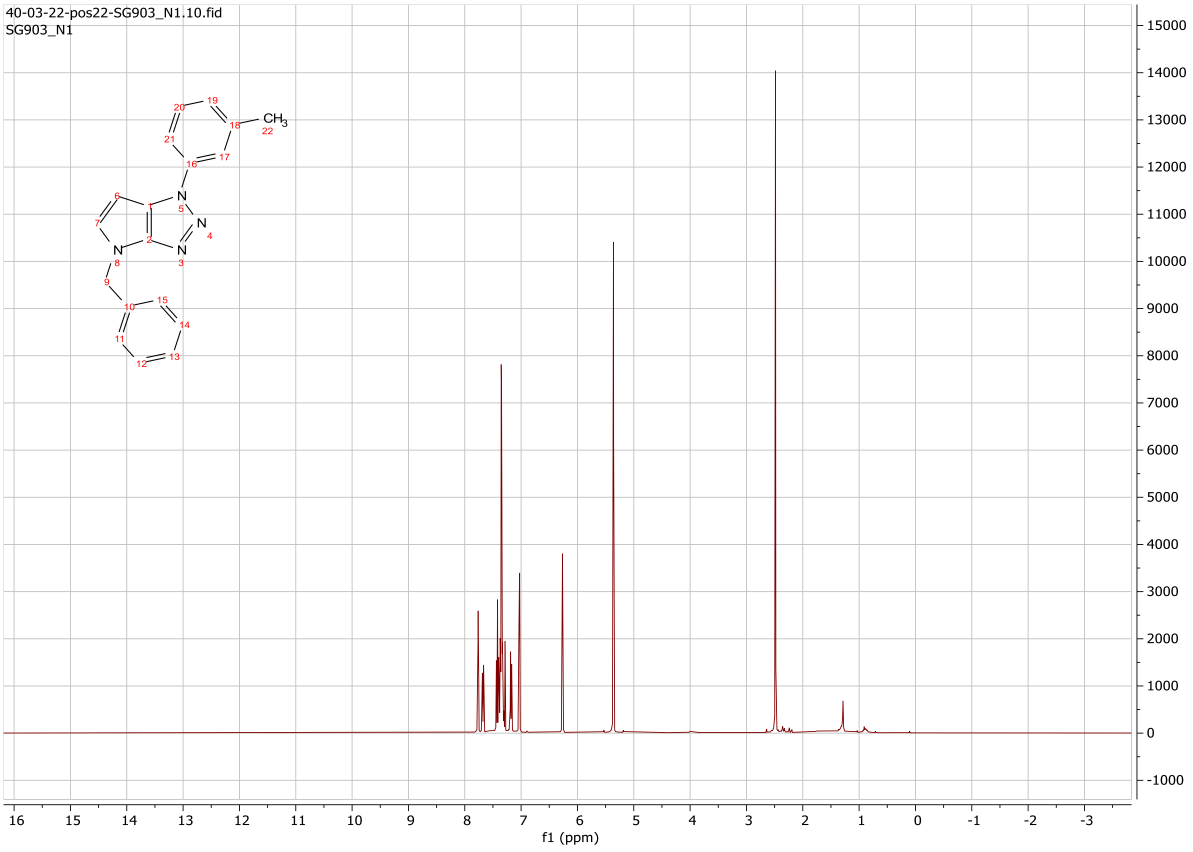
40-03-22-pos22-SG903_N1.10.fid
SG903_N1
f1 (ppm)

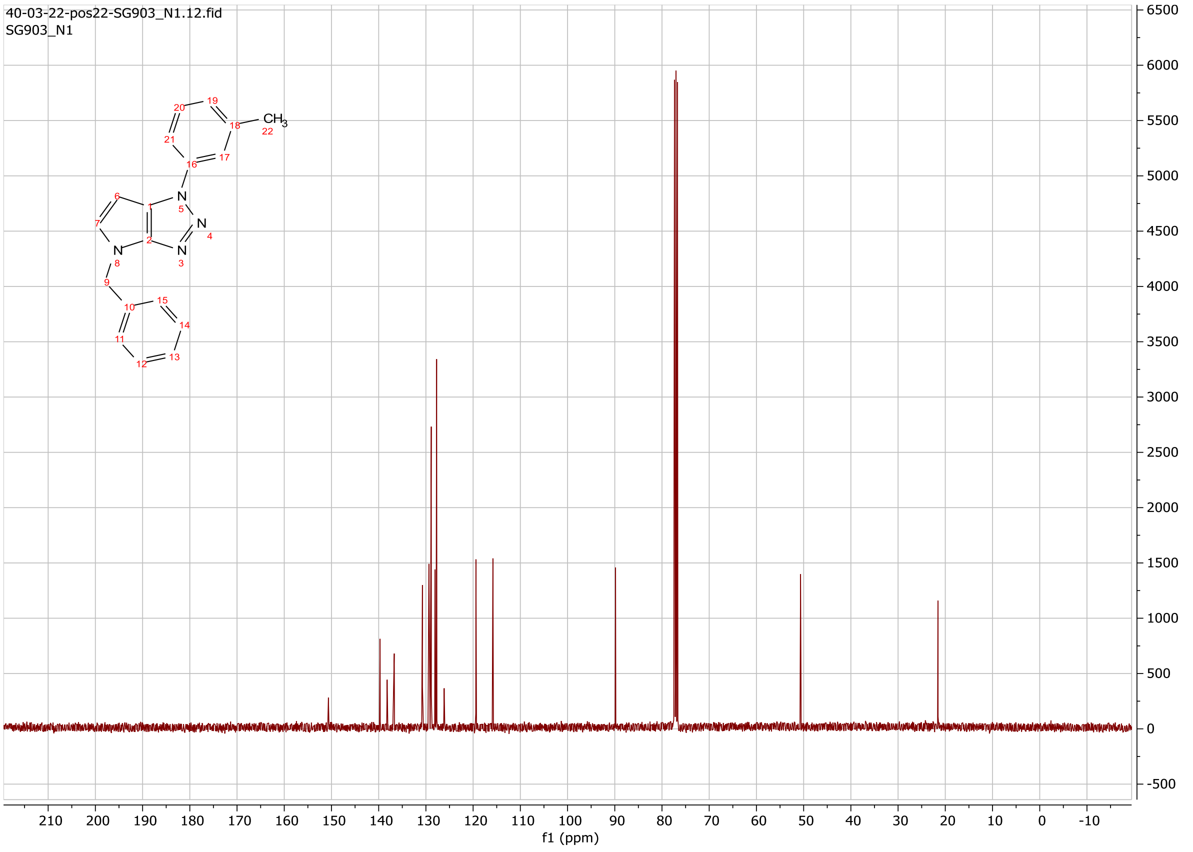
40-03-22-pos22-SG903_N1.12.fid
SG903_N1
f1 (ppm)

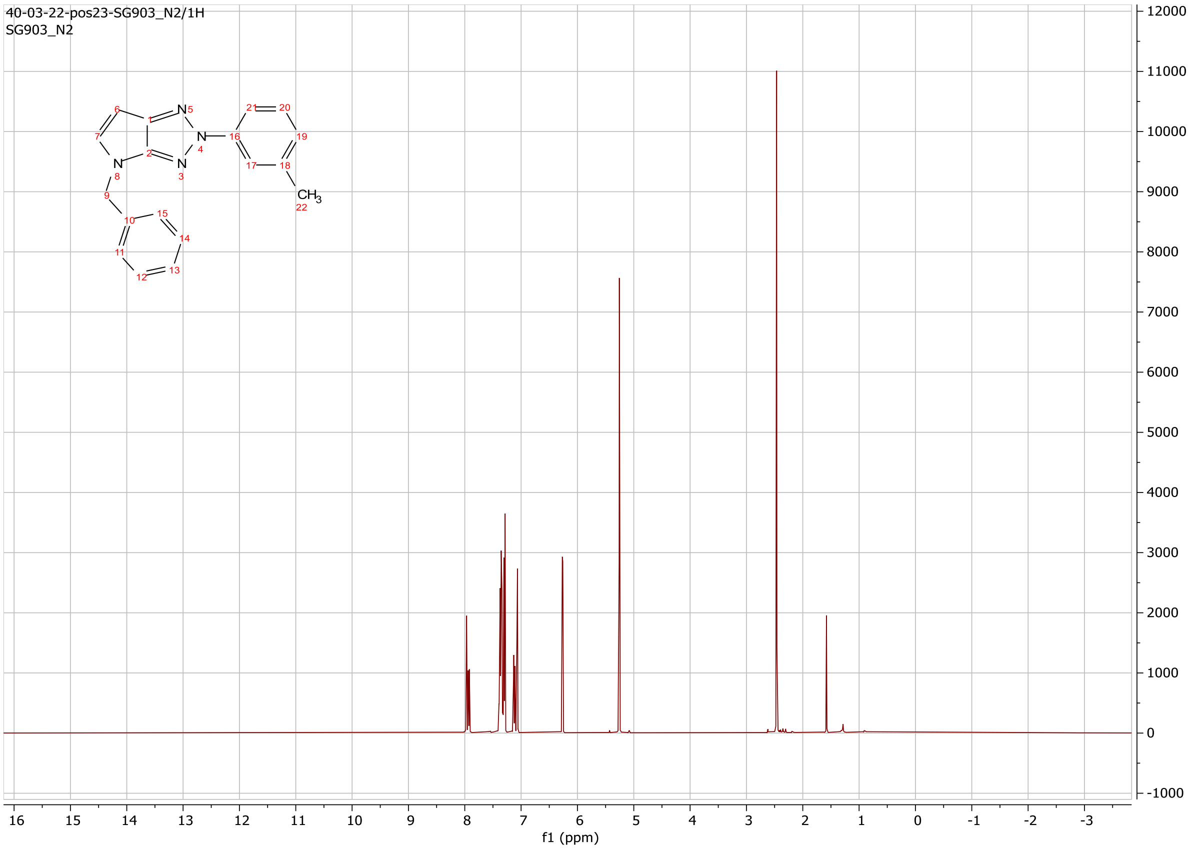
40-03-22-pos23-SG903_N2/1H
SG903_N2
f1 (ppm)

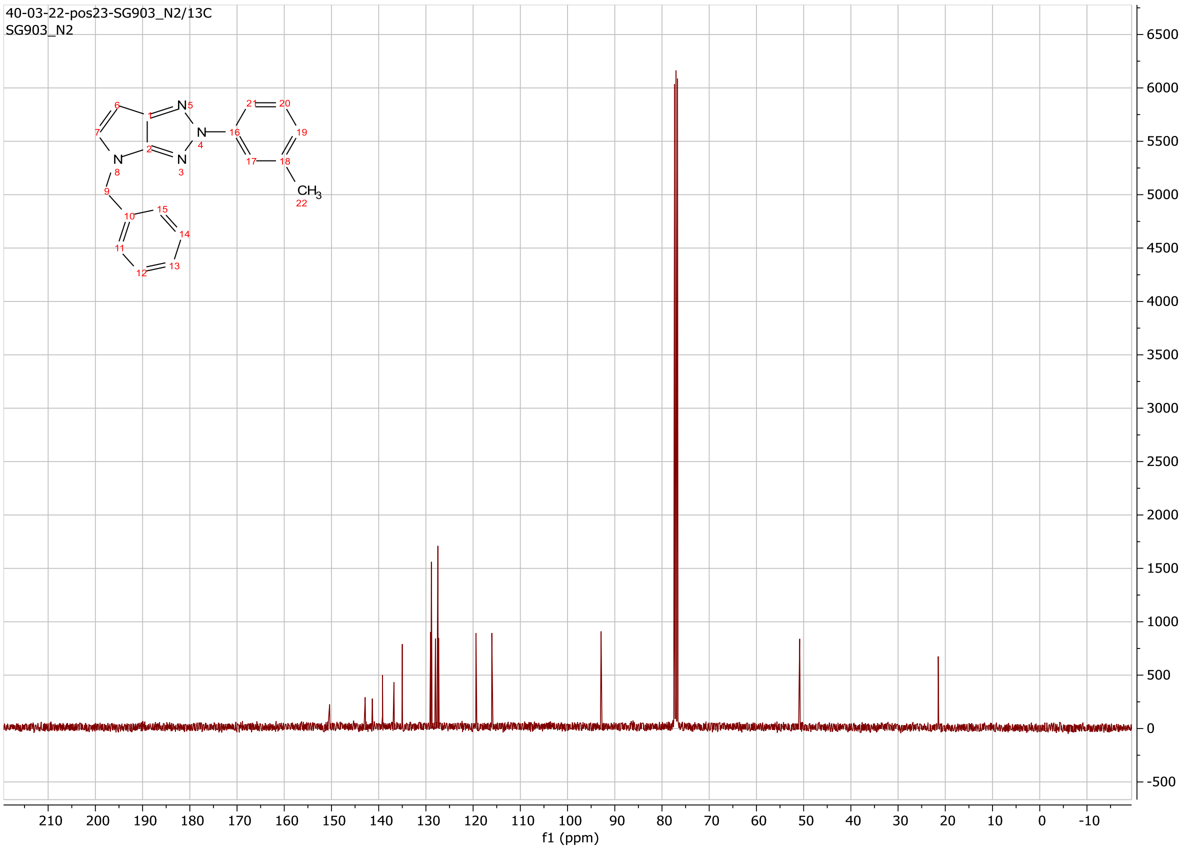
40-03-22-pos23-SG903_N2/13C
SG903_N2
f1 (ppm)

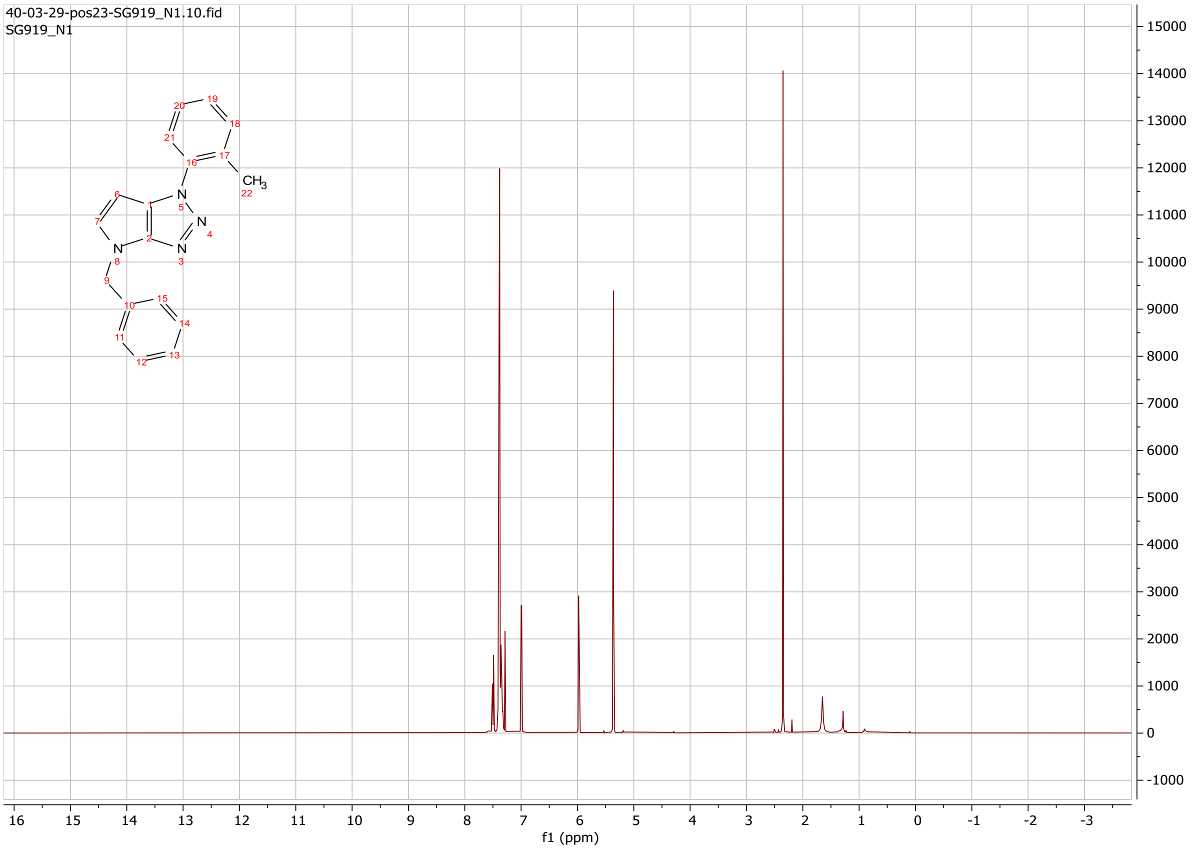
40-03-29-pos23-SG919_N1.10.fid
SG919_N1
f1 (ppm)

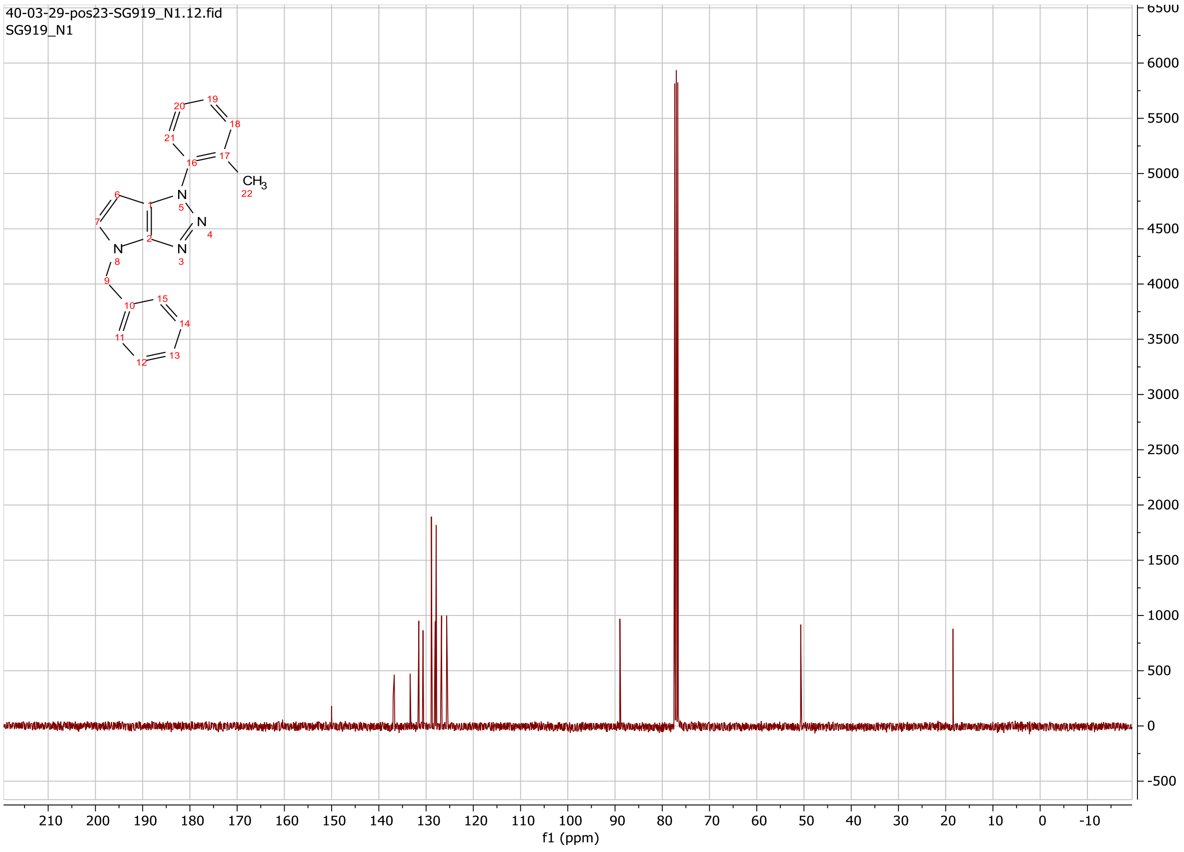
40-03-29-pos23-SG919_N1.12.fid
SG919_N1
f1 (ppm)

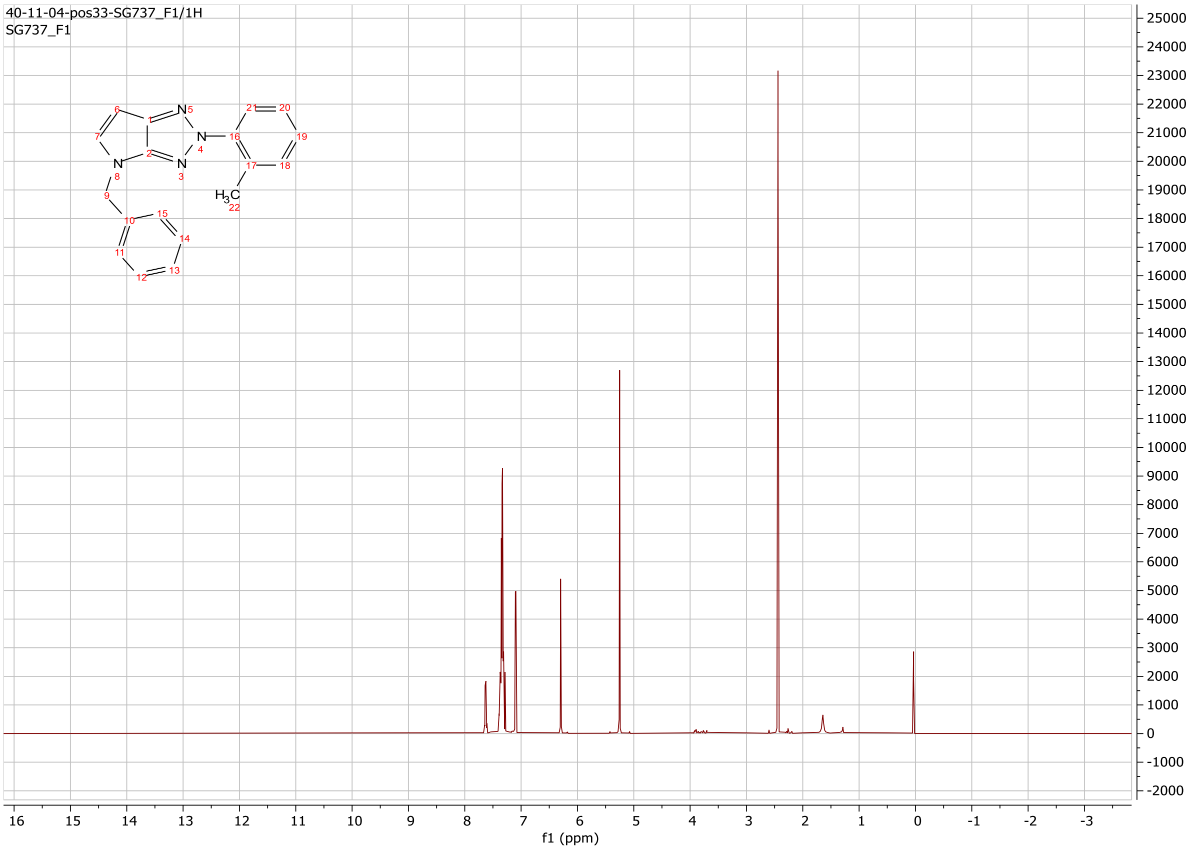
40-11-04-pos33-SG737_F1/1H
SG737_F1
f1 (ppm)

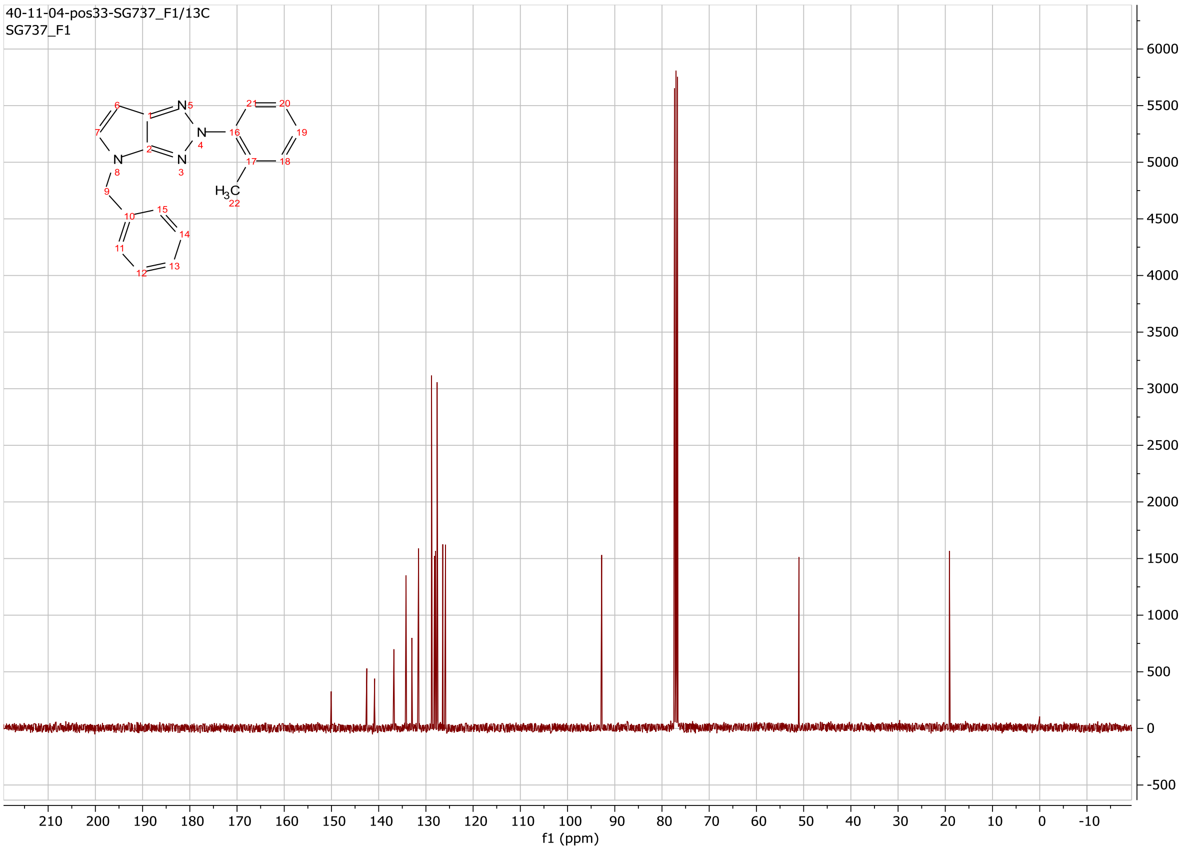
40-11-04-pos33-SG737_F1/13C
SG737_F1
f1 (ppm)

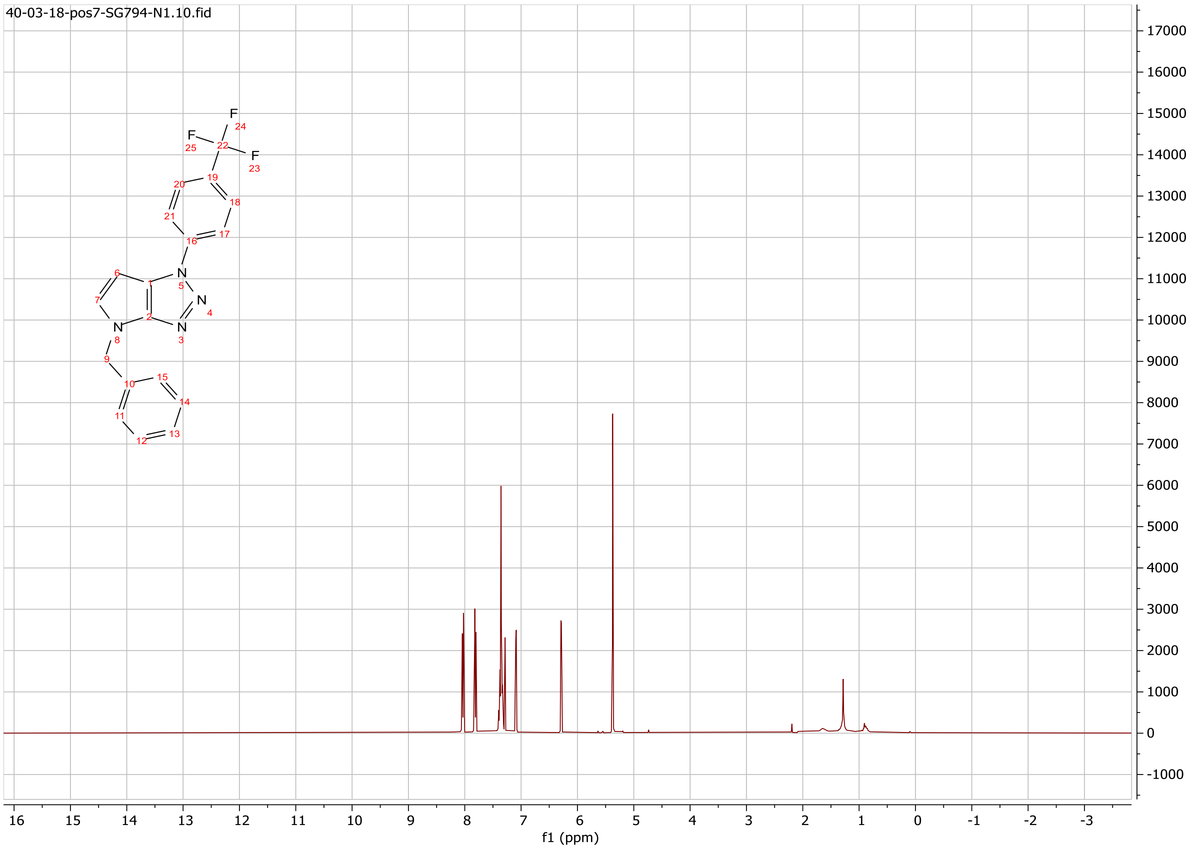
40-03-18-pos7-SG794-N1.10.fid
f1 (ppm)

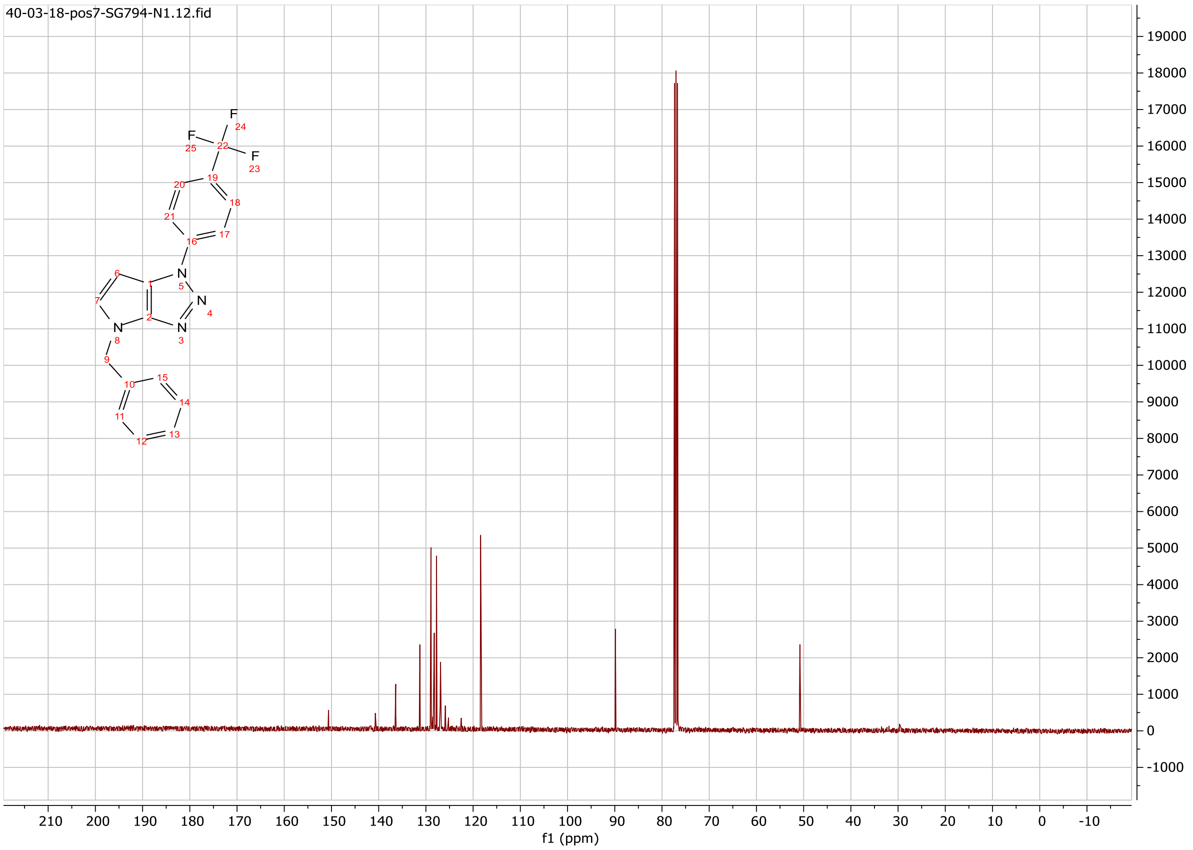
40-03-18-pos7-SG794-N1.12.fid
f1 (ppm)

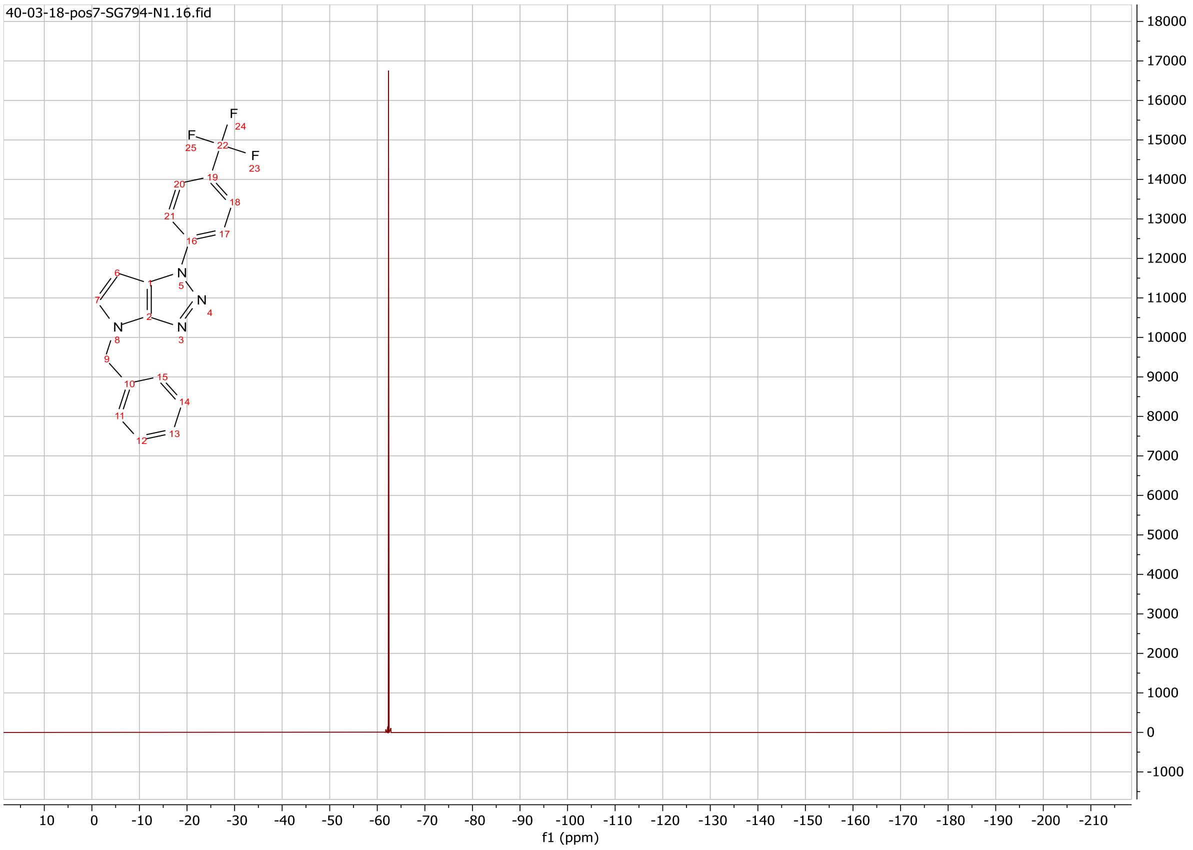
40-03-18-pos7-SG794-N1.16.fid
f1 (ppm)

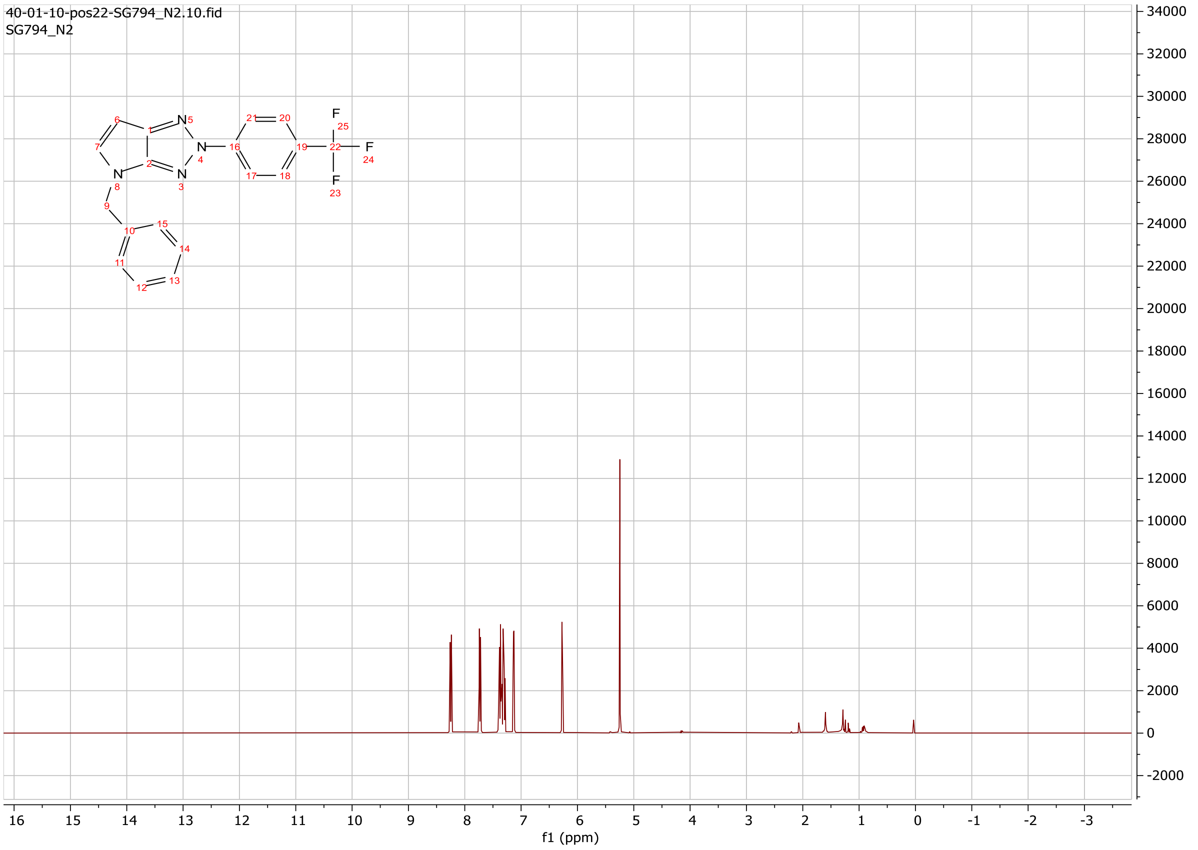
40-01-10-pos22-SG794_N2.10.fid
SG794_N2
f1 (ppm)

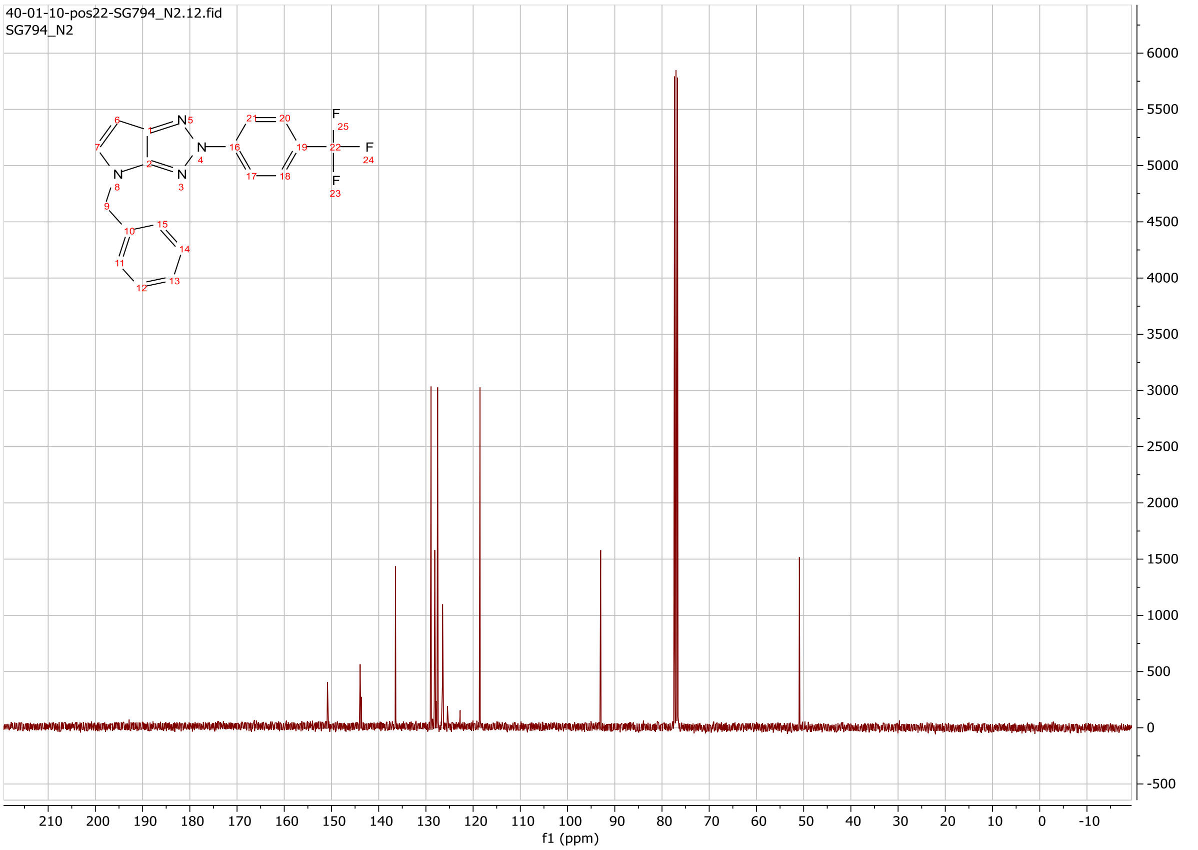
40-01-10-pos22-SG794_N2.12.fid
SG794_N2
f1 (ppm)

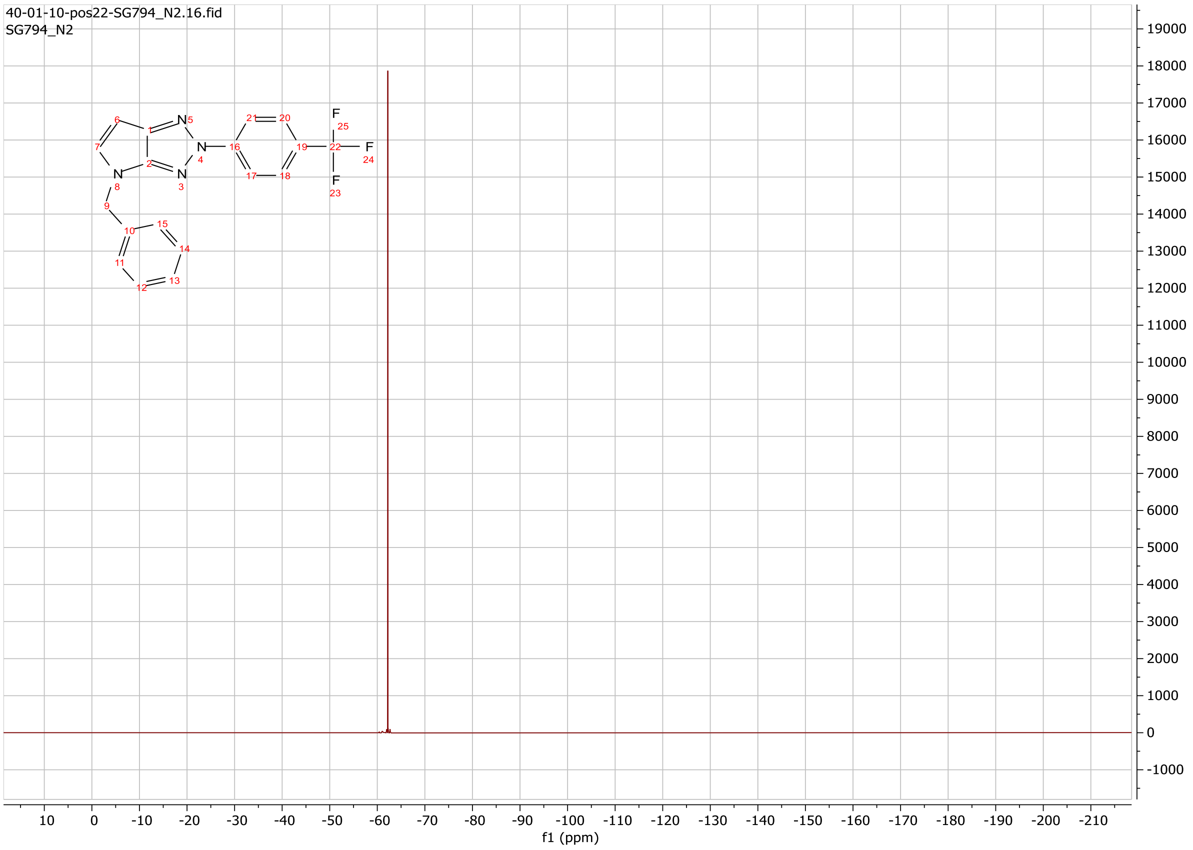

40-01-10-pos22-SG794_N2.16.fid
SG794_N2
f1 (ppm)

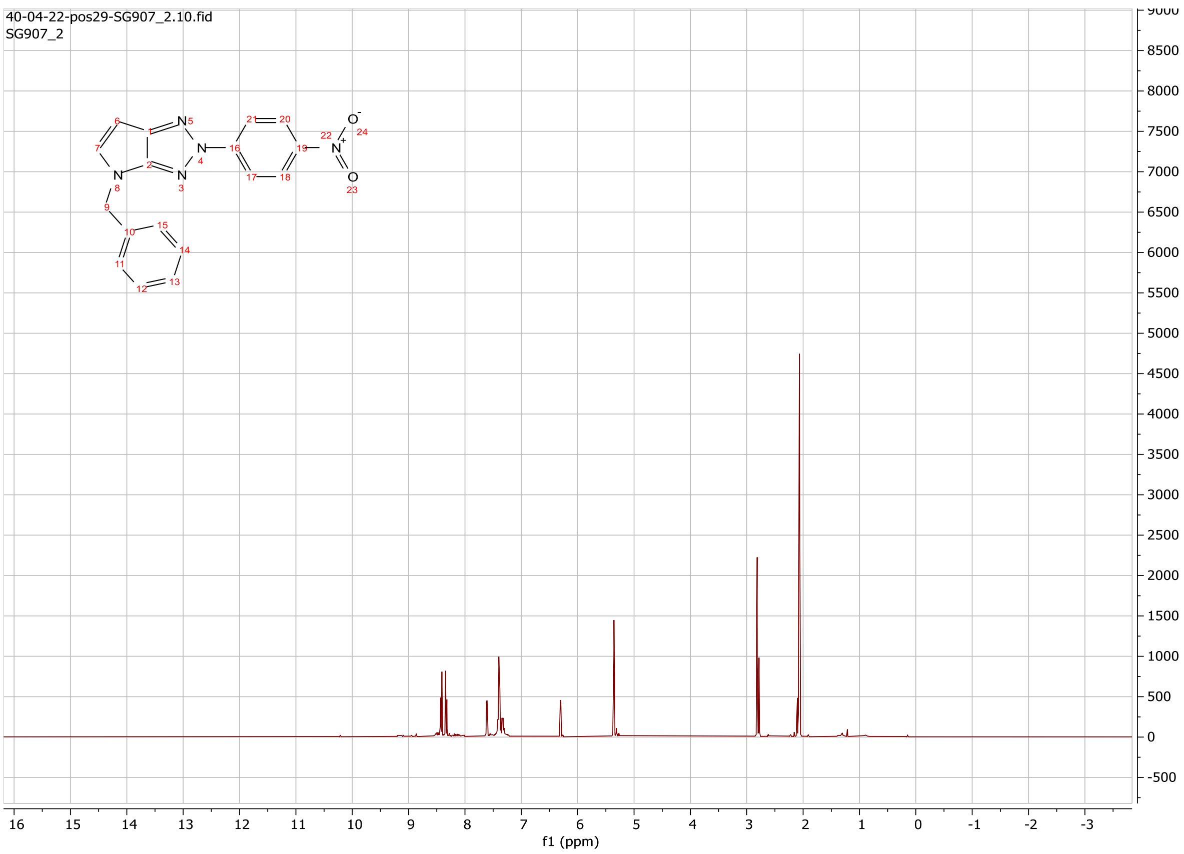

40-04-22-pos29-SG907_2.10.fid
SG907_2
f1 (ppm)

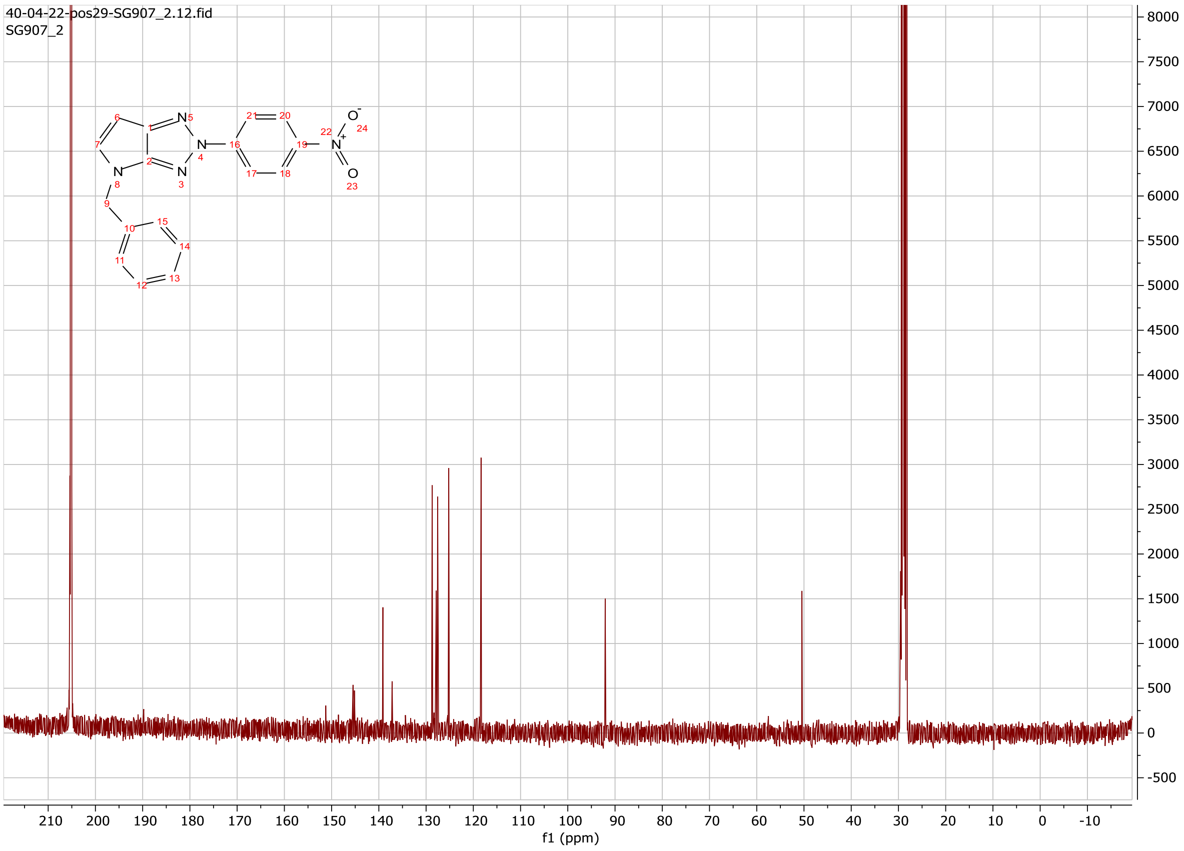

40-04-22-pos29-SG907_2.12.fid
SG907_2
f1 (ppm)

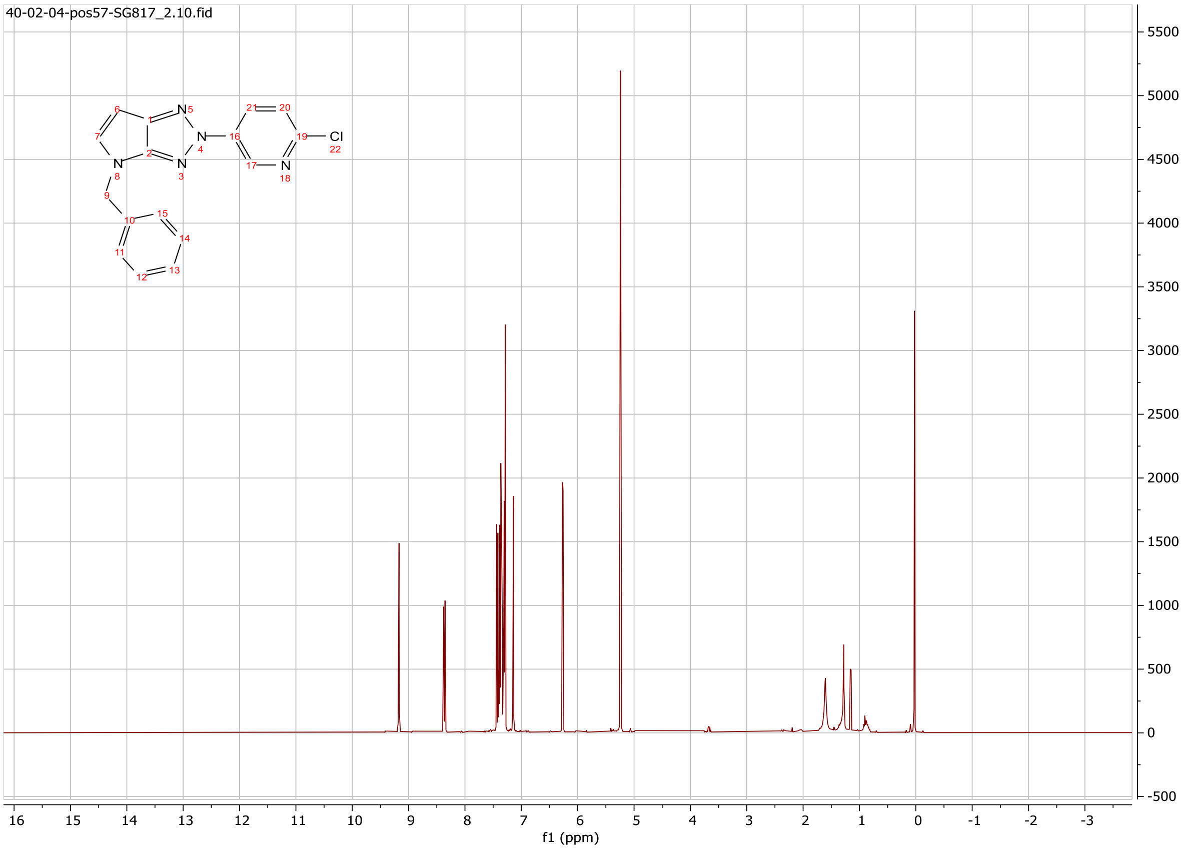
40-02-04-pos57-SG817_2.10.fid
f1 (ppm)

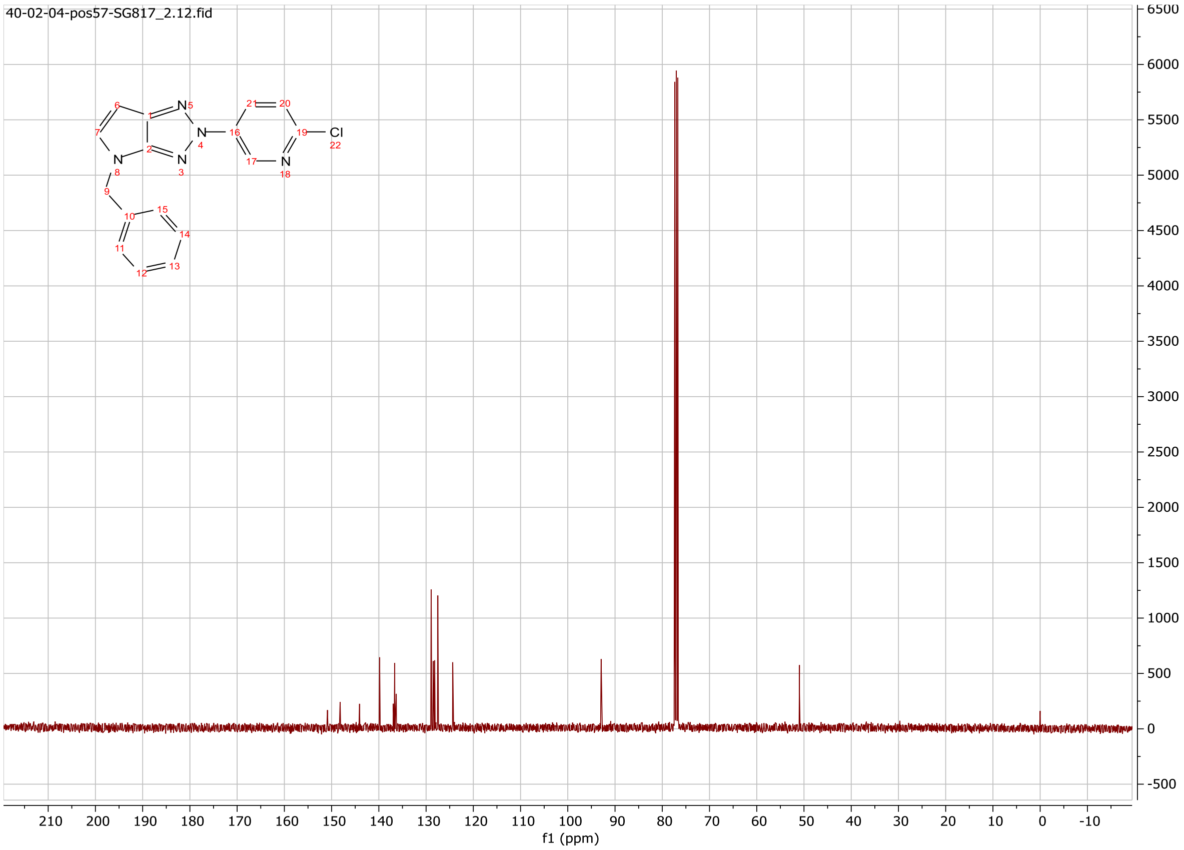
40-02-04-pos57-SG817_2.12.fid
f1 (ppm)

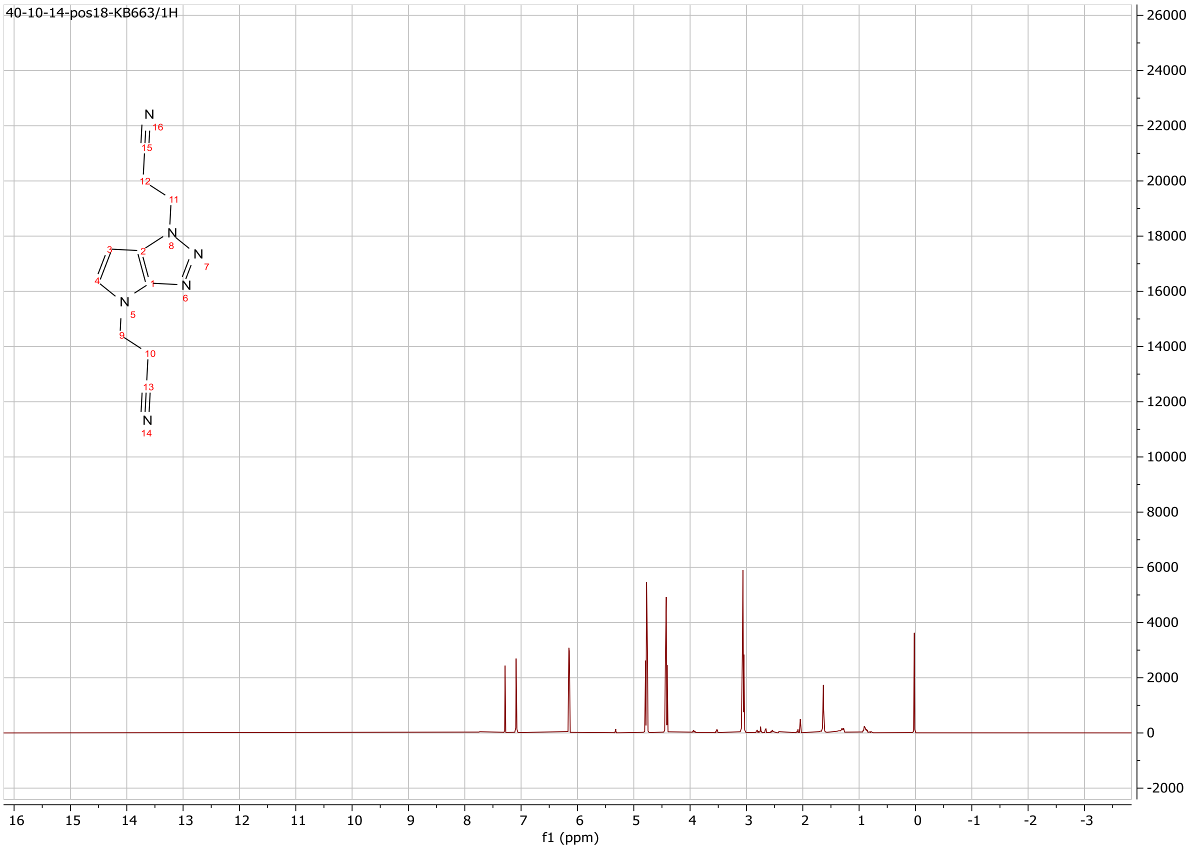
40-10-14-pos18-KB663/1H
f1 (ppm)

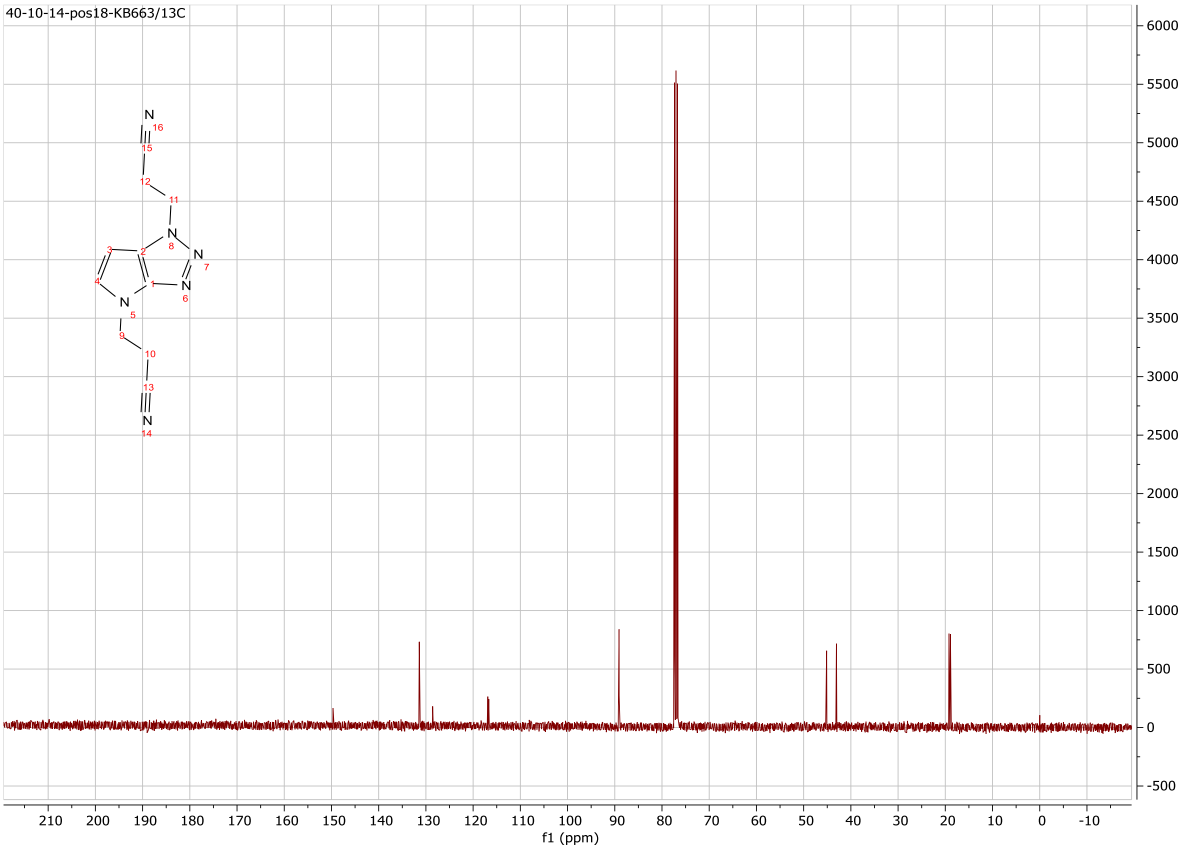
40-10-14-pos18-KB663/13C
f1 (ppm)

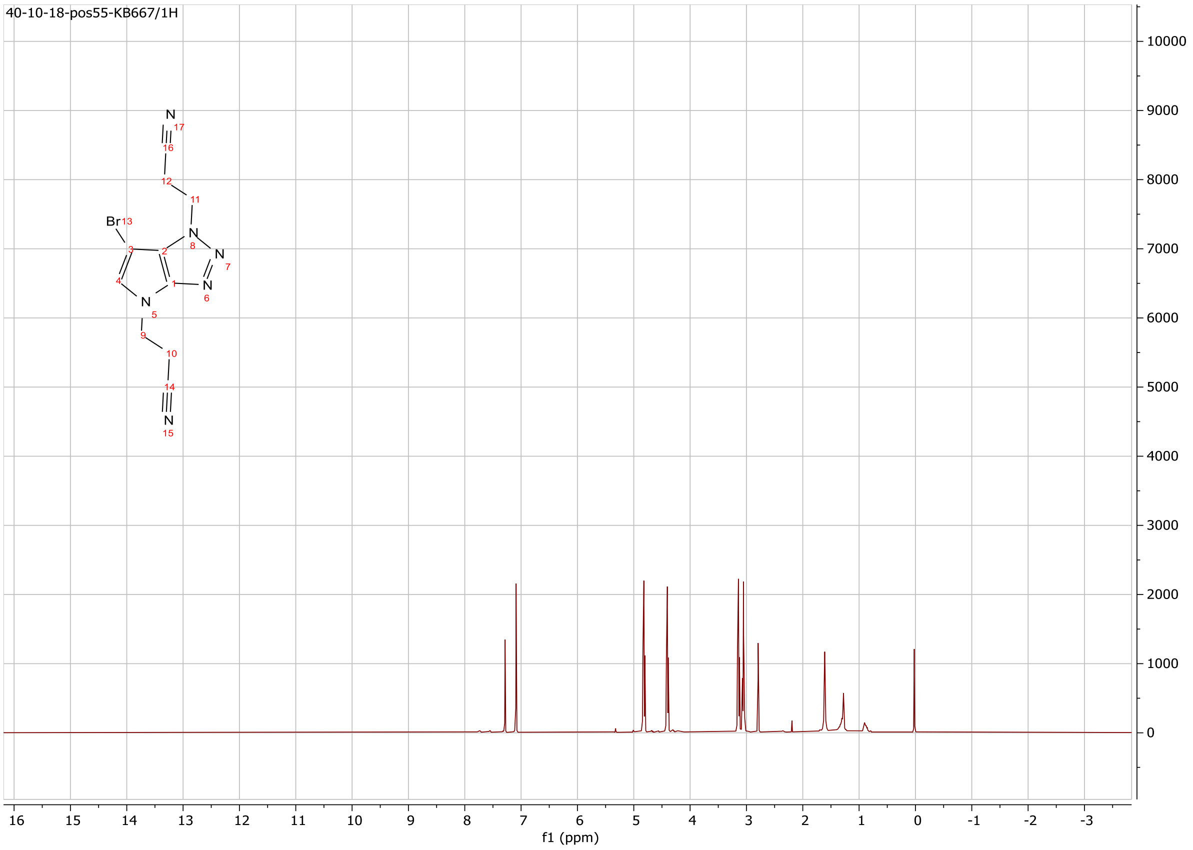
40-10-18-pos55-KB667/1H
f1 (ppm)

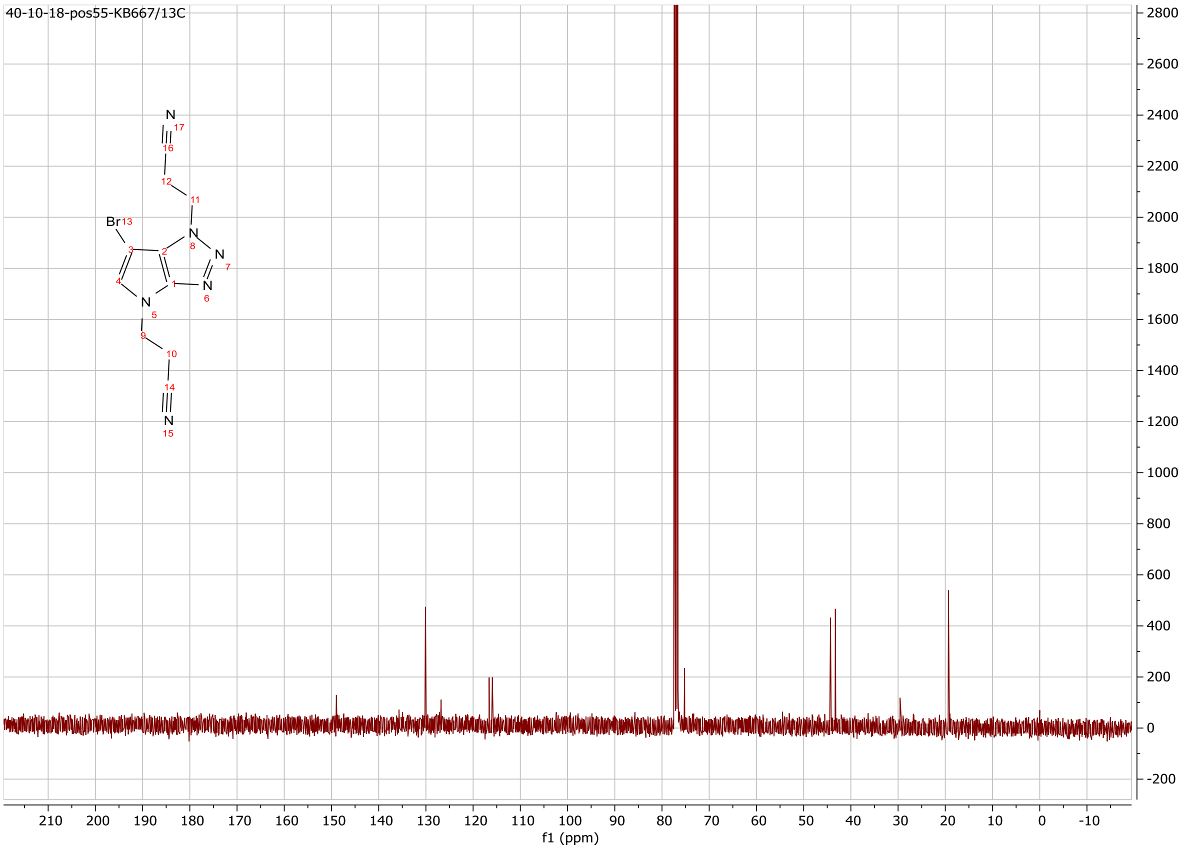
40-10-18-pos55-KB667/13C
f1 (ppm)

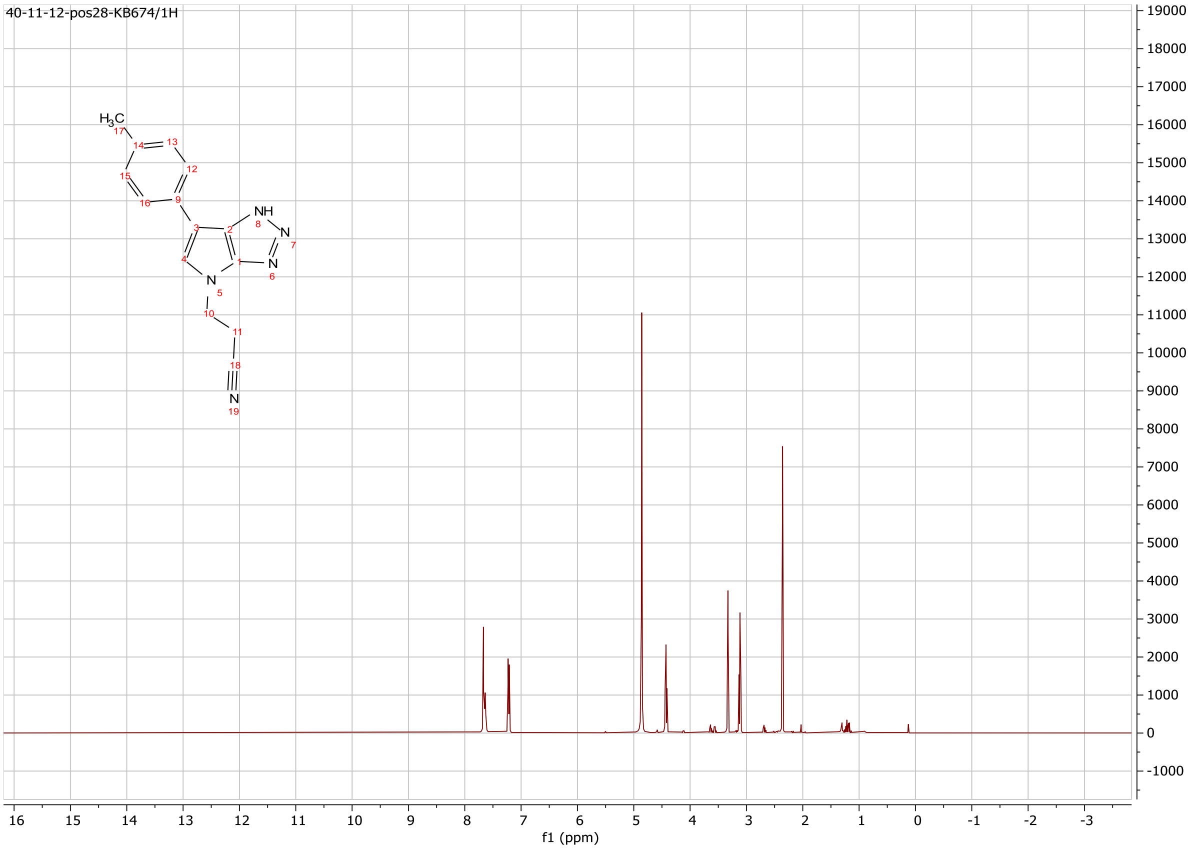
40-11-12-pos28-KB674/1H
f1 (ppm)

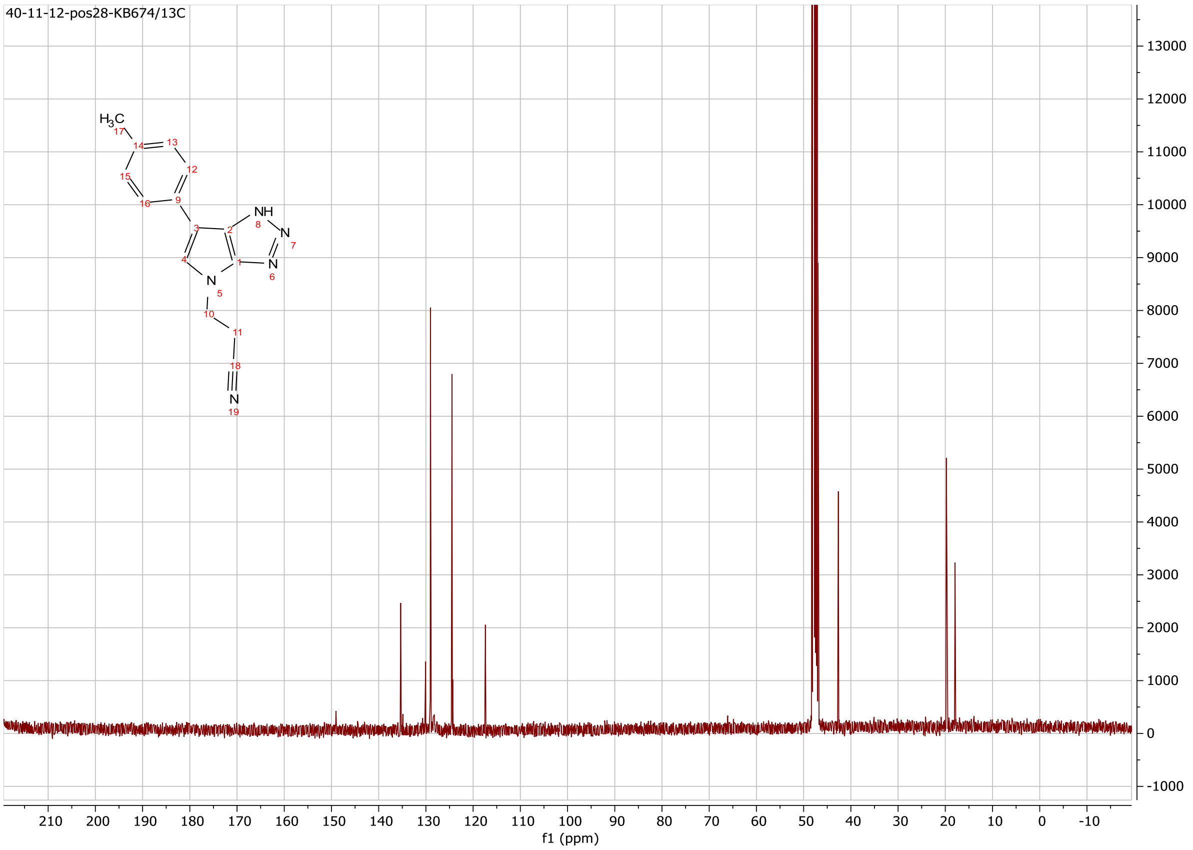
40-11-12-pos28-KB674/13C
f1 (ppm)

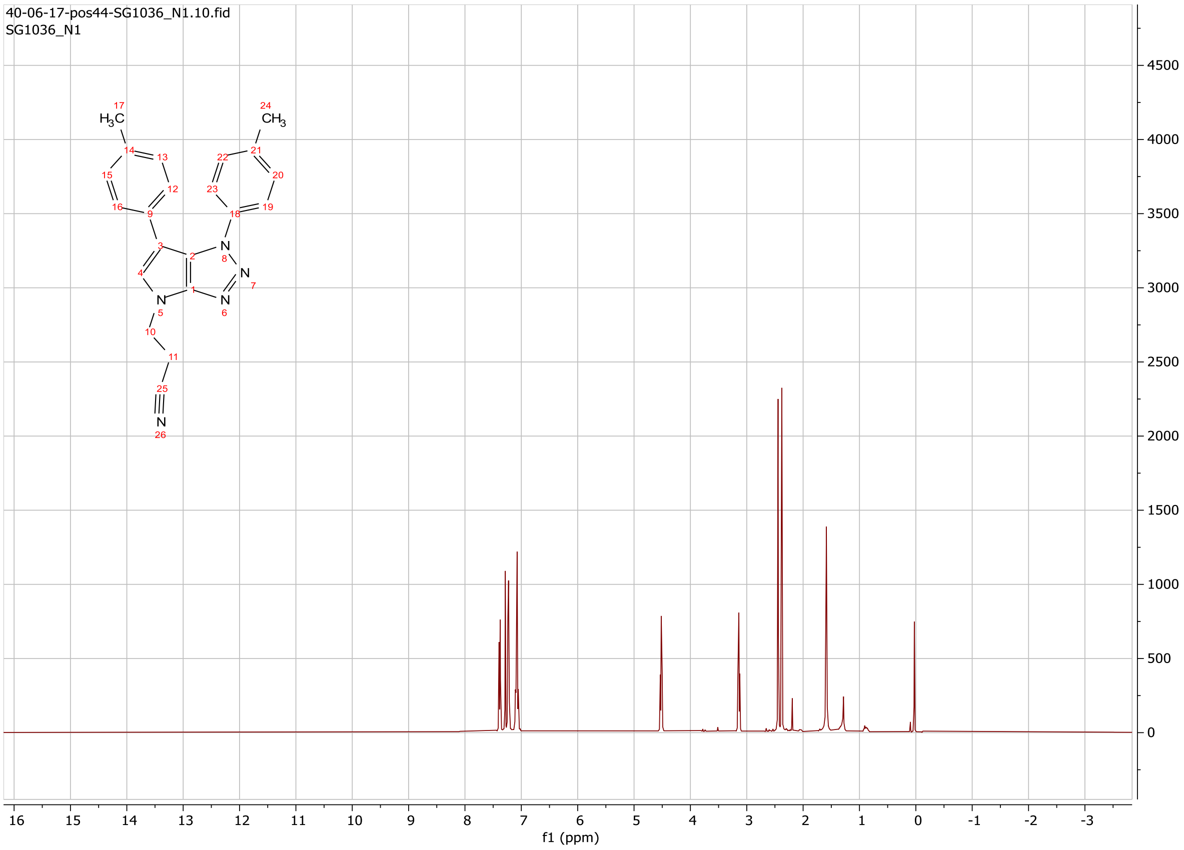
40-06-17-pos44-SG1036_N1.10.fid
SG1036_N1
f1 (ppm)

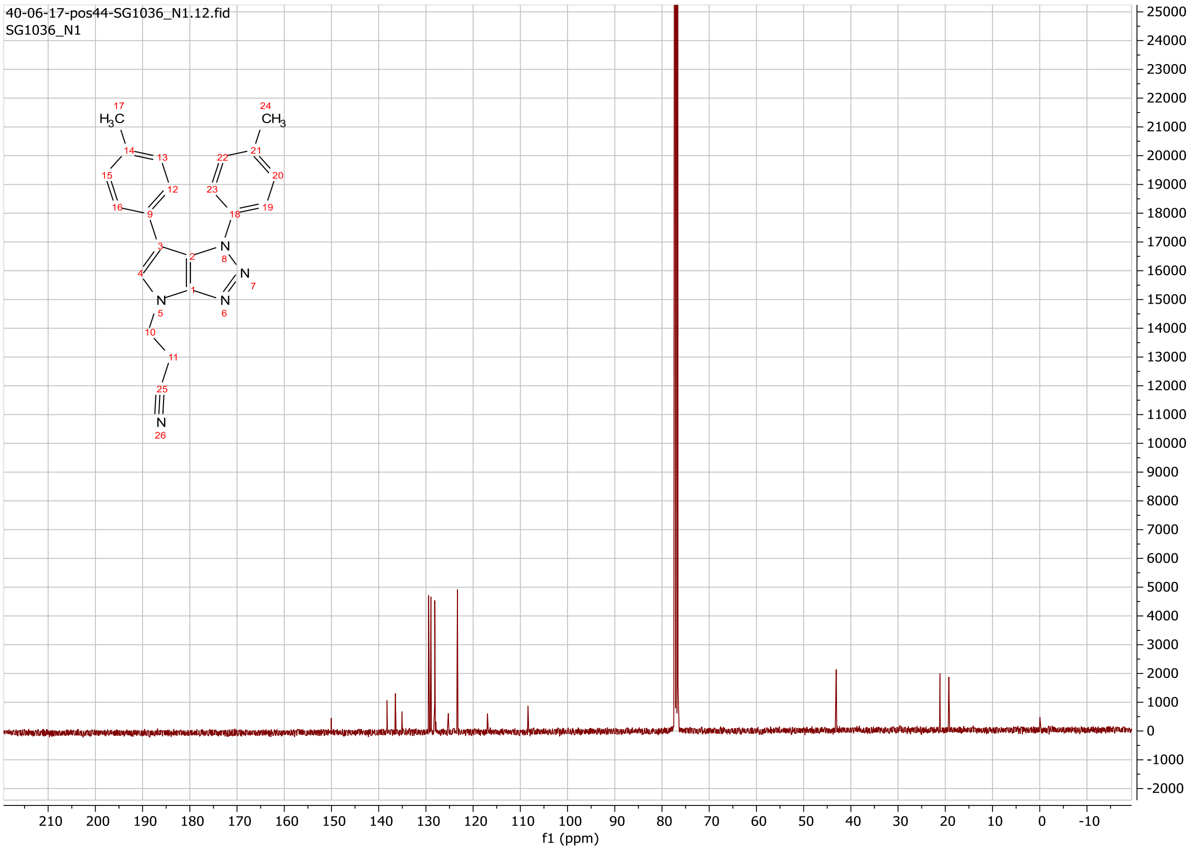
40-06-17-pos44-SG1036_N1.12.fid
SG1036_N1
f1 (ppm)

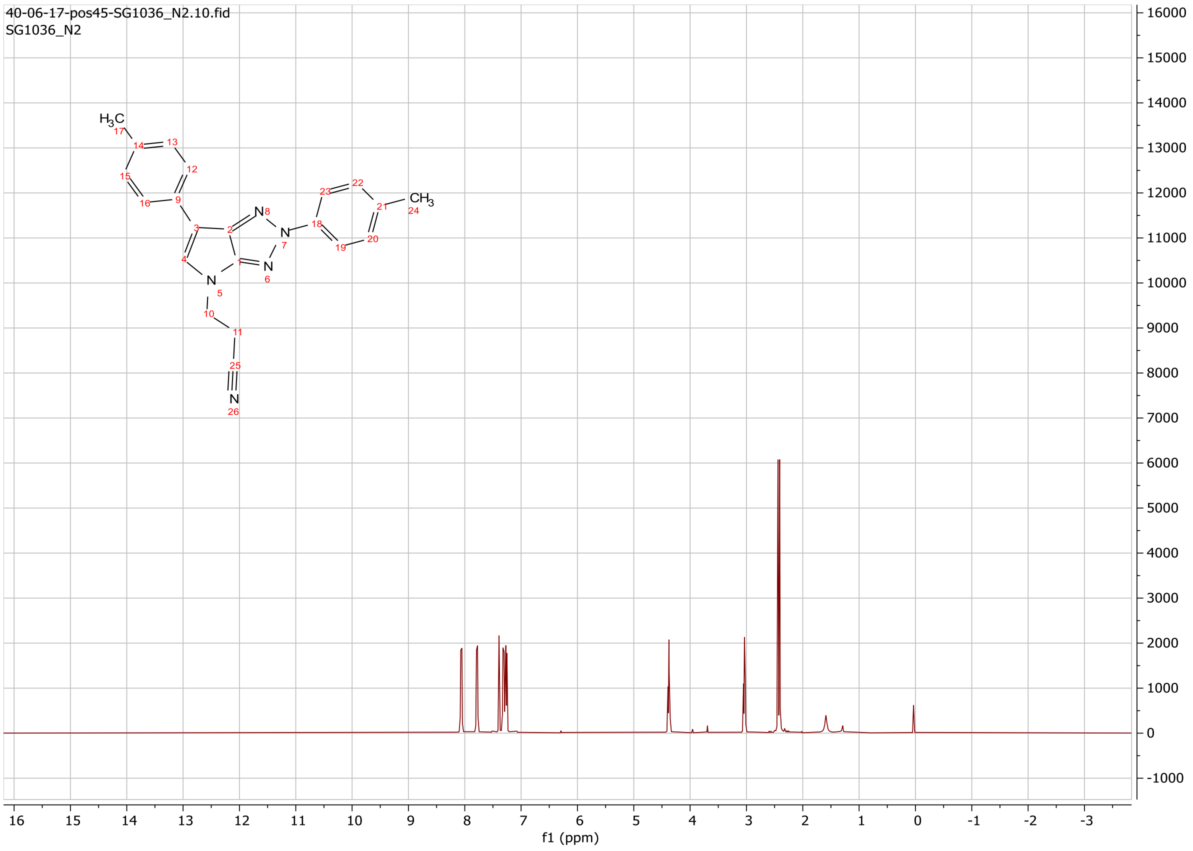
40-06-17-pos45-SG1036_N2.10.fid
SG1036_N2
f1 (ppm)

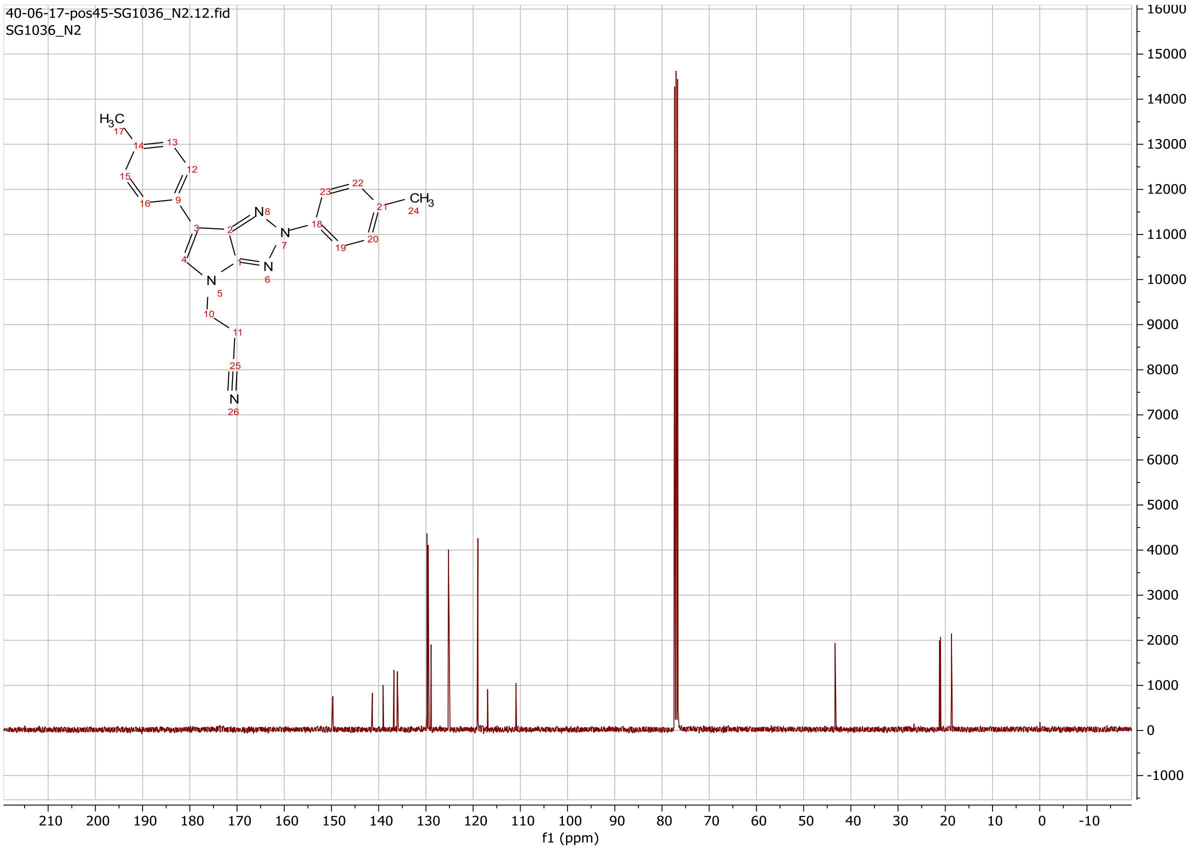
40-06-17-pos45-SG1036_N2.12.fid
SG1036_N2
f1 (ppm)

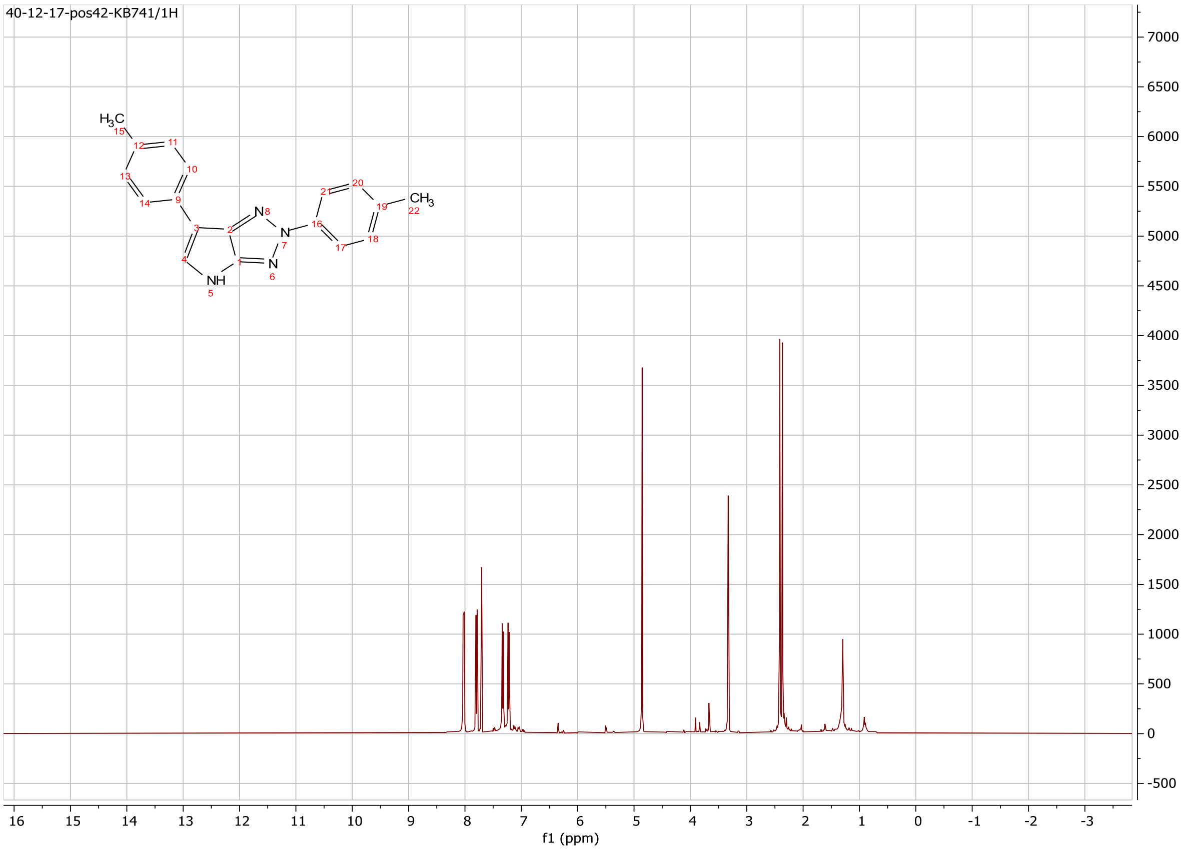
40-12-17-pos42-KB741/1H
f1 (ppm)

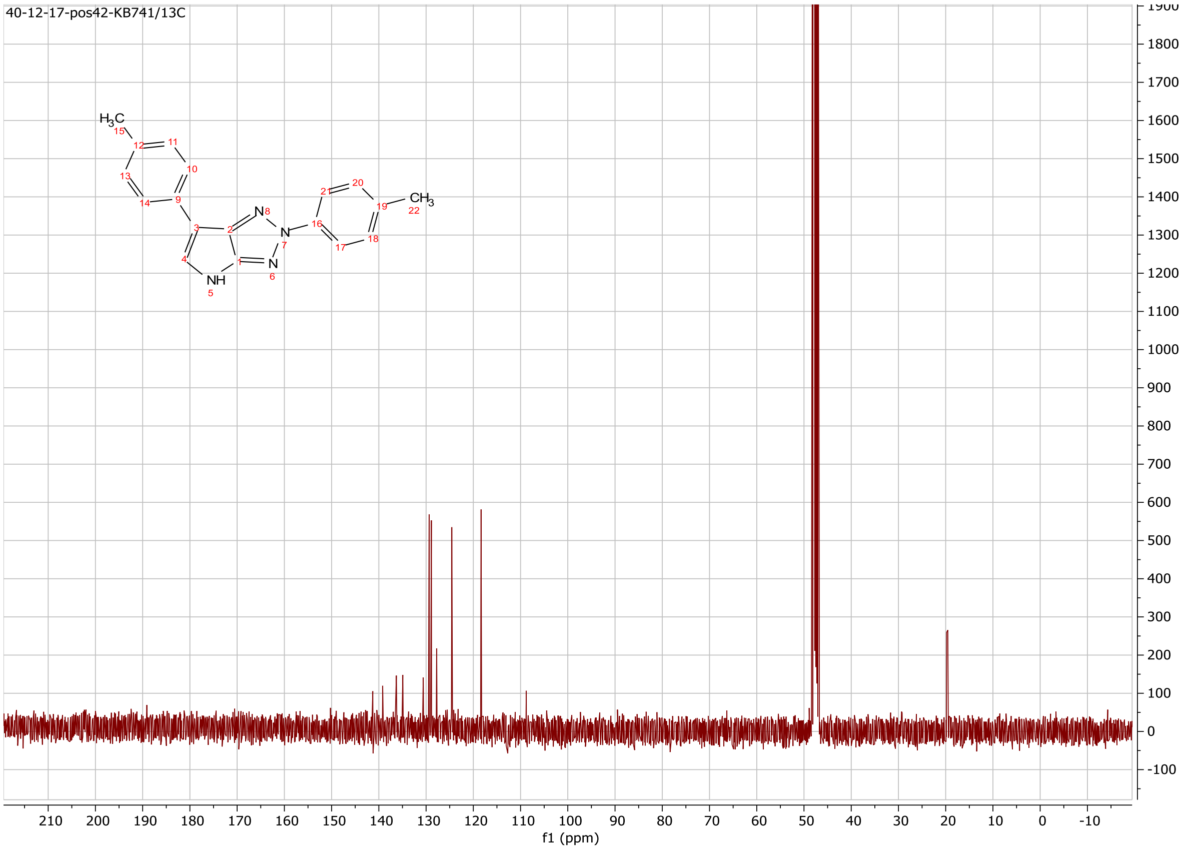
40-12-17-pos42-KB741/13C
f1 (ppm)

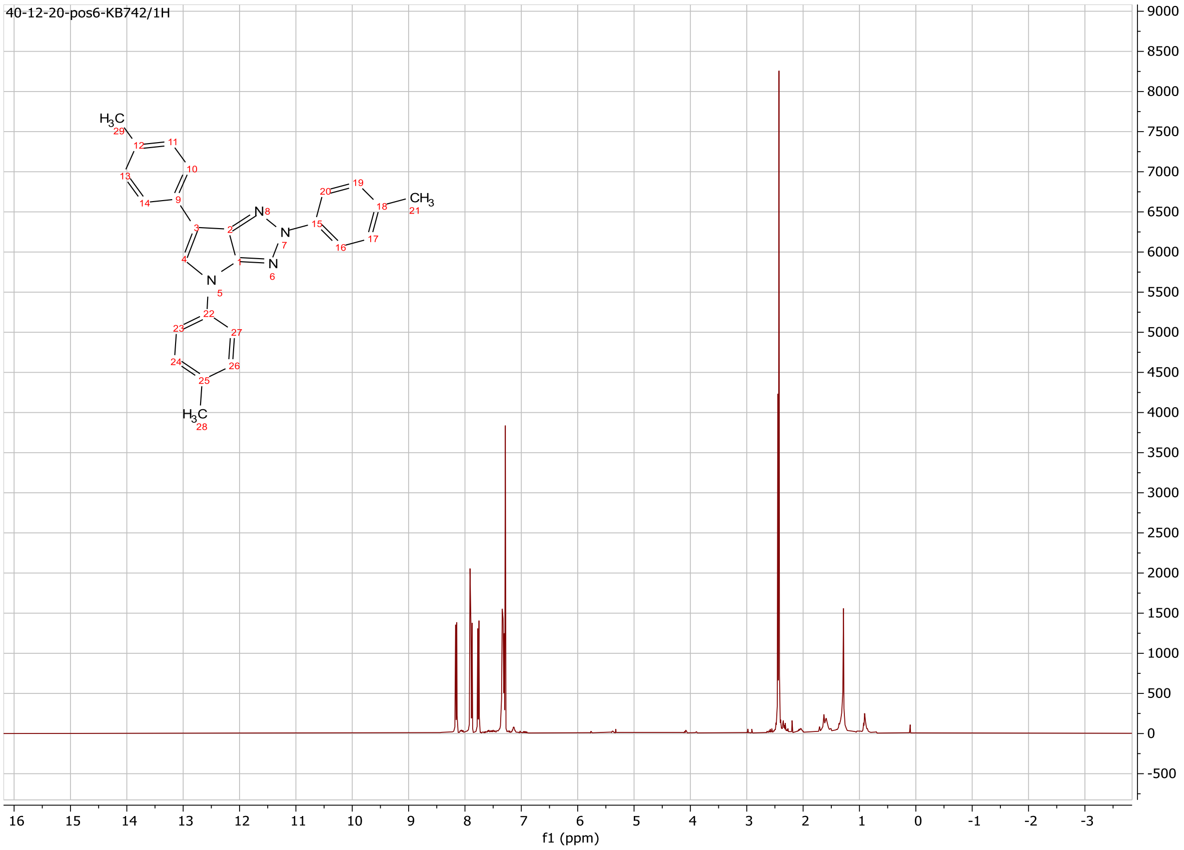
40-12-20-pos6-KB742/1H
f1 (ppm)

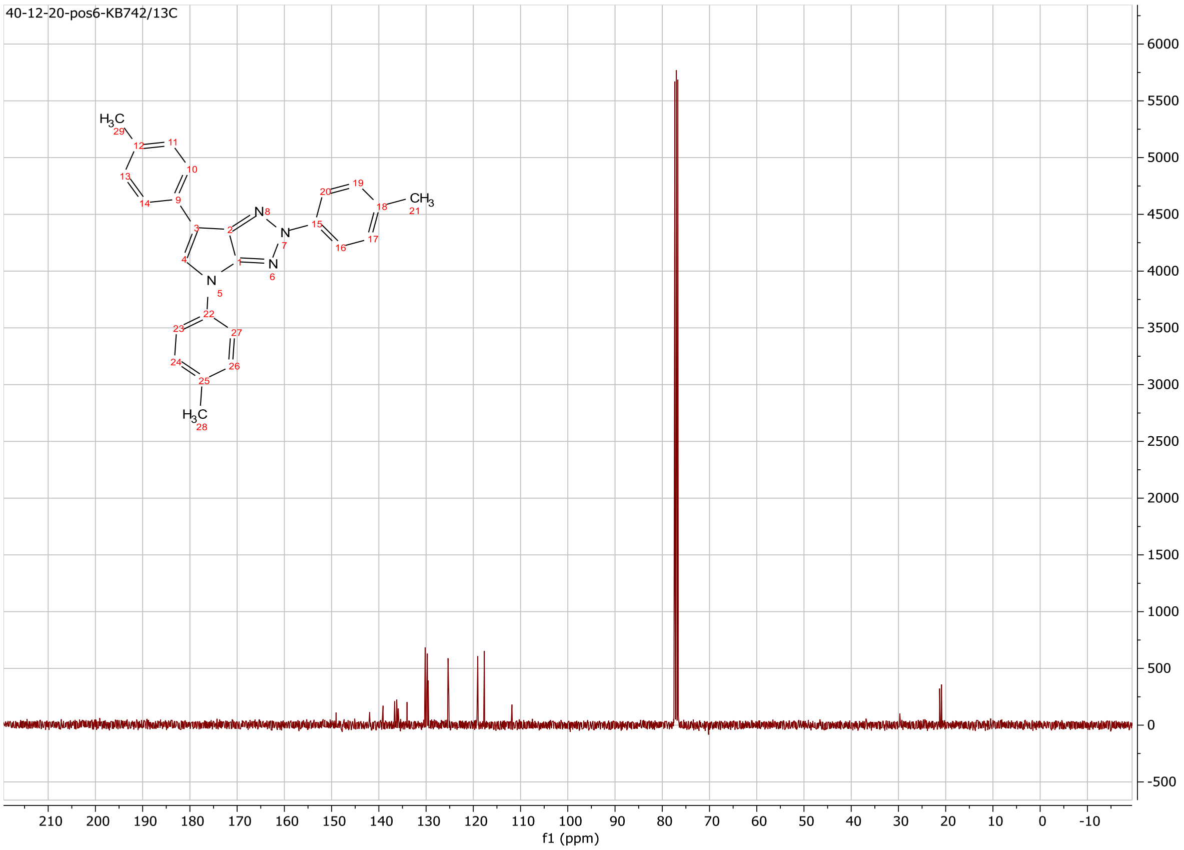
40-12-20-pos6-KB742/13C
f1 (ppm)

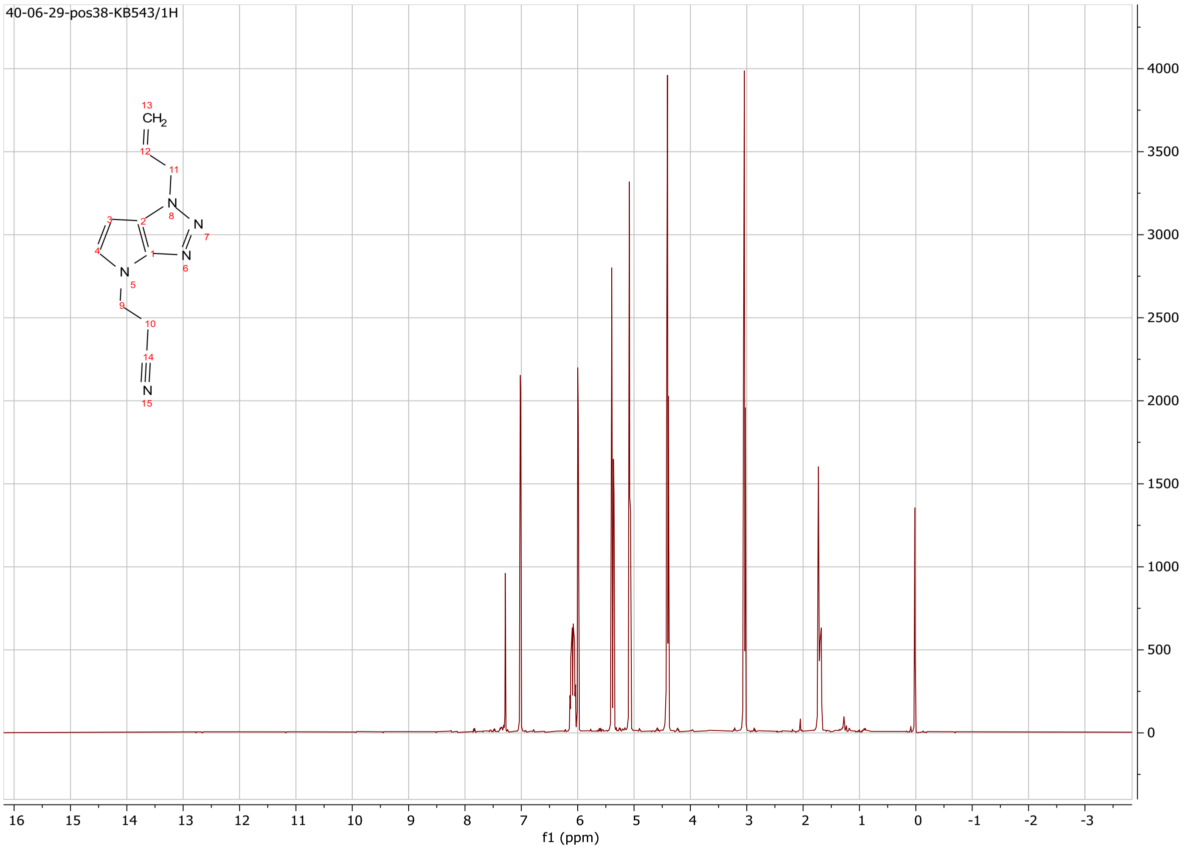
40-06-29-pos38-KB543/1H
f1 (ppm)

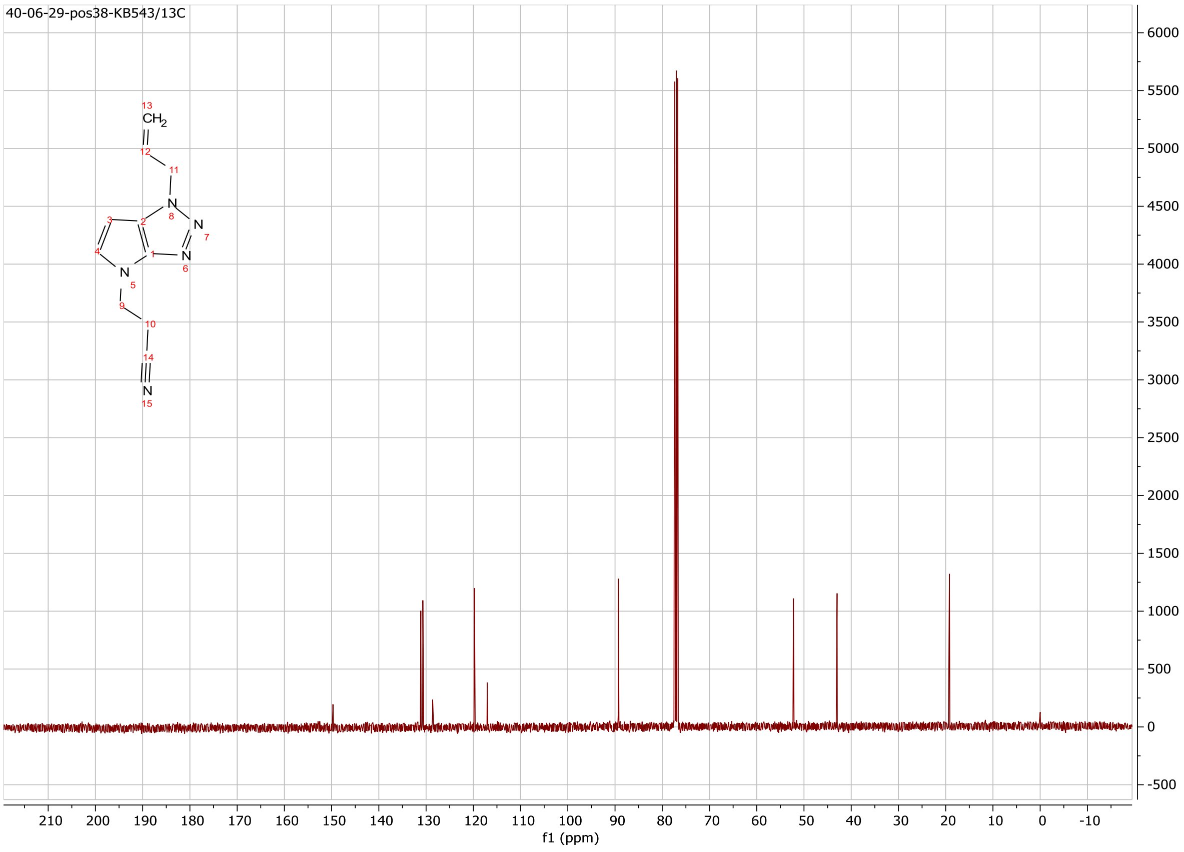
40-06-29-pos38-KB543/13C
f1 (ppm)

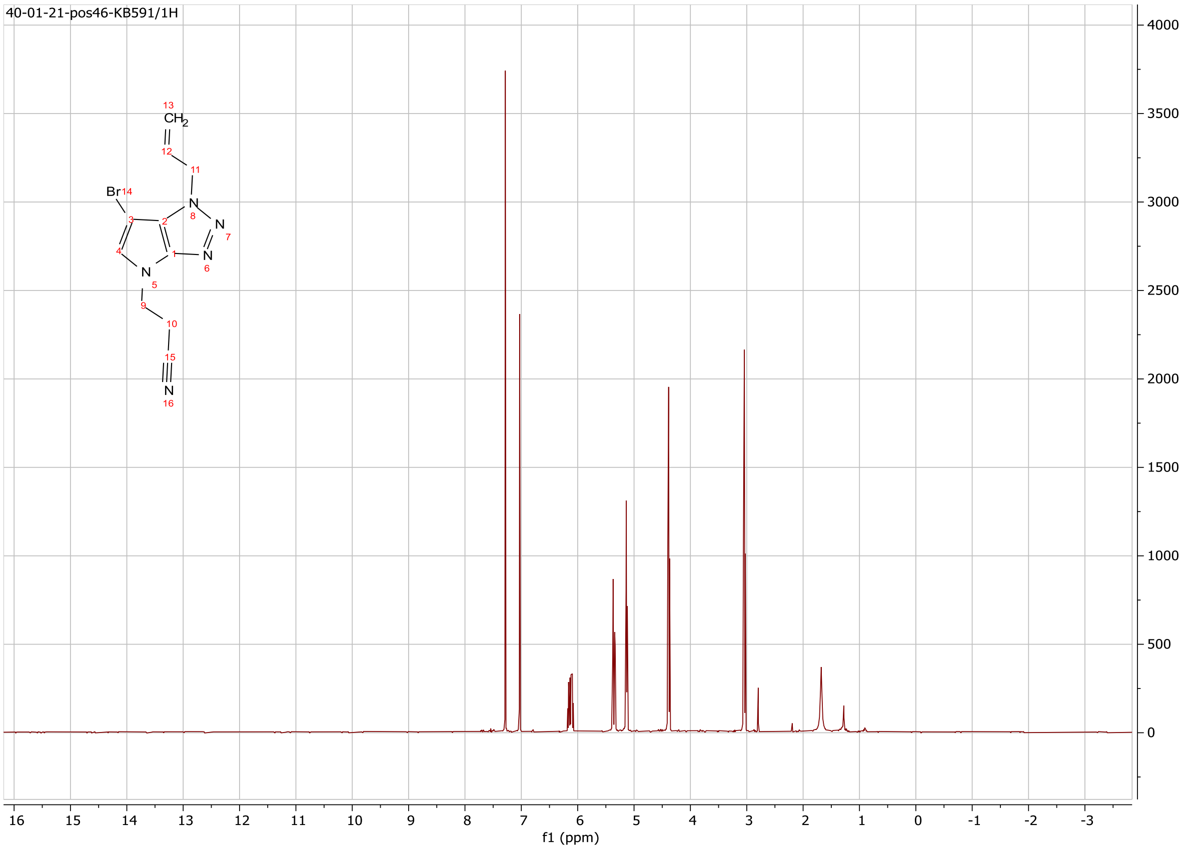
40-01-21-pos46-KB591/1H
f1 (ppm)

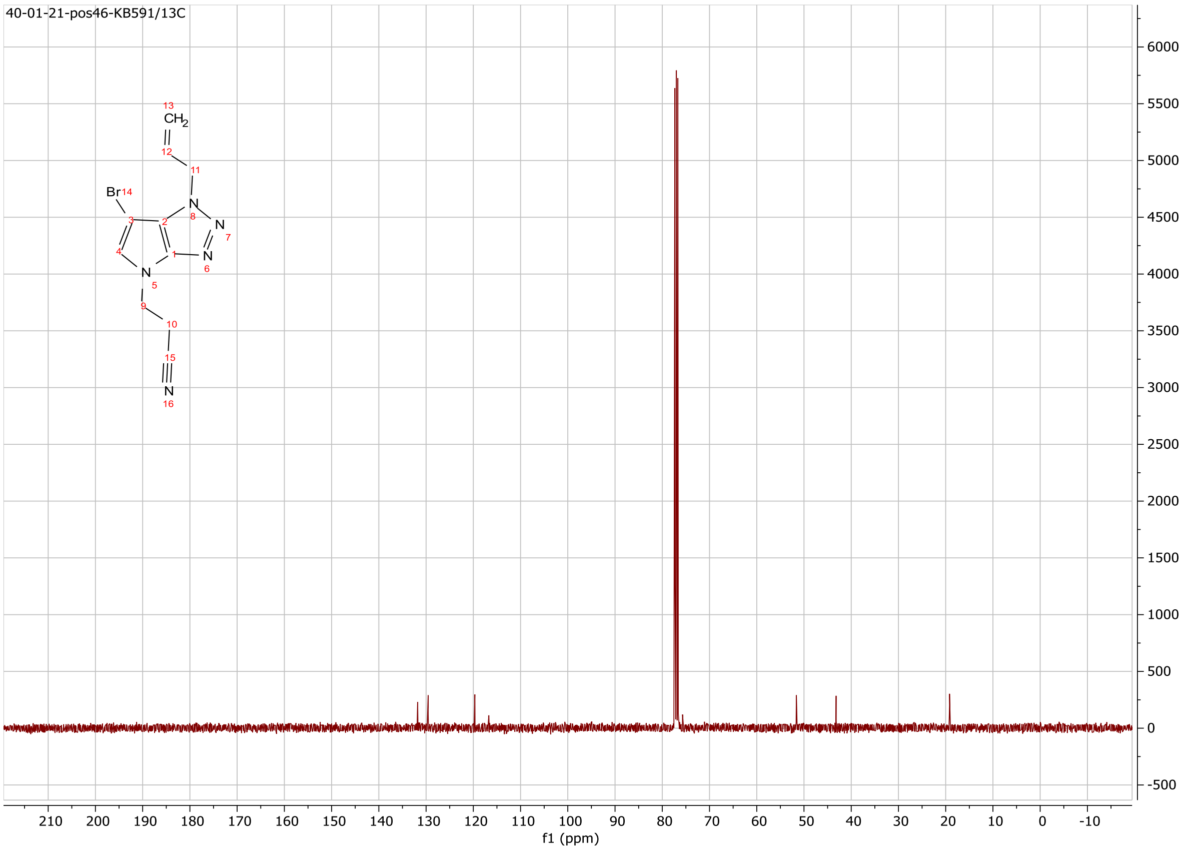
40-01-21-pos46-KB591/13C
f1 (ppm)

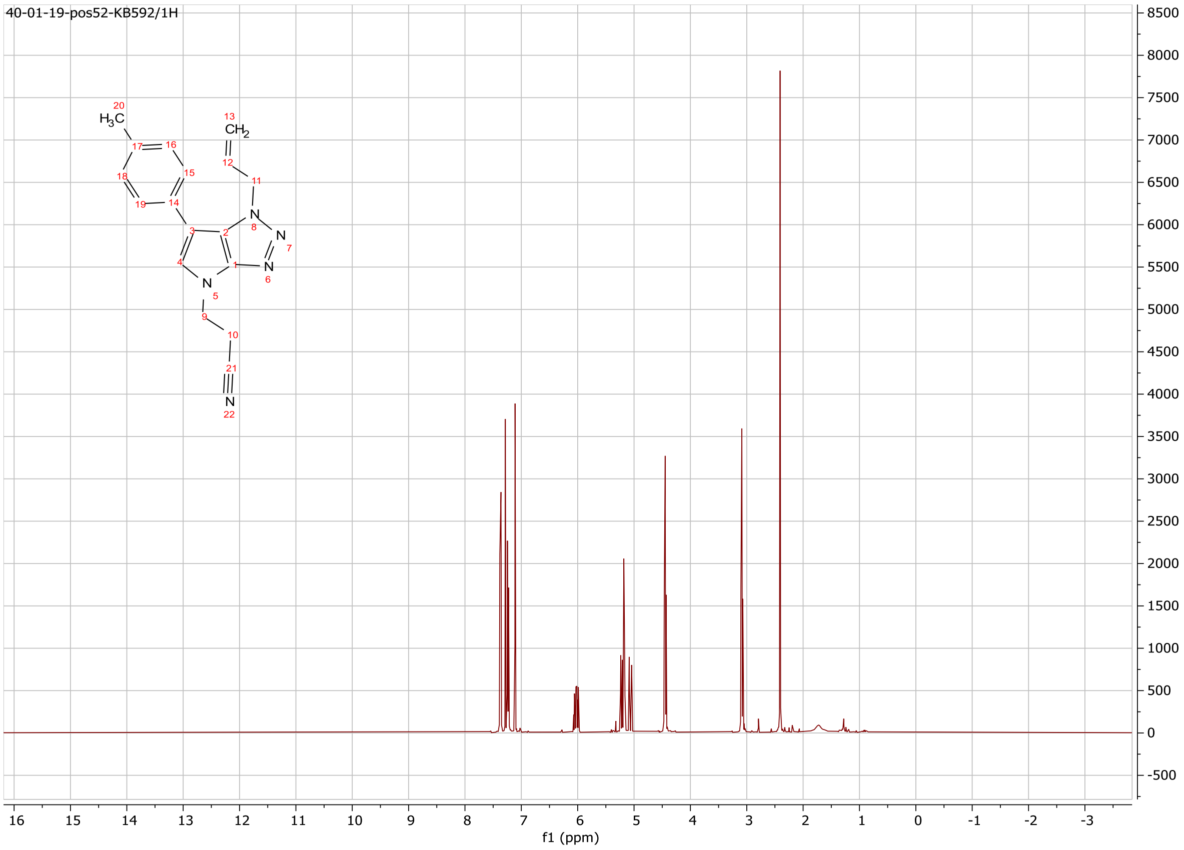
40-01-19-pos52-KB592/1H
f1 (ppm)

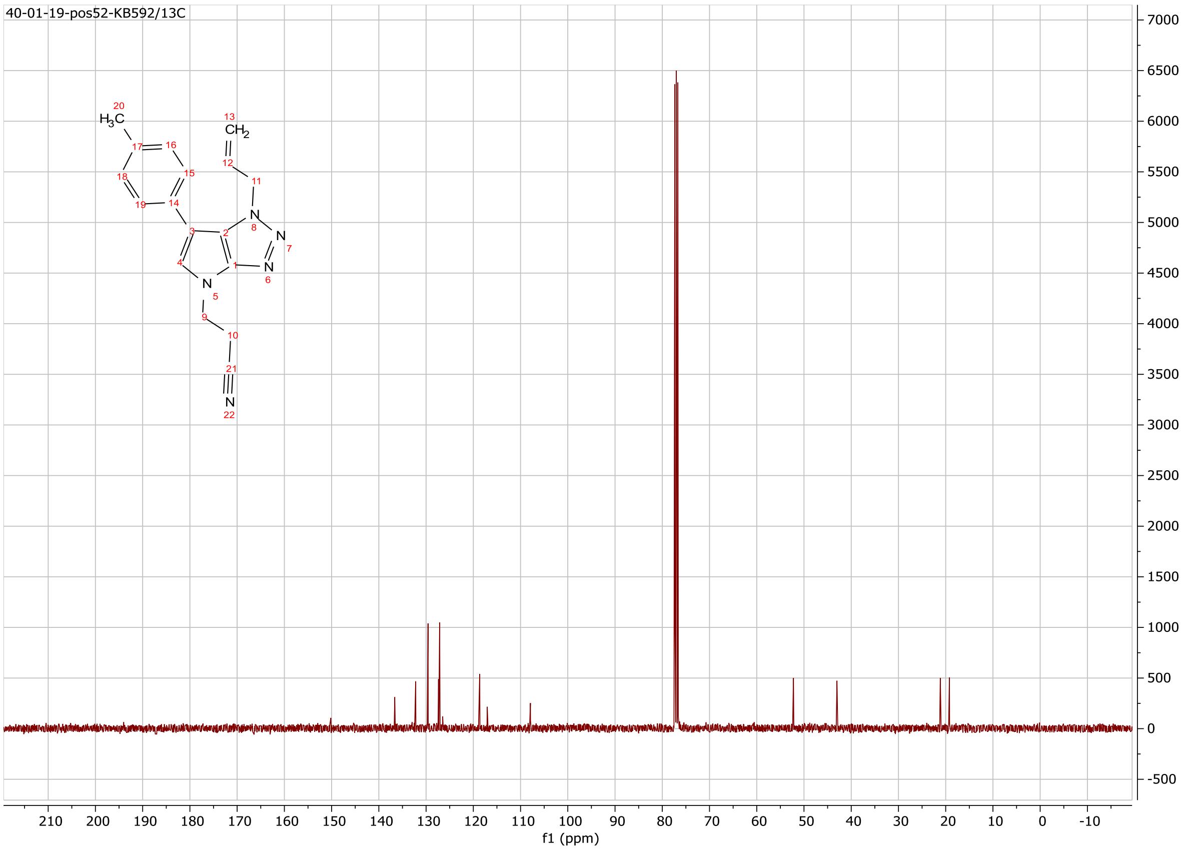
40-01-19-pos52-KB592/13C
f1 (ppm)

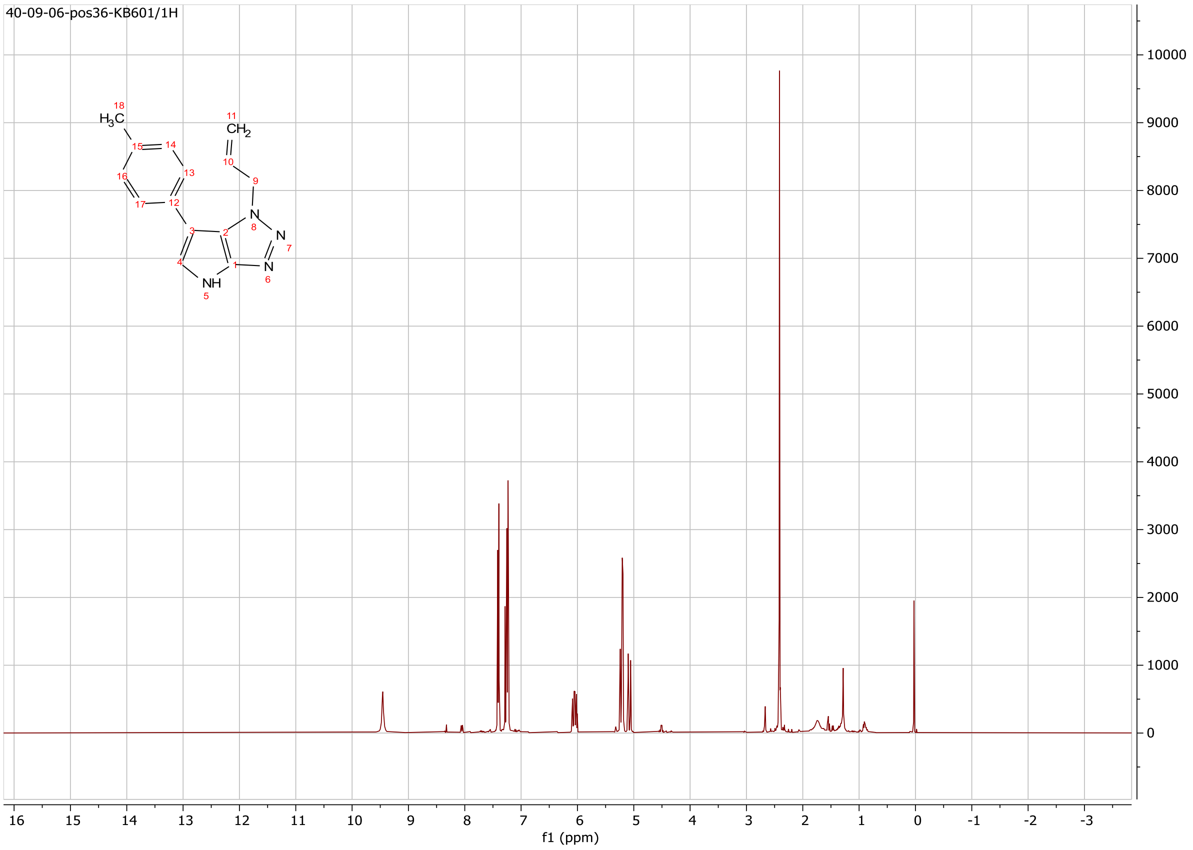
40-09-06-pos36-KB601/1H
f1 (ppm)

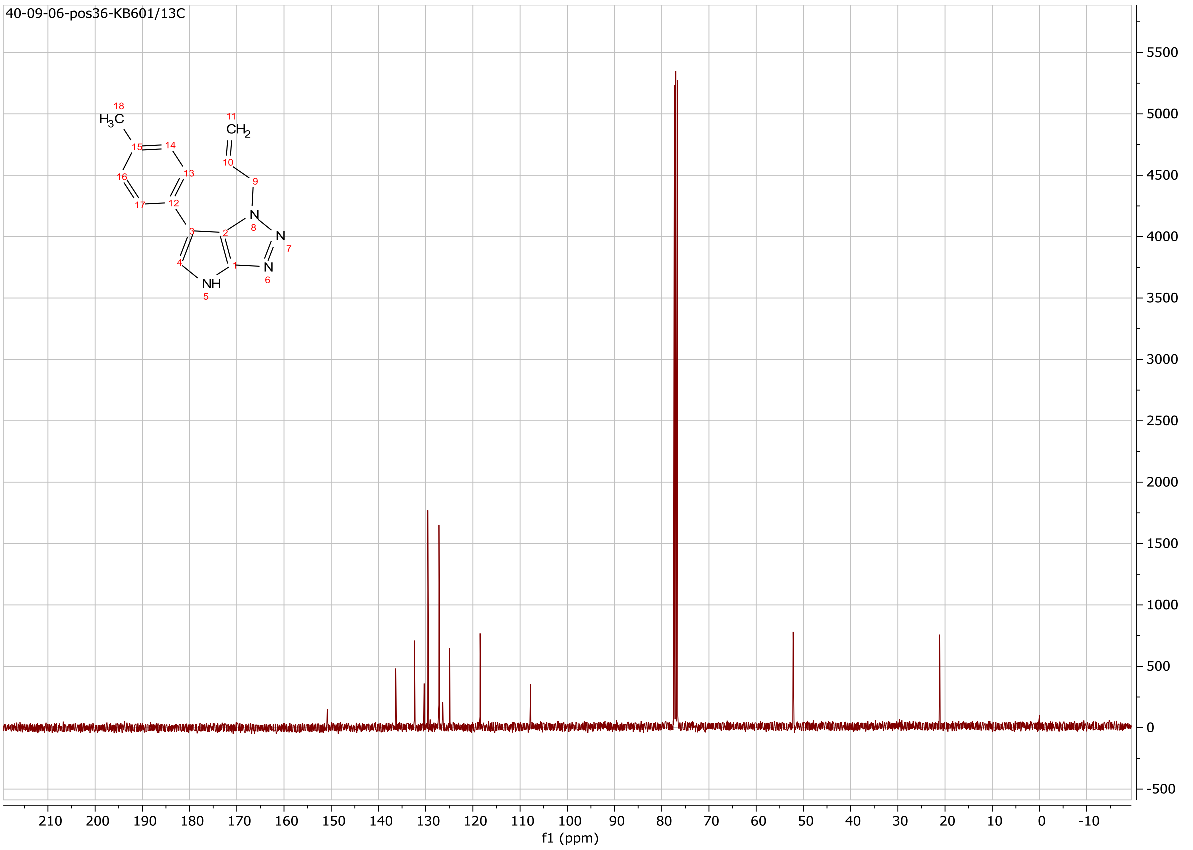
40-09-06-pos36-KB601/13C
f1 (ppm)

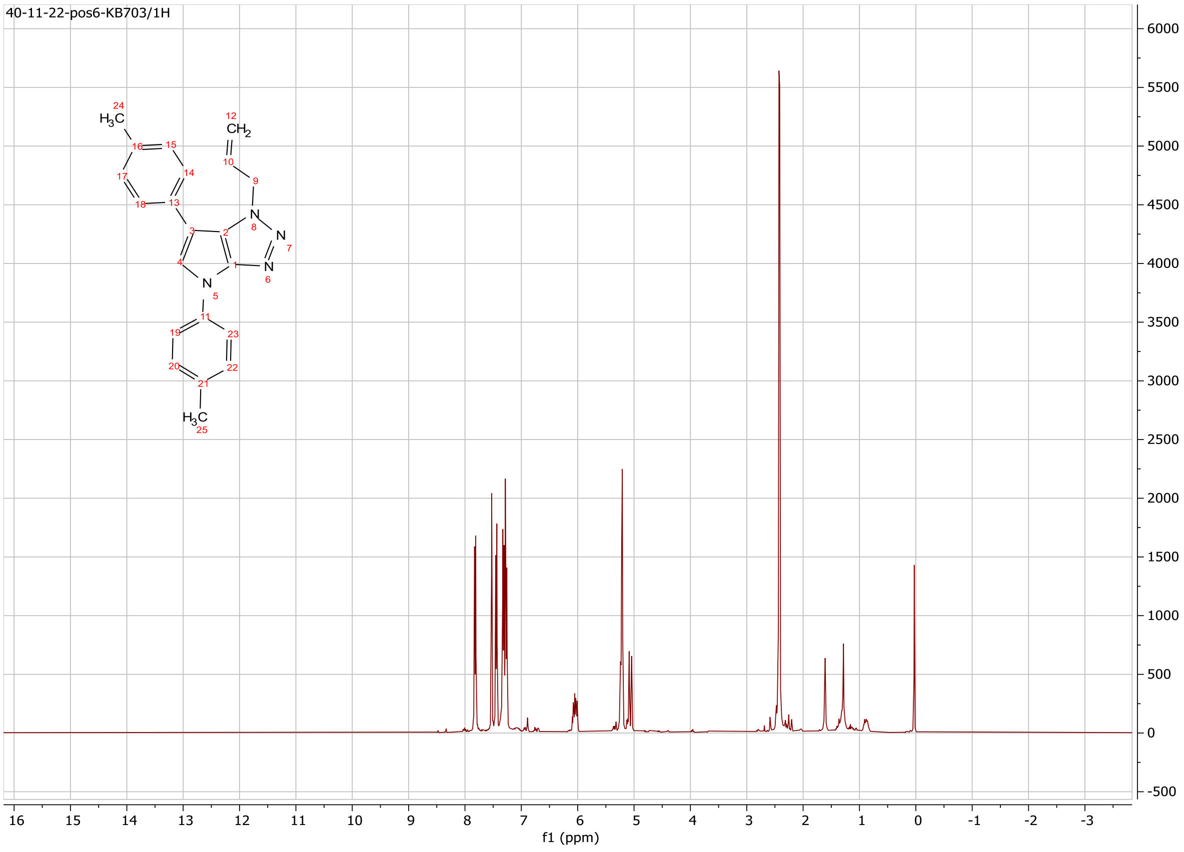
40-11-22-pos6-KB703/1H
f1 (ppm)

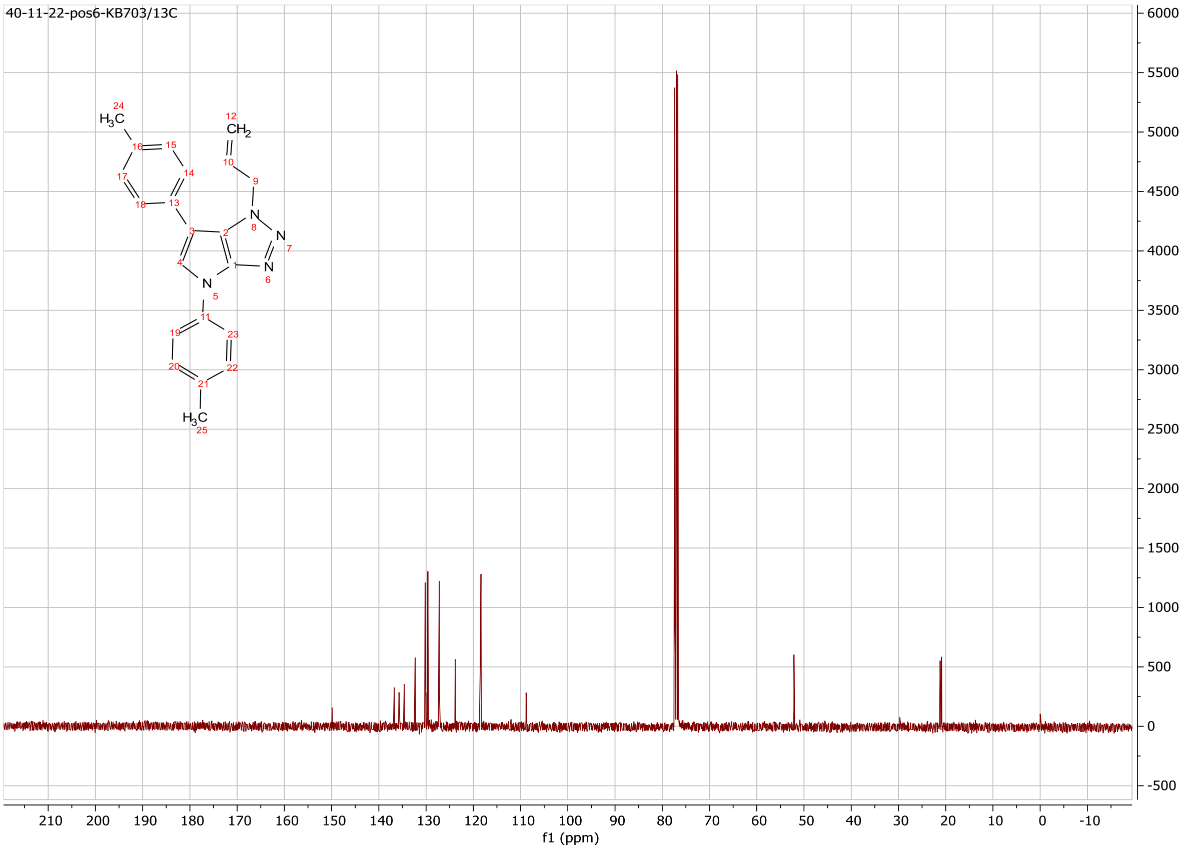
40-11-22-pos6-KB703/13C
f1 (ppm)

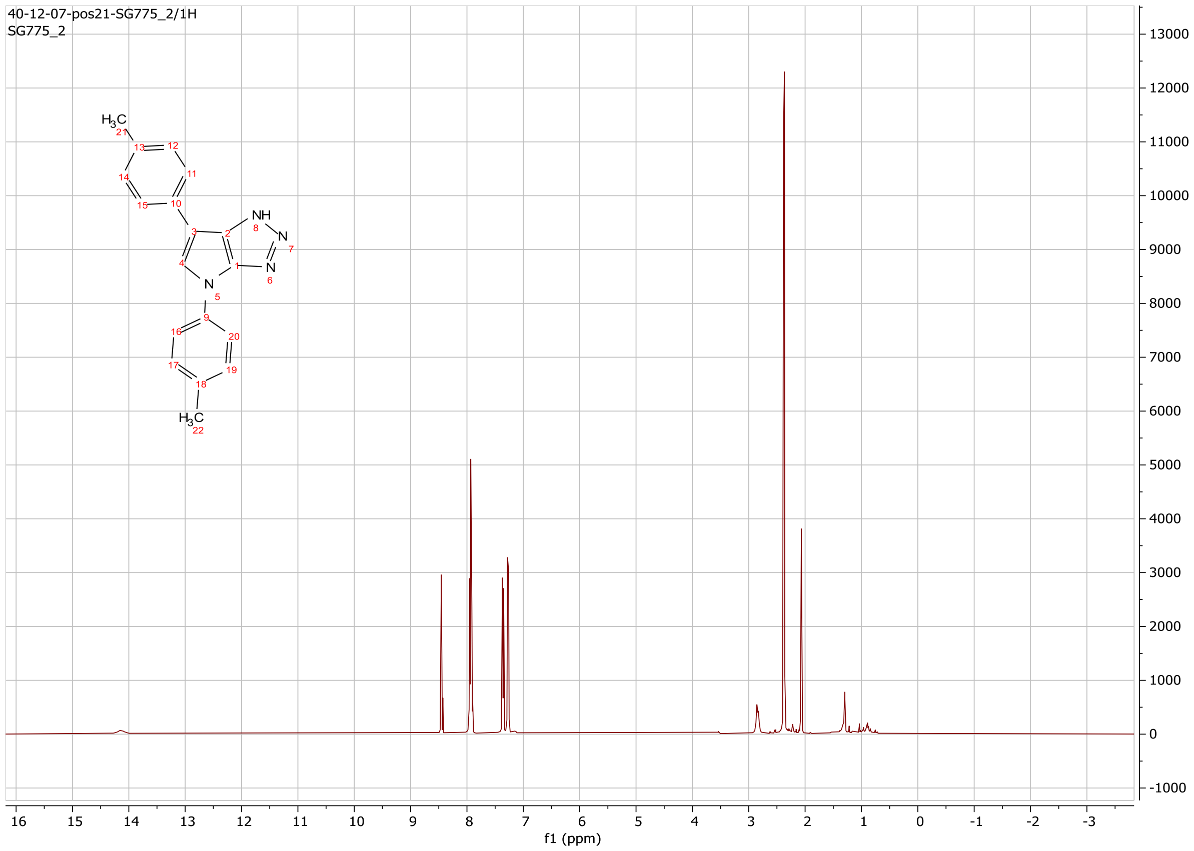
40-12-07-pos21-SG775_2/1H
SG775_2
f1 (ppm)

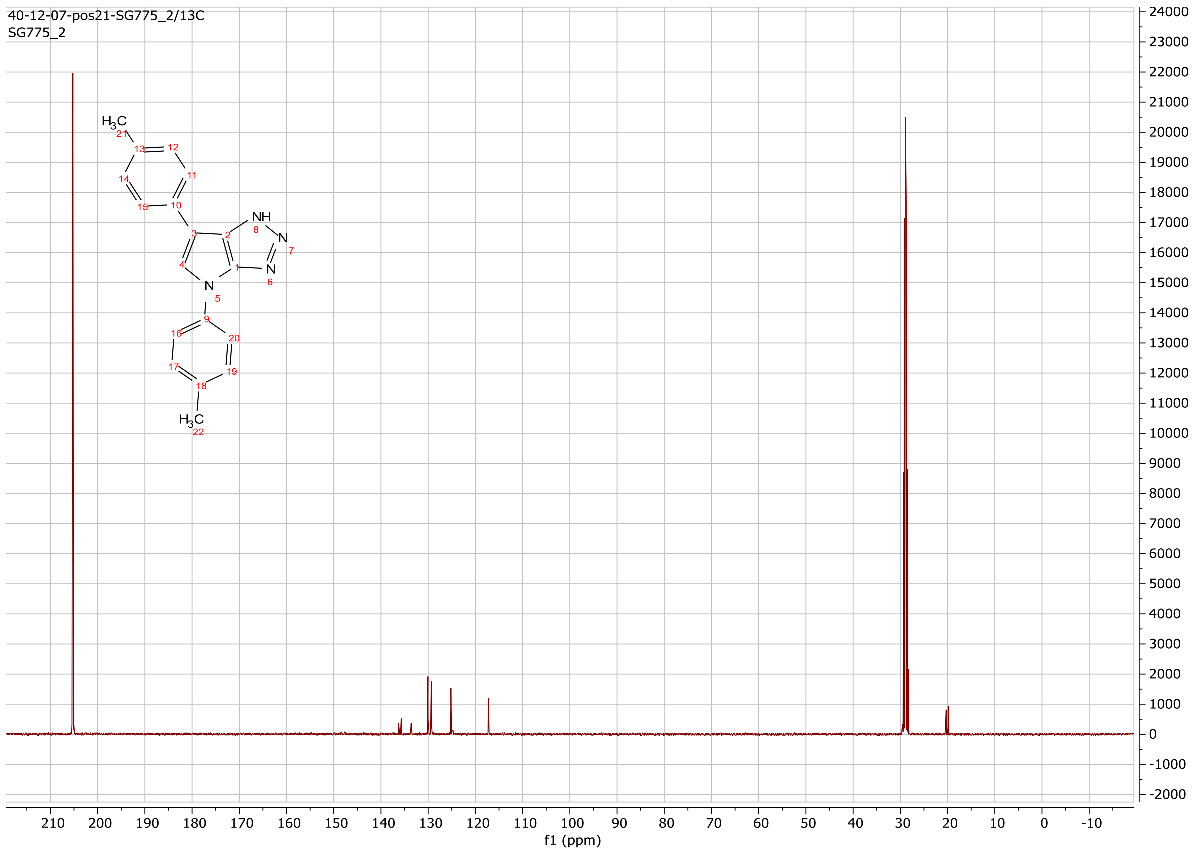
40-12-07-pos21-SG775_2/13C
SG775_2
f1 (ppm)

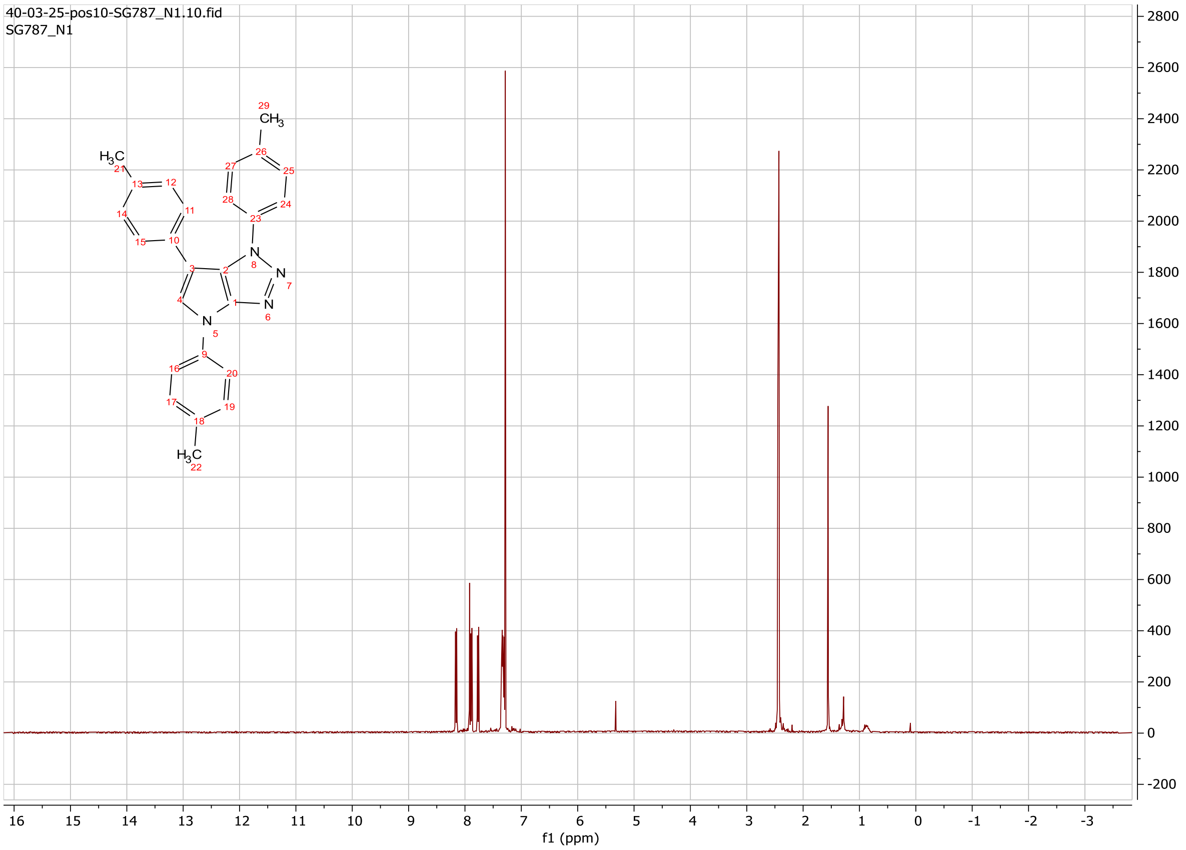
40-03-25-pos10-SG787_N1.10.fid
SG787_N1
f1 (ppm)

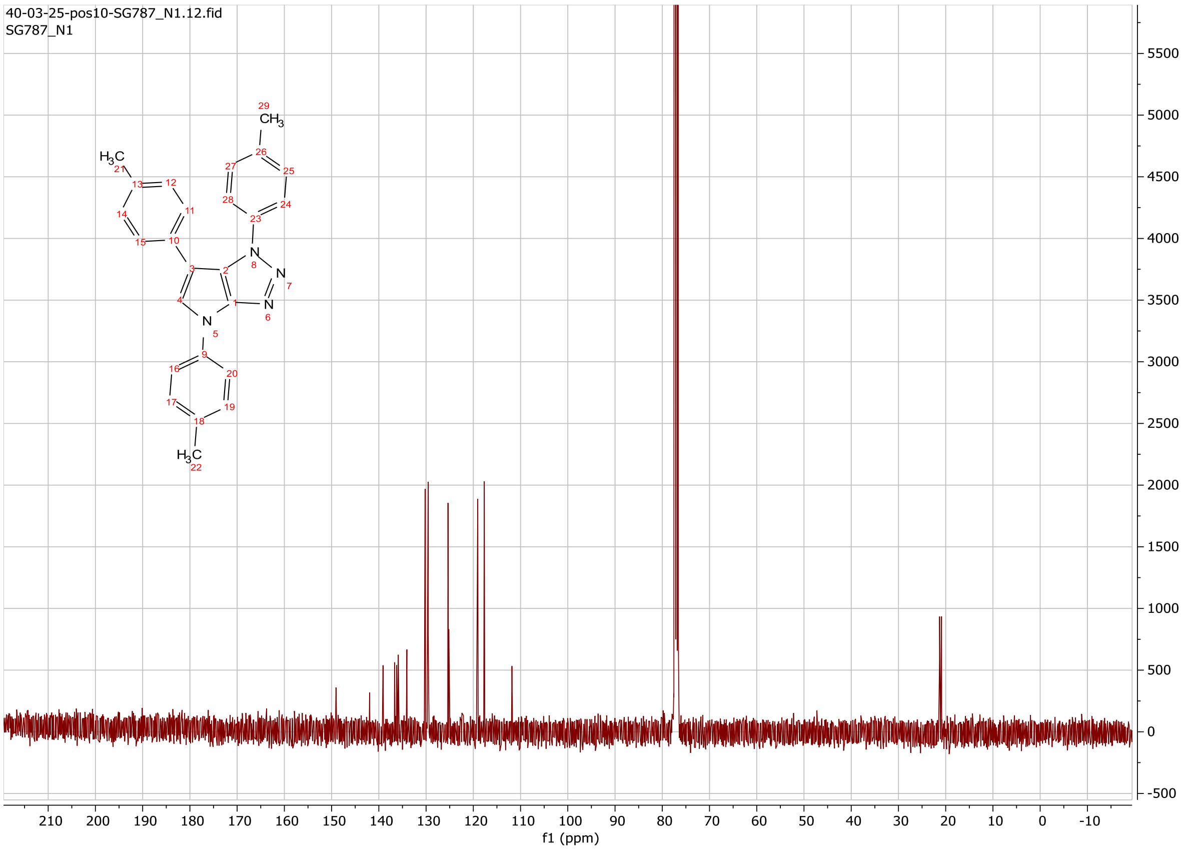
40-03-25-pos10-SG787_N1.12.fid
SG787_N1
f1 (ppm)
